# Sulfonyl Homoserine Lactones Are Tunable Probes to Inhibit the Quorum Sensing Receptor RhlR and Reduce Swarming Motility in *Pseudomonas aeruginosa*

**DOI:** 10.1021/acsinfecdis.5c00542

**Published:** 2025-09-17

**Authors:** Guadalupe Aguirre-Figueroa, Diana A. Morales Mijares, Isabel D. Cannell, Irene M. Stoutland, Helen E. Blackwell

**Affiliations:** Department of Chemistry, 5228 University of Wisconsin−Madison, 1101 University Avenue, Madison, Wisconsin 53706, United States

**Keywords:** autoinducer, chemical probes, Gram-negative bacteria, intercellular communication, LuxI/LuxR-type quorum sensing, virulence

## Abstract

We report non-native small molecules capable of inhibiting a key quorum sensing receptor—RhlR—in the opportunistic pathogen *Pseudomonas aeruginosa*. This protein is a member of the LuxR-type receptor family that is common to Gram-negative bacteria and recognizes *N-*acyl l-homoserine lactone (AHL) signals. RhlR has emerged as an increasingly important regulator of virulence pathways in *P. aeruginosa*, in concert with several other quorum sensing receptors, including LasR, QscR, and PqsR. Chemical inhibition of RhlR represents an approach to both study the role of RhlR in this quorum sensing signaling hierarchy and attenuate infection by *P. aeruginosa*. Small-molecule RhlR antagonists with high potencies and defined modes of action remain relatively scarce, however. AHL analogs with non-native acyl side chains represent a well-studied class of LuxR-type receptor modulators, but replacement of the native amide with alternate isosteres has been far less examined. In the current study, we investigated the activity of a series of sulfonamide AHL analogs as RhlR antagonists using transcriptional reporter assays. We identified *meta*-substituted aryl- and short-chain alkylsulfonyl lactones as potent and efficacious classes of synthetic RhlR antagonists. The activity profiles of the aryl derivatives were readily tuned via altering the position and electronics of aryl ring substituents. The most potent antagonists were active in *P. aeruginosa* and could significantly reduce its swarming motility, a key virulence determinant. Computational modeling revealed these compounds can be accommodated within the RhlR ligand-binding site and certain interactions may be required for high inhibitory potency.

## Graphical abstract

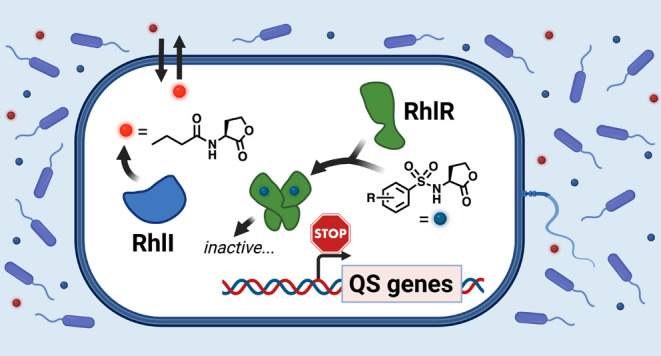

Quorum sensing (QS) is a chemical signaling process used by common bacteria to detect their population density and initiate group behaviors once a threshold population has been achieved.^1,2^ In general, QS allows for the controlled production of group-beneficial public goods. The best-characterized QS pathway in Gram-negative bacteria is dependent on the production and detection of *N*-acyl l-homoserine lactone (AHL) autoinducers.^3,4^ AHLs are produced by LuxI-type synthases and sensed by LuxR-type transcription factors in the cytoplasm (Figure 1). At low cell densities, AHLs are generated at low basal levels, but as the population density increases and dependent on environmental conditions, the local concentration and thus the intracellular concentration of AHL increase. Once a threshold concentration is achieved, AHLs will productively bind to their cognate LuxR-type proteins, after which the complex typically dimerizes, binds to DNA, and regulates expression levels of QS-associated genes. These genes are involved with diverse group behaviors, including biofilm formation, motility, conjugation, sporulation, bioluminescence, and virulence factor production.^1,5,6^ Many common bacterial pathogens use LuxI/LuxR-type QS to initiate colonization and infection, including *Pseudomonas aeruginosa*.^7,8^ Accordingly, interest in QS as a target to attenuate virulence has attracted considerable interest,^9,10^ attention that continues to increase due to the rapid rise of antibiotic-resistant strains.^11^

**Figure 1:**
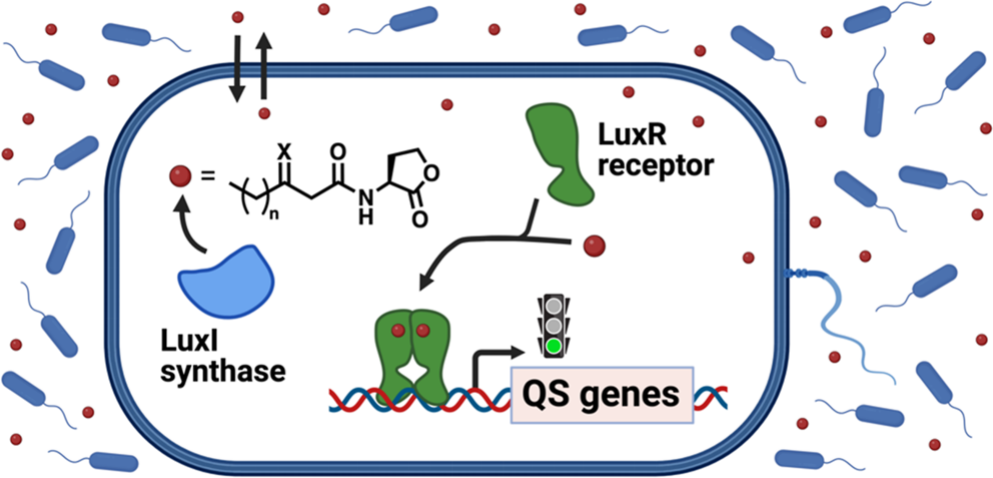
Simplified schematic of LuxI/LuxR-type quorum sensing (QS) in Gram-negative bacteria.

*P. aeruginosa* is a Gram-negative opportunistic pathogen that regulates many aspects of virulence through QS pathways.^7,12^ This bacterium is one of the leading causes of hospital-acquired infections and has been assigned a high-priority ESKAPE pathogen by the Infectious Diseases Society of America due to increased rates of resistance to standard antimicrobial therapies and infection mortality.^13^ *P. aeruginosa* utilizes a relatively complex QS system to regulate virulence, including two LuxI/LuxR-type protein pairs (Figure 2). The LasI/LasR and RhlI/RhlR pairs produce and respond to *N*-(3-oxo)-dodecanoyl l-homoserine lactone (OdDHL) or *N*-butanoyl l-homoserine lactone (BHL) signals, respectively.^14^ Additionally, an “orphan” LuxR-type receptor in *P. aeruginosa*, QscR, which lacks an associated LuxI-homologue, can bind to and is strongly activated by OdDHL.^15^ Together, a hierarchy is established within this QS system to regulate virulence factor production.^16^ For example, LasR regulates the production of elastase B and exotoxin A, as well as the Rhl system, through the regulation of the *rhlI* and *rhlR* genes. In turn, RhlR regulates the production of additional toxic exofactors, such as pyocyanin and HCN, and rhamnolipid biosurfactants. Interestingly, QscR has been proposed to be a negative regulator of both the Las and Rhl QS systems, but the exact mechanism is still being unravelled.^17^ A non-LuxR homologue, PqsR, also plays a role in tuning these interconnected LuxI/LuxR systems in *P. aeruginosa* via a quinolone signal (PQS).^18^

**Figure 2:**
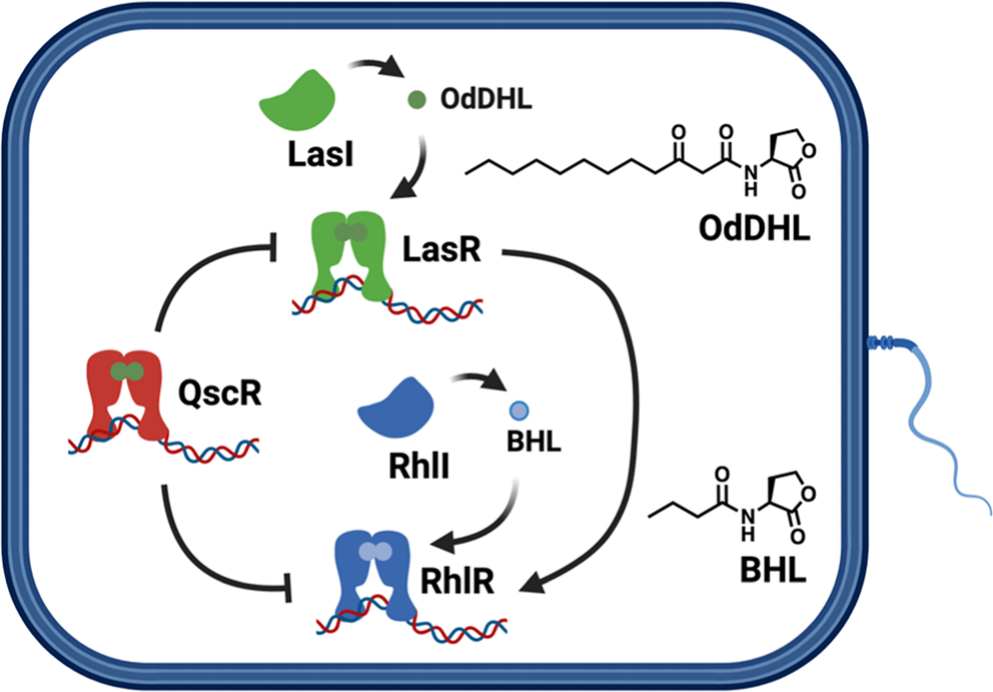
Schematic of the *P. aeruginosa* LuxI/LuxR-type QS network. Pointy-headed arrows indicate positive regulation; flat-headed arrows indicate negative regulation. LasR and QscR respond to OdDHL, while RhlR responds to BHL.

Most investigations into intercepting QS signaling in *P. aeruginosa* have focused on LasR due to its prominent role in the QS hierarchy. However, increasing reports have shown that RhlR plays a more prevalent role in *P. aeruginosa* virulence than previously believed, especially in chronic infections. For example, Dekimpe and Deziel have demonstrated that clinical isolates of *P. aeruginosa* with dysfunctional Las systems can still produce numerous virulence factors historically associated with LasR, including elastase B.^19^ In a related study, Cruz et al. reported a LasR-null isolate from a cystic fibrosis patient remained QS active via RhlR and highly virulent.^20^ This past work suggested that RhlR can replace LasR’s role under certain conditions, highlighting the complexity and unknowns of *P. aeruginosa* *’s* QS network.^7,12,16^ Recent reports by Paczkowski and others showing new connections between RhlR and the Pqs QS circuit (via PqsE) have begun to illuminate this complexity in *P. aeruginosa* and prompt further questions.^21–23^

For the past 25+ years, there has been considerable research into the development of chemical and biological strategies to modulate LasR and thereby study QS pathways in *P. aeruginosa*,^24–29^ but due to the emerging prominence of RhlR in *P. aeruginosa* infections, investigations have started to shift toward also targeting RhlR. Our laboratory and others have focused on developing small-molecule inhibitors and activators of RhlR. Early efforts by Suga and co-workers developed AHL analogs that exhibited largely agonistic behavior toward RhlR.^30^ In 2013, Bassler and co-workers reported a thiolactone analog (mBTL) that modulated Rhl-associated phenotypes^31^ and was found to strongly agonize RhlR.^26,32^ Our laboratory has since screened our in-house non-native AHL analog library for RhlR modulators and identified both efficacious agonists and antagonists.^33–36^ Certain of these compounds were shown to attenuate the production of pyocyanin and rhamnolipids in *P. aeruginosa*, two key virulence factors controlled by RhlR.^37^ An ongoing issue, however, with current AHL-derived RhlR antagonists (and for LuxR-type protein antagonists overall) is their moderate potencies, with the strongest antagonists having IC_50_ values in the low to mid micromolar regime (as measured typically in cell-based transcriptional reporter assays). This potency profile in RhlR is perhaps not surprising (assuming these close BHL analogs are competitive inhibitors), as BHL has an EC_50_ of ∼5–10 μM in *P. aeruginosa* RhlR reporter systems.^34^ Additional drawbacks of AHL-derived probes can include hydrolytic instability,^38,39^ enzymatic degradation,^38,39^ and active efflux (at least in *P. aeruginosa*).^40^ These liabilities are most pronounced for aliphatic AHLs. Non-AHL derivatives, including natural products and close analogs, have been reported to modulate RhlR; however, their potencies and/or selectivities are typically considerably lower than AHLs.^41–43^ These issues, when combined, motivate the continued search for alternative chemical scaffolds to yield potent RhlR antagonists.

To start to identify such classes and build from the structures of known active AHL-type RhlR modulators, we turned our attention to *N*-sulfonyl l-homoserine lactones (HLs). Isosteric replacement of an amide bond by a sulfonamide has been a widely used strategy in ligand design to reduce enzymatic cleavage by amidases while also improving properties like stability in water and increased receptor-binding ability through hydrogen bonding interactions.^44^ To date, replacement of the AHL amide with a sulfonamide has been explored in a relatively limited capacity in probe design (Figure 3).^45^ An initial report by Castang et al. investigated the activity of racemic alkyl (straight chain and 3-oxo), phenylethyl, and phenylpropyl sulfonyl HLs as QS modulators^46^ and ultimately demonstrated that certain analogs were capable of inhibiting LuxR from *Vibrio fischeri* at low micromolar levels. The first enantiomerically pure (l-isomer) alkyl sulfonyl HLs were reported by our laboratory; we found various of these analogs to be highly potent inhibitors of LuxR in *V. fischeri* and TraR in *Agrobacterium tumefaciens* yet inactive in LasR in *P. aeruginosa*.^47,48^ Kim et al., Yadav et al., and Porras et al. have further explored aryl sulfonyl HLs as QS inhibitors by preparing enantiomerically pure benzenesulfonyl HLs either with or without *para* substituents.^49–51^ These compounds were reported to inhibit TraR in *A. tumefaciens*, pigment production in *Chromobacterium violaceum* (presumed via CviR), and biofilm formation in *P. aeruginosa* (presumed via LasR), by up to ∼70% at midnanomolar to high micromolar levels. Related *N*-(4-aminobenzene) sulfonyl HL derivatives were studied by Sun et al. and Zhao et al.,^52,53^ revealing certain *N-*amidoaryl and heterocyclic sulfonyl HLs could inhibit pigment production in *C. violaceum*, again with IC_50_ values in the low micromolar range. These past studies highlight the potential of the sulfonyl HL scaffold for modulation of a range of LuxR-type receptors. That said, only a limited set was examined overall and in disparate assays, and the connections among compound structure, LuxR-type receptor specificity (if any), efficacy, and potency remain unknown. In the current study, we sought to start to define this chemical space using RhlR as a target, in view of its relevance to QS signaling in *P. aeruginosa*, using a focused set of sulfonyl HLs.

**Figure 3:**
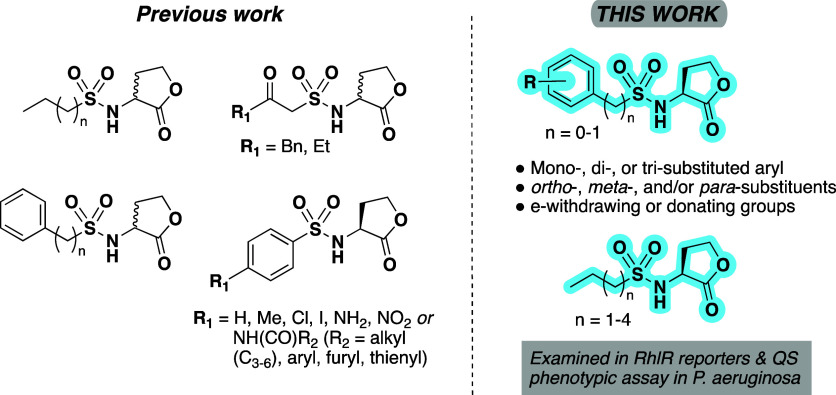
Chemical structures of previously reported sulfonyl HLs (left) and the scaffolds described in this work (right).

Herein, we report the design, synthesis, and biological characterization of a library of sulfonyl HLs with the goal of uncovering new modulators for RhlR in *P. aeruginosa* and structure–activity relationship (SAR) trends that are important for antagonism in this receptor. Forty-five aryl and alkyl sulfonyl HLs were synthesized and screened using an *Escherichia coli* RhlR reporter system. Several classes of RhlR antagonists were identified, with efficacies (% inhibition) and potencies (IC_50_ values) that could be widely tuned by altering the positions, electronics, and sterics of the substituents on their aryl tails (Figure 3). A small set of partial agonists also were discovered, suggesting that RhlR can be both inhibited and activated by the sulfonyl HL class. The most potent antagonists maintained their activity in a *P. aeruginosa* RhlR reporter system and could significantly reduce the swarming motility of wild-type *P. aeruginosa* in a RhlR-dependent manner. Computational docking studies support these compounds being accommodated in the ligand-binding site of RhlR and reveal interactions that may impact activity. Overall, this work serves to further underscore the value of the sulfonyl HL scaffold for LuxR-type receptor modulation and help define a new chemical scaffold to alter RhlR—and thereby QS—activity in *P. aeruginosa*.

## Results and Discussion

### Library Design and Synthesis

The first set of sulfonyl HL scaffolds selected for study were inspired by *N-*phenylacetyl HLs (PHLs) with substituents in the *para* position, as these compounds have been shown to be among the most active and potent antagonists of RhlR activity (e.g., PHLs **C19** and **C20** in Figure 4).^33^ We designed a series of benzyl sulfonyl HLs (BnSHLs) that effectively replaced the PHL amide with a sulfonamide and contained a range of aryl substituents. To examine the impact of removing the methylene group in the BnSHL tail, we synthesized a series of benzenesulfonyl HL homologues (BSHLs) with a variety of mono-, di-, and trisubstitutions on the aryl ring. Most of these substituents were halogens (in part due to ready commercial availability), but we also explored several other electron-withdrawing and -donating groups. We also generated a small set of alkyl sulfonyl HLs (ASHLs) to complement prior work by Castang et al.^46^ and our own^47,48^ (see above) but focusing on shorter tail lengths better matched to that of the native BHL ligand. All compounds were synthesized via coupling L-HL to various sulfonyl chlorides in acceptable yields for screening (30–60%; see Methods) and purified to >95% purity prior to biological testing (see Supporting Information for full characterization data). We focus our discussion below on the set of compounds with the most interesting activity trends in RhlR (Figure 4); the full set of structures synthesized is shown in Figure S1.

**Figure 4:**
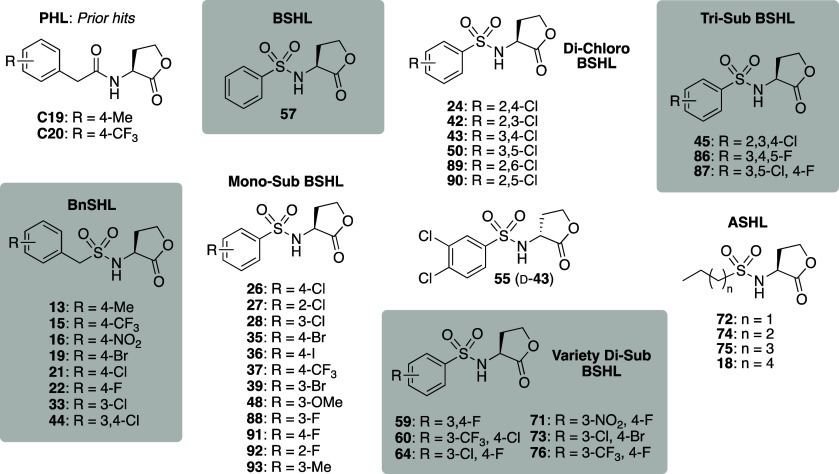
Chemical structures of the sulfonyl HLs examined in this study and selected known RhlR antagonist controls (**C19** and **C20**).^33^ Compounds listed numerically by the substructure class. ASHLs **74**, **75**, and **18** were part of prior studies by our lab^47,48^ but examined in RhlR here.

### Biological Activity Assessment

To start, the sulfonyl HLs were evaluated for their ability to antagonize RhlR using a previously described *E. coli* strain (JLD271) harboring a RhlR expression plasmid and a *rhlI* reporter plasmid.^34^ This *E. coli* reporter produces RhlR, and protein activity in the presence of an added small molecule can be measured via the production of β-galactosidase as quantified via luminescence (see Methods).^54^ Compounds were screened in competition against RhlR’s native ligand, BHL (at its EC_50_ value in this strain, 10 μM), over a range of concentrations to generate antagonism dose–response curves (see Supporting Information) and calculate IC_50_ values (listed in Table 1). A small set of compounds was also screened for RhlR agonism in the *E. coli* reporter; the agonism assay format was identical to the antagonism assay, except no BHL was added.

**Table 1:** IC_50_ and Efficacy Data for Compounds in the *E. coli* RhlR Reporter Strain^a^ ^,^ ^g^

compound	aryl/alkyl substituents	IC_50_ (μM)	95% CI (μM)^b^	max. RhlR inhibition (%)^c^
PHLs				
**C19** ^d^	4-CH_3_	5.46	4.05–7.14	89
**C20** ^d^	4-CF_3_	10.3	7.30–14.5	93
BnSHLs				
**13**	4-Me	918	740–1150	90
**15**	4-CF_3_	--^e^	--	35^f^
**16**	4-NO_2_	1290	820–2066	97
**19**	4-Br	348	237–510	77
**21**	4-Cl	219	156–303	99
**22**	4-F	205	146–287	94
**33**	3-Cl	125	104–151	99
**44**	3,4-Cl	205	136–308	82
BSHL				
**57**	--	715	481–1070	93
Mono-Sub BSHLs				
**26**	4-Cl	452	358–575	96
**27**	2-Cl	545	359–835	93
**28**	3-Cl	143	107–192	97
**35**	4-Br	166	118–232	99
**36**	4-I	295	202–428	99
**37**	4-CF_3_	333	265–417	99
**39**	3-Br	29.9	20.9–42.7	90
**48**	3-OMe	210	149–296	88
**88**	3-F	120	92.1–156	92
**91**	4-F	301	190–463	97
**92**	2-F	863	534–1460	91
**93**	3-Me	127	92.3–177	95
Di-Chloro BSHLs				
**24**	2,4-Cl	1590	1210–2100	99
**42**	2,3-Cl	67.8	51.0–89.9	95
**43**	3,4-Cl	54.3	38.7–75.9	99
**50**	3,5-Cl	--	--	50^f^
**55**	3,4-Cl (D-**43**)	195	143–264	99
**89**	2,6-Cl	>1000	--	86
**90**	2,5-Cl	1100	761–1620	92
Variety Di-Sub BSHLs				
**59**	3,4-F	87.8	67.5–114	87
**60**	3-CF_3_, 4-Cl	93.1	65.5–133	90
**64**	3-Cl, 4-F	31.3	21.6–45.6	90
**71**	3-NO_2_, 4-F	257	200–328	62
**73**	3-Cl, 4-Br	118	85.7–163	98
**76**	3-CF_3_, 4-F	69.4	45.2–106	84
Tri-Sub BSHLs				
**45**	2,3,4-Cl	1030	617–1729	80
**86**	3,4,5-F	24.5	16.9–35.3	80
**87**	3,5-Cl, 4-F	68.0	47.3–99.1	85
ASHLs				
**72**	C_3_	--	--	45^f^
**74**	C_4_	35.2	20.0–61.4	70
**75**	C_5_	20.6	13.5–31.2	84
**18**	C_6_	36.4	25.7–51.3	77

a See Methods and Supporting Information for details of assay and data analysis.

b CI = confidence intervals for IC_50_ values.

c Max. inhibition calculated based on treatment with BHL at 10 μM (set to 0%) and media control (set to 100%).

d Previously reported RhlR antagonists from our lab.^33^

e “--” = not calculated as inhibition was ≤50% at max. conc. tested (5 mM).

f Compound displays partial agonism (dose–response curve in Supporting Information) or likely partial agonism (**72**).

g Compound groupings (down the left column) follow discussion in text.

### Halogen-Substituted BnSHLs Consistently Inhibit RhlR Activity with Modest Potencies

We examined a range of BnSHLs with aryl rings containing electron-withdrawing groups (−CF_3_, –NO_2_), halogens (–F, –Cl, –Br), and electron-donating (–Me) groups (Figure 4), to both explore their activity profiles and collect SARs for RhlR antagonism to compare to their PHL analogs. Compound **15** (4-CF_3_ BnSHL) displayed only a low inhibitory activity (35%), even at high concentrations (Table 1). We were interested to determine if **15** instead displayed agonistic activity in RhlR (and thus could be classified as a partial agonist).^24^ Compound **15** was indeed capable of about 30% RhlR activation at high concentrations (see Supporting Information). This activity profile contrasted significantly with its PHL analog (4-CF_3_ PHL, **C20**), which displayed 93% antagonism and an IC_50_ value of 10.3 μM, indicating introduction of the sulfonamide linker in at least this PHL scaffold negatively impacted inhibitory activity in RhlR.

The other BnSHLs investigated all displayed moderate to high RhlR antagonism, albeit with differences in potency (Table 1). Compounds **13** (4-Me BnSHL) and **16** (4-NO_2_ BnSHL) were capable of strong RhlR inhibition (>90%) yet had very high IC_50_ values at 918 μM and 1290 μM, respectively (∼170-fold less potent than parent 4-Me PHL **19**). The remaining three BnSHLs all contained *para*-halogens (4-Br BnSHL, **19**; 4-Cl BnSHL, **21**; and 4-F BnSHL, **22**) and were moderate to very strong RhlR inhibitors (77–99%) and approximately 3-fold more potent in RhlR than **13** or **16**. Interestingly, shifting the chloro-substituent to the *meta*-position (as in 3-Cl BnSHL, **33**) increased potency relative to 4-Cl analog **21** (125 vs 219 μM, respectively). The dichloro analog (3,4-di-Cl BnSHL, **44**) was slightly less efficacious (82%) but comparable in potency to the monochloro BnSHL analogs. These results indicate that halogen-substituted BnSHLs antagonize RhlR activity, and the inclusion of a *meta*-chloro substituent onto their aryl rings can improve potency relative to other electron-withdrawing and -donating groups. Overall, while the tested BnSHLs had reduced potencies in RhlR compared to the PHL analogs, several exhibited stronger efficacies (>95% inhibition), suggesting that sulfonyl HLs have promise as RhlR inhibitors.

### Monosubstituted BSHLs with *meta*-Halogens Are RhlR Antagonists

We next examined the activities of BSHLs in RhlR. As an initial comparison, the unsubstituted parent BSHL (**57**; Figure 4) displayed a relatively weak potency (715 μM) but strong inhibition (93%; Table 1). This inhibitory activity trends with results of Kim et al. showing **57** can antagonize other LuxR homologues by ∼50% (see above).^49^ Turning to the monosubstituted BSHLs, all these compounds were capable of strong to near complete RhlR inhibition (88–99%) and had potencies either similar to the parent BSHL or stronger. These potencies varied based on both the substituent and its position. For example, the 3-chloro-BSHL (**28**) was 3-fold more potent than either the 4-chloro or 2-chloro BSHLs (**26** and **27**, respectively) and comparable in potency to the 3-chloro BnSHL (**33**) with an IC_50_ value of 143 μM. The fluoro-BSHL series showed a similar trend, with the 3-fluoro BSHL (**88**) two- to seven-fold more potent than the 4-fluoro- or 2-fluoro-BSHLs (**91** and **92**, respectively) and comparable in potency to the 3-chloro BSHL (**28**). The bromo-BSHL series continued this trend favoring the *meta*-substituted isomer, with 3-bromo BSHL (**39**) almost 6-fold more potent than the 4-bromo BSHL (**35**) and with the lowest IC_50_ value in this class (29.9 μM). Electron-donating groups (–Me, –OMe) at the *meta*-position yielded weaker potencies, as did a larger electron-withdrawing group at the *para*-position (–I, –CF_3_), compared to **39**. These data indicated that *meta*-halogen monosubstitution in BSHLs results in strong RhlR antagonism, with bromine giving optimal results among the tested halogens.

### Dichloro-BSHLs Reveal a Preferred Aryl Substitution Pattern for RhlR Antagonism

We proceeded to investigate the effects of adding multiple halogens to BSHLs on RhlR antagonism. A series of disubstituted BSHLs with chloro-substituents was examined to start (due to synthetic accessibility relative to the bromo series; Figure 4) and compared to 3-Cl BSHL (**28**). In these new compounds, two *meta-*substituted dichloro isomers (2,3-di-Cl BSHL, **42**, and 3,4-di-Cl BSHL, **43**) had over 2-fold lower IC_50_ values than 3-Cl BSHL (**28**) (Table 1). In turn, the dichloro isomers that either lacked *meta*-chloro substituents (2,4-di-Cl-BSHL, **24**, and 2,6-di-Cl-BSHL, **89**) or had *meta*- and *ortho*-chloro substituents in a 2,5-pattern (2,5-di-Cl BSHL, **90**) had higher IC_50_ values relative to **28**. Interestingly, the isomer with two *meta*-chloro substituents (3,5-Cl-BSHL, **50**) displayed partial agonist behavior, only reducing RhlR activity to about 50%, indicating that the benefits of *meta* substitution were not additive for antagonism. Nevertheless, all of the other dichloro-BSHLs examined were capable of strong RhlR antagonism (86–99% inhibition). These results further underscore the value of *meta* substitutions for BSHL potency against RhlR and also reveal that dichlorosubstitutions can be either beneficial or detrimental to potency and efficacy.

### BSHL Stereochemistry Impacts RhlR Antagonism Potency but Not Efficacy

We were curious whether native l-stereochemistry was important for BSHL antagonistic activity in RhlR, and we investigated the activity of 3,4-di-Cl BSHL **55**, the d-stereoisomer of **43** (Figure 4). Prior studies have shown that d-stereoisomers of both native and non-native AHLs,^55^ including ASHLs,^56^ have reduced activities in LuxR-type receptors, a trend that has been interpreted as their inability to engage in specific interactions that engender receptor activation or inhibition. We observed that BSHL **55** was approximately 3-fold less potent than the l-stereoisomer **43** (195 μM vs 54.3 μM, respectively) yet still was capable of comparably strong RhlR inhibition (99%; Table 1). This drop in potency aligns with past work and supports the premise that native HL stereochemistry is important for high potency in RhlR antagonism by BSHLs. The matching high efficacies of the two BSHL stereoisomers, however, were unexpected and to our knowledge not previously observed in AHL analogs. We return to this result below.

### Additional Substituted BSHLs Confirm Substituent Patterns That Garner Strong RhlR Antagonism

Next, we examined a series of six disubstituted BSHLs to identify other substituent combinations that could further enhance RhlR antagonistic activity in this analogue class (Table 1). We focused on BSHLs with electron-withdrawing substituents in the *meta* and *para* positions, as this 3,4-pattern yielded the highest potency and efficacy in our analysis of dichloro BSHLs above. The 3,4-di-F (**59**), 3-CF_3_, 4-Cl (**60**), and 3-CF_3_, 4-F (**76**) BSHLs had potencies comparable to those of 3,4-di-Cl BSHL (**43**) but slightly weaker efficacies (84–90%) overall. Replacing 3-CF_3_ with a nitro group (in 3-NO_2_, 4-F BSHL **71**) reduced both potency and efficacy markedly. One analog, 3-Cl, 4-Br BSHL (**73**), was over 2-fold less potent than **43** yet maintained similar efficacy (98%), indicating that a larger *para* substituent alongside the *meta*-chloro substituent was deleterious for RhlR inhibition. Conversely, installation of a smaller substituent in the *para* position (as in 3-Cl, 4-F BSHL, **64**) gave a 2-fold higher trending potency than 3,4-di-Cl BSHL (**43**).

A set of trisubstituted BSHLs were also examined to determine if more than two substituents were beneficial or detrimental to RhlR inhibitory activity, again focusing on halogen substituents (Table 1). The addition of a 2-chloro in 2,3,4-tri-Cl BSHL (**45**) reduced potency by at least 2 orders of magnitude (IC_50_ ∼1000 μM, with 80% activity) relative to 3,4-Cl BSHL (**43**). In contrast, addition of another halogen in the 5 position (as in 3,4,5-F BSHL, **86**, and 3,5-Cl-4F BSHL, **87**) gave an over 2-fold higher or no change in potency relative to 3,4-Cl BSHL (**43**), respectively, with only a modest reduction in efficacy (80–85% activity). These results suggest that trihalosubstituted BSHLs with both *ortho* and *para* substitutions (like **45**) are disfavored for RhlR antagonism and are congruent with the low potency of 2,4-dichloro BSHL (**24**). Likewise, the potency trends for the trihalosubstituted BSHLs with both *meta* and *ortho* substitutions (like **86**) are consistent with the higher relative potencies of 3,4-halo BSHLs overall.

### ASHLs Are Potent and Efficacious Inhibitors of RhlR

As our final test compounds, we surveyed a small set of ASHLs (Figure 4) for RhlR antagonism with acyl chains ranging from three to six carbons, a length range chosen to mimic RhlR’s native ligand BHL. The C_4_, C_5_, and C_6_ ASHLs (**74**, **75**, and **18**, respectively) had comparable inhibitory potencies (∼28 μM on average) that were similar to the most potent BSHLs in this study; the C_5_ and C_6_ derivatives displayed higher efficacies (77–84%) relative to the C_4_ analog (70%) but weaker overall than the top BSHLs. Interestingly, the C_3_-AHSL **72** with a four-atom tail length analogous to BHL was only capable of 45% inhibition at the highest concentration tested (and most likely is a weak partial agonist like **15** and **50**). These findings indicate that ASHLs with tails at least one atom longer than BHL are capable of RhlR antagonism.

### Activity of RhlR Antagonists in *P. aeruginosa* Reporter Assays

We were interested in determining whether these new RhlR antagonists identified in an *E. coli* RhlR reporter maintained their activity in the native organism, *P. aeruginosa*. We tested a set of some of the most active RhlR inhibitors identified above (BSHLs **39**, **43**, and **59**; ASHL **75**) in a *P. aeruginosa* mutant (PA14 Δ*rhlI*Δ*lasI*) that lacks functional AHL synthases and contains a reporter plasmid that allows RhlR activity to be measured via the production of GFP (as quantified via fluorescence; see Methods). Antagonism of RhlR was measured in the presence of BHL and OdDHL (the latter signal was needed to induce the production of RhlR via LasR). All four compounds were found to moderately inhibit RhlR in this *P. aeruginosa* strain (**39** (47%), **43** (59%), **59** (49%), and **75** (67%)) at the highest concentrations tested but exhibited substantial losses in potency (IC_50_ > 1 mM) relative to the *E. coli* RhlR reporter (see Supporting Information for dose–response curves). This result was not unexpected, as previous studies in our laboratory and others have shown that LuxR-type protein modulator efficacy and potency are commonly reduced in *P. aeruginosa* vs *E. coli* *.* ^24^ The reasons for this reduction in activity are likely multifold, but we believe that they may stem from the low permeability of *P. aeruginosa* to small molecules, its lower (native) level of RhlR expression,^57^ its abundance of efflux pumps,^40^ and/or the cross-interaction of compounds with other targets (LasR, QscR, and potentially others) that then regulate RhlR.^36^ Preliminary results indicate that cross reactivity in at least LasR and QscR is not a major reason for their lower activity in *P. aeruginosa* (data not shown). Nevertheless, these reporter data for sulfonyl HLs **39**, **43**, **59**, and **75** in *P. aeruginosa* indicate that these compounds are capable of antagonizing RhlR activity in the native host.

### Lead RhlR Antagonists Inhibit Swarming Motility in *P. aeruginosa*

We next evaluated the ability of the four sulfonyl HLs **39**, **43**, **59**, and **75** capable of inhibiting RhlR in the *P. aeruginosa* reporter to reduce swarming motility in a wild-type *P. aeruginosa* strain (PAO1). Swarming is a rapid, group-coordinated movement of a bacterial population across a semisolid surface, linked to increased expression of virulence factors and increased antibiotic resistance^58^ and regulated in *P. aeruginosa*, at least in part, by the Rhl system.^59^ The results of these phenotypic assays are shown in Figure 5 (see Methods for protocol). The swarming ability of PAO1 in the presence of vehicle (DMSO) served as a positive control (set to 100%). As a key negative control, we examined the swarming ability of a PAO1 Δ*rhlR* mutant in the presence of the vehicle and observed a 25% motility reduction relative to the positive control (set to 0%). Treatment of PAO1 with BSHLs **39**, **43**, and **59** (at 500 μM) yielded a 60% reduction in swarming activity relative to the controls, while treatment with ASHL **75** (at 250 μM due to solubility limits in this assay format) yielded a 30% reduction in swarming. The ability to reduce swarming in PAO1 with these compounds supports the antagonistic trends observed in the RhlR reporter assays above and demonstrates the ability of these AHL analogs to reduce a *rhl*-dependent phenotype in wild-type *P. aeruginosa*.

**Figure 5:**
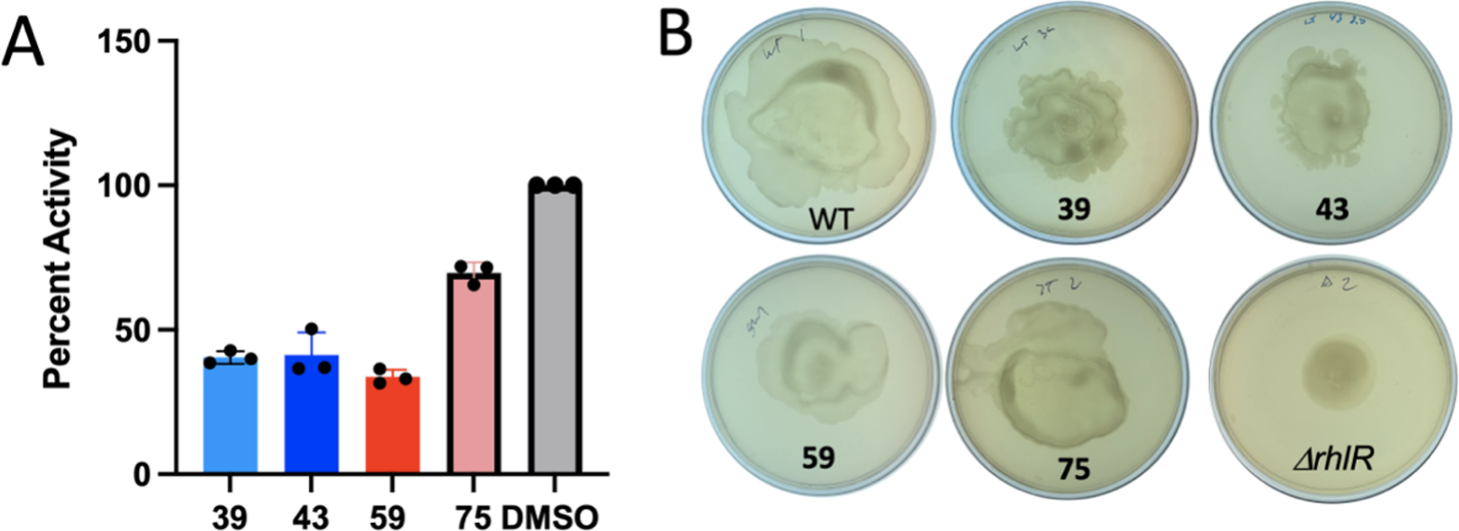
Swarming motility assay data in *P. aeruginosa*. (A) Plot of swarming activity in *P. aeruginosa* PAO1 treated with BSHLs (**39**, **43**, and **59**; at 500 μM) and ASHL (**75**; at 250 μM [due to solubility limitations]). Area of the colony after 42 h incubation was used to calculate swarming activity (see Methods). Positive control = PAO1 treated with vehicle (DMSO, 100%). Negative control = PAO1 Δ*rhlR* treated with vehicle (0%). Error bars = standard deviation of the means of triplicate samples. (B) Representative images of *P. aeruginosa* swarming on agar plates used to generate data in panel A.

### Computational Docking Reveals Sulfonyl HLs Are Accommodated within the BHL-Binding Site in RhlR

As the final stage of this study, we were interested in exploring putative interactions sulfonyl HLs could have in the RhlR ligand-binding site, assuming these compounds engage this site to elicit their antagonistic activity, to begin to unravel their mechanisms of action. We performed computational docking studies using the reported structure of RhlR bound to the synthetic agonist mBTL (PDB ID: 7R3J),^23^ an AHL analog with an aryl tail approximating compounds in this study. The BSHLs **39**, **43**, **55**, and **59** and ASHL **75** were each docked into the ligand-binding site of this structure (see Methods) and, with the exception of **55**, were found able to form headgroup-binding contacts also observed in the crystal structures of RhlR bound to mBTL (and to BHL; PDB ID: 8B4A),^23^ namely, hydrogen bonds between the HL ring and W68 and between the sulfonamide (in place of the amide) and Y64, D81, and S135 (Figures 6A and S2). Although the S135 contact varies, hydrogen bonds to residues analogous to RhlR W68, Y64, and S135 are highly conserved in crystal structures of other LuxR-type receptors bound to AHLs, including LasR,^60^ TraR,^61^ QscR,^62^ and SdiA.^63^ Our docking experiments also revealed that the sulfonamides of the 3-Br (**39**), 3,4-di-Cl (**43**), and 3,4-di-F (**59**) BSHLs are positioned for a potential hydrogen bonding contact with Y72 (purple line in Figure 6A). Interestingly, none of the predicted poses for BSHL **55** (again, the d *-*enantiomer of **43**) positioned the HL ring to interact with W68; rather, the lowest-energy pose shows the HL in **55** twisted, potentially allowing an interaction between the HL and S135 (Figure 6B). A crystal structure of LasR bound to a different synthetic aryl AHL agonist (BB0126) shows this ligand in a twisted conformation similar to the docked pose of **55** in RhlR, indicating that such a pose could be biologically relevant.^26^ The sulfonamides in **43** and **55** adopt similar conformations, however, which may help explain why BSHL **55** maintains comparable inhibitory activity as **43** in RhlR despite its inverted (non-native) stereochemistry (Figure 6B).

**Figure 6:**
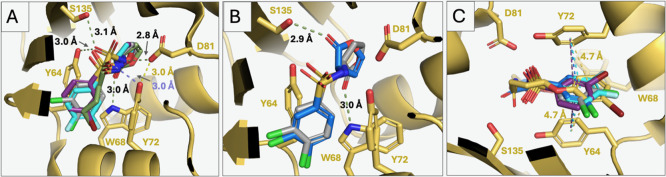
Views of selected sulfonyl HLs computationally docked into the ligand-binding site of the RhlR crystal structure with mBTL (PDB ID: 7R3J, in gold). See Figure S2 for images of individual docked ligands. (A) Comparison of docked poses of 3-Br BSHL (**39**, purple), 3,4-di-Cl BSHL (**43**, gray), 3,4-di-F BSHL (**59**, cyan), and C_5_ ASHL (**75**, green), highlighting HL contacts. Hydrogen bonds representing conserved HL interactions are shown in green, the hydrogen bond between Y72 and D81 observed in the mBTL-bound crystal structure is shown in gold, and the potential additional hydrogen bond from the BSHL sulfonyls to Y72 is shown in purple. (B) Lowest-energy pose of 3,4-di-Cl BSHL (**55**, the d-enantiomer of **43**, blue) compared to that of BSHL **43** (gray). Alternative hydrogen bonding pattern of the HL headgroup shown in green. (C) Docked poses of BSHLs **39** (purple), **43** (gray), **55** (blue), and **59** (cyan) showing the overlap of the BSHL phenyl rings with that of mBTL (gold). The distance from the center of the mBTL phenyl ring to the center of the Y64 and Y72 rings is shown in gold. Corresponding distances from Y64 and Y72 to **39**, **43**, **55**, and **59** are 4.4–5.0 Å and are shown in purple (**39**), gray (**43**), blue (**55**), and cyan (**59**).

Turning to the aryl tails, the phenyl rings of BSHLs **39**, **43**, **55**, and **59** were predicted to occupy a similar position to that of mBTL (Figure 6C) and may form π–π interactions with the side chains of both Y64 and Y72, as has been suggested for mBTL.^23^ The distances from the phenyl rings of **39**, **43**, **55**, and **59** to Y64 and Y72 are 4.4–5.0 Å, measured between the ring centers, comparable to the corresponding distances for mBTL (4.7 Å) and reasonable distances for π–π interactions in proteins.^64,65^ Phenyl ring substituents can affect these interactions (e.g., electron-withdrawing groups), which may contribute to the effects of ring substituents on the potency of BSHLs in the current study. Overall, these docking results suggest that sulfonyl HLs can adopt a binding pose in the RhlR ligand-binding site that maintains binding contacts that are shown to be important for AHL interactions with RhlR and with other LuxR-type receptors. How such contacts engender RhlR antagonism (as opposed to RhlR agonism by mBTL) remains unknown and will require additional study.

## Summary and Conclusions

The goal of this study was to explore the activity of AHL analogs containing sulfonamides as amide bioisosteres in the LuxR-type QS receptor, RhlR, from the common bacterial pathogen *P. aeruginosa*. Methods to block the activity of this receptor are attractive due to its prominent role in controlling the production of virulence factors in *P. aeruginosa*. Sulfonyl HLs have previously been shown to modulate the activity of other LuxR-type receptors yet have not been explored extensively as chemical probes to study QS and, to our knowledge, have not been evaluated systematically as a scaffold for activity in RhlR. We designed and synthesized a range of aryl and alkyl HLs and evaluated their activity in an *E. coli* RhlR reporter assay for antagonistic activity. Many active compounds were identified, the majority of which could block the activity of RhlR by >90% at mid- to high-micromolar concentrations. The most potent antagonists were 3,4-dihalosubstituted BSHLs and ASHLs with C_4_–C_6_ tails, with IC_50_ values only ∼2–3 times higher than the EC_50_ value for RhlR’s native ligand, BHL. Compound potency could be readily tuned in the BnSHL and BSHL classes via the repositioning of electron-withdrawing substituents on the aryl ring. These seemingly subtle structural changes allowed for up to ∼40-fold potency gains, as exemplified by BnSHL **16** vs **33** (4-NO_2_ → 3-Cl), BSHL **24** vs **43** (2,4-Cl → 3,4-Cl), and BSHL **45** vs **86** (2,3,4-Cl → 3,4,5-F) (key SAR summarized in Figure 7). The preference for the short BSHL and ASHL scaffolds is congruent with the short native RhlR ligand, BHL. Computational docking experiments revealed that these derivatives were readily accommodated in the BHL-binding site and can engage in interactions observed in structures of RhlR and LasR with other AHL-type ligands.

**Figure 7:**
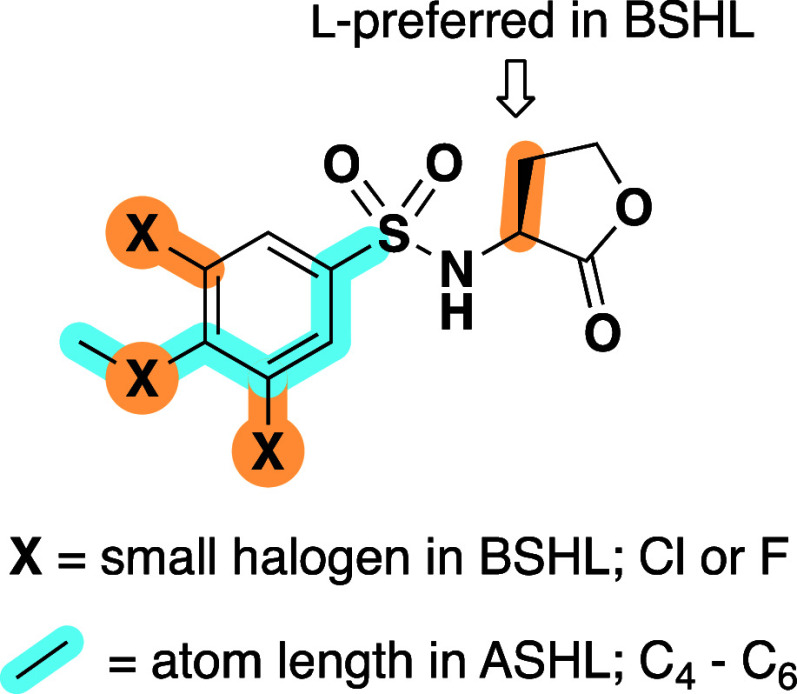
General structure of a sulfonyl HL that summarizes SARs for RhlR antagonism.

A small number of partial agonists were identified in each substructure class, indicating that sulfonyl HLs are capable of both RhlR agonism and antagonism, at least in the *E. coli* reporter. Interestingly, inversion of native HL stereochemistry only reduced antagonism potency 4-fold, without impacting efficacy (99%), for 3,4-Cl BSHL (**55** vs **43**). We are unaware of this effect in any other non-native AHL. Examining other BSHLs with D-HL headgroups is warranted to understand whether this activity trend extends beyond this stereoisomer pair. Our docking experiments predicted that the inversion of stereochemistry prevents **55** from making key HL-binding contacts but does not necessarily alter the positioning of the tail phenyl ring relative to its enantiomer, **43**. The lead RhlR antagonists remained efficacious in a *P. aeruginosa* RhlR reporter (albeit losing potency relative to the *E. coli* reporter) and were capable of inhibiting swarming motility in wild-type *P. aeruginosa*, a virulence phenotype linked to the Rhl system. Overall, this study provides new chemical tools to interrogate the role of RhlR in *P. aeruginosa* QS networks and virulence, a function of increasing research interest, and highlights the utility of the sulfonyl HL as a scaffold for QS modulator design. Ongoing work is focused on systematically investigating the activity of these compounds in other LuxR-type receptors to define SARs and any receptor selectivity, further delineating their molecular mechanisms of RhlR antagonism, and exploring their ability to attenuate *P. aeruginosa* infection.

## Methods

### General

All new compounds were fully characterized for purity and identity (see Supporting Information). Details of strains and plasmids are given in Table S1. Compounds **26**, **27**, **33**, **35**, **39**, **43**, and **64** had a modest effect on bacterial growth at >2 mM concentrations (as determined by monitoring OD_600_ over time); these concentrations were avoided in the studies herein. The remaining compounds had no effect on growth over the concentration range tested. All compounds were tested in bacteriological assays in triplicate and in at least three separate trials using unique cultures. IC_50_ and EC_50_ values in reporter assays were calculated using GraphPad Prism software (version 9.0) with a variable slope sigmoidal curve fit.

### Synthesis of Sulfonyl HLs

All compounds were synthesized via coupling L-HL to appropriate sulfonyl chlorides using established coupling reactions^66^ and purified to ≥95% purity via silica gel chromatography. See Supporting Information for a representative synthesis of compound **57**.

### Reporter Assay Methods

RhlR activity in *E. coli* strain JLD271 (Δ*sdiA*) was measured via a β-galactosidase reporter (*rhI-lacZ*) using the Promega Beta-Glo Assay System as previously described.^54^ RhlR activity in *P. aeruginosa* PA14 (Δ*rhlI*Δ*lasI*) was measured via a GFP reporter (*rhlA-gfp*). RhlR has been shown to activate *rhlI* and *rhlA* to similar levels in *P. aeruginosa*, allowing for comparisons between the two reporter systems.^67^ See Supporting Information for both reporter protocols.

### Swarming Motility Assay Method

The motility assay was based in part on a previously reported procedure.^68^ *P. aeruginosa* PAO1 was grown overnight with 500 or 250 μM compound, or vehicle (DMSO), at 37 °C and normalized to OD_600_ = 0.4. Cells were washed with sterile water twice and resuspended with 500 (**39**, **43**, and **59**) or 250 (**75**) μM fresh compound or vehicle (DMSO), in M8 minimal medium. Cells (5 μL) were spotted in the center of agar plates (100 mm diameter) generated from M8 minimal medium + 1 mM MgSO_4_ + 0.2% glucose + 0.5% Casamino acids + 0.5% agar. Plates were covered and the cells were grown for 24–48 h at 37 °C prior to imaging (using an iPhone 14 camera). Photographs were analyzed using ImageJ to calculate the area of the swarm and normalized to the area of (*P. aeruginosa* PAO1 + vehicle) – (*P. aeruginosa* PDO111 (Δ*rhlR*) + vehicle).

### Molecular Docking Method

Compounds were docked to the ligand-binding site of chain C of the reported crystal structure of RhlR bound to PqsE and the synthetic agonist mBTL (PDB ID: 7R3J)^23^ using the SwissDock server running AutoDock Vina.^69,70^ The protein was held static, and a box size of 20 Å^3^ centered around the ligand-binding site was sampled with an exhaustivity of 10. Figures were created in PyMol (Schrödinger, LLC., version 3.1.1 (2020)). See Supporting Information for an additional discussion of docking analysis.

## Supplementary Material



## Notes

Full details of experimental methods, compound synthesis and characterization, structures of additional compounds, dose–response activity curves, and computational docking analyses (PDF)
