# Correction to “Sulfonyl Homoserine Lactones are Tunable Probes to Inhibit the Quorum Sensing Receptor RhlR and Reduce Swarming Motility in *Pseudomonas aeruginosa*”

**DOI:** 10.1021/acsinfecdis.5c00927

**Published:** 2025-11-05

**Authors:** Guadalupe Aguirre-Figueroa, Diana A. Morales Mijares, Isabel D. Cannell, Irene M. Stoutland, Helen E. Blackwell

The structure of compound **71** is incorrect in Figure 4 and Table 1. The correct structure contains a 4-chloro group instead of a 4-fluoro group and is shown in the corrected Figure 4 below. Accordingly, the aryl substituents for compound **71** should be listed as “3-NO_2_, 4-Cl” in Table 1. Text on page 2840 of the article that describes compound **71** should state, “Replacing 3-CF_3_ with a nitro group (in 3-NO_2_, 4-Cl BSHL **71**) reduced both potency and efficacy markedly.” This error does not alter the conclusions of the study.

**Figure 4:**
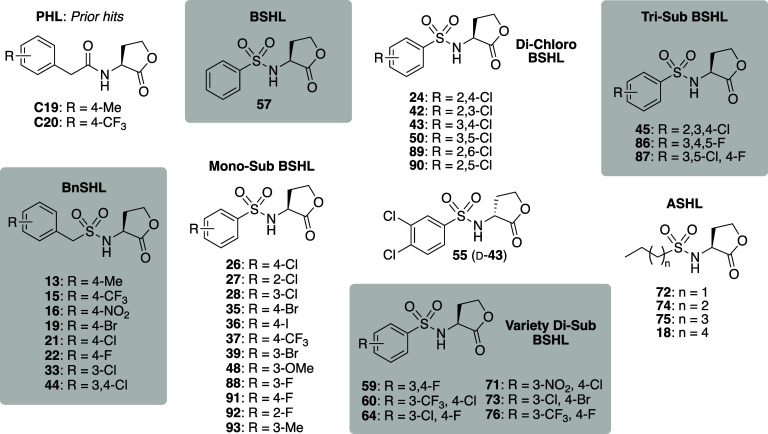
Chemical structures of the sulfonyl HLs examined in this study and selected known RhlR antagonist controls (**C19** and **C20**).^33^ Compounds listed numerically by substructure class. ASHLs **74**, **75**, and **18** were part of prior studies by our lab^47,48^ but examined in RhlR here.

There are errors in two structures in the Supporting Information. The structures of compounds **38** and **71** are incorrect in Figure S1. Please see the Supporting Information file also corrected here. Neither of these errors alter the conclusions of the study.

## Supplementary Material



## Notes

Corrected Figure S1 (PDF)

