# Supplementary material for: Sulfonyl Homoserine Lactones Are Tunable Probes to Inhibit the Quorum Sensing Receptor RhlR and Reduce Swarming Motility in Pseudomonas aeruginosa
Source: ACS Infect Dis. 2025 Sep 17;11(10):2836–46. doi: 10.1021/acsinfecdis.5c00542 (PMC12456999; doi:10.1021/acsinfecdis.5c00542)
Supplement: Supplementary file 1 [file id5c00542_si_001.pdf]

SUPPORTING INFORMATION

Sulfonyl Homoserine Lactones are Tunable Probes to Inhibit the Quorum Sensing Receptor RhlR and Reduce Swarming Motility in *Pseudomonas aeruginosa*

Guadalupe Aguirre-Figueroa, Diana A. Morales Mijares, Isabel D. Cannell, Irene M. Stoutland, and Helen E. Blackwell*

Department of Chemistry, University of Wisconsin–Madison, 1101 University Ave., Madison, WI 53706, USA

**Corresponding author (H.E.B.):* blackwell@chem.wisc.edu

CONTENTS

- General experimental and instrumentation information
- Synthesis of a representative BSHL
- **Figure S1.** Structures of prior RhlR modulators and all the compounds synthesized in this study
- **Table S1.** Bacterial strains and plasmids used in this study
- Construction of *P. aeruginosa* PA14 Δ*rhlI*Δ*lasI* mutant
- *E. coli* and *P. aeruginosa* RhlR reporter assay protocols
- **Scheme S1.** Schematic of *E. coli* reporter plasmids and assay
- Dose-response reporter assay data for compounds in the *E. coli* RhlR reporter
- Dose-response reporter assay data for compounds in the *P. aeruginosa* RhlR reporter
- Additional commentary on computational docking experiments
- **Figure S2.** Additional views of BHL and non-native AHLs docked into RhlR
- Compound characterization data
- References
- NMR spectral data for compounds

General experimental and instrumentation information.

All standard chemicals, reagents, and solvents were obtained from commercial sources (Acros Organics, Ambeed, Apollo Scientific, AstaTech, Matrix Scientific, Oakwood Chemical, Santa Cruz Biotechnology, Sigma-Aldrich, and TCI America) and used without additional purification. BHL and OdDHL were purchased from Cayman Chemical. Control compounds **C19** and **C20** were sourced from our in-house compound library (originally reported in 2007).[1, 2] Antibiotics (ampicillin, chloramphenicol, and gentamicin), arabinose, and Luria−Bertani (LB) growth medium were purchased from DOT Scientific Inc. Water (18 MΩ) was purified using a Sartorious Arium Pro System. The Beta-Glo® Reagent (6-*O*-β-galactopyranosyl-luciferin), the substrate for the Beta-Glo® Assay System, was purchased from Promega Corporation. Analytical thin-layer chromatography (TLC) was performed on 250 μm glass backed silica plates with a F-254 fluorescent indicator from Silicycle. TLC plate visualization was performed using UV light. Compound stock solutions were prepared in DMSO and stored at -20 °C in sealed glass vials until use. DMSO did not exceed 1% in the biological testing of any compound in this study.

Bacteria were cultured in LB medium at 37 °C with shaking at 200 rpm with appropriate antibiotics unless otherwise noted. Cell growth was determined by measuring the absorbance of the culture at 600 nm (OD_{600}). Absorbance, luminescence, and fluorescence measurements were obtained using a BioTek Synergy 2 plate reader running Gen5 software (version 3.12) analysis software.

NMR spectra were recorded in deuterated solvents at 500 MHz on a Bruker Avance spectrometer equipped with a DCH cryoprobe. Chemical shifts are reported in parts per million using residual tetramethylsilane (TMS) as a reference. Couplings are reported in hertz (Hz). Electrospray ionization mass spectrometry (ESI-MS) data were collected on a Waters Acquity™ LCMS.

Synthesis of a representative BSHL.

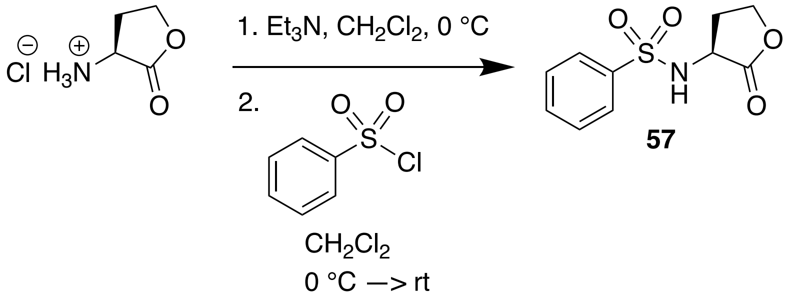

The synthesis of BSHL **57** is provided as a representative example of the synthetic methods used in this study. (*S*)-(-)-α-Amino-*γ*-buyrolactone hydrobromide (200 mg, 1.10 mmol, 1.0 equiv.) and triethylamine (0.457 mL, 3.30 mmol, 3.0 equiv.) were dissolved in anhydrous CH_2Cl_2 (10 mL) in a dry 100-mL round-bottom glass flask. The reaction was allowed to stir at 0 °C for 5 min. Benzenesulfonyl chloride (0.210 mL, 1.65 mmol, 1.5 equiv.) was dissolved in anhydrous CH_2Cl_2 (3 mL) in a glass vial and added to the round-bottom flask via syringe in three 1-mL portions, every 5 min, at 0 °C. The reaction was allowed warm to room temperature and stirred overnight (~16 h). The reaction mixture then was washed with 1 M HCl (3 x 30 mL), saturated sodium bicarbonate (3 x 30 mL), and brine (1 x 30 mL). The organic layer was isolated, dried over magnesium sulfate, and concentrated under reduced pressure. The crude material was purified by flash silica gel column chromatography (100% CH_2Cl_2 followed by 1% MeOH in CH_2Cl_2). Compound **57** was isolated as a yellow-orange solid (120 mg, 45% isolated yield).

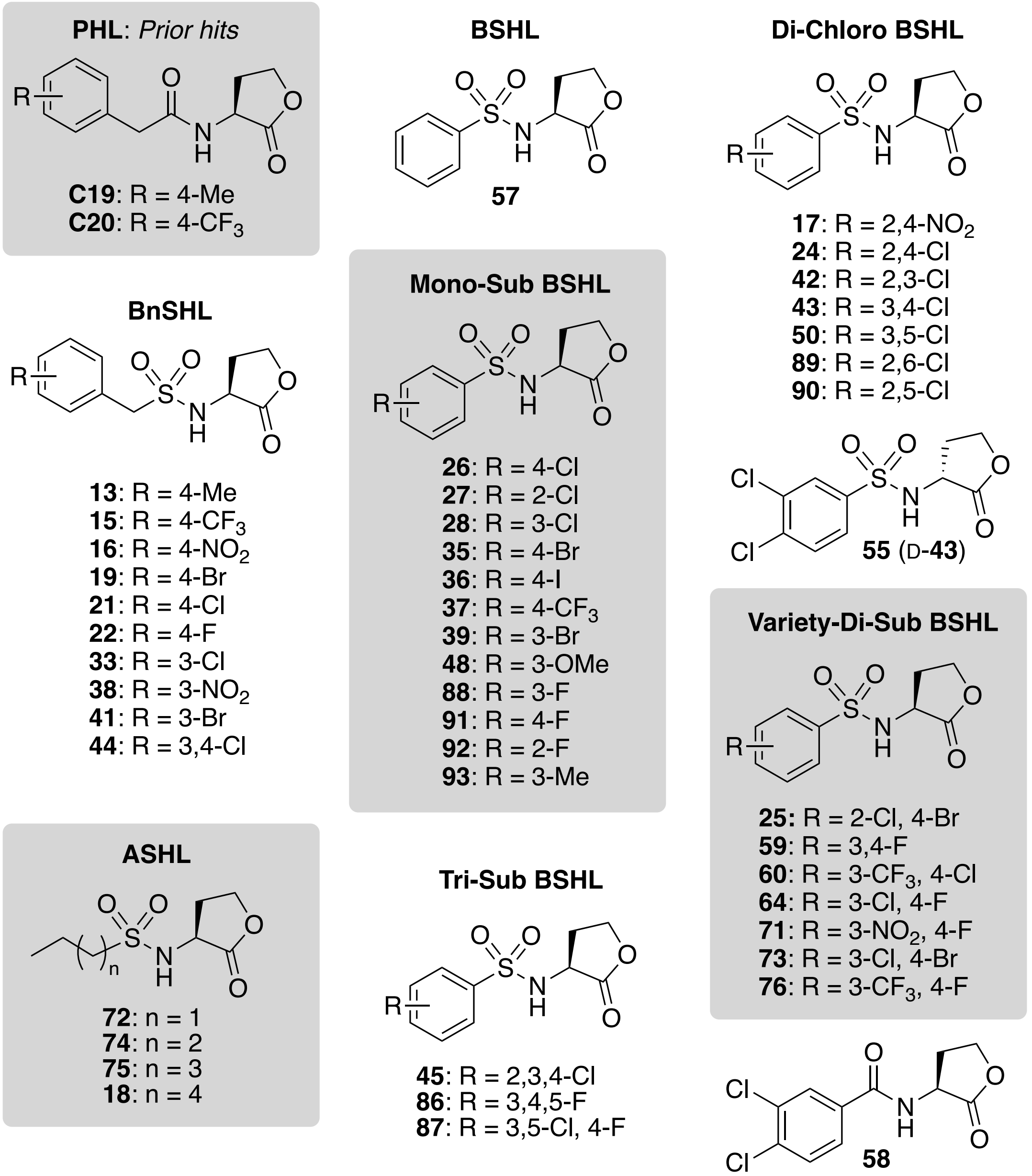

Figure S1. Structures of prior RhlR modulators and all the compounds synthesized in this study. Bioactivity data for compounds **17**, **25**, **38**, **41**, and **58** in RhlR not included in this report.

Table S1. Bacterial strains and plasmids used in this study.[a]

Strain or Plasmid	Description	Reference or source
E. coli		
JLD271	K-12 ΔlacX74 sdiA271::Cam; ClR	[3]
pJN105-R2	arabinose-inducible RhlR expression plasmid; GentR	[4]
pSC11-rhlI	*rhlI'-lacZ* transcriptional fusion RhlR reporter plasmid, AmpR	[4]
P. aeruginosa		
PAO1	Wild-type strain	In-house
PDO111	*rhlR::Tn501-11* derivative of PAO1 strain	[5]
PA14 Δ*rhlI*Δ*lasI*	Wild-type PA14 strain with Δ*rhlI* and Δ*lasI* mutations	[6]; McKee and Chandler *(see below)*
pPROBE-GT *rhlA*-GFP	*rhlA-gfp* transcriptional fusion reporter plasmid; GentR	[7]

[a]ClR = Chloramphenicol resistance. AmpR = ampicillin resistance. GentR = gentamicin resistance.

Construction of *P. aeruginosa* PA14 Δ*rhlI*Δ*lasI* mutant.

The PA14 Δ*rhlI*Δ*lasI* strain was a generous gift from Prof. Josephine Chandler's lab (University of Kansas). The strain was generated from PA14 by first introducing the Δ*rhlI* mutation by allelic exchange using the pEXG2 Δ*rhlI* suicide plasmid,[8] then subsequently introducing the Δ*lasI* mutation using the pEXG2 Δ*lasI* suicide plasmid.[9] Delivery of the suicide plasmid was by mating following by gentamicin selection (200 μg/mL) and sucrose counterselection. Both mutations were confirmed by PCR amplification and Sanger sequencing.

***E. coli* and *P. aeruginosa* RhlR reporter assay protocols.**

***E. coli* RhlR reporter assay.** RhlR activity in *E. coli* strain JLD271 (Δ*sdiA*) was measured via a β-galactosidase reporter using the Promega Beta-Glo® Assay System as previously described.[10] A schematic of the reporter plasmids and assay protocol is shown in **Scheme S1**. Briefly, a single colony of *E. coli* JLD271 harboring the RhlR expression plasmid pJN105-R and the *rhlI-lacZ* transcriptional fusion reporter pSC11-R (**Table S1**) was grown in LB medium with 100 µg/mL ampicillin and 10 µg/mL gentamicin overnight (~16 h). The culture was diluted 1:10 in fresh LB medium and grown to an OD_{600} = 0.25 (0.23–0.28) (~0.5 h). Arabinose was added to a final concentration of 4 mg/mL to induce the expression of pJN105-R. Aliquots (2 µL) of compound DMSO stock solution or serial dilution were added to the inner wells (i.e., non-periphery rows) of a 96-well microtiter plate. To six inner wells, DMSO alone (vehicle) was added as a negative control. For agonism assays, BHL was added to six inner wells as a positive control (to achieve 1 mM). For antagonism assays, BHL was added to six inner wells as a positive control and to all of the other sample wells except for the vehicle control wells (to achieve 10 µM). Subculture (198 µL) was then dispensed into each inner well. To the outer (periphery) wells, 200 µL of water were added to maintain humidity during incubation. Plates were then incubated for 4 h (shaking) at 37 °C.

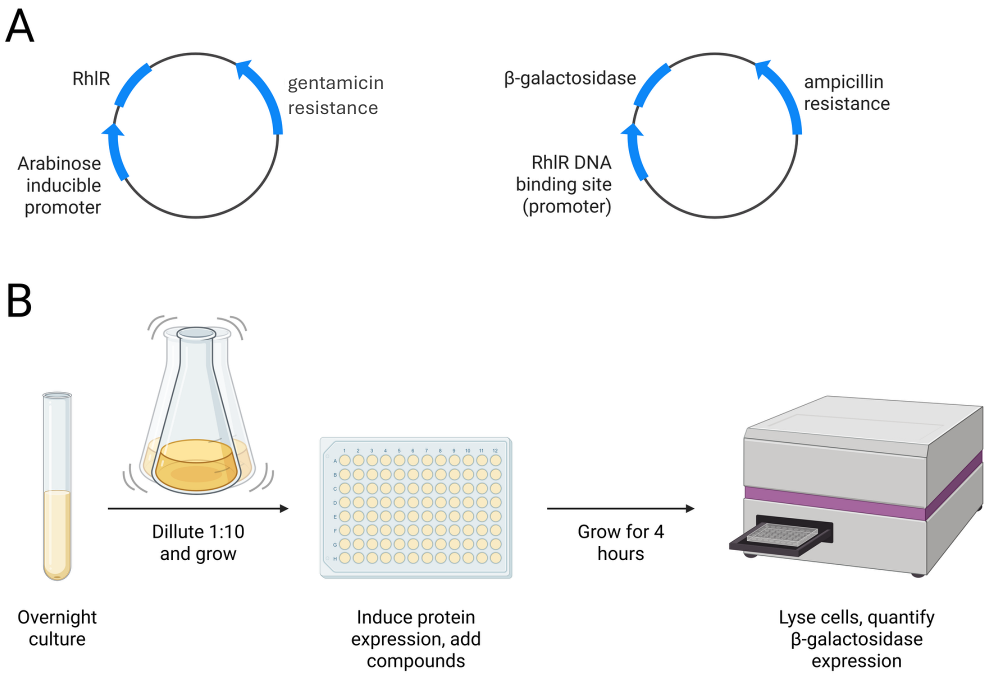

Scheme S1. Schematic of *E. coli* reporter plasmids and assay. (A) Representation of plasmids used in the heterologous reporter system. (B) General reporter assay workflow. BioRender used in graphics generation.

After the incubation period, the OD_{600} of each well was measured using a plate reader. Cell dilution plates were prepared by transferring 10 µL from the incubated culture plate wells to a fresh 96-well plate and adding 90 µL of 18 MΩ water to each well. Next, 10 µL aliquots of diluted Beta-Glo® reagent (1:2 with 18 MΩ water) were added to each well of a white 384-well microtiter plate, followed by 10 µL of culture from each well of the dilution plate. The 384-well plate was incubated (without shaking; i.e., static) for 30 min at 30 °C. After incubation, luminescence of each well was measured using a plate reader. To determine levels of RhlR activity, raw luminescence was divided by OD_{600} to correct for growth variation and then normalized to the negative (0%) and positive (100%) control wells.

P. aeruginosa **RhlR reporter assay.** RhlR activity in *P. aeruginosa* PA14 *ΔrhlIΔlasI* was measured via a GFP reporter. Briefly, a single colony of *P. aeruginosa* strain PA14 *ΔrhlIΔlasI* harboring the transcriptional fusion reporter pPROBE-GT *rhlA*-GFP (**Table S1**) was grown in LB medium with 40 µg/mL gentamicin overnight (~20 h). The culture was diluted 1:100 in fresh LB medium, without antibiotic, to an OD_{600} = 0.25 (0.22–0.30) (~1 h). Aliquots (2 µL) of compound DMSO stock solution or serial dilution were added to the inner wells (i.e., non-periphery rows) of a black, clear bottom 96-well plate. To 12 inner wells, DMSO alone (vehicle) was added to serve as controls (negative and positive). For the six negative control wells (no BHL), 198 µL of bacterial culture plus OdDHL (to achieve 10 µM) were added. For the six positive control wells (no test compound), 198 µL of bacterial culture plus OdDHL (10 µM) and BHL (to achieve 4 µM) were added. To all the remaining wells containing test compound, 198 µL of bacterial culture plus OdDHL (10 µM) and BHL (4 µM) were added. Water (200 µL) was added to the outer (periphery) wells to maintain humidity during incubation. Plates were incubated for 4 h at 37 °C. After incubation, the OD_{600} and GFP production (excitation at 500 nm, emission at 540 nm) were read for each well using a plate reader. To determine levels of RhlR activity, raw GFP production was divided by OD_{600} to correct for growth variation and then normalized to the negative (0%) and positive (100%) control wells.

<u>Dose-response reporter assay data for compounds in the *E. coli* RhlR reporter.</u>

Dose-response curves for RhlR antagonism by compounds against 10 μM BHL using the *E. coli* JLD271 RhlR reporter are provided below. Compound names are indicated on the x-axis of each plot. All assays performed in triplicate as at least three separate biological replicates. Error bars indicate SD of n = 3 trials. Curve fits generated using GraphPad Prism software (v9.0) using a variable slope sigmoidal curve fit. IC_{50} values calculated from these dose-response curves are listed in **Table 1** in the main text. ND = not determined.

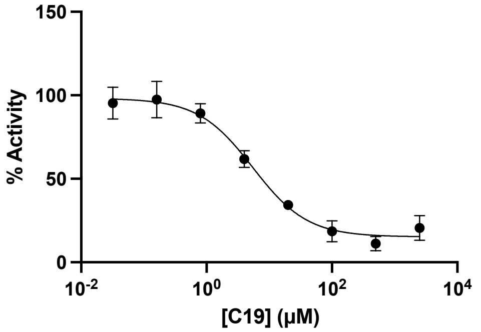

IC_{50} = 5.46 μM
95% CI = 4.05 μM to 7.14 μM

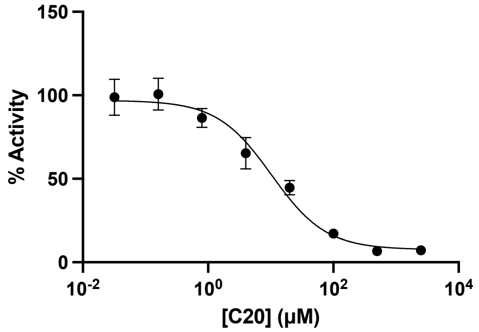

IC_{50} = 10.3 μM
95% CI = 7.30 μM to 14.5 μM

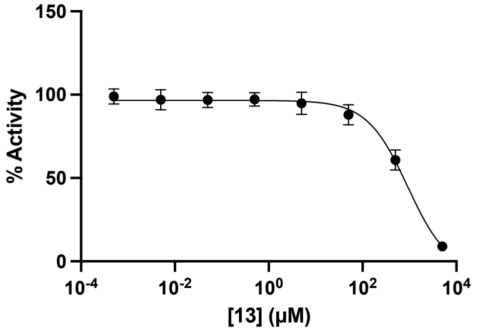

IC_{50} = 918 μM
95% CI = 740 μM to 1150 μM

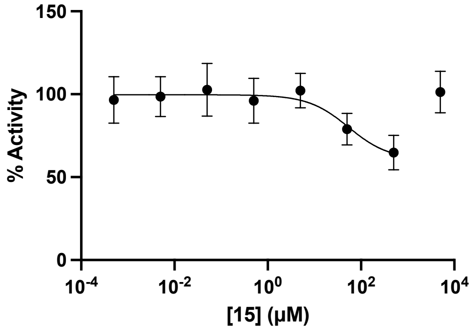

IC_{50} = ND (partial agonist; non-monotonic curve)[11]

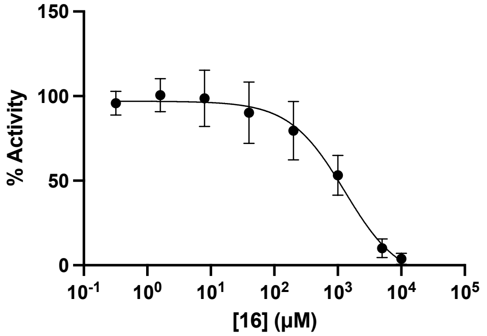

IC_{50} = 1290 μM
95% CI = 820 μM to 2066 μM

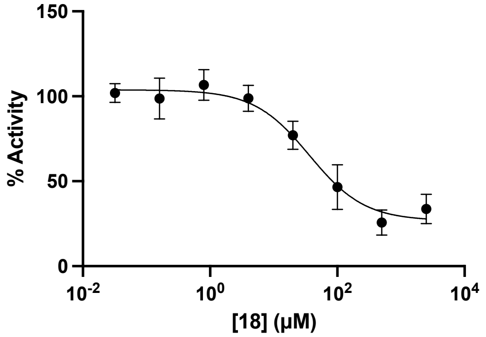

IC_{50} = 36.4 μM
95% CI = 25.7 μM to 51.3 μM

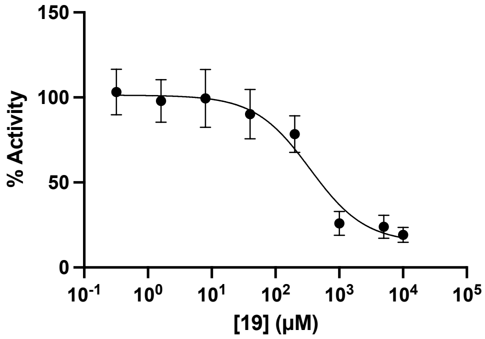

IC_{50} = 348 μM
95% CI = 237 μM to 510 μM

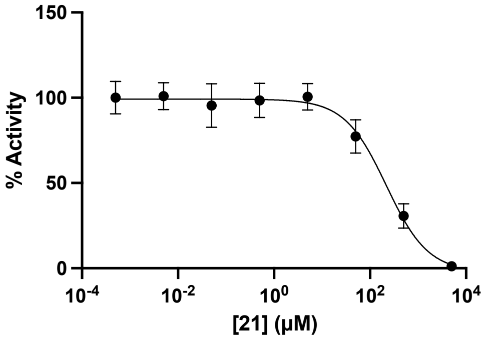

IC_{50} = 219 μM
95% CI = 156 μM to 303 μM

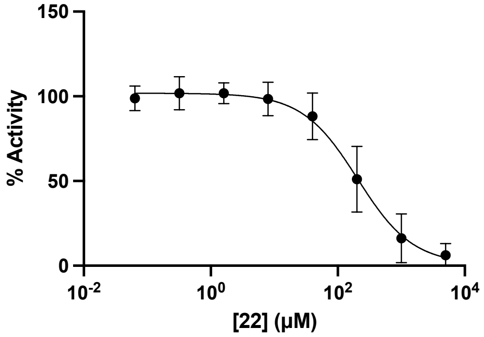

IC_{50} = 205 μM
95% CI = 146 μM to 287 μM

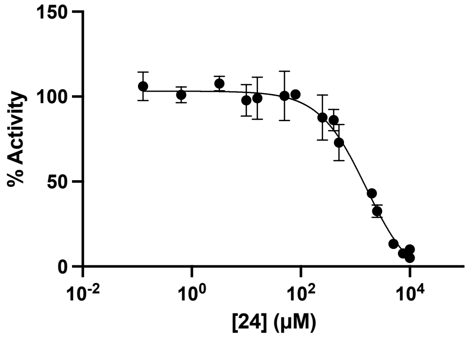

IC_{50} = 1590 μM
95% CI = 1210 μM to 2100 μM

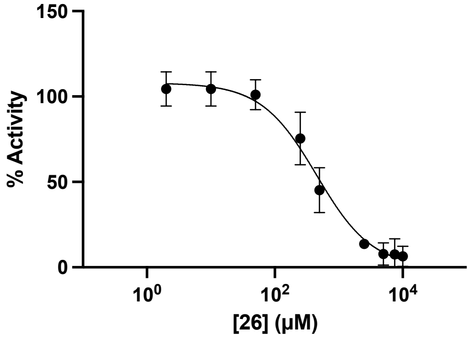

IC_{50} = 452 μM
95% CI = 358 μM to 575 μM

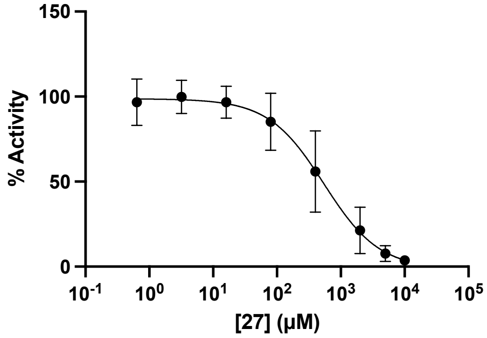

IC_{50} = 545 μM
95% CI = 359 μM to 835 μM

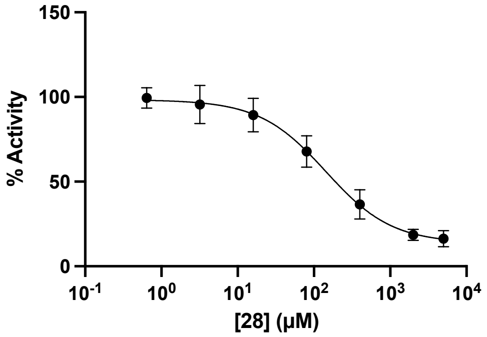

IC_{50} = 143 μM
95% CI = 107 μM to 192 μM

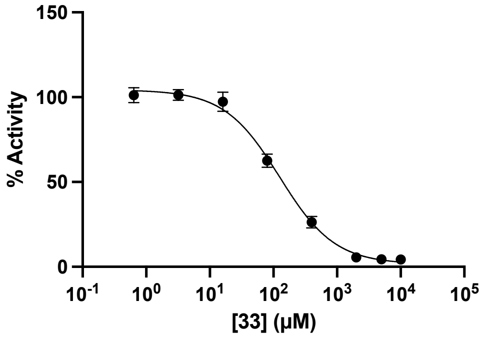

IC_{50} = 125 μM
95% CI = 104 μM to 151 μM

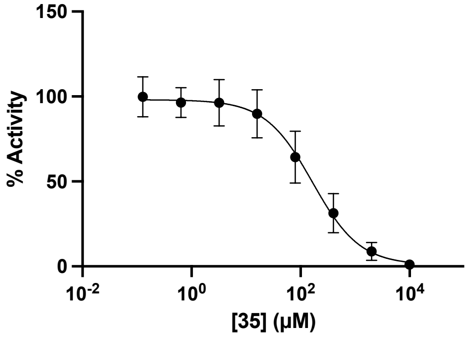

IC_{50} = 166 μM
95% CI = 118 μM to 232 μM

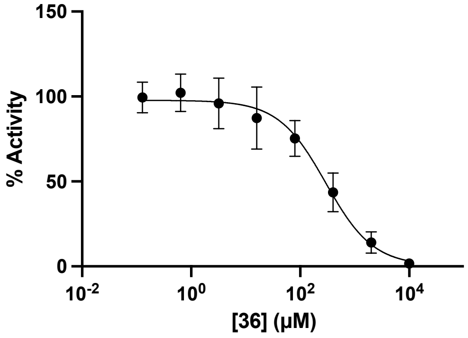

IC_{50} = 295 μM
95% CI = 202 μM to 428 μM

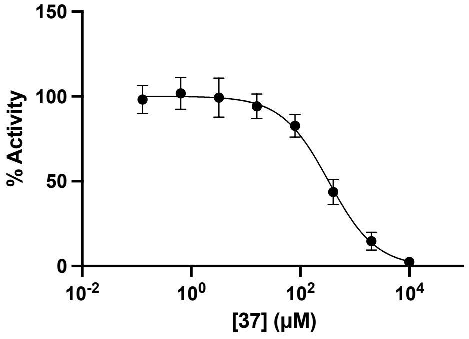

IC_{50} = 333 μM
95% CI = 265 μM to 417 μM

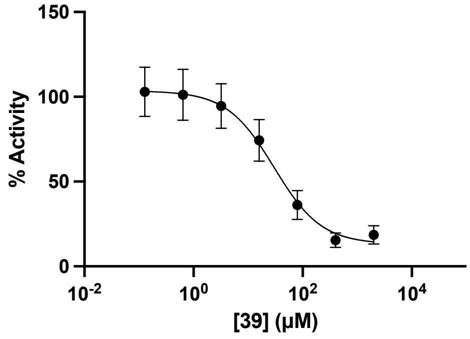

IC_{50} = 29.9 μM
95% CI = 20.9 μM to 42.7 μM

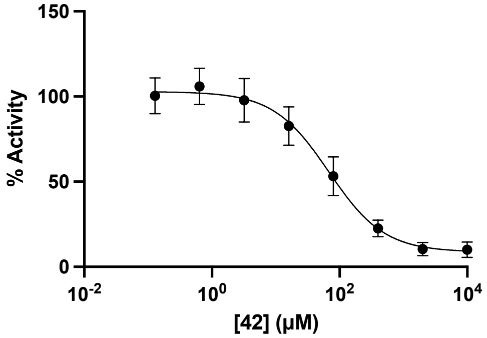

IC$_{50}$ = 67.8 μM
95% CI = 51.0 μM to 89.9 μM

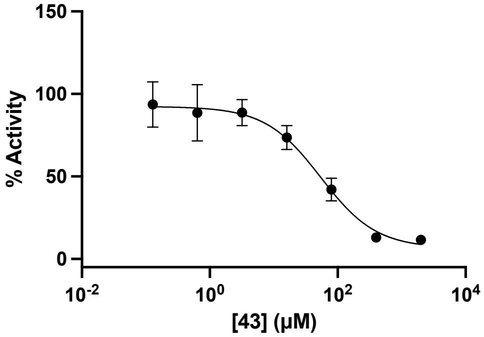

IC$_{50}$ = 54.3 μM
95% CI = 38.7 μM to 75.9 μM

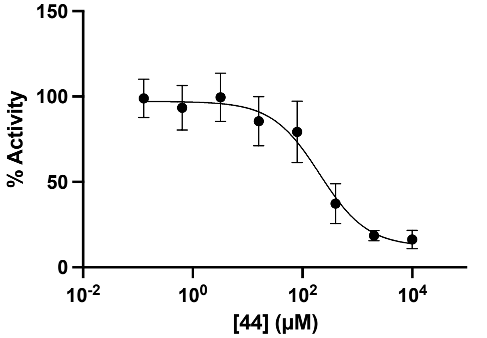

IC$_{50}$ = 205 μM
95% CI = 136 μM to 308 μM

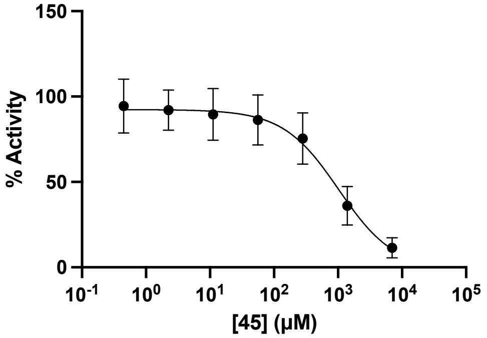

IC$_{50}$ = 1030 μM
95% CI = 617 μM to 1729 μM

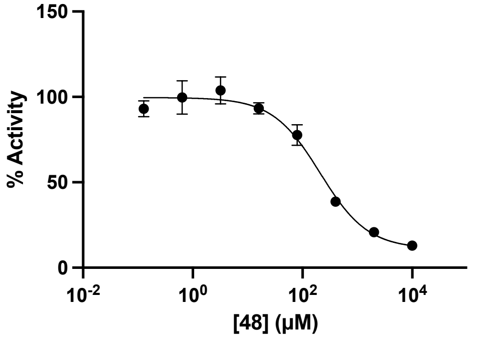

IC$_{50}$ = 210 μM
95% CI = 149 μM to 296 μM

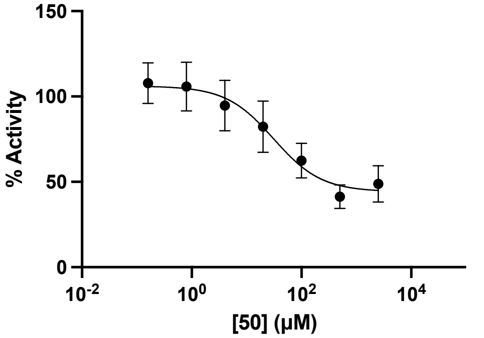

IC$_{50}$ = ND (partial agonist)

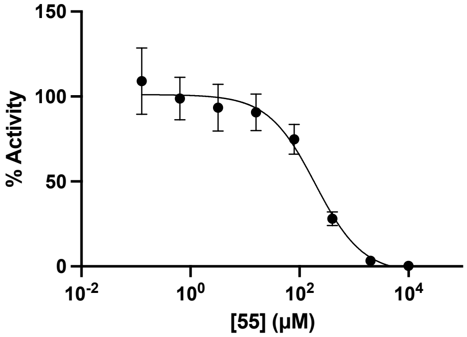

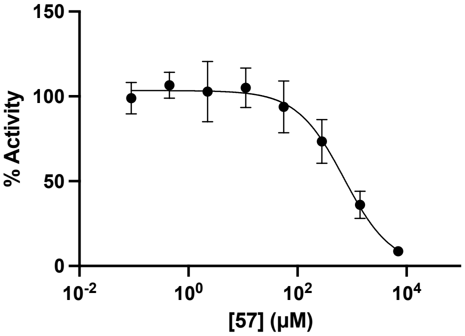

IC_{50} = 195 μM
95% CI = 143 μM to 264 μM

IC_{50} = 715 μM
95% CI = 481 μM to 1070 μM

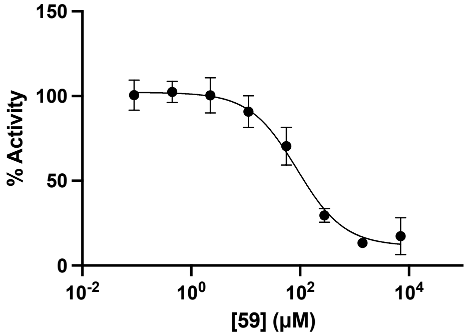

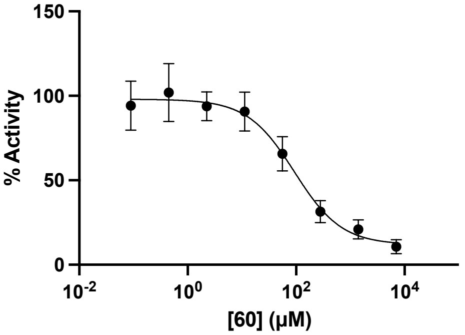

IC_{50} = 87.8 μM
95% CI = 67.5 μM to 114 μM

IC_{50} = 93.1 μM
95% CI = 65.5 μM to 133 μM

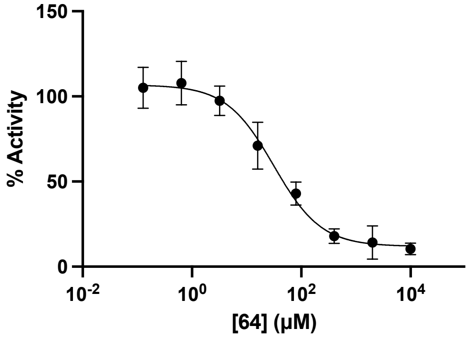

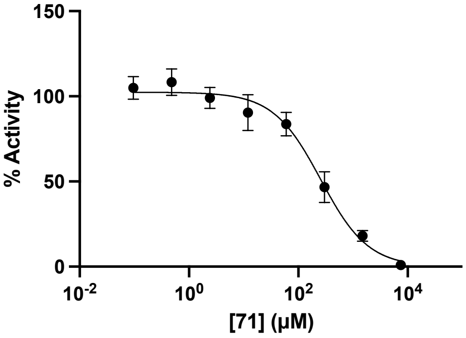

IC_{50} = 31.3 μM
95% CI = 21.6 μM to 45.6 μM

IC_{50} = 257 μM
95% CI = 200 μM to 328 μM

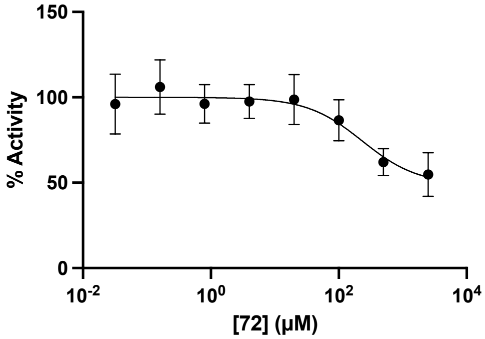

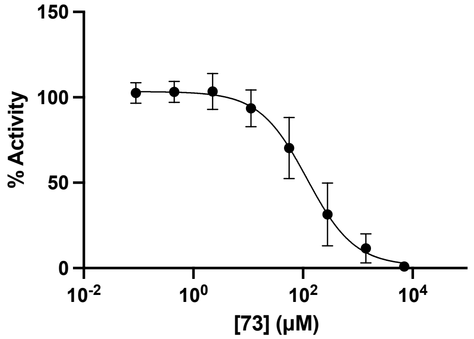

IC_{50} = ND

IC_{50} = 118 μM
95% CI = 85.7 μM to 163 μM

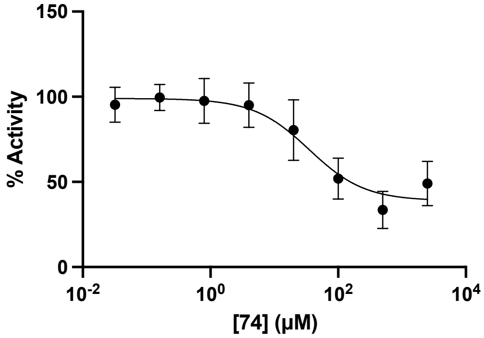

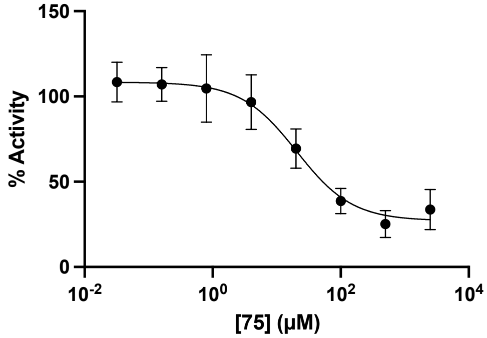

IC_{50} = 35.2 μM
95% CI = 20.0 μM to 61.4 μM

IC_{50} = 20.6 μM
95% CI = 13.5 μM to 31.1 μM

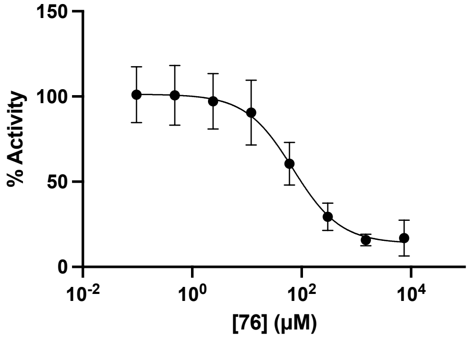

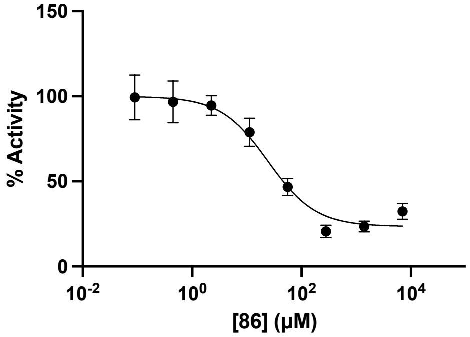

IC_{50} = 69.4 μM
95% CI = 45.2 μM to 106 μM

IC_{50} = 24.5 μM
95% CI = 16.9 μM to 35.3 μM

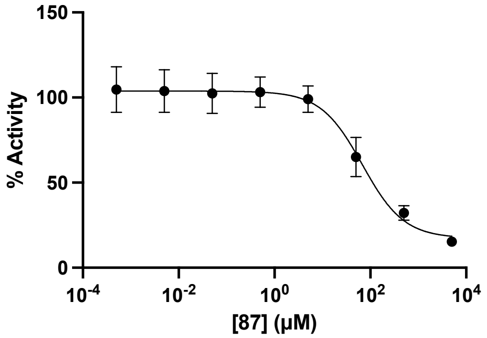

IC_{50} = 68.0 μM
95% CI = 47.3 μM to 99.1 μM

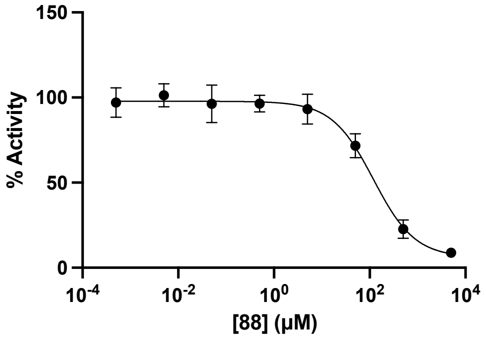

IC_{50} = 120 μM
95% CI = 92.1 μM to 156 μM

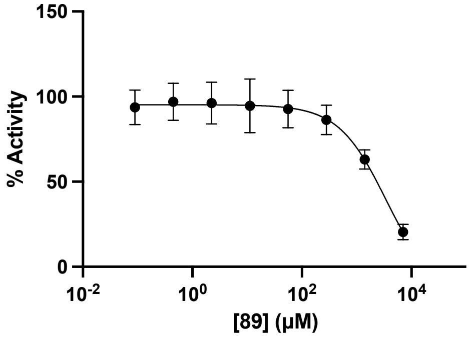

IC_{50} = >1000 μM
95% CI = ND

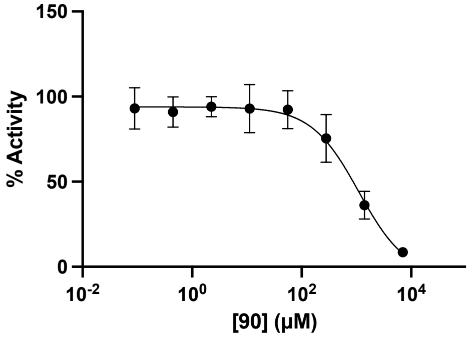

IC_{50} = 1100 μM
95% CI = 761 μM to 1620 μM

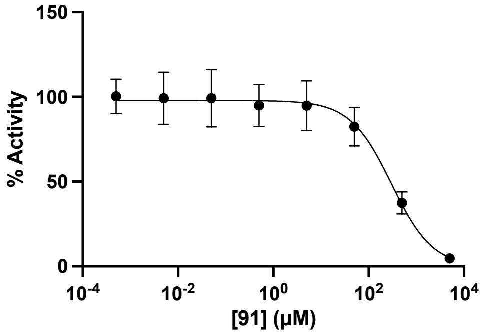

IC_{50} = 301 μM
95% CI = 190 μM to 463 μM

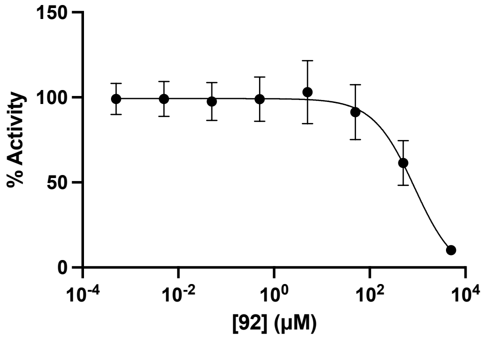

IC_{50} = 863 μM
95% CI = 534 μM to 1460 μM

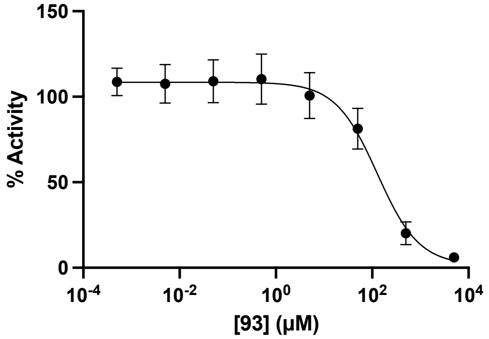

IC_{50} = 127 μM
95% CI = 92.3 μM to 177 μM

Dose-response curves for RhlR agonism by BHL and compounds **15** and **50** using the *E. coli* JLD271 RhlR reporter are provided below. Compound names are indicated on the x-axis of each plot. All assays performed in triplicate as at least three separate biological replicates. Error bars indicate SD of n = 3 trials. Curve fits generated using GraphPad Prism software (v9.0) using a variable slope sigmoidal curve fit.

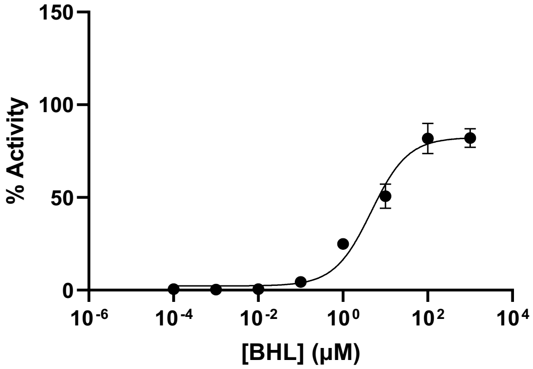

IC_{50} = 8.73 μM
95% CI = 7.64 μM to 10.11 μM

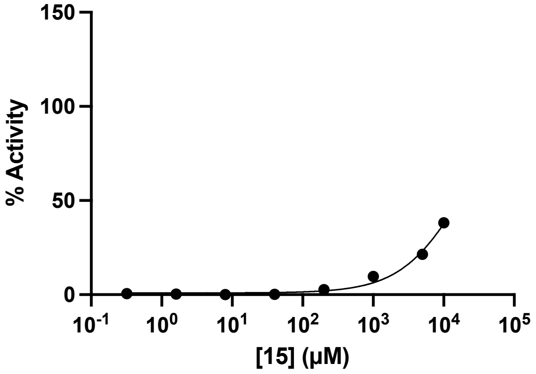

Max Activation = 38%

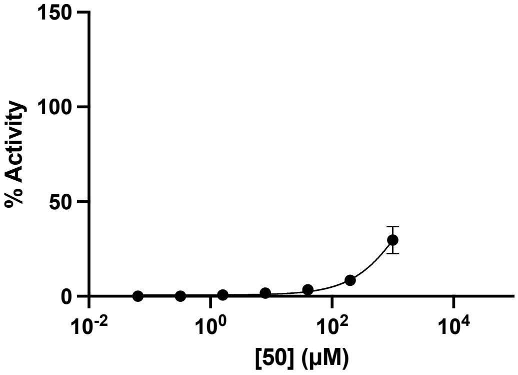

Max Activation = 30%

Dose-response reporter assay data for compounds in the *P. aeruginosa* RhlR reporter.

Dose-response curves for RhlR agonism by BHL and for RhlR antagonism by selected compounds against 4 µM BHL using the *P. aeruginosa* PA14 RhlR reporter are provided below. Compound names are indicated on the x-axis of each plot. All assays performed in triplicate as at least three separate biological replicates. Error bars indicate SD of n = 3 trials. Curve fits generated using GraphPad Prism software (v9.0) using a variable slope sigmoidal curve fit. IC_{50} values for antagonists were not calculated as the curves did not bottom out over the concentrations tested in the reporter system.

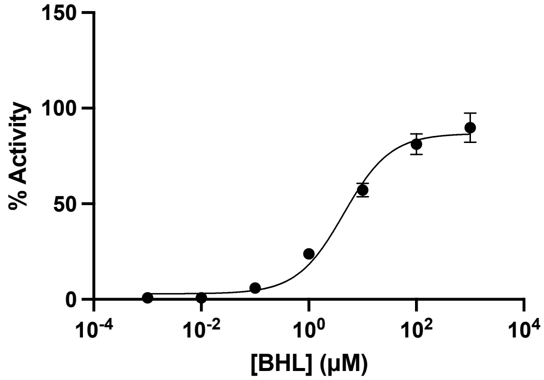

EC_{50} = 4.47 µM
95% CI = 3.38 µM to 5.85 µM

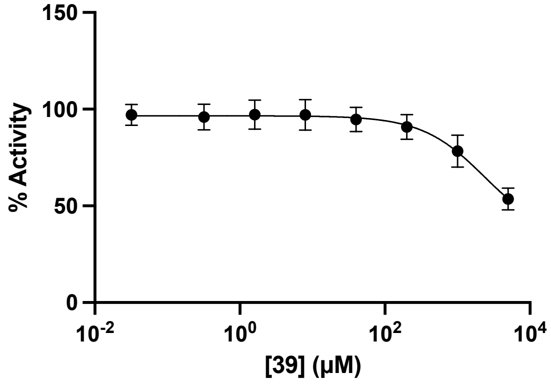

Max Inhibition = 47%

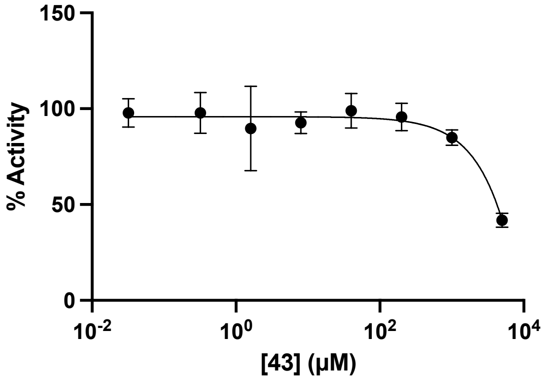

Max Inhibition = 59%

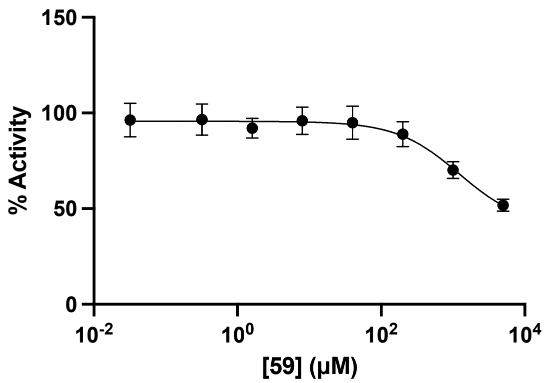

Max Inhibition = 49%

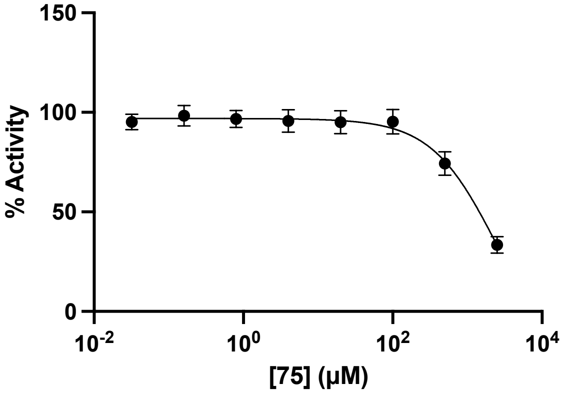

Max Inhibition = 67%

Additional commentary on computational docking experiments.

The crystal structure of RhlR bound to synthetic agonist mBTL (PDB ID: 7R3J)[12] was used for docking experiments with sulfonyl HLs to allow for more direct comparisons between the position of mBTL and docked sulfonyl HLs that contain an aromatic tail group. We are aware, of course, that mBTL and these sulfonyl HLs have opposing activities in RhlR, with the former a potent RhlR agonist and the latter RhlR antagonists. Thus, any shared or contrasting contacts between these compounds need to be considered carefully in assigning any mechanistic rationale to their agonistic or antagonist outcome. It is also important to note that the ligand-binding pockets of the BHL- and mBTL-bound crystal structures (PDB IDs: 8B4A and 7R3J, respectively) do not show major differences in backbone or side chain position.[12] Selected additional images of the docked compounds are shown in **Figure S2**.

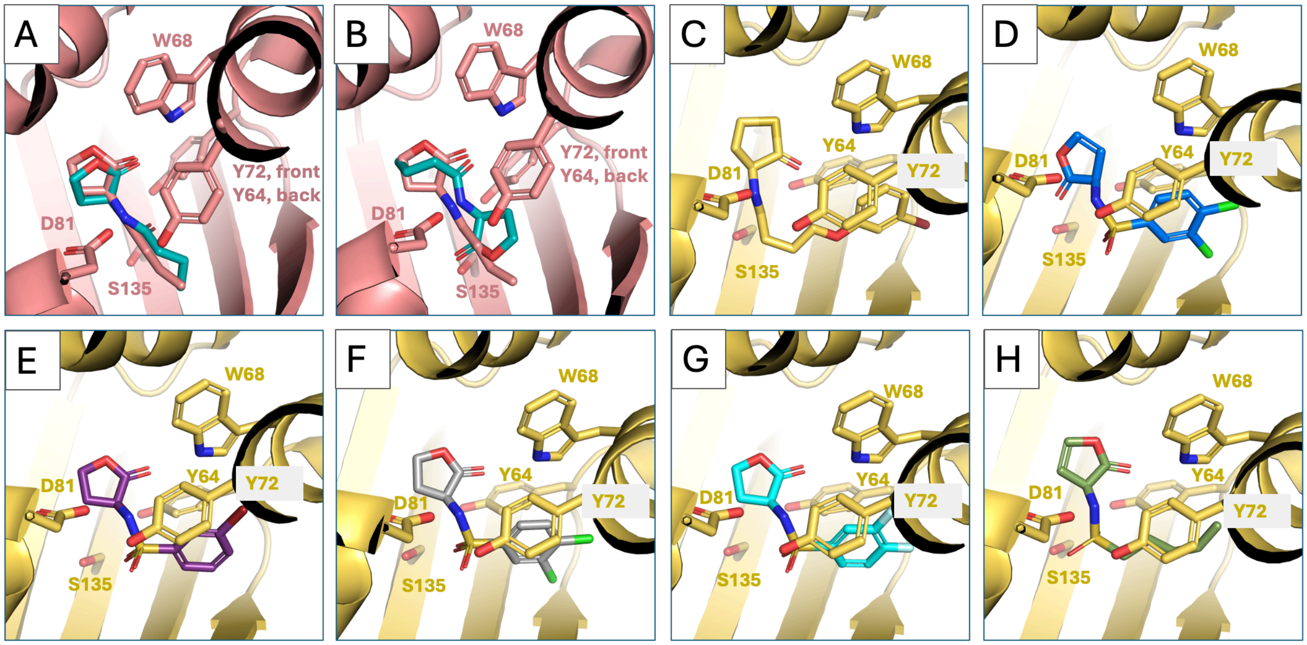

Figure S2. Additional views of BHL and non-native AHLs docked into RhlR. (A) BHL (dark teal) docked the crystal structure of RhlR (PDB ID: 8B4A, pink) bound to BHL (pink). (B) Flipped pose of BHL (dark teal) docked to the crystal structure of RhlR (8B4A, pink) bound to BHL (pink). (C) Crystal structure of mBTL (gold) bound to RhlR (PDB ID: 7R3J, gold). The structure of RhlR bound to mBTL is in gold in panels D→H. (D) BSHL **55** (blue) docked to RhlR. (E) BSHL **39** (purple) docked to RhlR. (F) BSHL **43** (gray) docked to RhlR. (G) BSHL **59** (teal) docked to RhlR. (H) BSHL **75** (green) docked to RhlR. For predicted H-bonds for representative compounds in panels C→H, see **Figure 6** in main text.

When we performed computational docking of BHL to the RhlR structure from the RhlR:BHL complex (PDB ID: 8B4A), some poses showed BHL flipped head-to-tail relative to BHL in the crystal structure (see **Figure S2A** vs. **S2B**), preventing HL-binding contacts that have been shown to be important for HL binding to RhlR and other LuxR-type receptors.[12] We also observed similar flipped poses for certain of the sulfonyl HLs docked into the RhlR structure from either the RhlR:mBTL complex (PDB ID: 7R3J) or the RhlR:BHL complex.[12] As there is no biochemical evidence for BHL adopting such flipped poses, it is unlikely that the flipped poses predicted for BHL or the sulfonyl HLs are biologically relevant. We therefore chose to focus on docked poses of sulfonyl HLs that made conserved HL-

binding contacts and were no more than 1.5 kcal/mol higher in energy than the lowest-energy docked pose. The poses selected for analysis are shown in the main text in **Figure 6** and above in **Figure S2**. Understanding how the predicted protein-ligand contacts contribute to the divergent outcomes between RhlR binding either BHL or mBTL (resulting in RhlR agonism) vs. the sulfonyl HLs (resulting in RhlR antagonism) will require additional mechanistic studies (e.g., *in vitro* binding and stability studies, etc.), which are ongoing.

Compound characterization data.

Note: Compounds **18**, **72**, **74**, and **75** (in racemic and/or enantiopure forms) and their associated characterization data have been previously reported by Castang *et al.*[13] and Geske *et al.*[2]

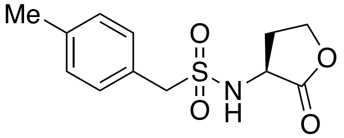

Compound 13: ^{1}H NMR (500 MHz, Methanol-d$_4$) δ 7.37 (d, *J* = 8.1 Hz, 2H), 7.19 (d, 2H), 4.42 (m, *J* = 1.9 Hz, 1H), 4.37 (m, 1H), 4.22 (m, *J* = 11.0, 9.1, 6.0 Hz, 1H), 2.51 (m, *J* = 12.5, 8.7, 6.0, 1.5 Hz, 1H), 2.34 (s, 3H), 2.15 (m, *J* = 12.4, 11.2, 8.8 Hz, 1H). ^{13}C NMR (126 MHz, Methanol-d$_4$) δ 175.87, 138.07, 130.73, 126.69, 128.73, 65.44, 59.44, 52.04, 52.08, 30.14, 19.80. ESI-EMM [M+NH$_4$]$^+$: calculated 287.1060; measured 287.1057.

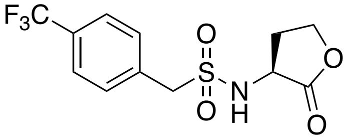

Compound 15: ^{1}H NMR (500 MHz, Methanol-d$_4$) δ 7.84 (s, 1H), 7.77 (d, *J* = 7.7 Hz, 1H), 7.66 (d, *J* = 7.8 Hz, 1H), 7.56 (t, *J* = 7.8 Hz, 1H), 4.80 (s, 1H), 4.59 (s, 3H), 4.48 (m, *J* = 11.6, 8.8 Hz, 1H), 4.37 (m, *J* = 9.0, 1.5 Hz, 1H), 4.25 (m, *J* = 11.0, 9.1, 6.0 Hz, 1H), 2.57 (m, *J* = 12.8, 8.8, 6.0, 1.5 Hz, 1H), 2.17 (m, 1H). ^{13}C NMR (126 MHz, Methanol-d$_4$) δ 173.32, 136.03, 132.69, 132.69, 130.28, 128.94, 126.13, 66.89, 60.39, 53.45, 31.39. ESI-EMM [M+NH$_4$]$^+$: calculated 341.0772; measured 341.0777.

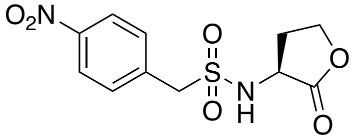

Compound 16: ^{1}H NMR (500 MHz, Methanol-d$_4$) δ 8.23 (d, *J* = 8.7 Hz, 2H), 7.76 (d, *J* = 8.7 Hz, 2H), 4.64 (s, 2H), 4.50 (m, *J* = 11.6, 8.8 Hz, 1H), 4.38 (m, *J* = 8.9, 1.5 Hz, 1H), 4.26 (m, *J* = 11.0, 9.1, 6.0 Hz, 1H), 2.59 (m, *J* = 12.4, 8.8, 6.0, 1.4 Hz, 1H), 2.19 (m, *J* = 12.4, 11.2, 8.8 Hz, 1H). ^{13}C NMR (126 MHz, Methanol-d$_4$) δ 175.92, 147.97, 137.41, 132.06, 122.98, 65.54, 58.97, 52.12, 30.09. ESI-EMM [M+Na]$^+$: calculated 323.0308; measured 323.0303.

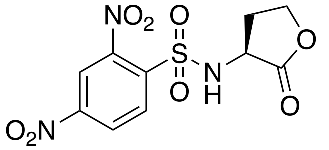

Compound 17: ^{1}H NMR (500 MHz, Methanol-d_4) δ 8.71 (d, *J* = 2.2 Hz, 1H), 8.56 (dd, *J* = 8.7, 2.3 Hz, 1H), 8.38 (d, *J* = 8.7 Hz, 1H), 4.57 (m, *J* = 11.5, 8.9 Hz, 1H), 4.34 (m, *J* = 11.1, 1.4 Hz, 1H), 4.22 (m, J = 11.1, 9.1, 6.1 Hz, 1H), 2.55 (m, J = 12.5, 9.0, 6.1, 1.4 Hz, 1H), 2.23 (m, J = 12.4, 11.3, 8.9 Hz, 1H). ^{13}C NMR (126 MHz, Methanol-d_4) δ 175.04, 150.05, 147.90, 139.51, 126.67, 120.09, 65.68, 52.33, 29.40. ESI-EMM $[M+NH_4]^+$: calculated 349.0449; measured 349.0447.

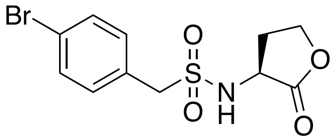

Compound 19: ^{1}H NMR (500 MHz, Methanol-d_4) δ 7.37 (d, *J* = 8.1 Hz, 2H), 7.19 (d, 2H), 4.42 (m, *J* = 1.9 Hz, 1H), 4.37 (m, 1H), 4.22 (m, *J* = 11.0, 9.1, 6.0 Hz, 1H), 2.51 (m, *J* = 12.5, 8.7, 6.0, 1.5 Hz, 1H), 2.34 (s, 3H), 2.15 (m, *J* = 12.4, 11.2, 8.8 Hz, 1H). ^{13}C NMR (126 MHz, Methanol-d_4) δ 177.30, 135.18, 133.50, 132.13, 130.95, 130.69, 129.49, 66.89, 60.40, 53.46, 31.42. ESI-EMM $[M+NH_4]^+$: calculated 351.0009; measured 351.0008.

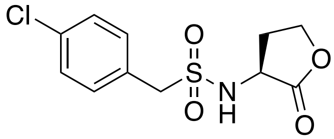

Compound 21: ^{1}H NMR (500 MHz, Chloroform-d) δ 7.49 (d, *J* = 8.5 Hz, 2H), 7.37 (d, *J* = 8.5 2H), 4.46 (m, 3H), 4.37 (m, *J* = 9.0, 1.5 Hz, 1H), 4.25 (m, *J* = 11.0, 9.1, 6.0 Hz, 1H), 2.56 (m, *J* = 12.5, 8.8, 6.0, 1.5 Hz, 1H), 2.34 (s, 3H), 2.18 (m, *J* = 12.4, 11.2, 8.8 Hz, 1H). ^{13}C NMR (126 MHz, Methanol-d_4) δ 175.91, 134.13, 132.44, 128.76, 128.19, 65.49, 58.87, 52.08, 30.11. ESI-EMM $[M+NH_4]^+$: calculated 307.0514; measured 307.0512.

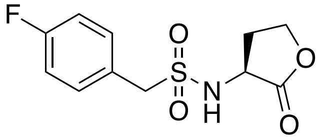

Compound 22: ^{1}H NMR (500 MHz, Methanol-d_4) δ 7.52 (dd, *J* = 8.6, 5.6 Hz, 2H), 7.10 (t, *J* = 8.8, 2H), 4.47(s, 2H), 4.44 (dd, *J* = 11.5, 8.8 Hz, 3H), 4.37 (td, *J* = 9.0, 1.5 Hz, 1H), 4.25 (m, *J* = 11.0, 9.1, 6.1 Hz, 1H), 2.55 (m, *J* = 12.6, 8.8, 6.0, 1.5 Hz, 1H), 2.18 (m, 1H). ^{13}C NMR (126 MHz, Methanol-d_4) δ 177.14, 165.16, 163.21, 134.06, 127.24, 116.05, 66.70, 59.99, 53.30, 31.36. ESI-EMM $[M+NH_4]^+$: calculated 291.0809; measured 291.0806.

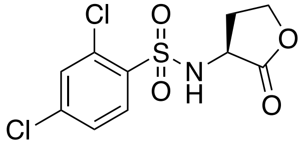

Compound 24: ^{1}H NMR (500 MHz, Methanol-d_4) δ 8.07 (d, *J* = 8.5, 1H), 7.68 (d, *J* = 2.1 Hz, 1H), 7.49 (dd, *J* = 8.5, 2.1 Hz, 1H), 4.35 (m, 2H), 4.19 (m, *J* = 11.0, 9.1, 6.1 Hz, 1H), 2.43 (m, 1H), 2.17 (m, 1H).

^{13}C NMR (126 MHz, Methanol-d_4) δ 175.0, 138.91, 137.55, 132.99, 131.51, 131.11, 127.13, 65.52, 51.76, 29.53. ESI-EMM $[M+NH_4]^+$: calculated 326.9968; measured 326.9964.

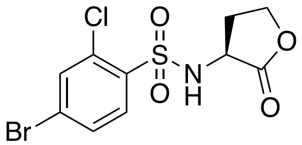

Compound 25: ^{1}H NMR (500 MHz, DMSO-d_6) δ 8.56 (d, *J* = 9.2 Hz, 1H), 7.99 (d, *J* = 1.9 Hz, 1H), 7.92 (dd, *J* = 8.5 Hz, 1H), 7.77 (dd, *J* = 8.5, 1.9 Hz, 1H), 4.41 (m, *J* = 11.3, 8.9 Hz, 1H), 4.27 (m, *J* = 8.9, 1.5 Hz, 1H), 4.12 (m, *J* = 11.3, 8.9, 1.5 Hz, 1H), 2.26 (m, 1H), 2.07 (m, 1H). ^{13}C NMR (126 MHz, Methanol-d_4) δ 174.82, 138.55, 134.29, 132.73, 132.06, 131.16, 127.13, 65.64, 51.96, 29.59. ESI-EMM $[M+NH_4]^+$: calculated 370.9462; measured 370.9462.

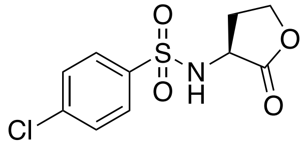

Compound 26: ^{1}H NMR (500 MHz, Methanol-d_4) δ 7.90 (m, 2H), 7.57 (m, 2H), 4.34 (m, 2H), 4.20 (m, *J* = 11.0, 9.1, 6.0 Hz, 1H), 2.45 (m, 1H), 2.05 (m, *J* = 12.5, 11.3, 8.8 Hz, 1H). ^{13}C NMR (126 MHz, Methanol-d_4) δ 175.05, 140.04, 138.56, 128.97, 128.46, 65.51, 51.63, 30.09. ESI-EMM $[M-H]^-$: calculated 273.9946; measured 273.9949.

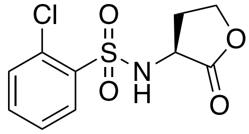

Compound 27: ^{1}H NMR (500 MHz, Methanol-d_4) δ 7.92 (t, *J* = 1.9 Hz, 1H), 7.84 (ddd, *J* = 7.8, 2.1, 1.0 Hz, 1H), 7.62 (ddd, *J* = 8.1, 2.1, 1.0 Hz, 1H), 7.54 (t, *J* = 7.9 Hz, 1H), 4.34 (m, 2H), 4.19 (m, *J* = 11.0, 9.1, 5.9, 1.4 Hz, 1H), 2.45 (m, *J* = 12.5, 8.6, 5.9, 1.4 Hz, 1H), 2.05 (m, *J* = 12.5, 11.2, 8.8 Hz, 1H). ^{13}C NMR (126 MHz, Methanol-d_4) δ 175.00, 143.23, 134.64, 132.43, 130.43, 126.62, 125.06, 65.50, 51.65, 30.11. ESI-EMM $[M+NH_4]^+$: calculated 293.0357; measured 293.0354.

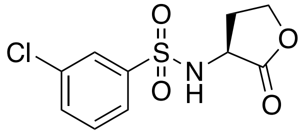

Compound 28: ^{1}H NMR (500 MHz, Methanol-d_4) δ 8.12 (m, 1H), 7.60 (m, 2H), 7.47 (ddd, *J* = 8.5, 6.5, 2.1 Hz, 1H), 4.34 (m, 2H), 4.17 (m, *J* = 11.0, 9.1, 6.1 Hz, 1H), 2.36 (m, *J* = 12.5, 8.8, 6.1, 1.5, 1H), 2.15 (m, *J* = 12.4, 11.2, 8.9 Hz, 1H). ^{13}C NMR (126 MHz, Methanol-d_4) δ 174.97, 138.53, 133.61, 131.78, 131.51, 130.32, 126.90, 65.43, 51.78, 29.53. ESI-EMM $[M-H]^-$: calculated 273.9946; measured 273.9948.

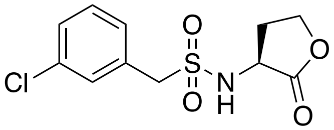

Compound 33: ^{1}H NMR (500 MHz, Methanol-d_4) δ 7.55 (m, 1H), 7.43 (m, 1H), 7.36 (m, 2H), 4.49 (m, 2H), 4.45 (m, 1H), 4.37 (m, *J* = 9.0, 1.5 Hz, 1H), 4.25 (m, *J* = 11.0, 9.1, 6.0 Hz, 1H), 2.56 (m, *J* = 12.4, 8.8, 6.0 Hz, 1H), 2.18 (m, *J* = 12.4, 11.2, 8.8 Hz, 1H). ^{13}C NMR (126 MHz, Methanol-d_4) δ 175.93, 133.82, 132.14, 130.77, 129.58, 129.33, 65.51, 59.03, 52.10, 30.06. ESI-EMM $[M+NH_4]^+$: calculated 307.0514; measured 307.0514.

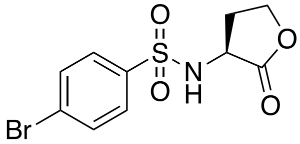

Compound 35: ^{1}H NMR (500 MHz, Methanol-d_4) δ 7.83 (d, *J* = 8.6 Hz, 1H), 7.73 (d, *J* = 8.7 Hz, 1H), 4.34 (m, 2H), 4.20 (m, *J* = 11.0, 9.1, 5.9 Hz, 1H), 2.45 (m, *J* = 12.5, 8.7, 5.9, 1.4 Hz, 1H), 2.05 (m, *J* = 12.5, 11.2, 8.8 Hz, 1H). ^{13}C NMR (126 MHz, Methanol-d_4) δ 175.03, 140.54, 132.02, 128.52, 126.92, 65.51, 51.63, 30.09. ESI-EMM $[M+NH_4]^+$: calculated 336.9852; measured 336.9851.

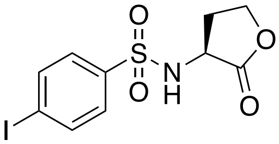

Compound 36: ^{1}H NMR (500 MHz, Methanol-d_4) δ 7.93 (d, *J* = 8.6 Hz, 2H), 7.65 (d, *J* = 8.6 Hz, 2H), 4.32 (m, 1H), 4.18 (m, *J* = 11.1, 9.1, 6.0 Hz, 1H), 2.43 (m, *J* = 12.6, 8.7, 5.9, 1.4 Hz, 1H), 2.04 (m, *J* = 12.5, 11.2, 8.8, 1H). ^{13}C NMR (126 MHz, Methanol-d_4) δ 175.05, 141.03, 138.15, 128.25, 99.24, 65.53, 51.68, 30.08. ESI-EMM $[M+NH_4]^+$: calculated 384.9714; measured 384.9714.

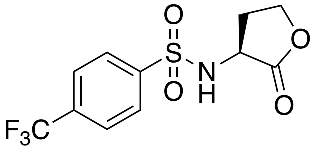

Compound 37: ^{1}H NMR (500 MHz, Chloroform-d) δ 7.90 (m, 2H), 7.62 (m, 2H), 5.28 (d, 1H), 4.43 (m, 1H), 4.20 (m, 1H), 3.97 (m, 1H), 2.71 (m, 1H), 2.27 (m, 1H). ^{13}C NMR (126 MHz, Chloroform-d) δ 173.91, 142.92, 134.95, 127.81, 126.50, 124.22, 122.05, 119.88, 66.03, 51.98, 31.15. ESI-EMM $[M+NH_4]^+$: calculated 327.0621; measured 327.0616.

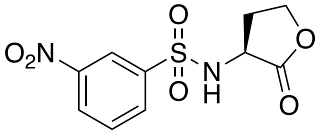

Compound 38: ^{1}H NMR (500 MHz, Methanol-d_4) δ 8.73 (m, *J* = 2.0 Hz, 1H), 8.47 (ddd, *J* = 8.2, 2.2, 1.0 Hz, 1H), 7.30 (t, *J* = 8.1 Hz, 1H), 4.47 (m, *J* = 11.4, 8.7 Hz, 1H), 4.34 (m, *J* = 9.0, 1.4 Hz, 1H), 4.22 (m, *J* = 11.0, 9.1,5.9 Hz, 1H), 2.53 (m, 1H), 2.11 (m, *J* = 12.5, 11.3, 8.8 Hz, 1H). ^{13}C NMR (126 MHz, Chloroform-d) δ 175.03, 148.27, 143.53, 132.40, 130.40, 126.66, 121.75, 65.53, 51.73, 30.10. ESI-EMM $[M+NH_4]^+$: calculated; measured. ESI-EMM: [M-H]- calculated 285.0187; measured 285.0187.

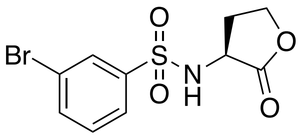

Compound 39: ^{1}H NMR (500 MHz, Methanol-d$_4$) δ 8.08 (t, 1H), 7.89 (ddd, *J* = 7.9, 1.8, 1.0 Hz, 1H), 7.78 (ddd, *J* = 8.0, 2.0, 1.0 Hz, 1H), 7.48 (t, *J* = 7.9 Hz, 1H), 4.35 (m, 2H), 4.20 (m, *J* = 11.0, 9.1, 5.9 Hz, 1H), 2.46 (m, *J* = 12.5, 8.7, 5.9, 1.4 Hz, 1H), 2.06 (m, *J* = 12.4, 11.2, 8.8, 1H). ^{13}C NMR (126 MHz, Methanol-d$_4$) δ 175.00, 143.34, 135.33, 130.64, 129.49, 125.47, 122.31, 65.51, 51.65, 30.11. ESI-EMM [M+NH$_4$]$^+$: calculated 336.9852; measured 336.9846.

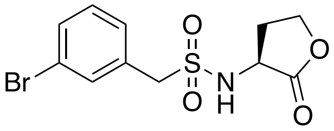

Compound 41: ^{1}H NMR (500 MHz, Methanol-d$_4$) δ 7.70 (t, *J* = 1.9 Hz, 1H), 7.51 (m, 2H), 7.29 (t, *J* = 7.9 Hz, 1H), 4.47 (m, 3H), 4.38 (m, *J* = 8.9, 1.5, 1H), 4.25 (m, *J* = 11.0, 9.1, 6.0 Hz, 1H), 2.56 (m, *J* = 12.4, 8.8, 6.0, 1.5 Hz, 1H), 2.18 (m, *J* = 12.5, 11.3, 8.8 Hz, 1H). ^{13}C NMR (126 MHz, Methanol-d$_4$) δ 175.77, 134.02, 133.34, 131.39, 130.87, 130.54, 121.82, 65.68, 58.84, 52.11, 30.15. ESI-EMM [M+NH$_4$]$^+$: calculated 351.0009; measured 351.0007.

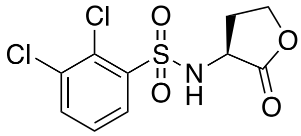

Compound 42: ^{1}H NMR (500 MHz, Methanol-d$_4$) δ 8.07 (dd, *J* = 8.0, 1.5 Hz, 1H), 7.78 (dd, *J* = 8.1, 1.6 Hz, 1H), 7.45 (t, *J* = 8.0 Hz, 1H), 4.40 (m, *J* = 11.4, 8.8 Hz, 1H), 4.32 (m, *J* = 9.0, 1.5 Hz, 1H), 4.18 (m, *J* = 11.0, 9.1, 6.1, 1H), 2.41 (m, *J* = 12.4, 8.8, 6.1, 1.5 Hz, 1H), 2.16 (m, 1H). ^{13}C NMR (126 MHz, Methanol-d$_4$) δ 173.34, 138.59, 135.97, 134.98, 130.43, 129.31, 127.46, 65.97, 52.19, 31.32. ESI-EMM [M+NH$_4$]$^+$: calculated 326.9968; measured 326.9963.

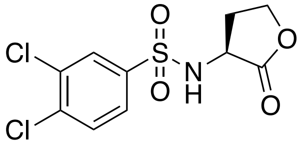

Compound 43: ^{1}H NMR (500 MHz, Methanol-d$_4$) δ 8.05 (d, *J* = 2.2 Hz, 1H), 7.81 (dd, *J* = 8.4, 2.1 Hz, 1H), 7.71 (d, *J* = 8.5 Hz, 1H), 4.33 (m, *J* = 9.0, 1.4 Hz, 1H), 4.20 (m, *J* = 11.1, 9.1, 5.9 Hz, 1H), 2.50 (m, *J* = 12.4, 8.7, 5.9, 1.4 Hz, 1H), 2.08 (m, *J* = 12.5, 11.3, 8.8 Hz, 1H). ^{13}C NMR (126 MHz, Methanol-d$_4$) δ 175.03, 141.65, 136.57, 132.80, 131.00, 128.75, 126.39, 65.54, 51.68, 30.11. ESI-EMM [M+NH$_4$]$^+$: calculated 326.9968; measured 326.9967.

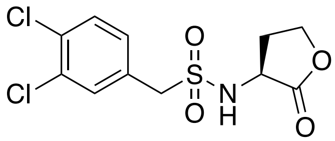

Compound 44: ^{1}H NMR (500 MHz, Methanol-d$_4$) δ 7.70 (d, *J* = 2.1 Hz, 1H), 7.51 (d, *J* = 8.3 Hz, 1H), 7.43 (dd, *J* = 8.3, 2.0 Hz, 1H), 4.47 (m, 3H), 4.38 (m, *J* = 8.9, 1,5 Hz, 1H), 4.26 (m, *J* = 11.0, 9.1, 6.0 Hz, 1H), 2.58 (m, *J* = 12.5, 8.8, 6.1, 1.5 Hz, 1H), 2.18 (m, *J* = 12.4, 11.2, 8.8 Hz, 1H). ^{13}C NMR (126

MHz, Methanol-d$_4$) δ 175.94, 132.78, 132.13, 131.85, 130.74, 130.11, 65.51, 58.33, 52.08, 30.07. ESI-EMM [M+NH$_4$]$^+$: calculated 341.0124; measured 341.0123.

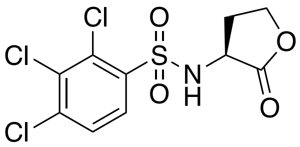

Compound 45: ^{1}H NMR (500 MHz, Methanol-d$_4$) δ 8.04 (d, *J* = 8.6 Hz, 1H), 7.67 (d, *J* = 8.7 Hz, 1H), 4.41 (dd, *J* = 11.4, 8.8 Hz, 1H), 4.33 (t, *J* = 9.0, 1.5 Hz, 1H), 4.19 (m, *J* = 11.0, 9.1, 6.0 Hz, 1H), 2.46 (m, *J* = 12.4, 8.8, 6.1, 1.5 Hz, 1H), 2.18 (m, 1H). ^{13}C NMR (126 MHz, Methanol-d$_4$) δ 175.0, 138.91, 137.55, 132.99, 131.51, 131.11, 127.13, 65.52, 51.76, 29.53. ESI-EMM [M+H]$^+$: calculated 343.9312; measured 343.9307.

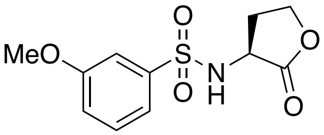

Compound 48: ^{1}H NMR (500 MHz, Methanol-d$_4$) δ 7.43 (m, 3H), 7.13 (ddd, *J* = 7.6, 2.5, 1.6 Hz, 1H), 4.27 (m, 2H), 4.14 (m, *J* = 11.0, 9.1, 5.9 Hz, 1H), 3.83 (s, 3H), 2.37 (m, *J* = 12.5, 8.6, 5.9,1.4 Hz, 1H), 2.00 (m, *J* = 12.5, 11.2, 8.8 Hz, 1H). ^{13}C NMR (126 MHz, Methanol-d$_4$) δ 175.07, 160.11, 142.20, 129.91, 118.59, 111.61, 65.50, 54.78, 51.62, 30.09. ESI-EMM [M-H]$^-$: calculated 270.0442; measured 270.0441.

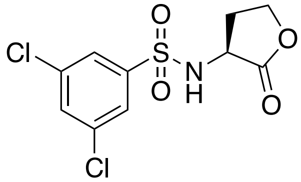

Compound 50: ^{1}H NMR (500 MHz, Methanol-d$_4$) δ 7.87 (d, *J* = 1.8 Hz, 2H), 7.72 (t, *J* = 1.9 Hz, 1H), 4.44 (m, *J* = 11.5, 8.7 Hz, 1H), 4.34 (m, *J* = 9.0, 1.4 Hz, 1H), 4.33 (m, *J* = 11.1, 9.1, 5.9 Hz, 1H), 2.52 (m, *J* = 12.4, 8.7, 5.9, 1.4 Hz, 1H), 2.10 (m, *J* = 12.4, 11.3, 8.8 Hz, 1H). ^{13}C NMR (126 MHz, Methanol-d$_4$) δ 174.98, 144.67, 135.53, 131.99, 125.28, 65.53, 51.71, 30.12. ESI-EMM [M+NH$_4$]$^+$: calculated 326.9968; measured 326.9966.

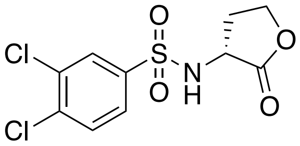

Compound 55: ^{1}H NMR (500 MHz, Methanol-d$_4$) δ 8.06 (d, *J* = 2.1 Hz, 1H), 7.82 (d, *J* = 8.4, 2.1 Hz, 1H), 7.72 (dd, *J* = 8.4 Hz, 1H), 4.41 (m, *J* = 9.0, 1.4 Hz, 1H), 4.34 (m, *J* = 11.0, 9.1, 5.9 Hz, 1H), 4.21 (m, *J* = 11.0, 9.1, 5.9 Hz, 1H), 2.51 (m, *J* = 12.5, 8.7, 5.9, 1.4 Hz, 1H), 2.09 (m, *J* = 12.4, 11.2, 8.8 Hz, 1H). ^{13}C NMR (126 MHz, Methanol-d$_4$) δ 175.04, 141.63, 136.58, 132.81, 131.01, 128.75, 126.40, 65.56, 51.69, 30.10. ESI-EMM [M+NH$_4$]$^+$: calculated 326.9968; measured 326.996.

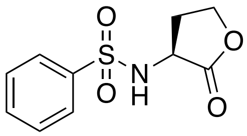

Compound 57: ^{1}H NMR (500 MHz, Methanol-d_4) δ 7.93 (m, 2H), 7.62 (m, *J* = 7.5 Hz, 1H), 7.57 (m, 1H), 4.39 (m, 2H), 4.18 (m, *J* = 11.1, 9.1, 6.0 Hz, 1H), 2.40 (m, 1H), 2.02 (m, *J* = 12.5, 11.2, 8.8 Hz, 1H). ^{13}C NMR (126 MHz, Methanol-d_4) δ 175.04, 141.15, 132.41, 128.80, 126.67, 65.45, 51.59, 30.08. ESI-EMM $[M+NH_4]^+$: calculated 259.0747; measured 259.0743.

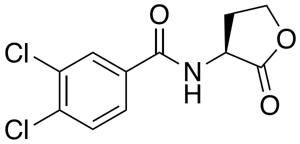

Compound 58: ^{1}H NMR (500 MHz, Methanol-d_4) δ 9.19 (d, 1H), 8.10 (d, *J* = 2.0 Hz, 1H), 7.83 (m, 2H), 4.80 (m, 1H), 4.43 (m, *J* = 8.8, 1.9 Hz, 1H), 4.29 (m, *J* = 10.5, 8.7, 6.6 Hz, 1H), 2.47 (m, 1H), 2.33 (m, *J* = 12.1, 10.6, 9.0 Hz, 1H). ^{13}C NMR (126 MHz, Methanol-d_4) δ 175.50, 164.31, 134.99, 134.23, 131.91, 131.41, 129.69, 128.05, 65.90, 49.06, 28.42. ESI-EMM $[M+NH_4]^+$: calculated 291.0298; measured 291.0297.

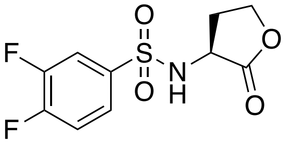

Compound 59: ^{1}H NMR (500 MHz, Methanol-d_4) δ 7.85 (ddd, *J* = 9.8, 7.3, 2.3 Hz, 1H), 7.76 (m, *J* = 8.8, 4.0, 2.3, 1.5 Hz, 1H), 7.48 (ddd, *J* = 10.1, 8.7, 7.6 Hz, 1H), 4.36 (m, 2H), 4.22 (m, *J* = 11.1, 9.1, 5.9 Hz, 1H), 2.50 (m, *J* = 12.6, 8.8, 5.9, 1.4 Hz, 1H), 2.09 (m, *J* = 12.5, 11.3, 8.8 Hz, 1H). ^{13}C NMR (126 MHz, Methanol-d_4) δ 175.09, 152.84, 149.90, 138.43, 124.31, 117.89, 116.62, 65.58, 51.68, 30.06. ESI-EMM $[M+Na]^+$: calculated 300.0113; measured 300.0105.

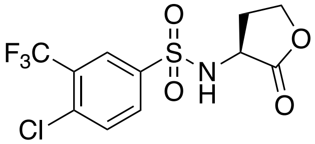

Compound 60: ^{1}H NMR (500 MHz, Methanol-d_4) δ 8.27 (d, *J* = 2.2 Hz, 1H), 8.12 (dd, *J* = 8.4, 2.2 Hz, 1H), 7.82 (d, *J* = 8.4 Hz, 1H), 4.45 (dd, *J* = 11.5, 8.7 Hz, 1H), 4.34 (m, *J* = 9.0, 1.4 Hz, 1H), 4.33 (m, *J* = 11.0, 9.1, 5.9 Hz, 1H), 2.53 (m, *J* = 12.5, 8.7, 5.9, 1.4 Hz, 1H), 2.12 (m, 1H). ^{13}C NMR (126 MHz, Methanol-d_4) δ 175.06, 141.18, 136.10, 132.01, 128.49, 126.21, 123.42, 123.42, 121.25, 65.55, 51.72, 30.10. ESI-EMM $[M+Na]^+$: calculated 365.9785; measured 365.9779.

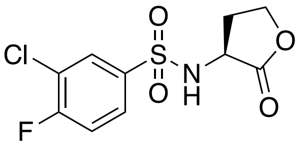

Compound 64: ^{1}H NMR (500 MHz, Methanol-d_4) δ 8.06 (dd, *J* = 6.8, 4.4, 2.3 Hz, 1H), 7.89 (ddd, *J* = 8.7, 4.4, 2.1 Hz, 1H), 7.44 (t, *J* = 8.8 Hz, 1H), 4.40 (m, *J* = 11.5, 8.6 Hz, 1H), 4.32 (m, *J* = 9.0, 1.4 Hz, 1H), 4.21 (m, *J* = 11.0, 8.6, 5.9, 1.4 Hz, 1H), 2.50 (m, *J* = 11.0, 9.1, 5.9 Hz, 1H), 2.08 (m, *J* = 12.4, 11.2, 8.8 Hz, 1H). ^{13}C NMR (126 MHz, Methanol-d_4) δ 175.03, 161.34, 159.31, 138.84, 129.66

127.90, 121.36, 117.06, 65.52, 51.65, 30.13. ESI-EMM $[M+NH_4]^+$: calculated 311.0263; measured 311.0262.

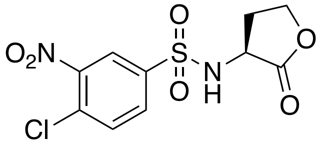

Compound 71: ^1H NMR (500 MHz, Methanol-d_4) δ 8.45 (d, *J* = 2.1 Hz, 1H), 8.12 (dd, *J* = 8.5, 2.2 Hz, 1H), 7.87 (d, *J* = 8.5 Hz, 1H), 4.49 (m, *J* = 11.5, 8.7 Hz, 1H), 4.35 (m, *J* = 8.9, 1.4 Hz, 1H), 4.23 (m, *J* = 11.1, 9.1, 5.9, 1.4 Hz, 1H), 2.56 (m, *J* = 12.5, 8.7, 5.9, 1.4 Hz, 1H), 2.13 (m, *J* = 12.5, 8.7, 5.9, 1.4 Hz, 1H). ^{13}C NMR (126 MHz, Methanol-d_4) δ 175.06, 142.03, 132.51, 131.07, 130.13, 124.03, 65.56, 51.77, 30.07. ESI-EMM $[M-H]^-$: calculated 318.9795; measured 318.9794.

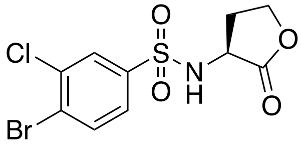

Compound 73: ^1H NMR (500 MHz, Methanol-d_4) δ 8.05 (d, *J* = 2.1 Hz, 1H), 7.89 (d, *J* = 8.4 Hz, 1H), 7.73 (dd, *J* = 8.4, 2.2 Hz, 1H), 4.41 (m, *J* = 11.5, 8.7 Hz, 1H), 4.34 (m, *J* = 8.9, 1.4 Hz, 1H), 4.21 (m, *J* = 11.1, 9.1, 5.9 Hz, 1H), 2.50 (m, 1H), 2.09 (m, *J* = 12.4, 11.2, 8.8 Hz, 1H). ^{13}C NMR (126 MHz, Methanol-d_4) δ 175.02, 142.36, 134.39, 128.42, 126.74, 126.30, 65.52, 51.68, 30.11. ESI-EMM $[M-H]^-$: calculated 351.9052; measured 351.9053.

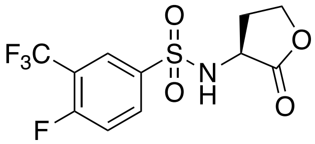

Compound 76: ^1H NMR (500 MHz, Methanol-d_4) δ 8.24 (m, 2H), 7.54 (m, 1H), 4.45 (m, *J* = 11.5, 8.7 Hz, 1H), 4.34 (m, *J* = 9.0, 1.4 Hz, 1H), 4.22 (m, *J* = 11.0, 9.1, 5.9 Hz, 1H), 2.53 (m, *J* = 12.4, 8.7, 5.9 Hz, 1H), 2.12 (m, *J* = 12.5, 11.3, 8.8 Hz, 1H). ^{13}C NMR (126 MHz, Methanol-d_4) δ 175.08, 161.70, 138.62, 133.70, 126.55, 121.67, 118.40, 117.79, 65.53, 51.69, 30.12. ESI-EMM $[M+NH_4]^+$: calculated 345.0527; measured 345.0526.

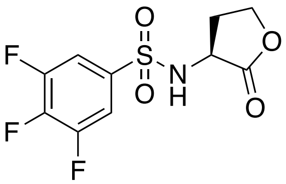

Compound 86: ^1H NMR (500 MHz, Methanol-d_4) δ 7.72 (dd, *J* = 7.3, 6.3 Hz, 2H), 4.44 (m, *J* = 11.5, 8.7 Hz, 1H), 4.35 (m, *J* = 8.9, 1.4 Hz, 1H), 4.22 (m, *J* = 11.1, 9.1, 5.9 Hz, 1H), 2.54 (m, *J* = 12.5, 8.7, 5.9, 1.4 Hz, 1H), 2.11 (m, *J* = 12.5, 11.3, 8.8 Hz, 1H). ^{13}C NMR (126 MHz, Methanol-d_4) δ 175.01, 151.82, 149.81, 143.31, 141.26, 138.08, 112.14, 65.54, 51.72, 30.10. ESI-EMM $[M+NH_4]^+$: calculated 313.0464; measured 313.0461.

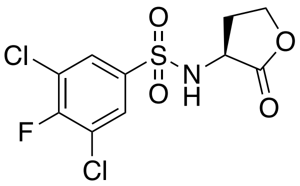

Compound 87: ^{1}H NMR (500 MHz, DMSO-d_6) δ 8.66 (d, *J* = 8.7 Hz, 1H), 8.02 (d, *J* = 6.2 Hz, 2H), 4.53 (m, *J* = 11.5, 8.7 Hz, 1H), 4.30 (m, *J* = 8.8, 1.3 Hz, 1H), 4.14 (m, *J* = 11.1, 8.8, 5.9 Hz, 1H), 2.35 (m, *J* = 12.2, 8.7, 5.9, 1.3 Hz, 1H), 1.96 (m, 1H). ^{13}C NMR (126 MHz, DMSO-d_6) δ 174.92, 157.01, 154.99, 139.70, 128.30, 122.54, 65.83, 51.98, 30.00. ESI-EMM $[M+NH_4]^+$: calculated 344.9873; measured 344.9870.

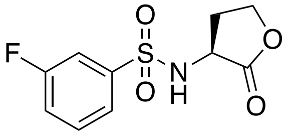

Compound 88: ^{1}H NMR (500 MHz, Methanol-d_4) δ 7.75 (ddd, *J* = 7.8, 1.7, 0.9 Hz, 1H), 7.66 (ddd, *J* = 8.6, 2.6, 1.7 Hz, 1H), 7.59 (td, *J* = 8.1, 5.4 Hz, 1H), 7.38 (tdd, *J* = 8.6, 2.6, 0.9 Hz, 1H), 4.35 (m, 2H), 4.20 (m, *J* = 11.0, 9.1, 5.9 Hz, 1H), 2.46 (m, 1H), 2.06 (m, *J* = 12.5, 11.3, 8.8 Hz, 1H). ^{13}C NMR (126 MHz, Methanol-d_4) δ 175.02, 162.44, 143.42, 130.94, 122.67, 119.36, 113.80, 65.52, 51.66, 30.07. ESI-EMM $[M+NH_4]^+$: calculated 277.0653; measured 277.0653.

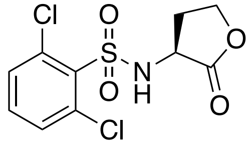

Compound 89: ^{1}H NMR (500 MHz, Methanol-d_4) δ 7.51 (m, 2H), 7.41 (dd, *J* = 8.7, 7.4 Hz, 1H), 4.41 (m, *J* = 11.3, 8.8 Hz, 1H), 4.30 (m, *J* = 9.0, 1.6 Hz, 1H), 4.18 (m, *J* = 10.8, 9.1, 6.1 Hz, 1H), 2.42 (m, *J* = 12.5, 8.9, 6.1,1.5 Hz, 1H), 2.17 (m, *J* = 12.4, 11.1, 8.9 Hz, 1H). ^{13}C NMR (126 MHz, Methanol-d_4) δ 174.90, 136.40, 134.64, 132.63, 131.26, 65.60, 51.89, 29.26. ESI-EMM $[M-H]^-$: calculated 307.9557; measured 307.9558

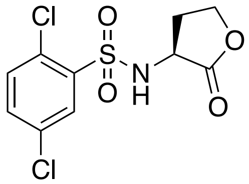

Compound 90: ^{1}H NMR (500 MHz, Methanol-d4) δ 8.06 (dd, *J* = 2.0, 0.9 Hz, 1H), 7.55 (t, *J* = 1.3 Hz, 2H), 4.39 (m, *J* = 11.4, 8.8 Hz, 1H), 4.30 (m, *J* = 8.9, 1.4 Hz, 1H), 4.16 (m, *J* = 11.0, 9.1, 6.0 Hz, 1H), 2.41 (m, *J* = 12.4, 8.8, 6.1, 1.5 Hz, 1H), 2.05 (m, *J* = 12.5, 11.2, 8.9 Hz, 1H). ^{13}C NMR (126 MHz, Methanol-d4) δ 174.96, 140.19, 133.29, 132.92, 132.64, 130.40, 129.88, 65.49, 51.79, 29.55. ESI-EMM $[M-H]^-$: calculated 307.9557; measured 307.9557.

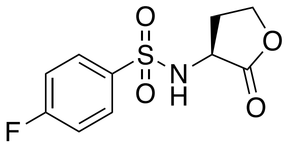

Compound 91: ^{1}H NMR (500 MHz, Methanol-d_4) δ 7.97 (dd, *J* = 8.9, 5.1 Hz, 2H), 7.29 (t, *J* = 8.8 Hz, 2H), 4.33 (m, 2H), 4.20 (m, *J* = 11.1, 9.1, 6.0 Hz, 1H), 2.44 (m, *J* = 12.5, 8.7, 6.0, 1.5 Hz, 1H), 2.05 (m, *J* = 12.4, 11.2, 8.8 Hz, 1H). ^{13}C NMR (126 MHz, Methanol-d_4) δ 175.13, 165.17, 137.40, 129.70, 115.82, 65.57, 51.63, 30.06. ESI-EMM $[M+NH_4]^-$: calculated 277.0653; measured 277.0653.

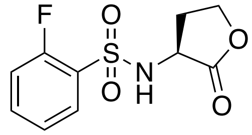

Compound 92: ^{1}H NMR (500 MHz, Methanol-d_4) δ 7.91 (td, *J* = 7.6, 1.9 Hz, 1H), 7.66 (m, 1H), 7.32 (m, 2H), 4.43 (m, *J* = 11.5, 8.8 Hz, 1H), 4.33 (m, *J* = 9.0, 1.4 Hz, 1H), 4.20 (m, *J* = 11.0, 9.1, 6.0 Hz, 1H), 2.44 (m, *J* = 12.4, 8.7, 5.9, 1.4 Hz, 1H), 2.13 (m, *J* = 12.5, 11.3, 8.8 Hz, 1H). ^{13}C NMR (126 MHz, Methanol-d_4) δ 175.09, 160.17, 158.15, 134.94, 129.37, 124.11, 116.68, 65.45, 51.71, 29.76. ESI-EMM $[M+NH_4]^+$: calculated 277.0653; measured 277.0651.

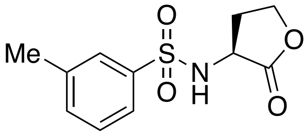

Compound 93: ^{1}H NMR (500 MHz, Methanol-d_4) δ 7.79 (m, 1H), 7.75 (m, *J* = 5.1, 3.1, 1.9 Hz, 1H), 7.47 (m, 1H), 4.34 (m, 2H), 4.21 (m, *J* = 11.1, 9.1, 5.9 Hz, 1H), 2.44 (s, 3H), 2.41 (m, 1H), 2.05 (m, *J* = 12.5, 11.2, 8.8 Hz, 1H). ^{13}C NMR (126 MHz, Methanol-d_4) δ 175.14, 140.88, 139.33, 133.18, 128.74, 126.99, 123.83, 65.55, 51.62, 30.04, 19.98. ESI-EMM $[M+NH_4]^+$: calculated 273.0904; measured 273.0901.

References.

[1] Geske, G. D., O'Neill, J. C., and Blackwell, H. E. (2007) *N*-phenylacetanoyl-L-homoserine lactones can strongly antagonize or superagonize quorum sensing in *Vibrio fischeri*, *ACS Chem. Biol. 2*, 315-319. https://doi.org/10.1021/cb700036x

[2] Geske, G. D., O'Neill, J. C., Miller, D. M., Mattmann, M. E., and Blackwell, H. E. (2007) Modulation of bacterial quorum sensing with synthetic ligands: systematic evaluation of *N*-acylated homoserine lactones in multiple species and new insights into their mechanisms of action, *J. Am. Chem. Soc. 129*, 13613-13625. https://doi.org/10.1021/ja074135h

[3] Lindsay, A., and Ahmer, B. M. (2005) Effect of sdiA on biosensors of *N*-acylhomoserine lactones, *J. Bacteriol. 187*, 5054-5058. https://doi.org/10.1128/JB.187.14.5054-5058.2005

[4] Welsh, M. A., Eibergen, N. R., Moore, J. D., and Blackwell, H. E. (2015) Small molecule disruption of quorum sensing cross-regulation in *Pseudomonas aeruginosa* causes major and unexpected alterations to virulence phenotypes, *J. Am. Chem. Soc. 137*, 1510-1519. https://doi.org/10.1021/ja5110798

[5] Brint, J. M., and Ohman, D. E. (1995) Synthesis of multiple exoproducts in *Pseudomonas aeruginosa* is under the control of RhlR-RhlI, another set of regulators in strain PAO1 with homology to the autoinducer-responsive LuxR-LuxI family, *J. Bacteriol. 177*, 7155-7163. https://doi.org/10.1128/jb.177.24.7155-7163.1995

[6] Rahme, L. G., Stevens, E. J., Wolfort, S. F., Shao, J., Tompkins, R. G., and Ausubel, F. M. (1995) Common virulence factors for bacterial pathogenicity in plants and animals, *Science 268*, 1899-1902. https://doi.org/10.1126/science.7604262

[7] Feltner, J. B., Wolter, D. J., Pope, C. E., Groleau, M. C., Smalley, N. E., Greenberg, E. P., Mayer-Hamblett, N., Burns, J., Deziel, E., Hoffman, L. R., and Dandekar, A. A. (2016) LasR Variant Cystic Fibrosis Isolates Reveal an Adaptable Quorum-Sensing Hierarchy in *Pseudomonas aeruginosa*, *mBio 7*, e01513-01516. https://doi.org/10.1128/mBio.01513-16

[8] Chugani, S., Kim, B. S., Phattarasukol, S., Brittnacher, M. J., Choi, S. H., Harwood, C. S., and Greenberg, E. P. (2012) Strain-dependent diversity in the *Pseudomonas aeruginosa* quorum-sensing regulon, *Proc. Natl. Acad. Sci. U. S. A. 109*, E2823-2831. https://doi.org/10.1073/pnas.1214128109

[9] Cabeen, M. T. (2014) Stationary phase-specific virulence factor overproduction by a lasR mutant of *Pseudomonas aeruginosa*, *PLoS One 9*, e88743. https://doi.org/10.1371/journal.pone.0088743

[10] Styles, M. J., Boursier, M. E., McEwan, M. A., Santa, E. E., Mattmann, M. E., Slinger, B. L., and Blackwell, H. E. (2022) Autoinducer-fluorophore conjugates enable FRET in LuxR proteins in vitro and in cells, *Nat. Chem. Biol. 18*, 1115-1124. https://doi.org/10.1038/s41589-022-01089-1

[11] Moore, J. D., Rossi, F. M., Welsh, M. A., Nyffeler, K. E., and Blackwell, H. E. (2015) A Comparative Analysis of Synthetic Quorum Sensing Modulators in *Pseudomonas aeruginosa*: New Insights into Mechanism, Active Efflux Susceptibility, Phenotypic Response, and Next-Generation Ligand Design, *J. Am. Chem. Soc. 137*, 14626-14639. https://doi.org/10.1021/jacs.5b06728

[12] Borgert, S. R., Henke, S., Witzgall, F., Schmelz, S., Zur Lage, S., Hotop, S. K., Stephen, S., Lubken, D., Kruger, J., Gomez, N. O., van Ham, M., Jansch, L., Kalesse, M., Pich, A., Bronstrup, M., Haussler, S., and Blankenfeldt, W. (2022) Moonlighting chaperone activity of the enzyme PqsE contributes to RhlR-controlled virulence of *Pseudomonas aeruginosa*, *Nat. Commun. 13*, 7402. https://doi.org/10.1038/s41467-022-35030-w

[13] Castang, S., Chantegrel, B., Deshayes, C., Dolmazon, R., Gouet, P., Haser, R., Reverchon, S., Nasser, W., Hugouvieux-Cotte-Pattat, N., and Doutheau, A. (2004) *N*-Sulfonyl homoserine lactones as antagonists of bacterial quorum sensing, *Bioorg. Med. Chem. Lett. 14*, 5145-5149. https://doi.org/10.1016/j.bmcl.2004.07.088

NMR spectral data for compounds.

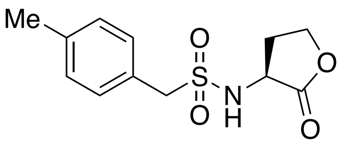

13

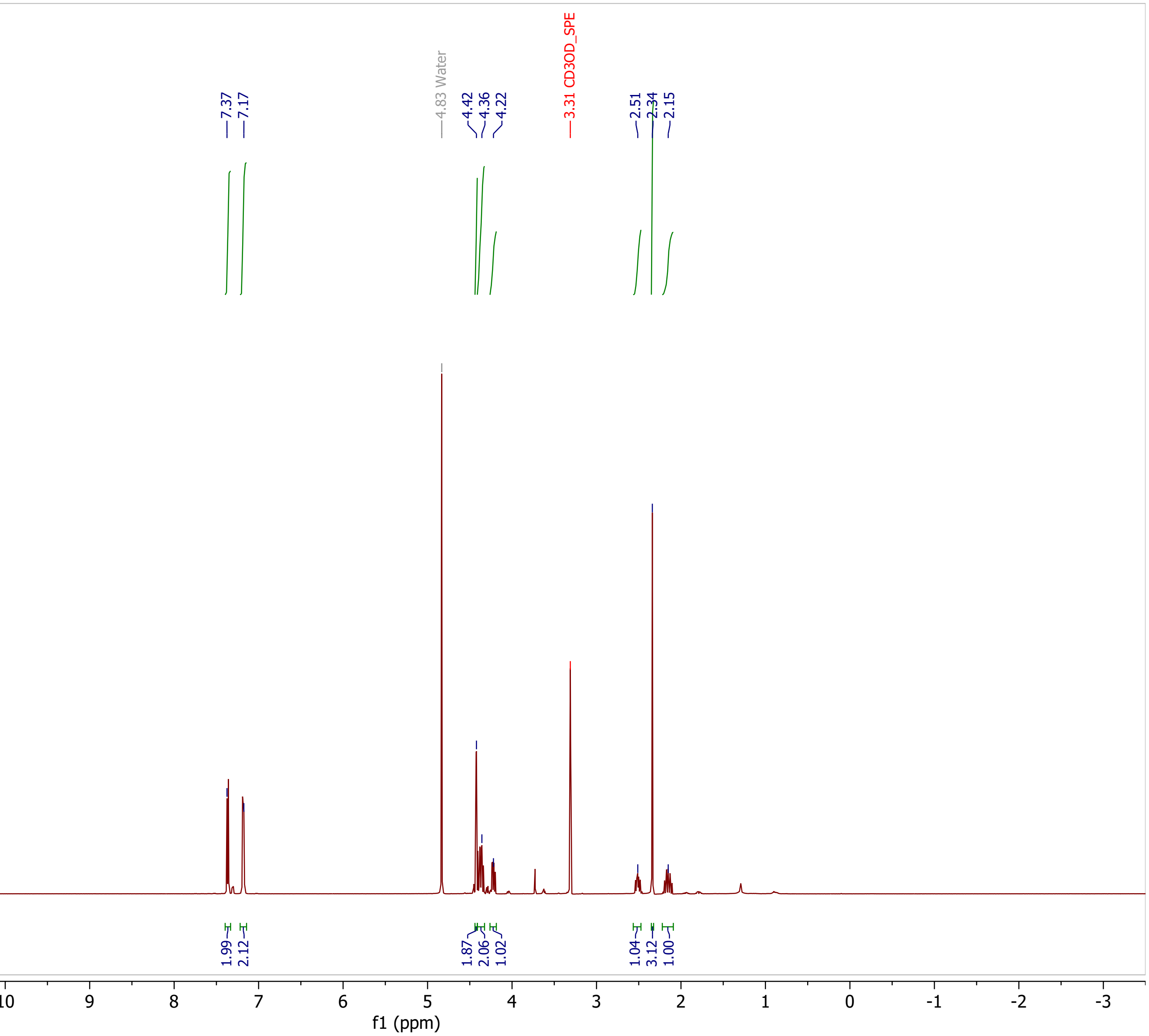

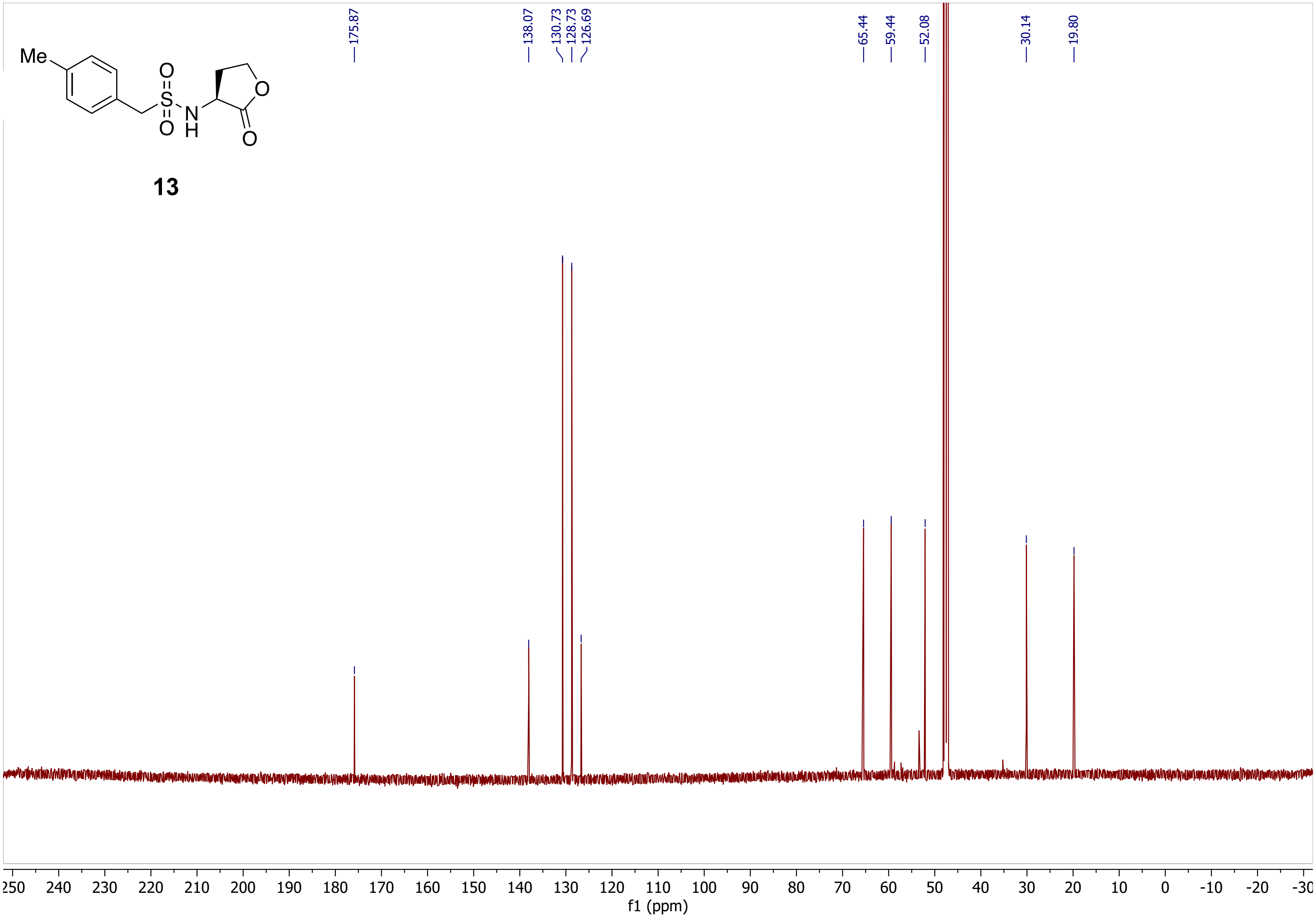
Me
O
S
O
N
H
O
O
13
175.87
138.07
130.73
128.73
126.69
65.44
59.44
52.08
30.14
19.80
250 240 230 220 210 200 190 180 170 160 150 140 130 120 110 100 90 80 70 60 50 40 30 20 10 0 -10 -20 -30
f1 (ppm)

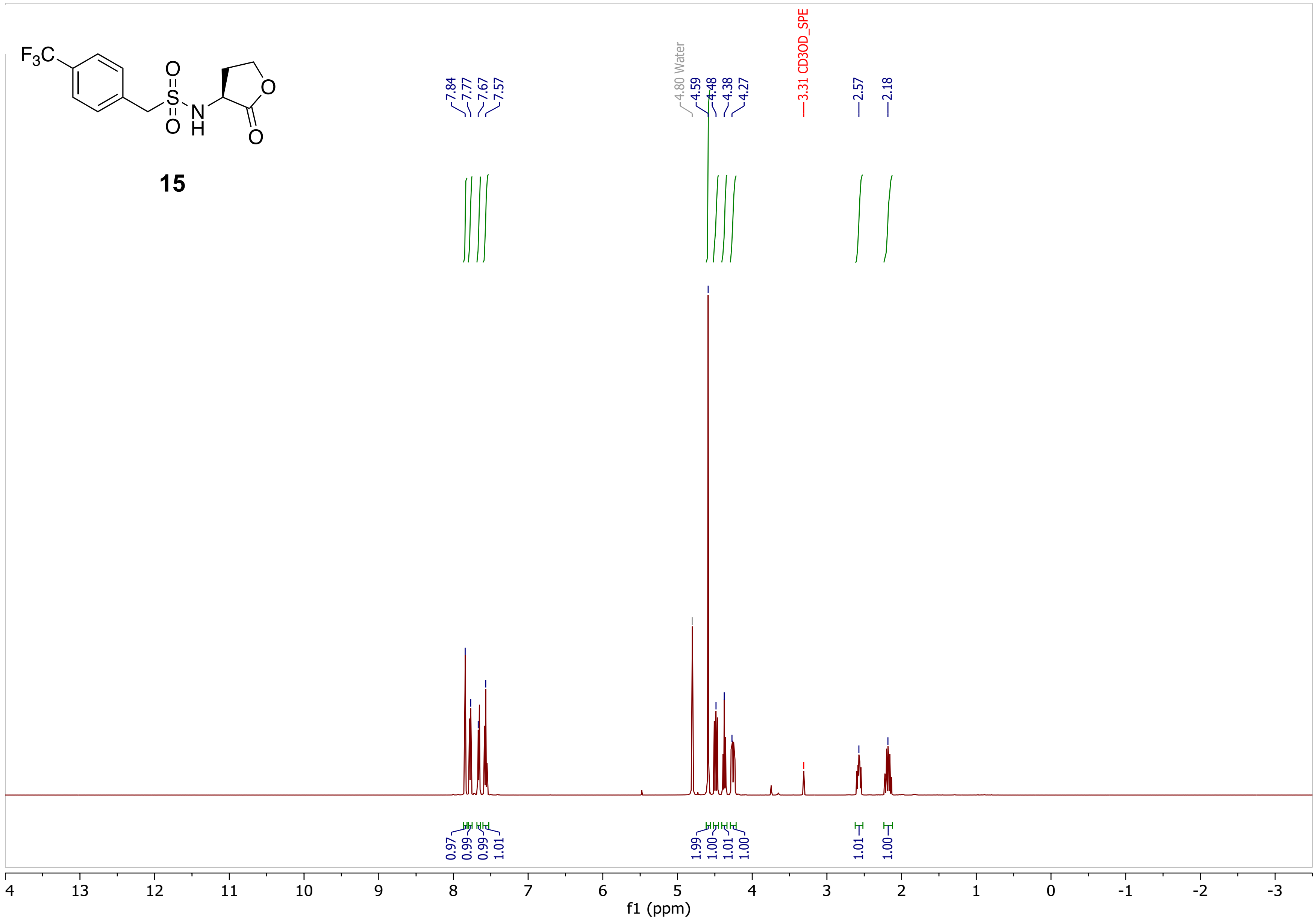

F3C
15
7.84
7.77
7.67
7.57
4.80 Water
4.59
4.48
4.38
4.27
3.31 CD3OD_SPE
2.57
2.18
0.97
0.99
0.99
1.01
1.99
1.00
1.01
1.00
1.01
1.00
f1 (ppm)

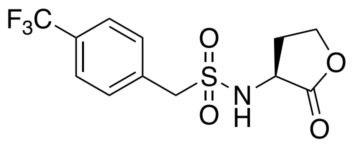
F3C
O
S
O
N
H
O
O

15

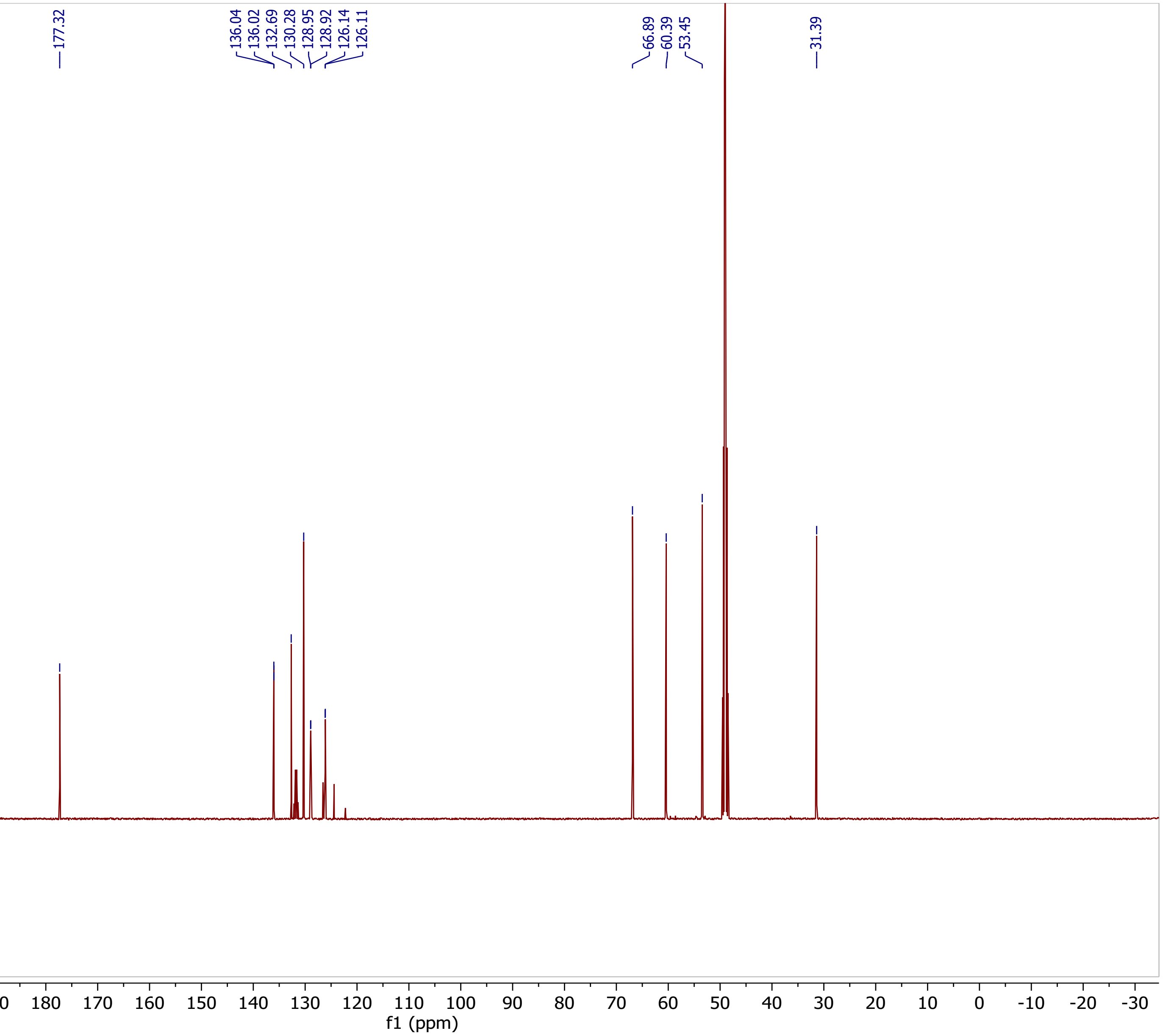
177.32
136.04
136.02
132.69
130.28
128.95
128.92
126.14
126.11
66.89
60.39
53.45
31.39
240 230 220 210 200 190 180 170 160 150 140 130 120 110 100 90 80 70 60 50 40 30 20 10 0 -10 -20 -30
f1 (ppm)

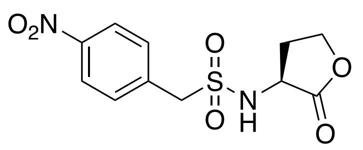

16

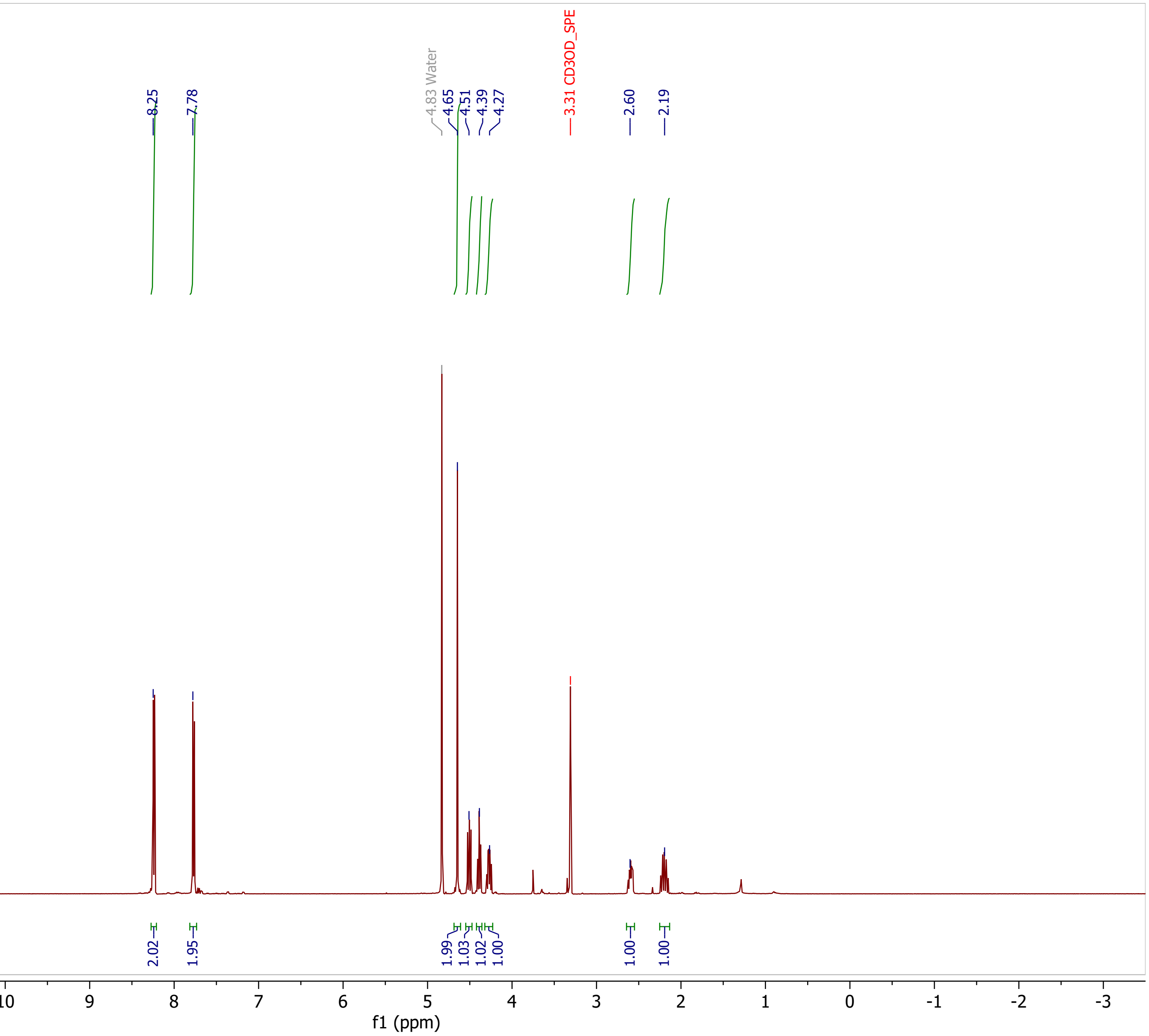
8.25
7.78
4.83 Water
4.65
4.51
4.39
4.27
3.31 CD3OD_SPE
2.60
2.19
2.02
1.95
1.99
1.03
1.02
1.00
1.00
1.00
14
13
12
11
10
9
8
7
6
5
4
3
2
1
0
-1
-2
-3
f1 (ppm)

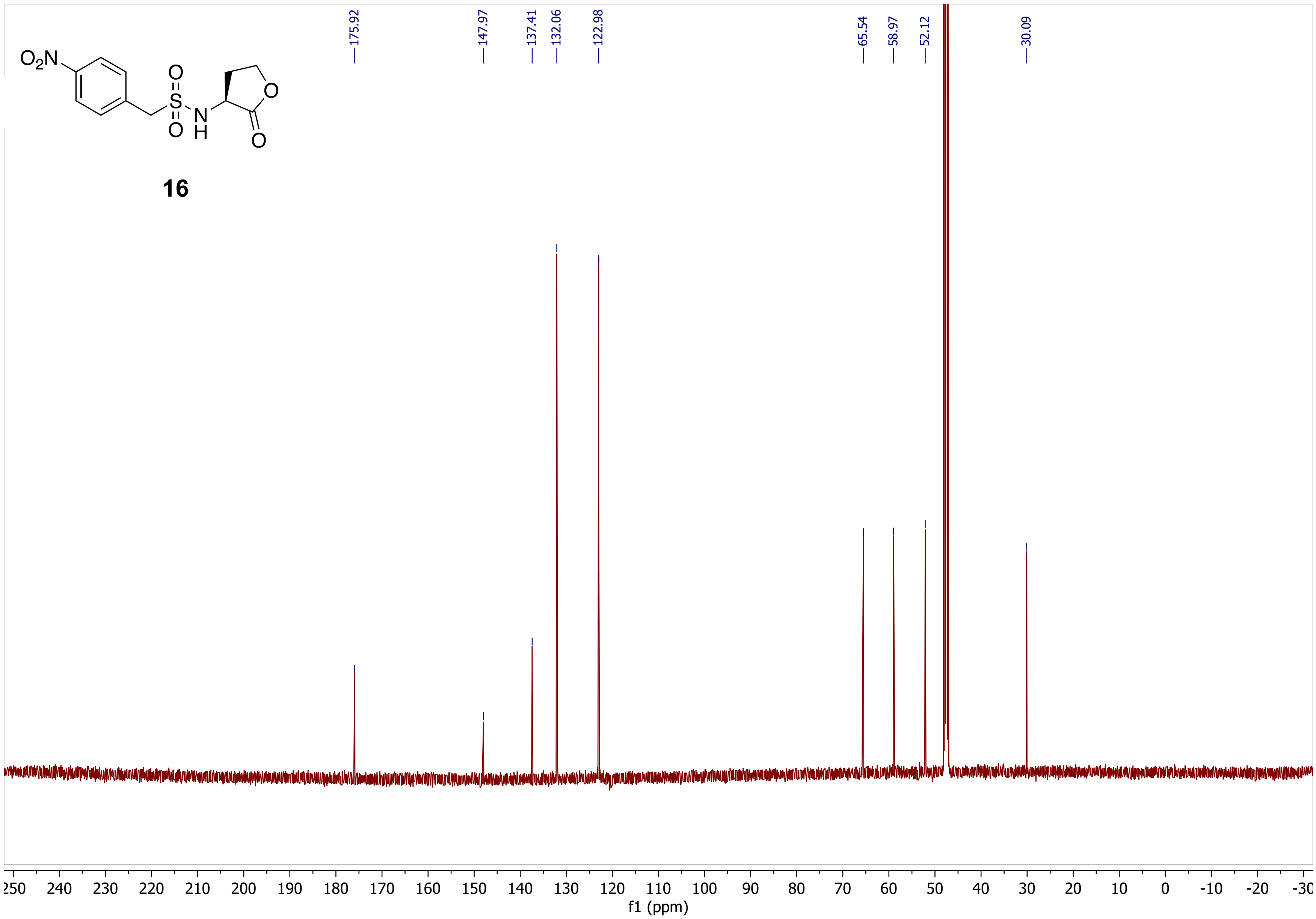

16
O_2N
175.92
147.97
137.41
132.06
122.98
65.54
58.97
52.12
30.09
f1 (ppm)

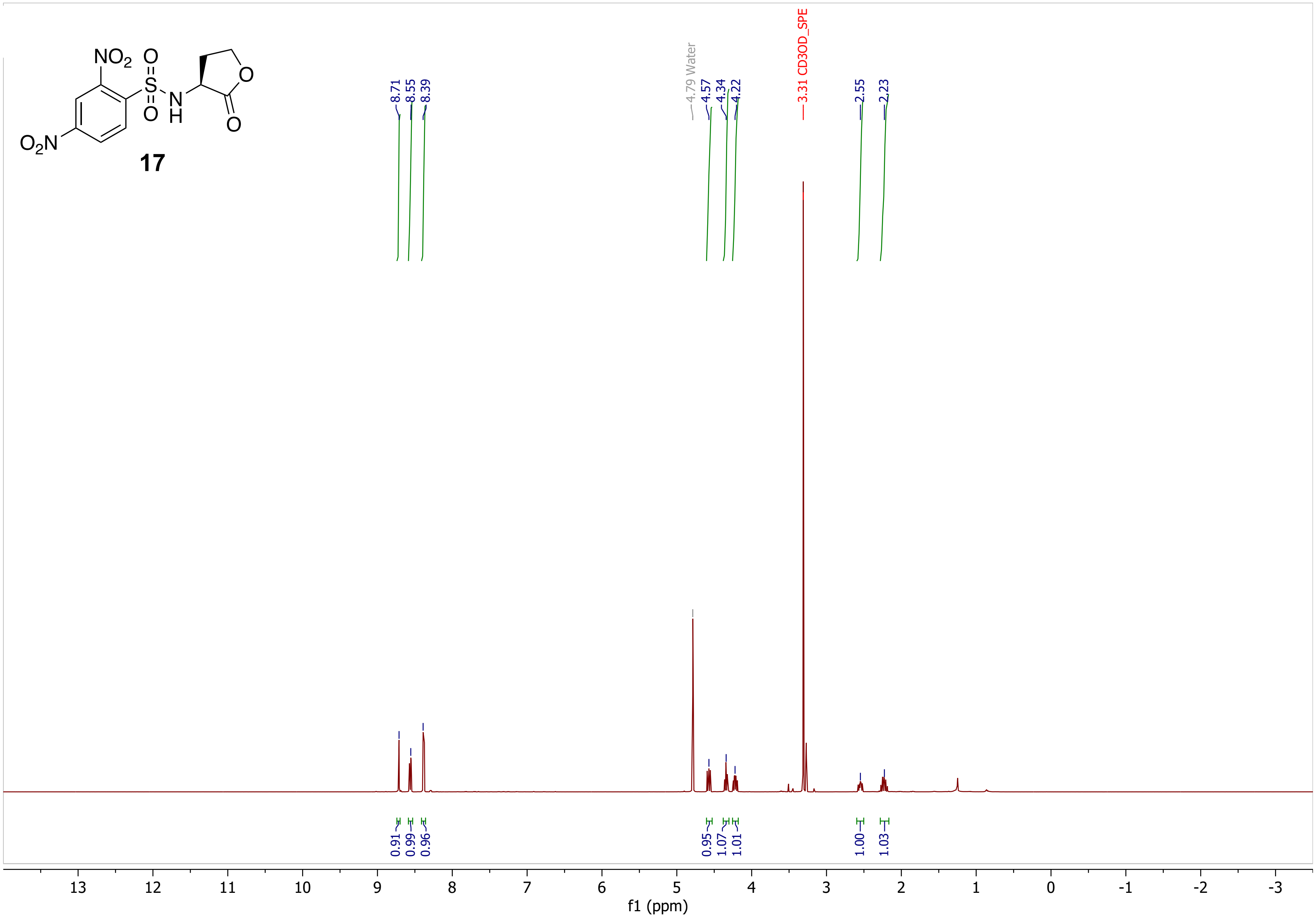
NO2
O
S
O
N
H
O
O
O2N
17
8.71
8.55
8.39
4.79 Water
4.57
4.34
4.22
3.31 CD3OD_SPE
2.55
2.23
0.91
0.99
0.96
0.95
1.07
1.01
1.00
1.03
13
12
11
10
9
8
7
6
5
4
3
2
1
0
-1
-2
-3
f1 (ppm)

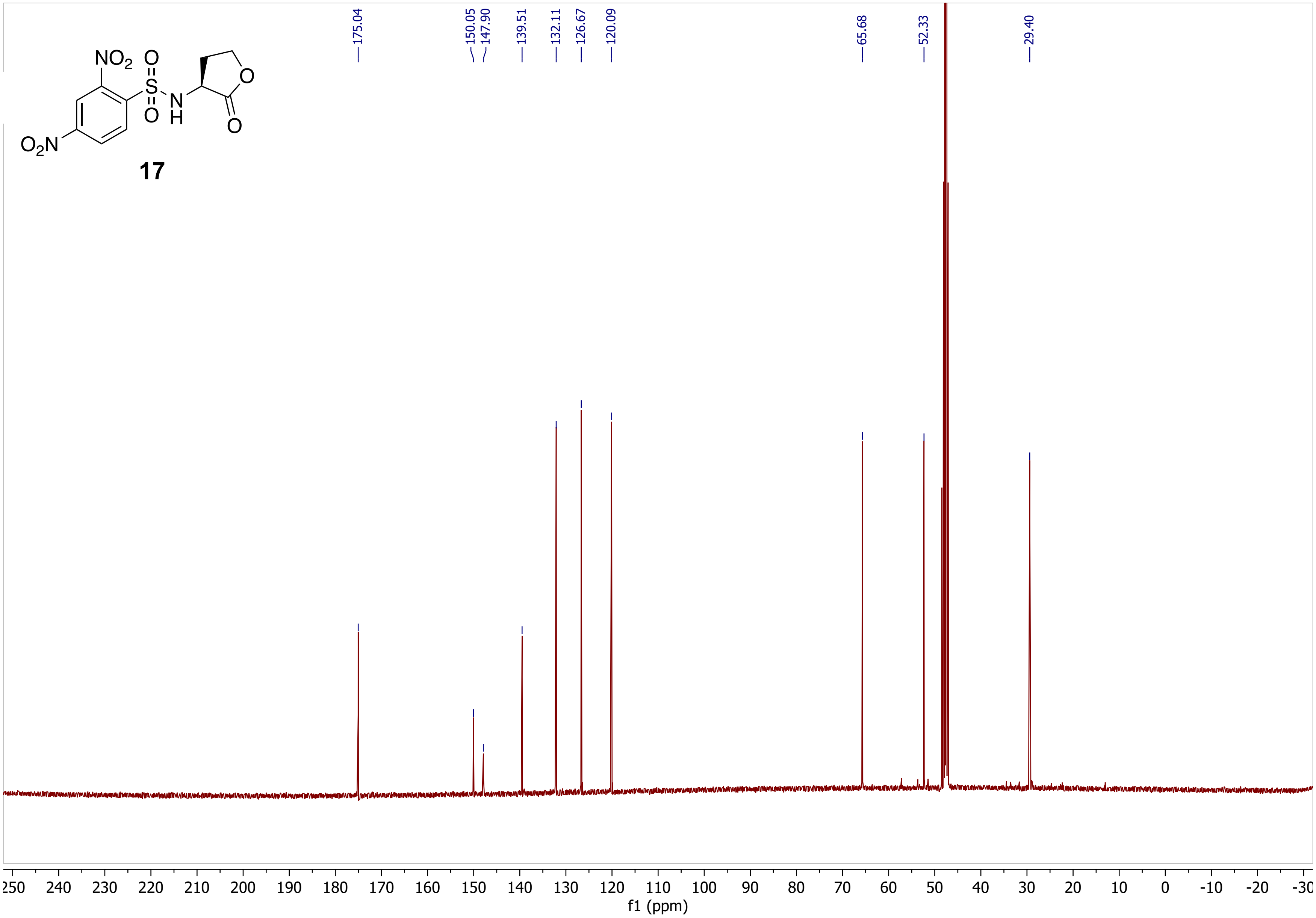
17
175.04
150.05
147.90
139.51
132.11
126.67
120.09
65.68
52.33
29.40
250 240 230 220 210 200 190 180 170 160 150 140 130 120 110 100 90 80 70 60 50 40 30 20 10 0 -10 -20 -30
f1 (ppm)

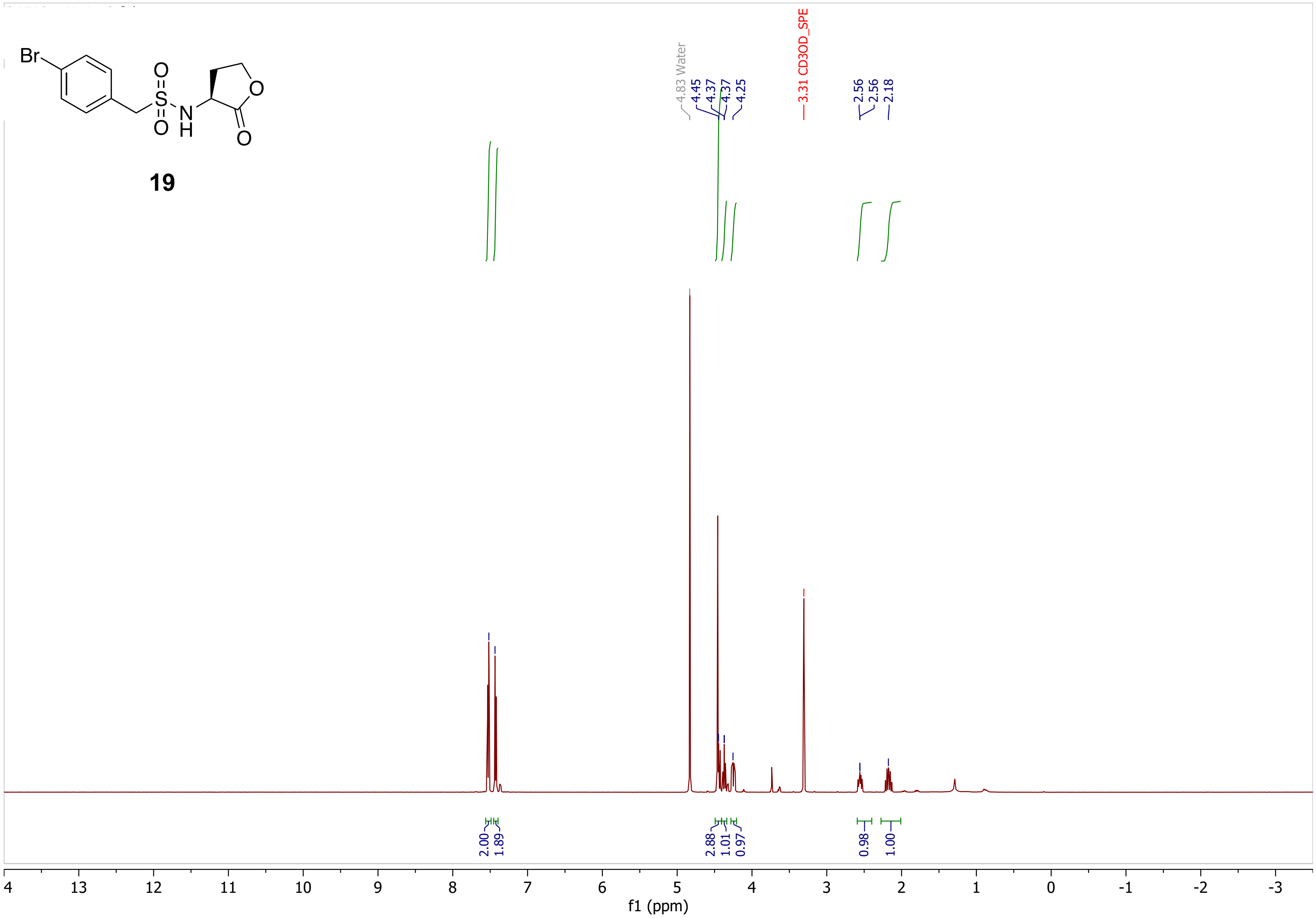

Br
O
S
O
N
H
O
O
19
4.83 Water
4.45
4.37
4.37
4.25
3.31 CD3OD_SPE
2.56
2.56
2.18
2.00
1.89
2.88
1.01
0.97
0.98
1.00
4
13
12
11
10
9
8
7
6
5
4
3
2
1
0
-1
-2
-3
f1 (ppm)

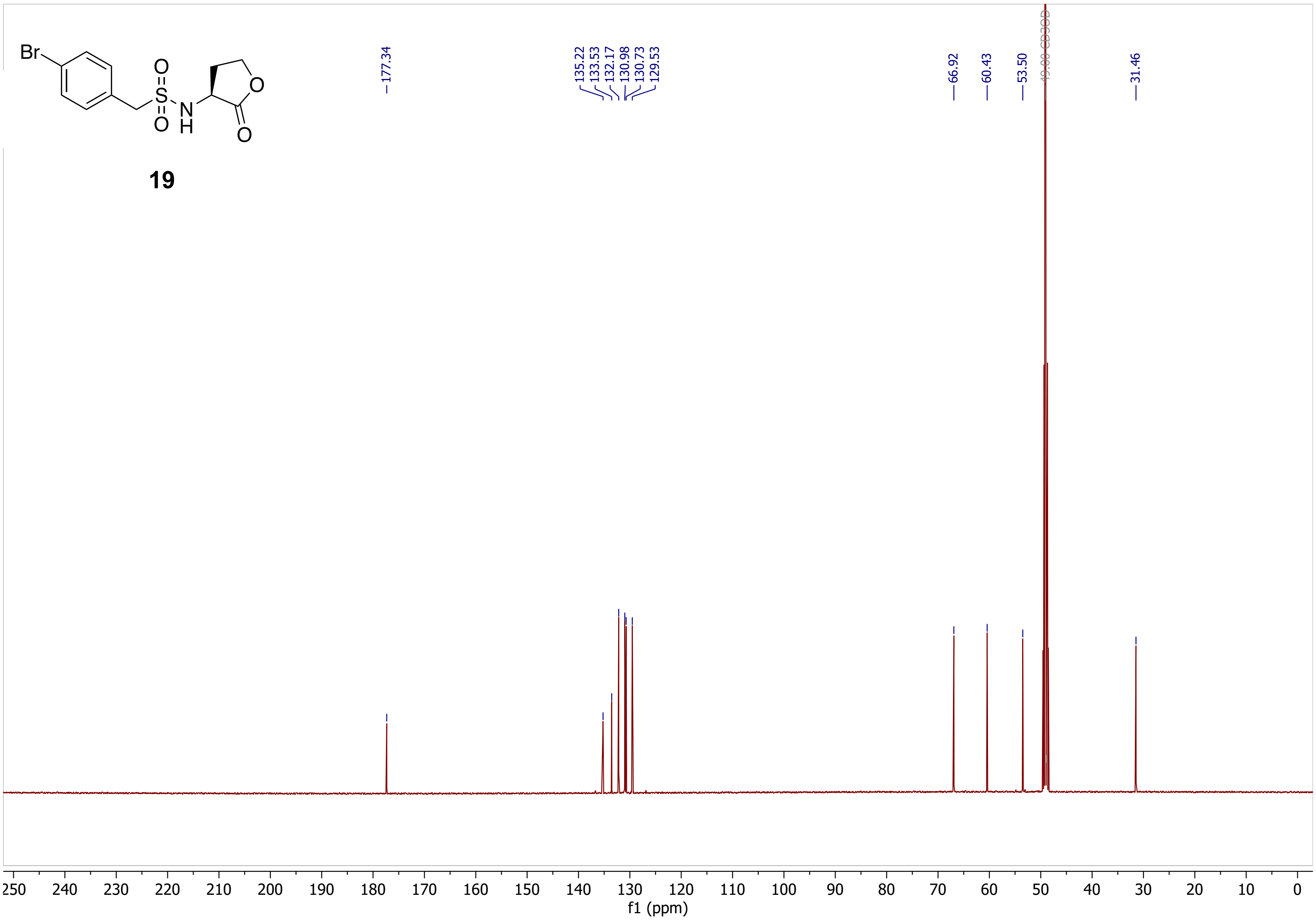

Br
O
S
O
N
H
O
O
19
177.34
135.22
133.53
132.17
130.98
130.73
129.53
66.92
60.43
53.50
49.00 CD3OD
31.46
250 240 230 220 210 200 190 180 170 160 150 140 130 120 110 100 90 80 70 60 50 40 30 20 10 0
f1 (ppm)

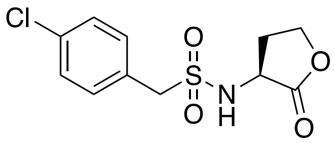
Cl
O
S
O
N
H
O
O

21

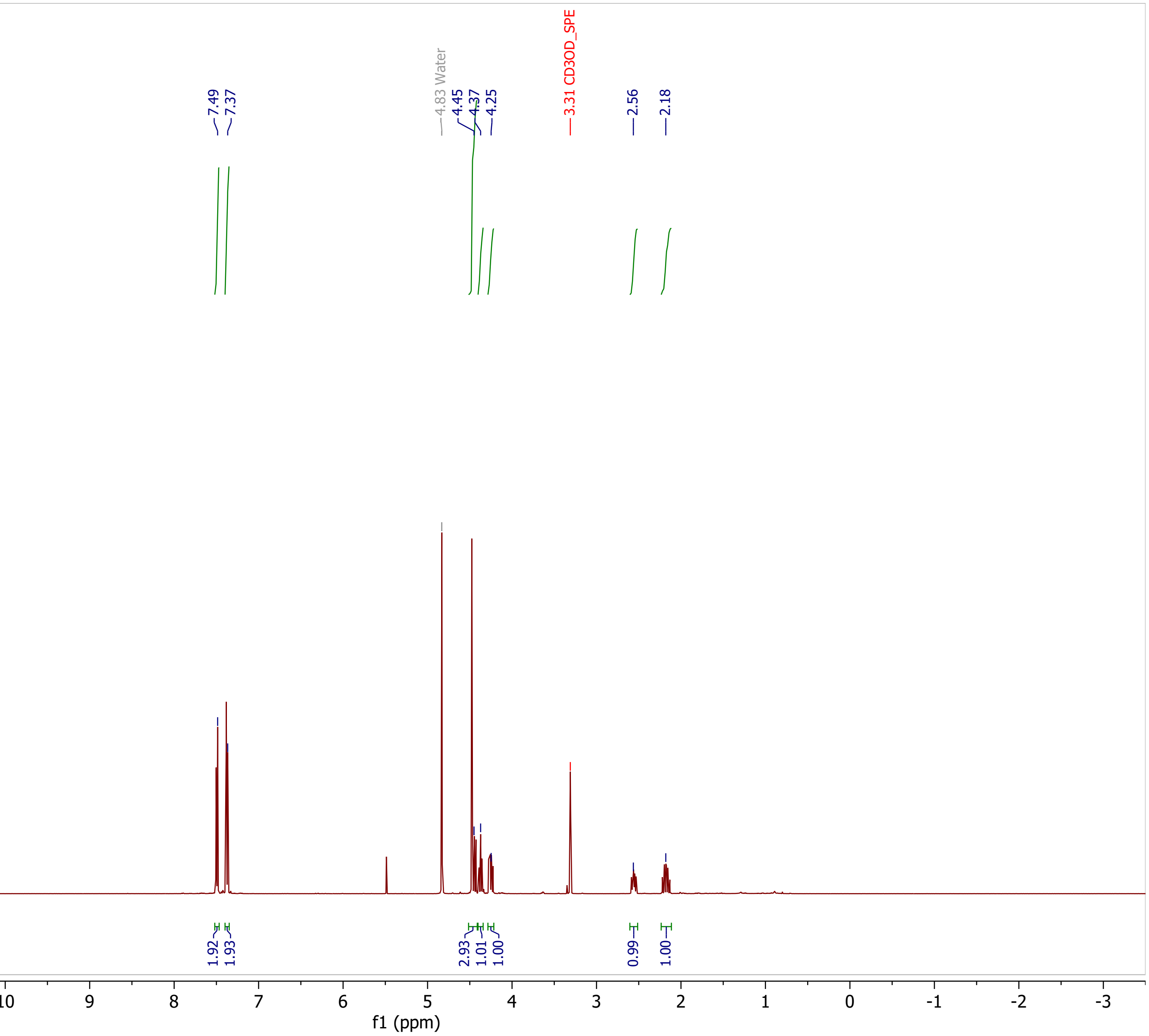
7.49
7.37
4.83 Water
4.45
4.37
4.25
3.31 CD3OD_SPE
2.56
2.18
1.92
1.93
2.93
1.01
1.00
0.99
1.00
4
13
12
11
10
9
8
7
6
5
4
3
2
1
0
-1
-2
-3
f1 (ppm)

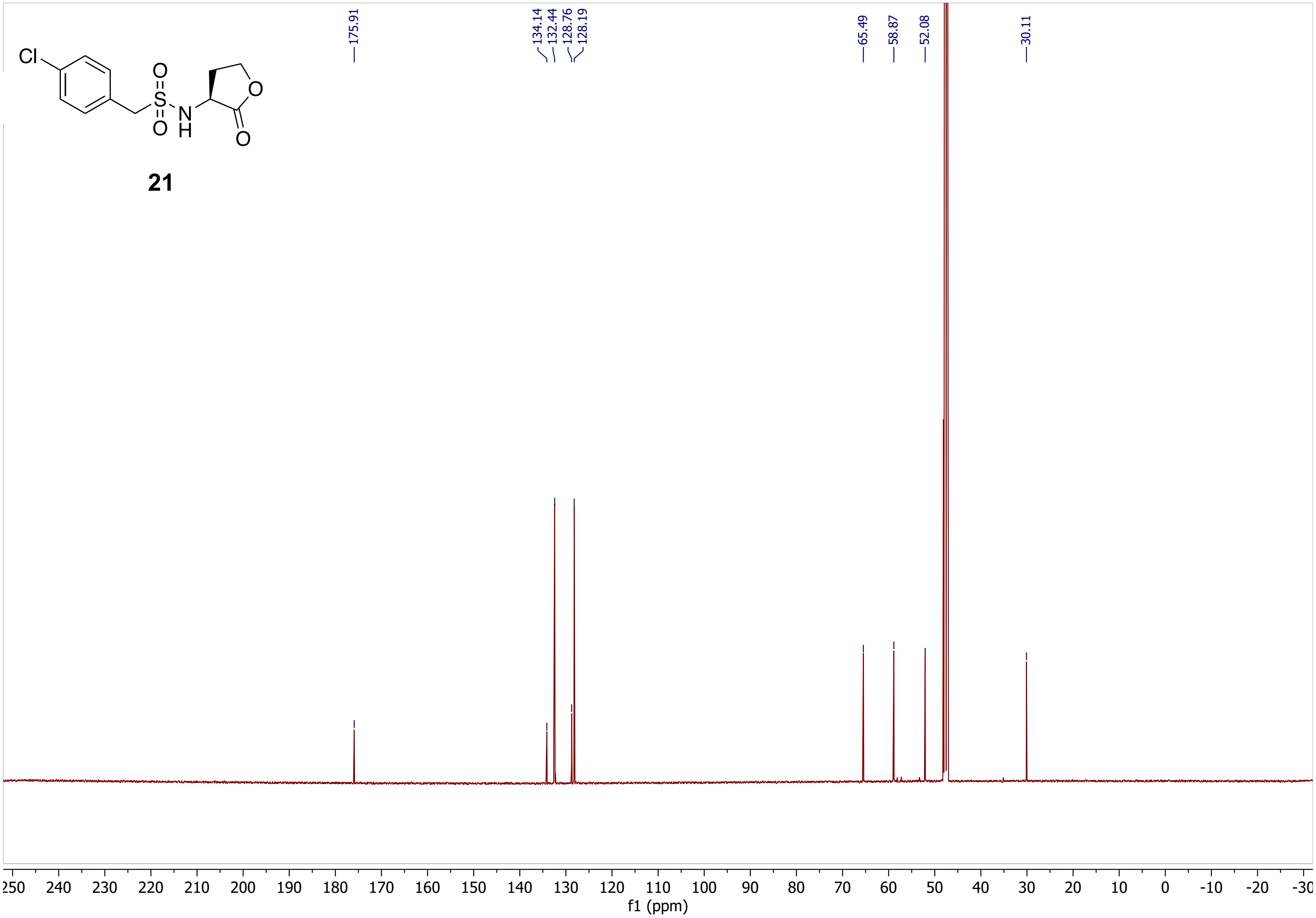
Cl
O
S
O
N
H
O
O
21
175.91
134.14
132.44
128.76
128.19
65.49
58.87
52.08
30.11
250 240 230 220 210 200 190 180 170 160 150 140 130 120 110 100 90 80 70 60 50 40 30 20 10 0 -10 -20 -30
f1 (ppm)

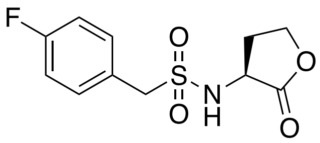
F
O
S
O
N
H
O
O

22

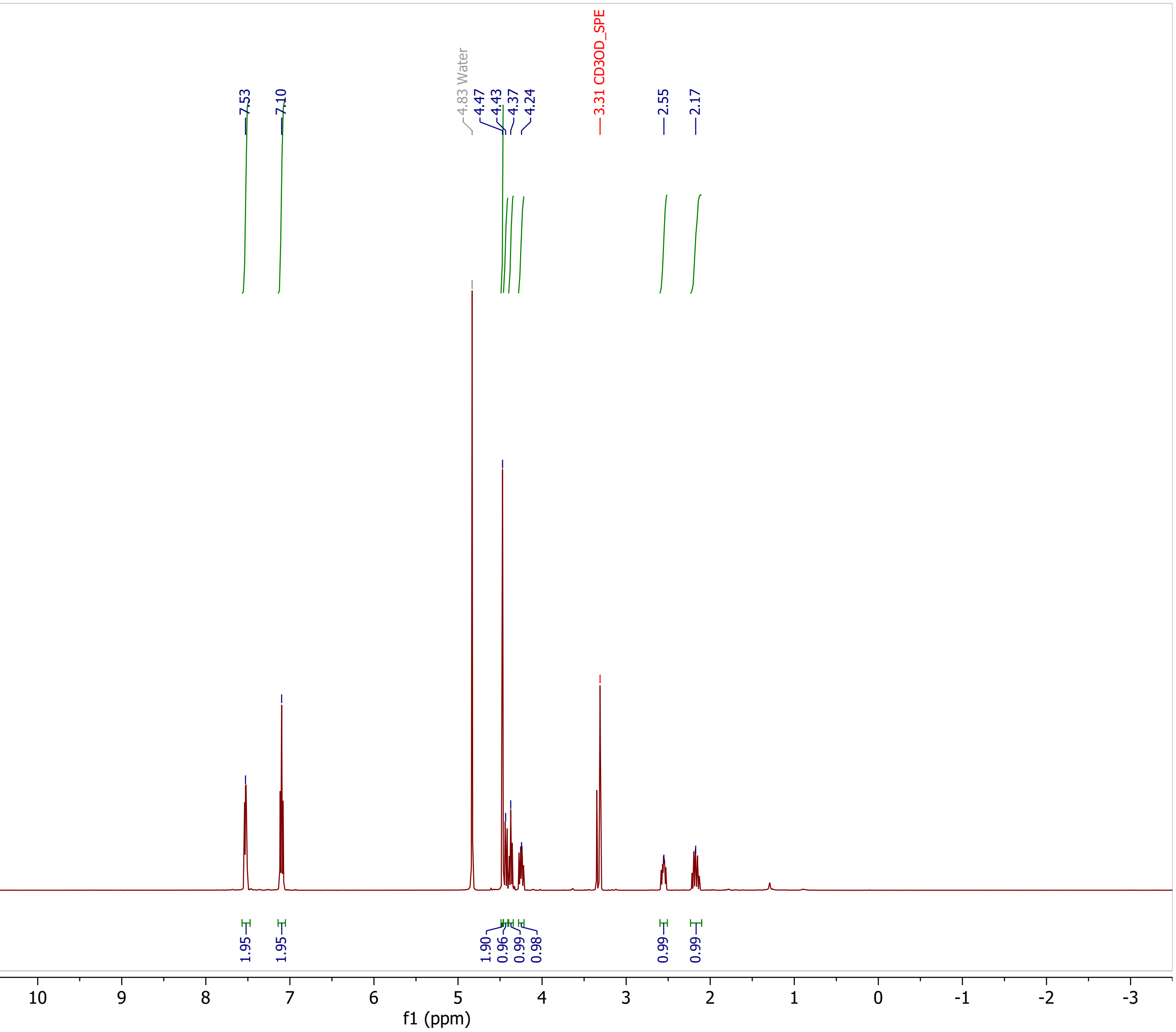
7.53
7.10
4.83 Water
4.47
4.43
4.37
4.24
3.31 CD3OD_SPE
2.55
2.17
1.95
1.95
1.90
0.96
0.99
0.98
0.99
0.99
f1 (ppm)

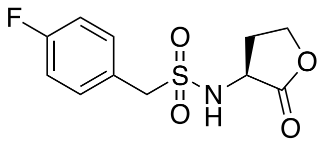
F
O
S
O
N
H
O
O

22

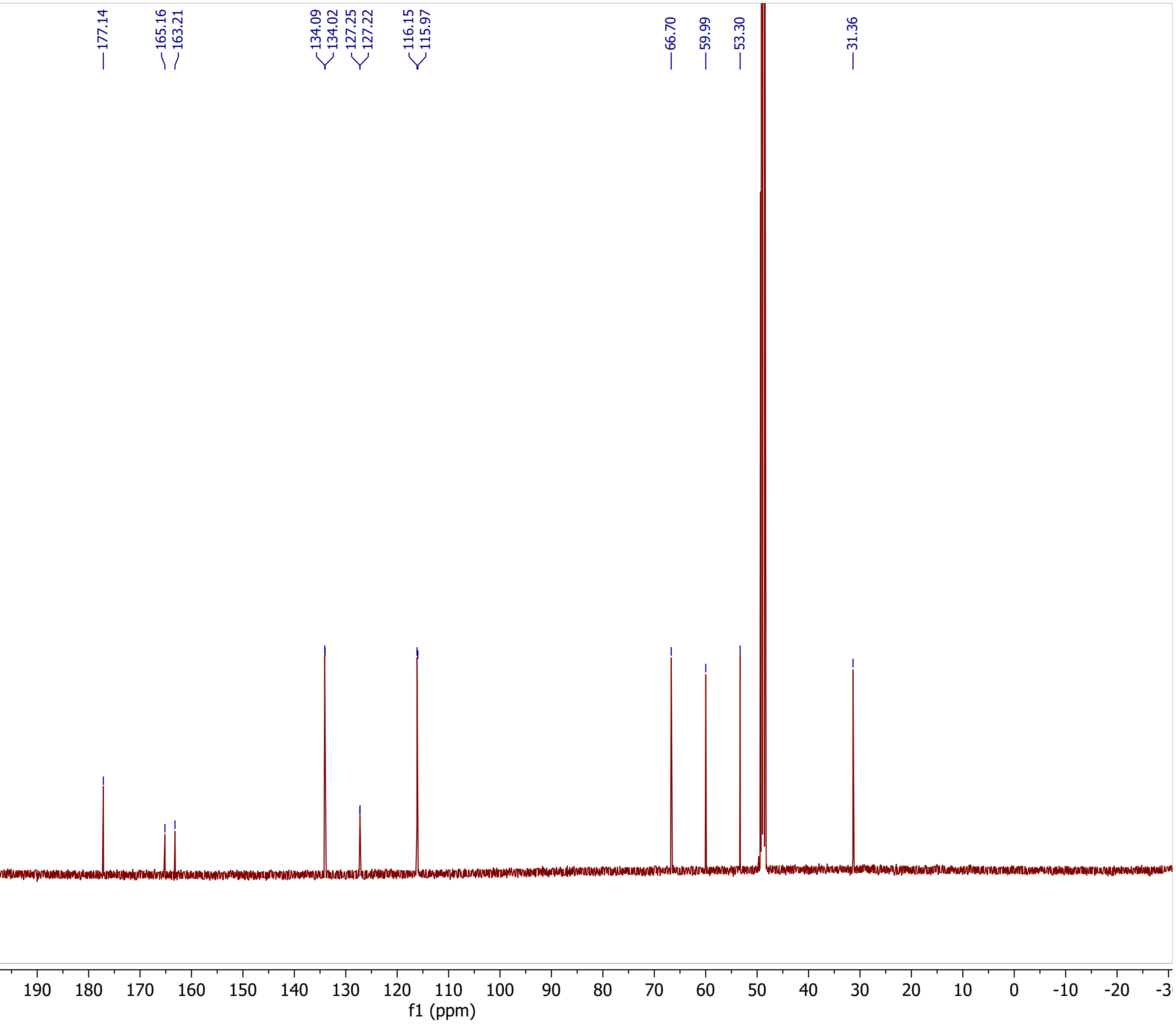
177.14
165.16
163.21
134.09
134.02
127.25
127.22
116.15
115.97
66.70
59.99
53.30
31.36
250 240 230 220 210 200 190 180 170 160 150 140 130 120 110 100 90 80 70 60 50 40 30 20 10 0 -10 -20
f1 (ppm)

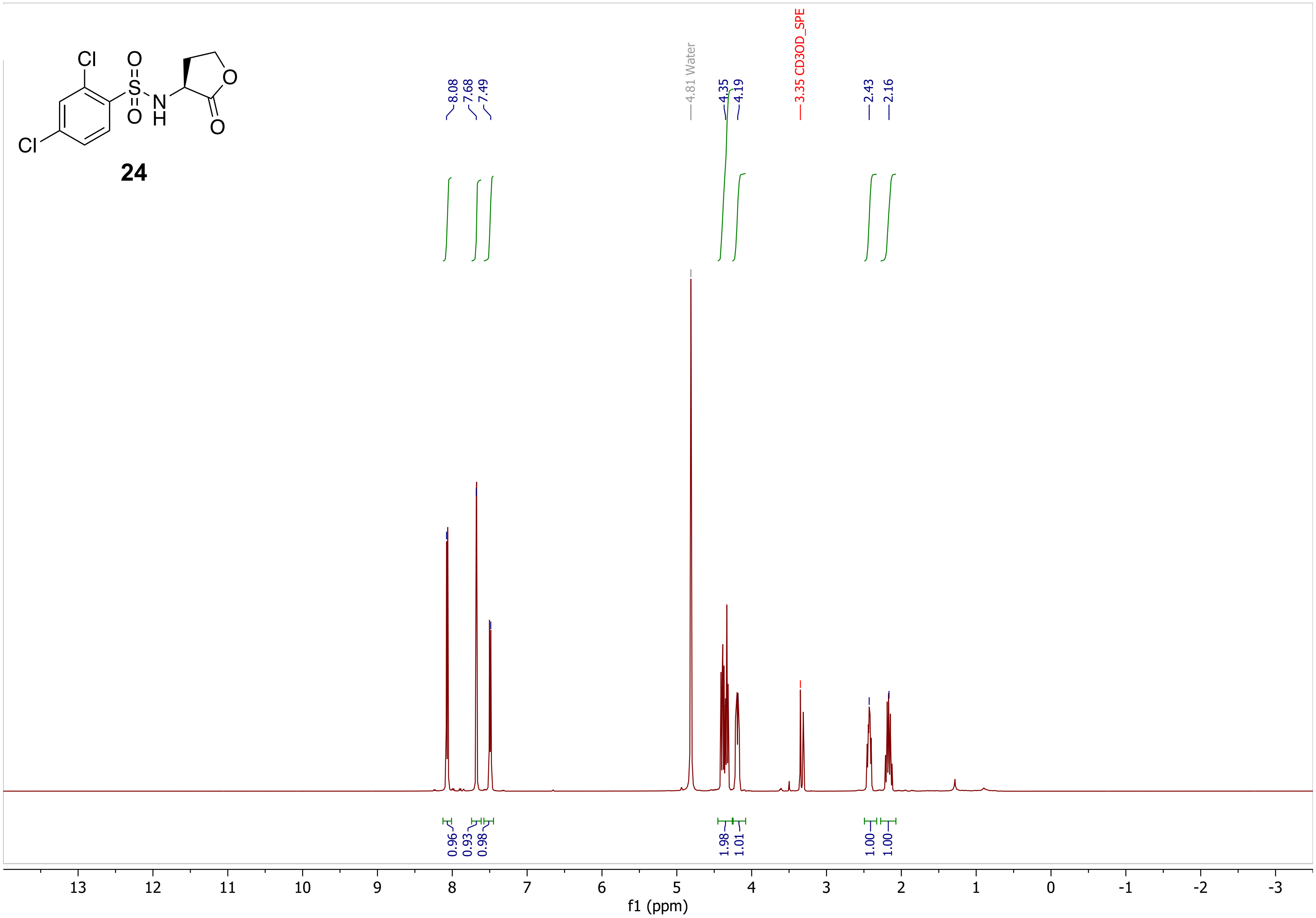

Cl
Cl
O
S
O
N
H
O
O
24
8.08
7.68
7.49
4.81 Water
4.35
4.19
3.35 CD3OD_SPE
2.43
2.16
0.96
0.93
0.98
1.98
1.01
1.00
1.00
13 12 11 10 9 8 7 6 5 4 3 2 1 0 -1 -2 -3
f1 (ppm)

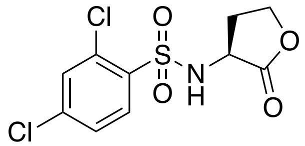

24

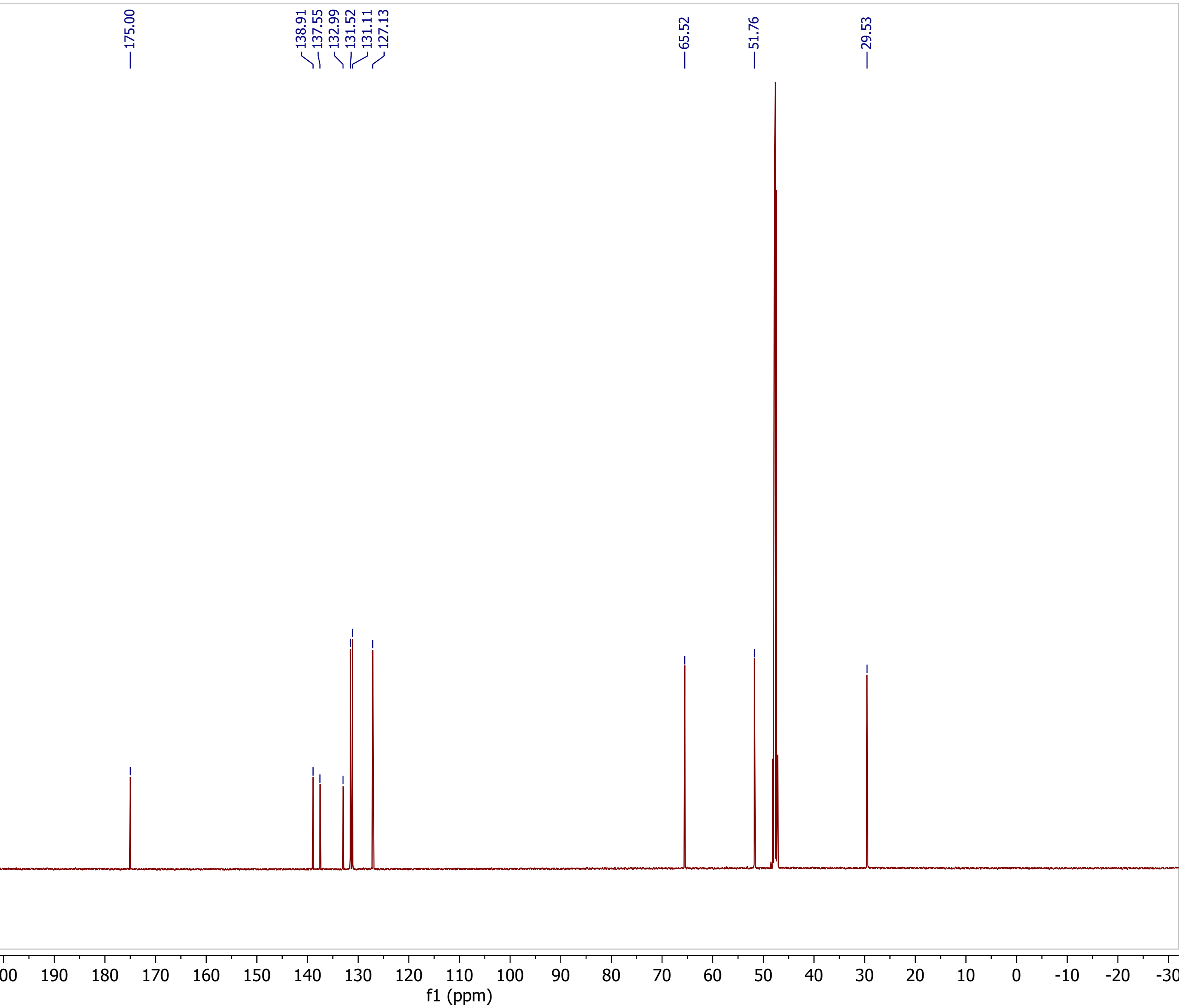
175.00
138.91
137.55
132.99
131.52
131.11
127.13
65.52
51.76
29.53
250 240 230 220 210 200 190 180 170 160 150 140 130 120 110 100 90 80 70 60 50 40 30 20 10 0 -10 -20 -30
f1 (ppm)

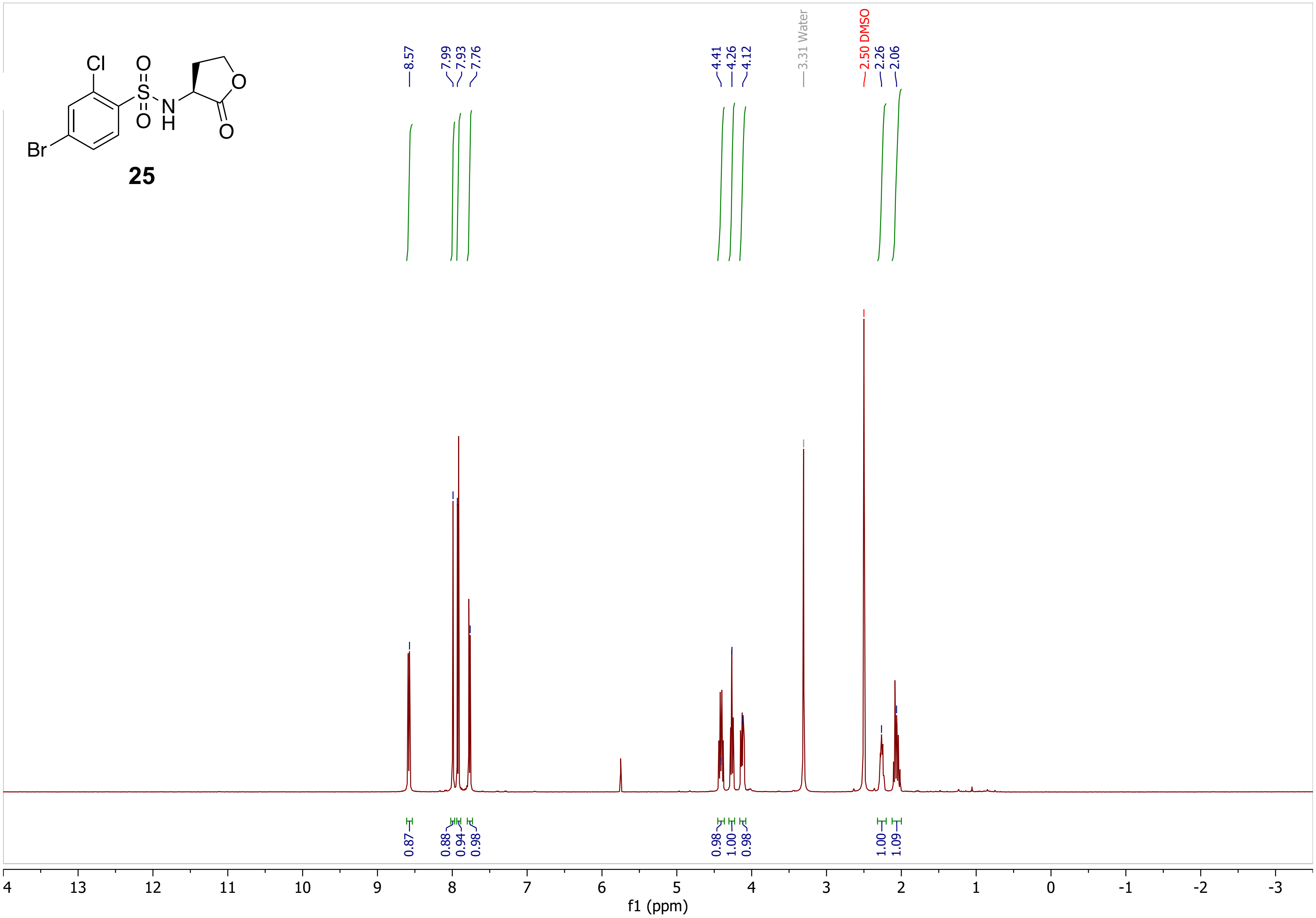
Cl
O
S
O
N
H
O
O
Br
25
8.57
7.99
7.93
7.76
4.41
4.26
4.12
3.31 Water
2.50 DMSO
2.26
2.06
0.87
0.88
0.94
0.98
0.98
1.00
0.98
1.00
1.09
f1 (ppm)

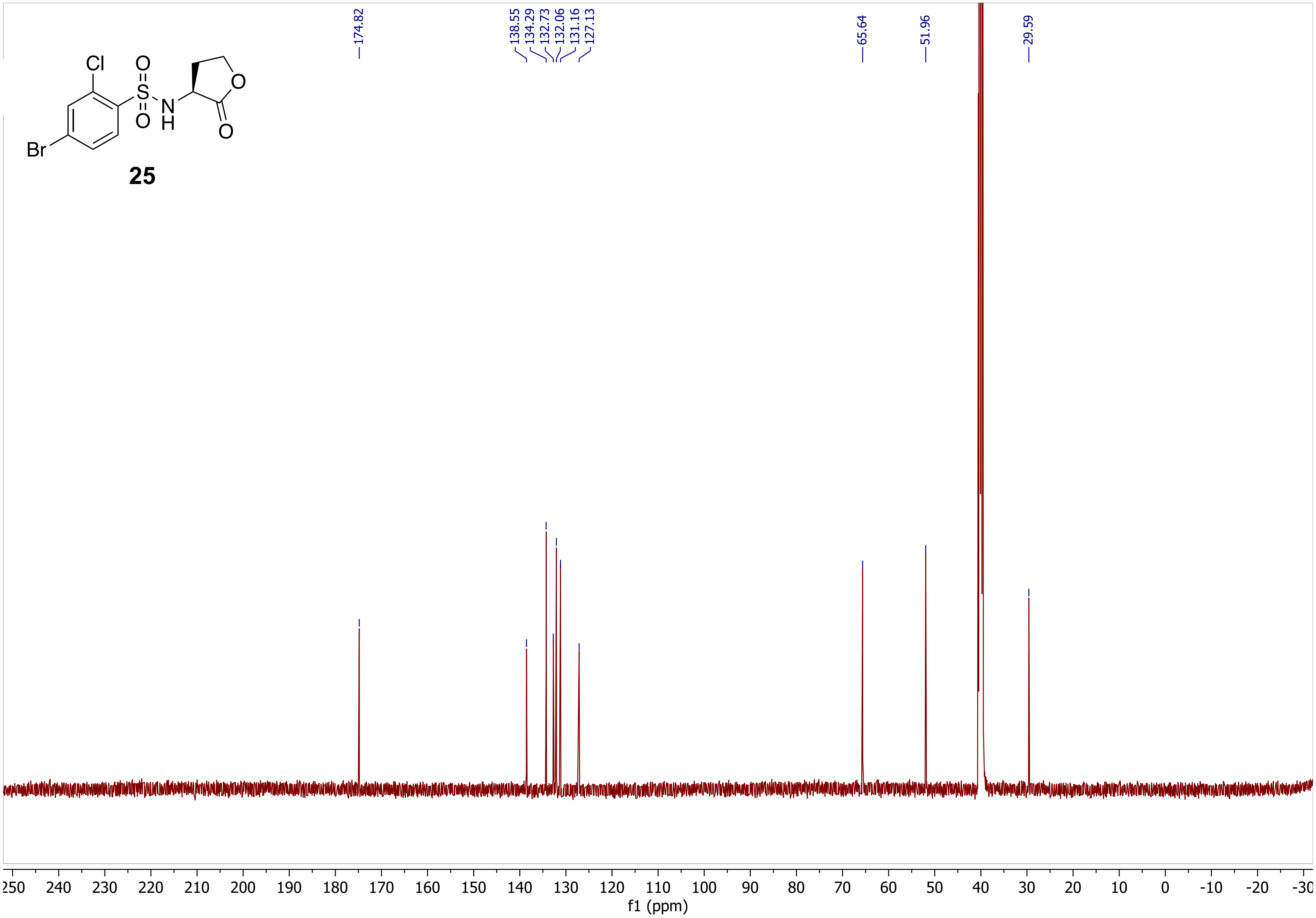

174.82
138.55
134.29
132.73
132.06
131.16
127.13
65.64
51.96
29.59
Cl
O
S
O
N
H
O
O
Br
25
250 240 230 220 210 200 190 180 170 160 150 140 130 120 110 100 90 80 70 60 50 40 30 20 10 0 -10 -20 -30
f1 (ppm)

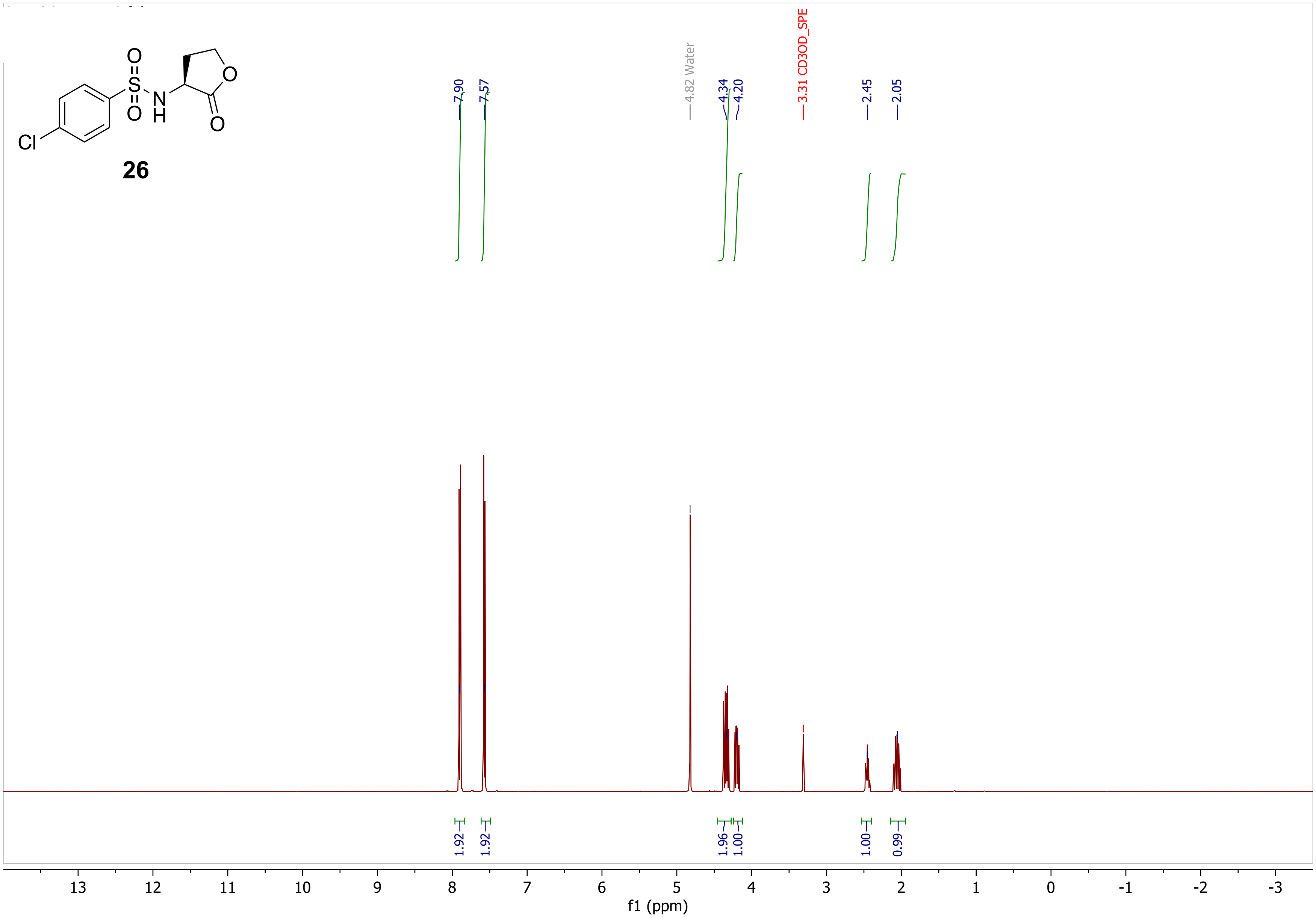
Cl
O
S
O
N
H
O
O
26
7.90
7.57
4.82 Water
4.34
4.20
3.31 CD3OD_SPE
2.45
2.05
1.92
1.92
1.96
1.00
1.00
0.99
13
12
11
10
9
8
7
6
5
4
3
2
1
0
-1
-2
-3
f1 (ppm)

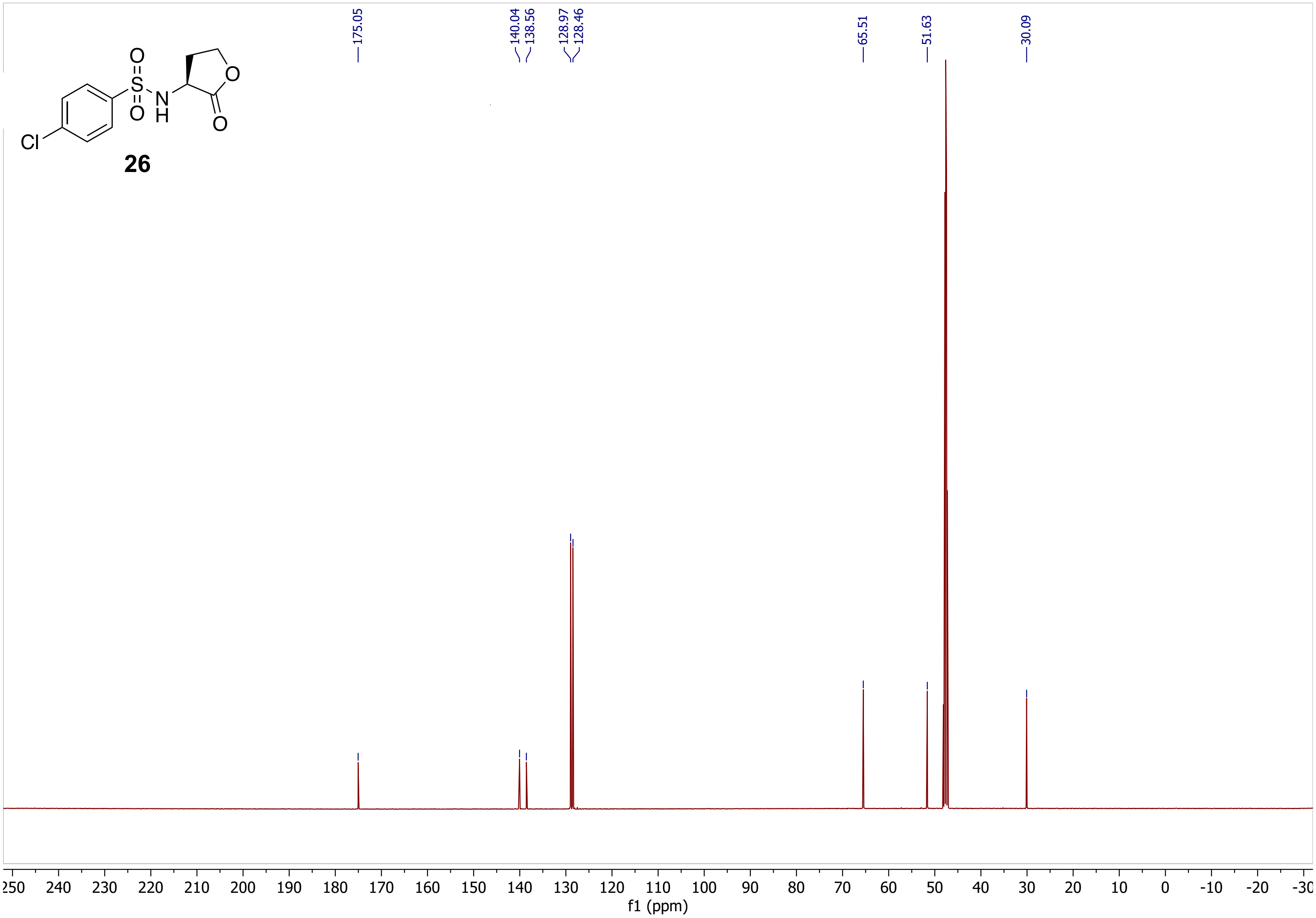
Cl
O
S
O
N
H
O
O
26
175.05
140.04
138.56
128.97
128.46
65.51
51.63
30.09
250 240 230 220 210 200 190 180 170 160 150 140 130 120 110 100 90 80 70 60 50 40 30 20 10 0 -10 -20 -30
f1 (ppm)

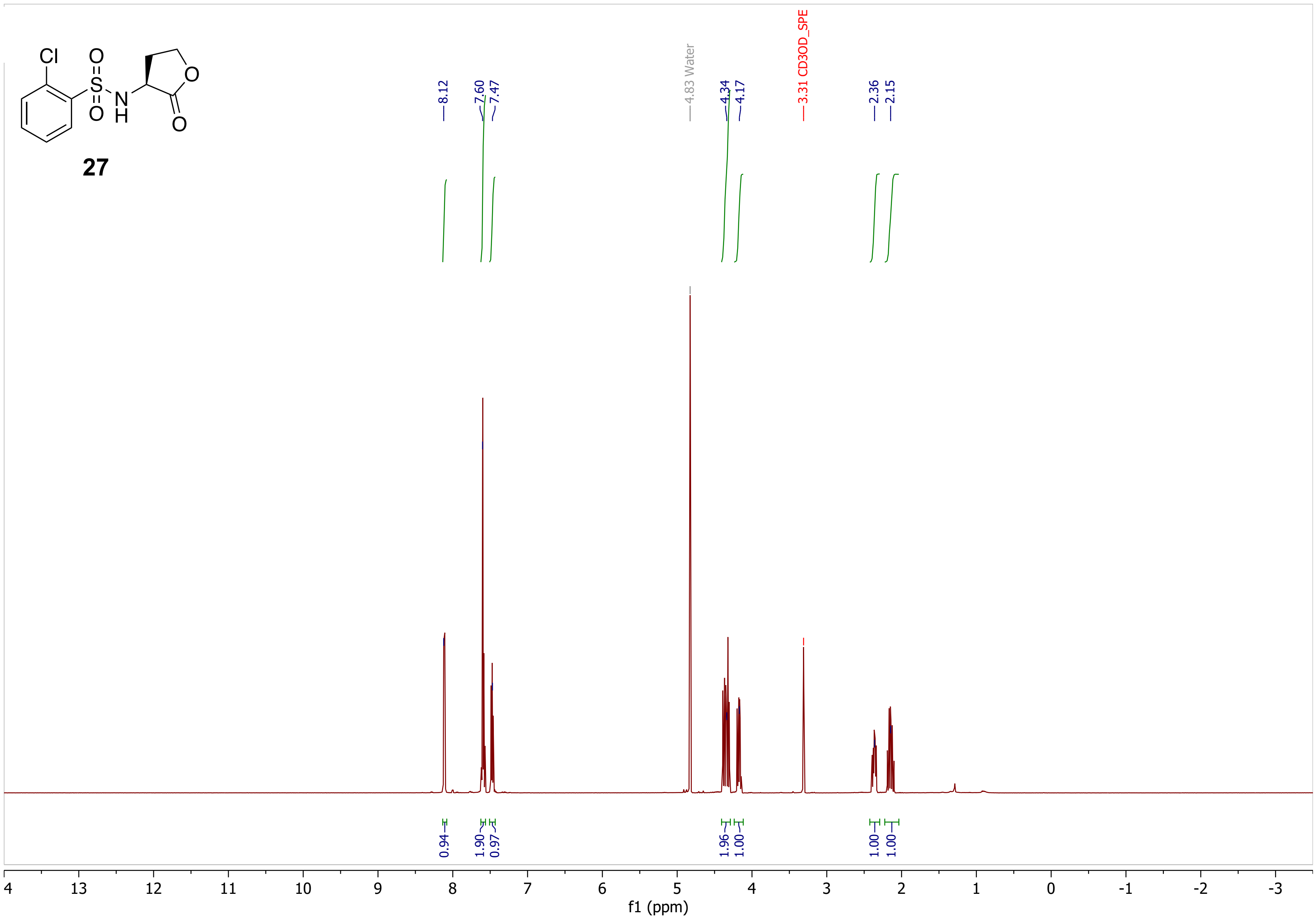
27
8.12
7.60
7.47
4.83 Water
4.34
4.17
3.31 CD3OD_SPE
2.36
2.15
0.94
1.90
0.97
1.96
1.00
1.00
1.00
f1 (ppm)

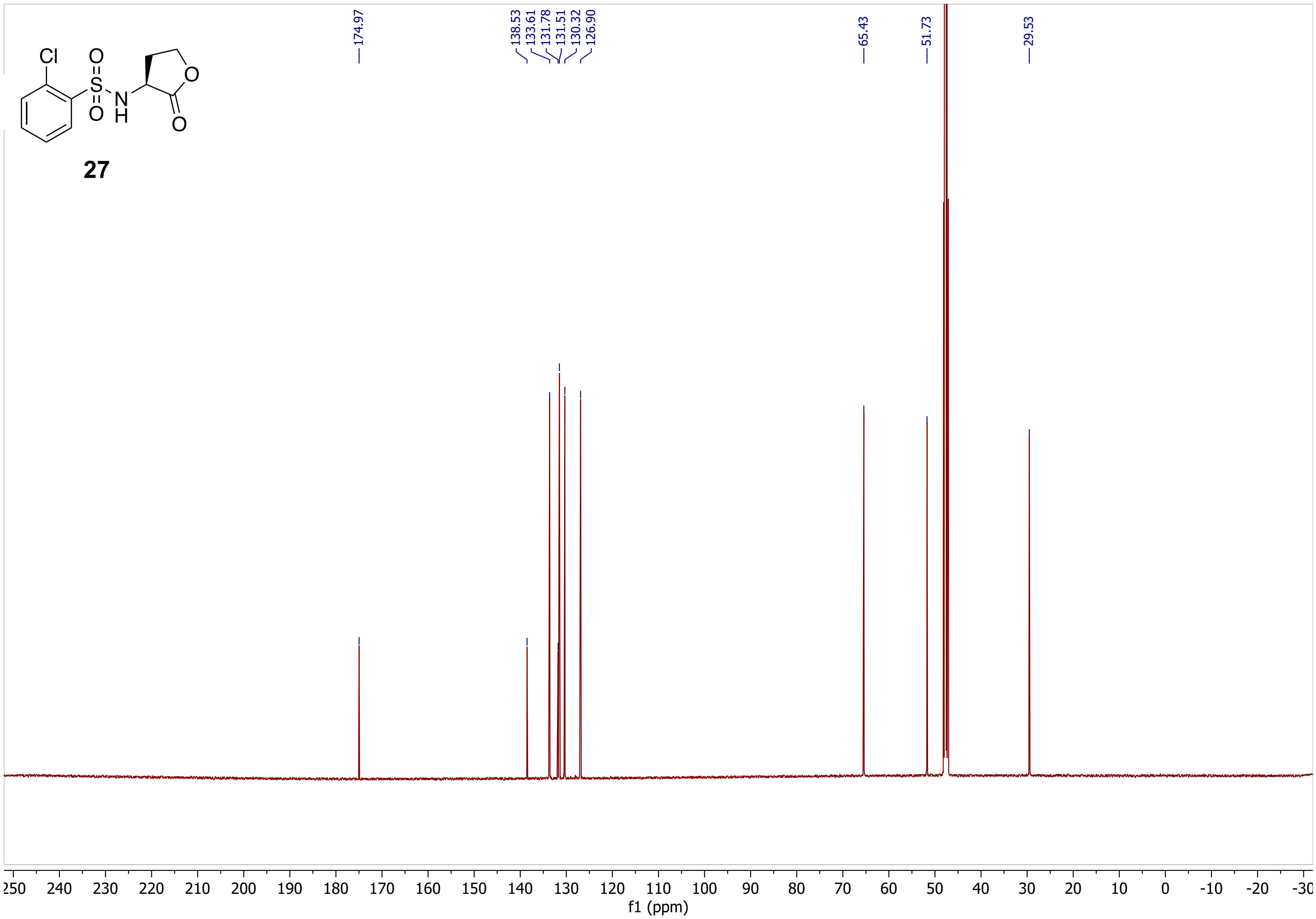

Cl
O
S
O
N
H
O
O
27
174.97
138.53
133.61
131.78
131.51
130.32
126.90
65.43
51.73
29.53
250
240
230
220
210
200
190
180
170
160
150
140
130
120
110
100
90
80
70
60
50
40
30
20
10
0
-10
-20
-30
f1 (ppm)

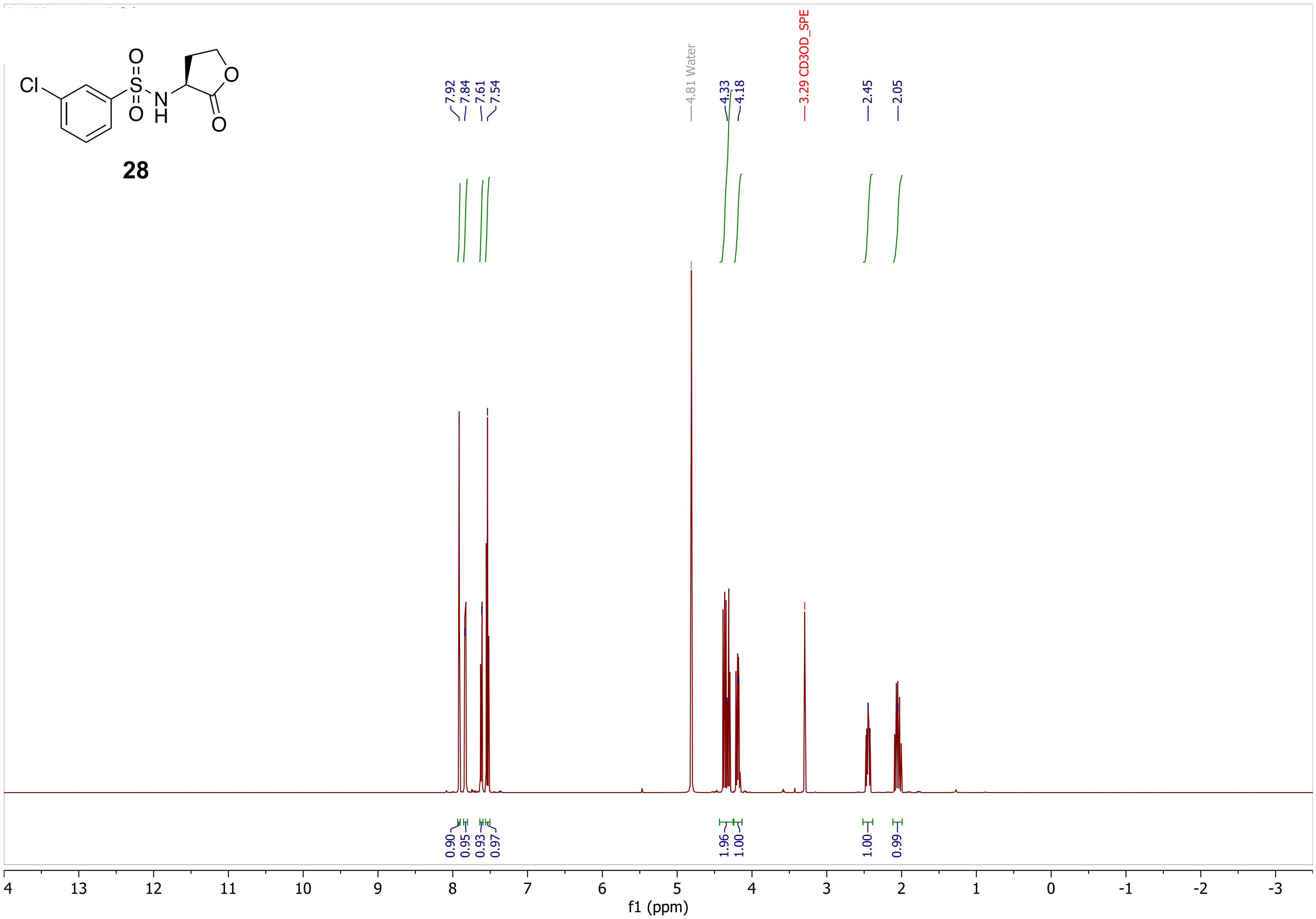
28
7.92
7.84
7.61
7.54
4.81 Water
4.33
4.18
3.29 CD3OD_SPE
2.45
2.05
0.90
0.95
0.93
0.97
1.96
1.00
1.00
0.99
f1 (ppm)

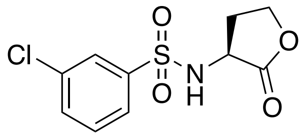

28

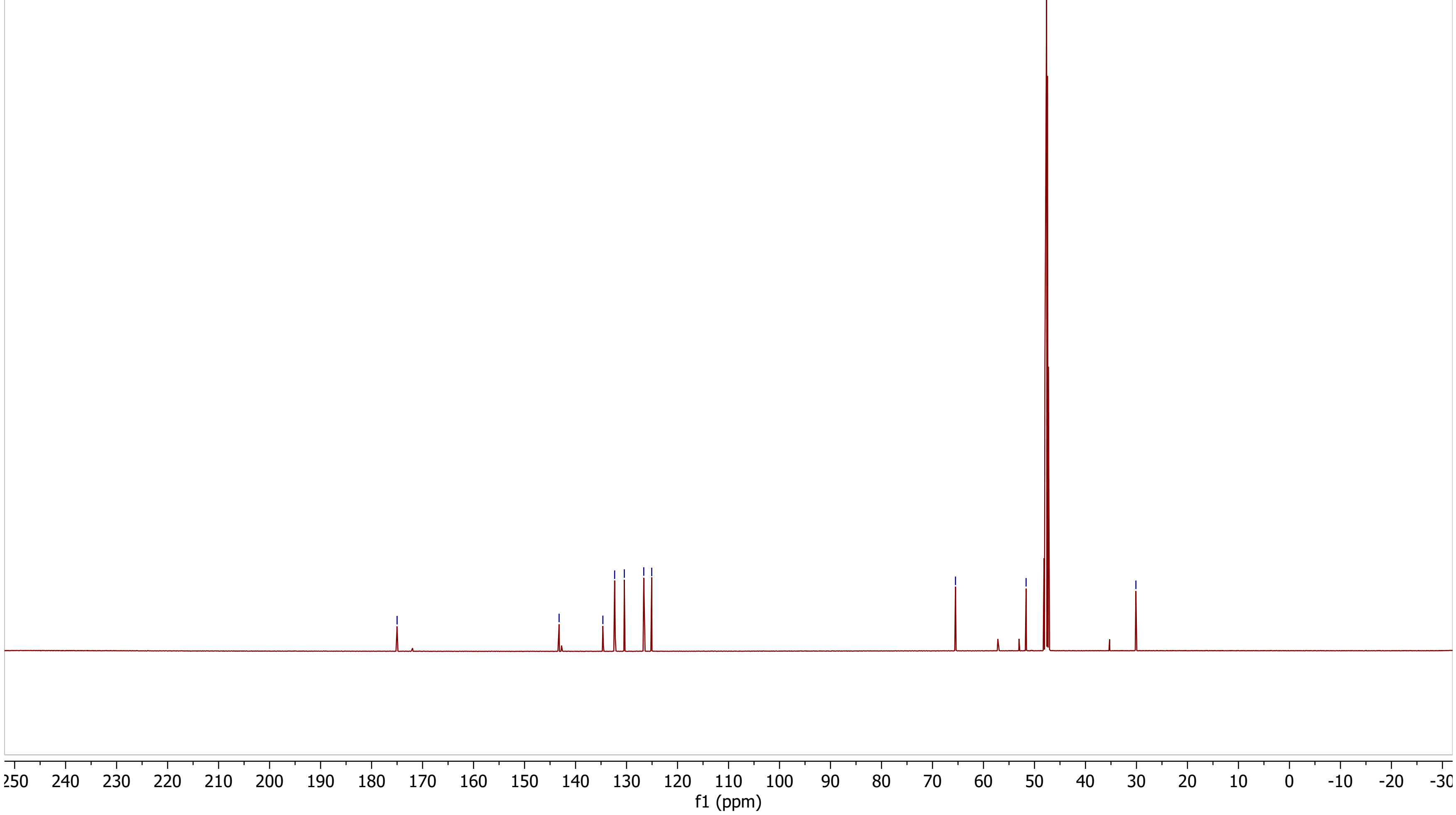
175.00
143.23
134.64
132.34
130.43
126.62
125.06
65.50
51.65
30.11
250 240 230 220 210 200 190 180 170 160 150 140 130 120 110 100 90 80 70 60 50 40 30 20 10 0 -10 -20 -30
f1 (ppm)

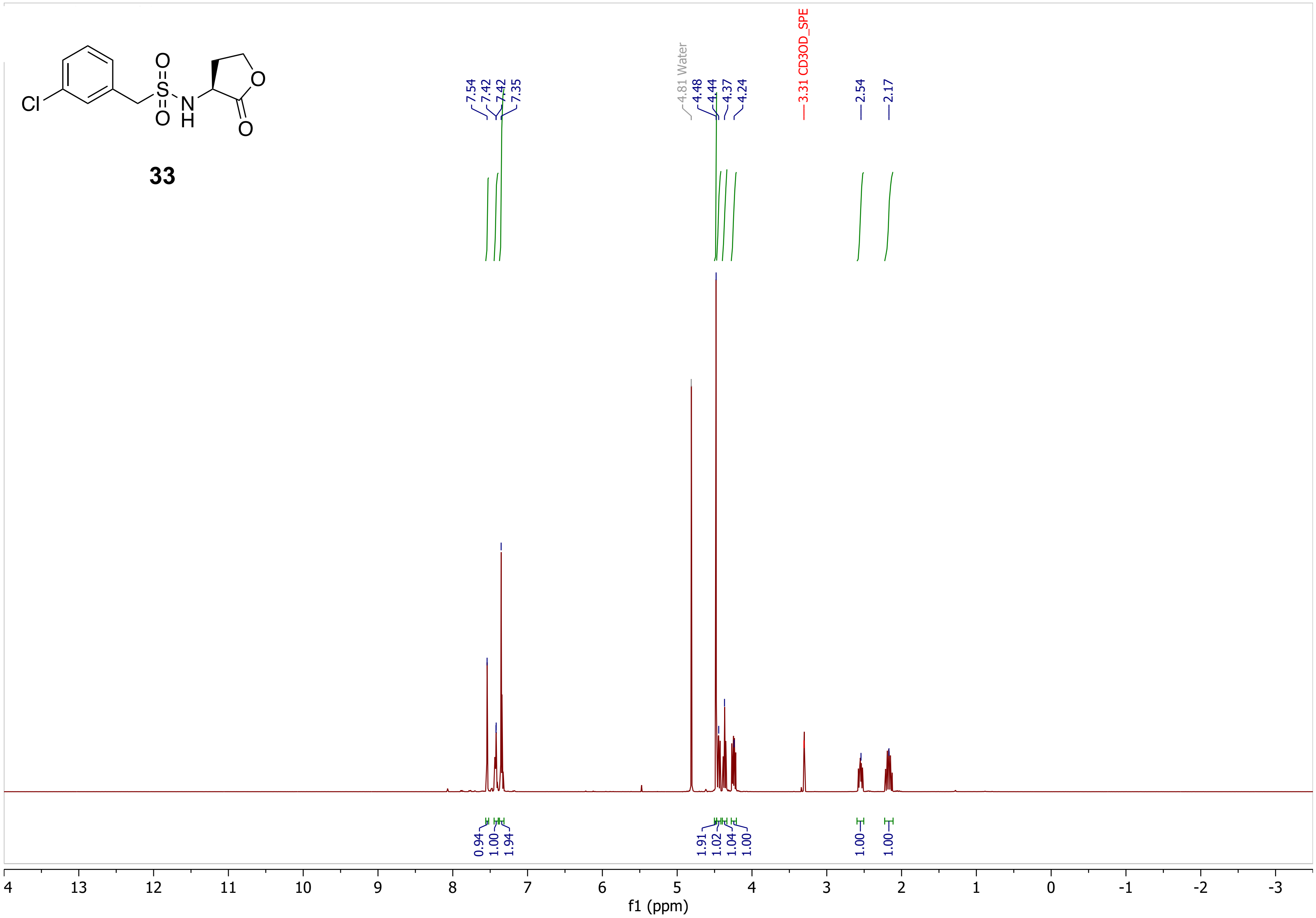

33
7.54
7.42
7.42
7.35
4.81 Water
4.48
4.44
4.37
4.24
3.31 CD3OD_SPE
2.54
2.17
0.94
1.00
1.94
1.91
1.02
1.04
1.00
1.00
1.00
f1 (ppm)

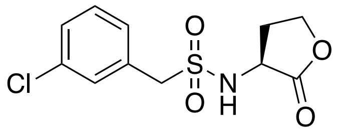

33

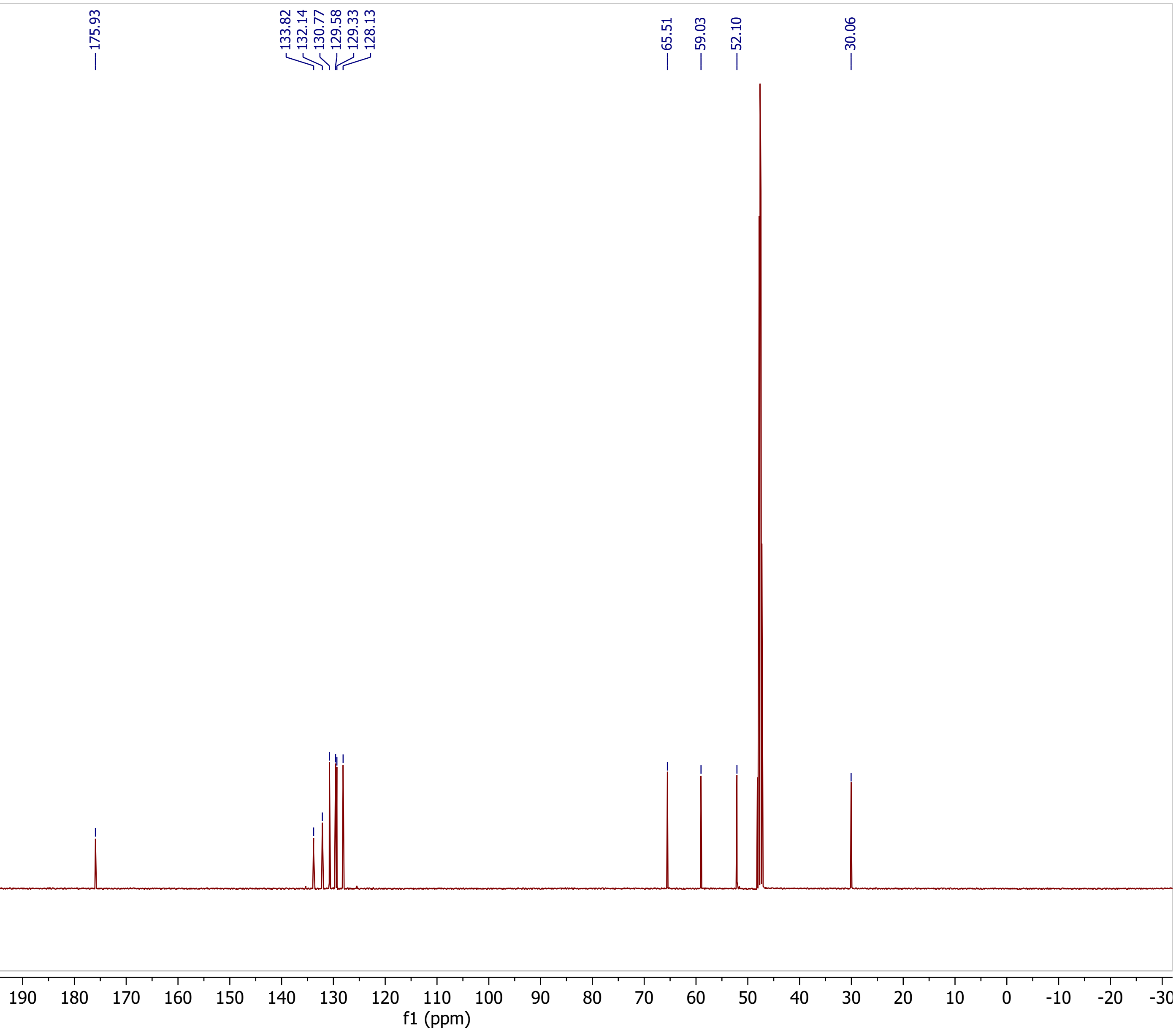
175.93
133.82
132.14
130.77
129.58
129.33
128.13
65.51
59.03
52.10
30.06
250 240 230 220 210 200 190 180 170 160 150 140 130 120 110 100 90 80 70 60 50 40 30 20 10 0 -10 -20 -30
f1 (ppm)

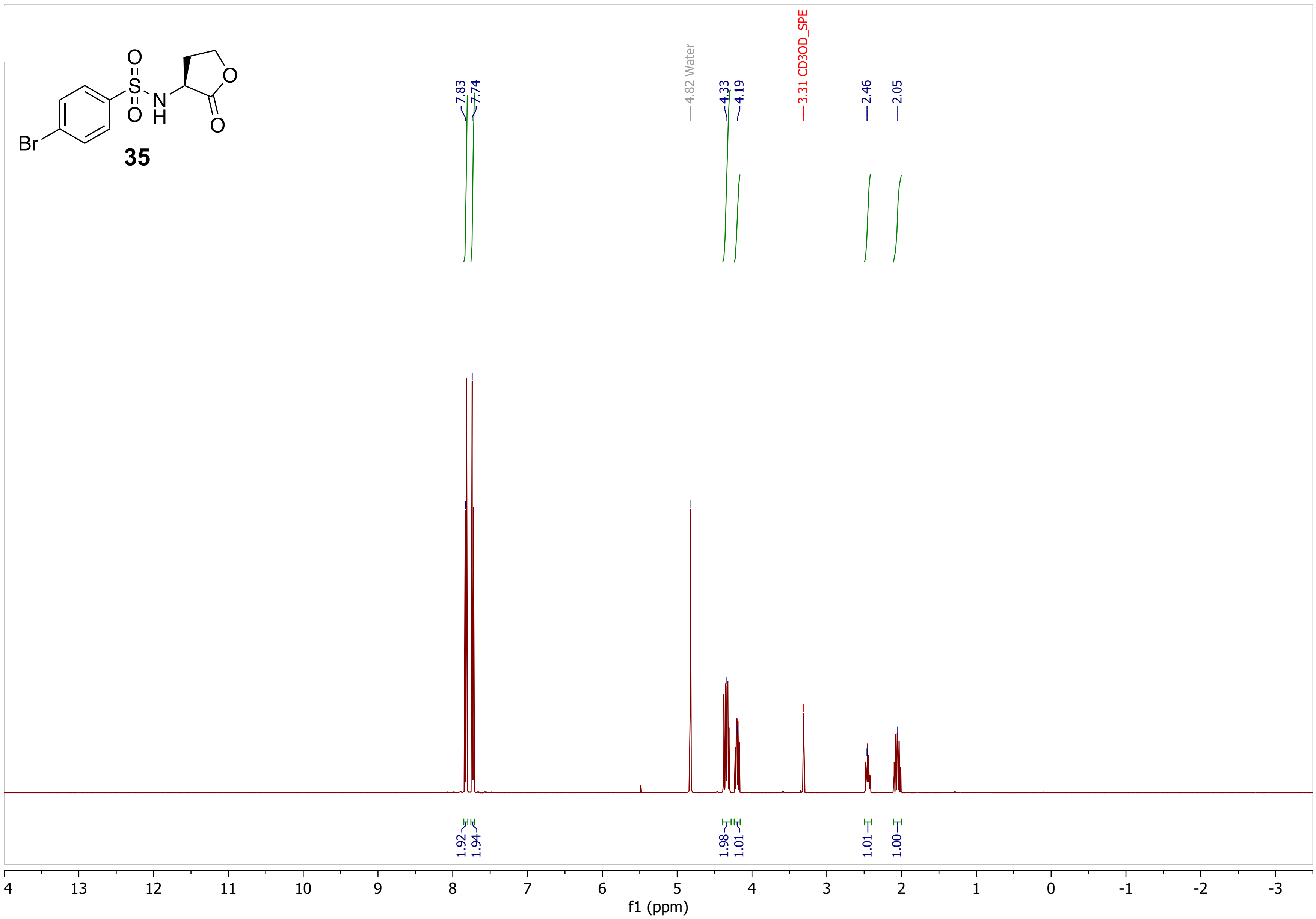

Br
O
S
O
N
H
O
O
35
7.83
7.74
4.82 Water
4.33
4.19
3.31 CD3OD_SPE
2.46
2.05
1.92
1.94
1.98
1.01
1.01
1.00
f1 (ppm)

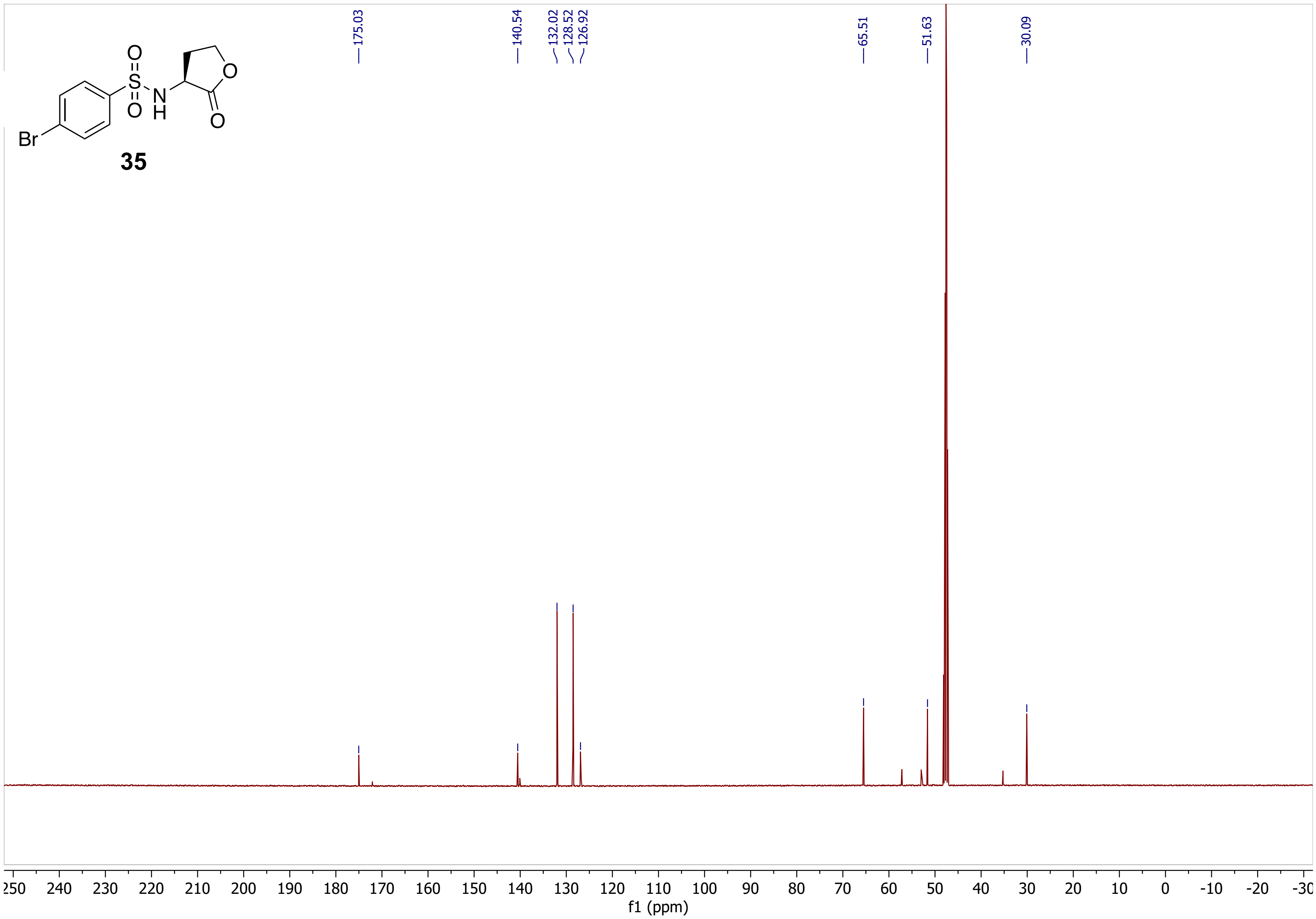

35
175.03
140.54
132.02
128.52
126.92
65.51
51.63
30.09
f1 (ppm)

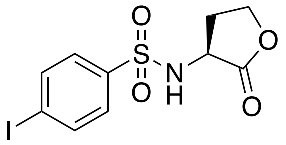

36

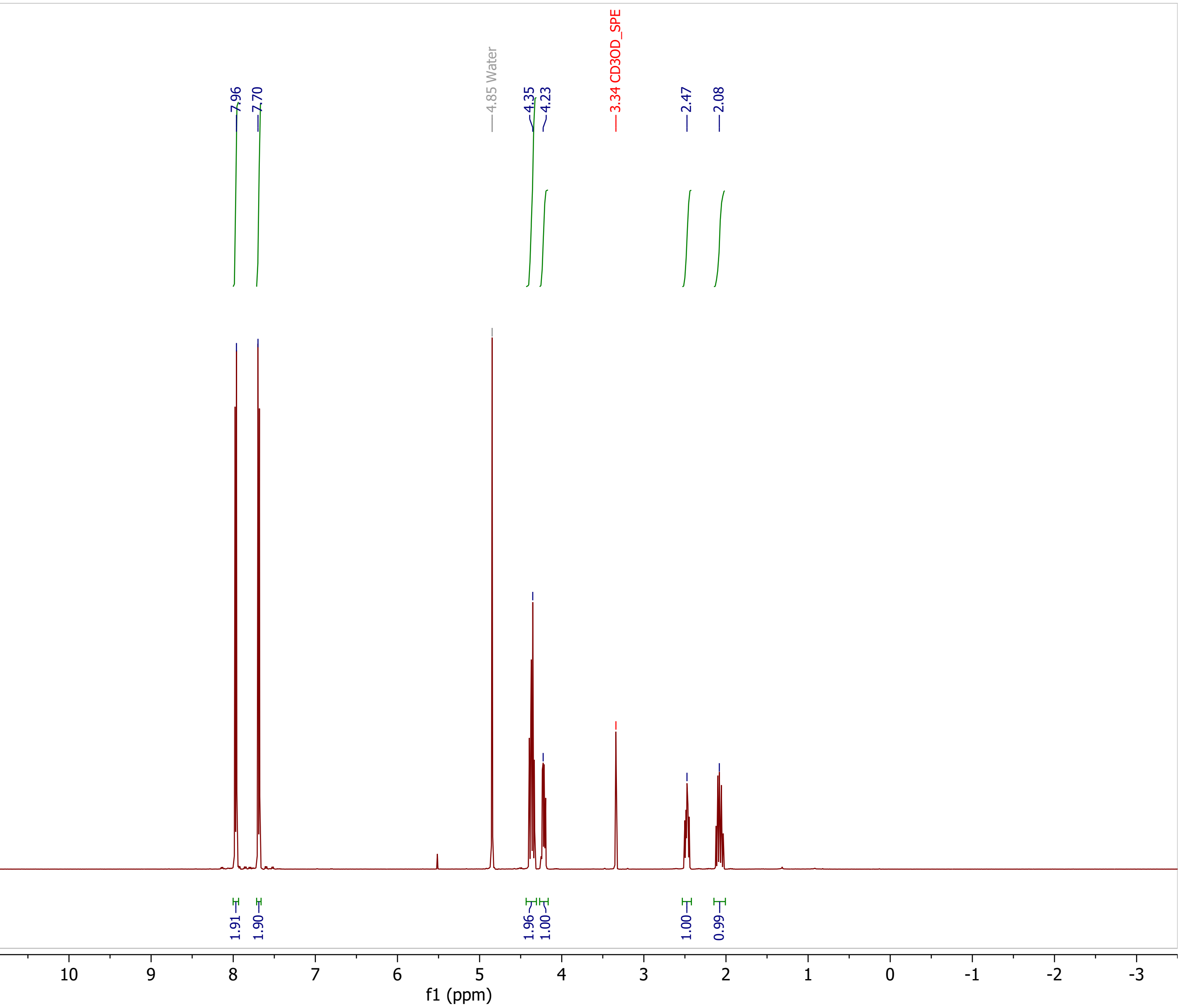
7.96
7.70
4.85 Water
4.35
4.23
3.34 CD3OD_SPE
2.47
2.08
1.91
1.90
1.96
1.00
1.00
0.99
4 13 12 11 10 9 8 7 6 5 4 3 2 1 0 -1 -2 -3
f1 (ppm)

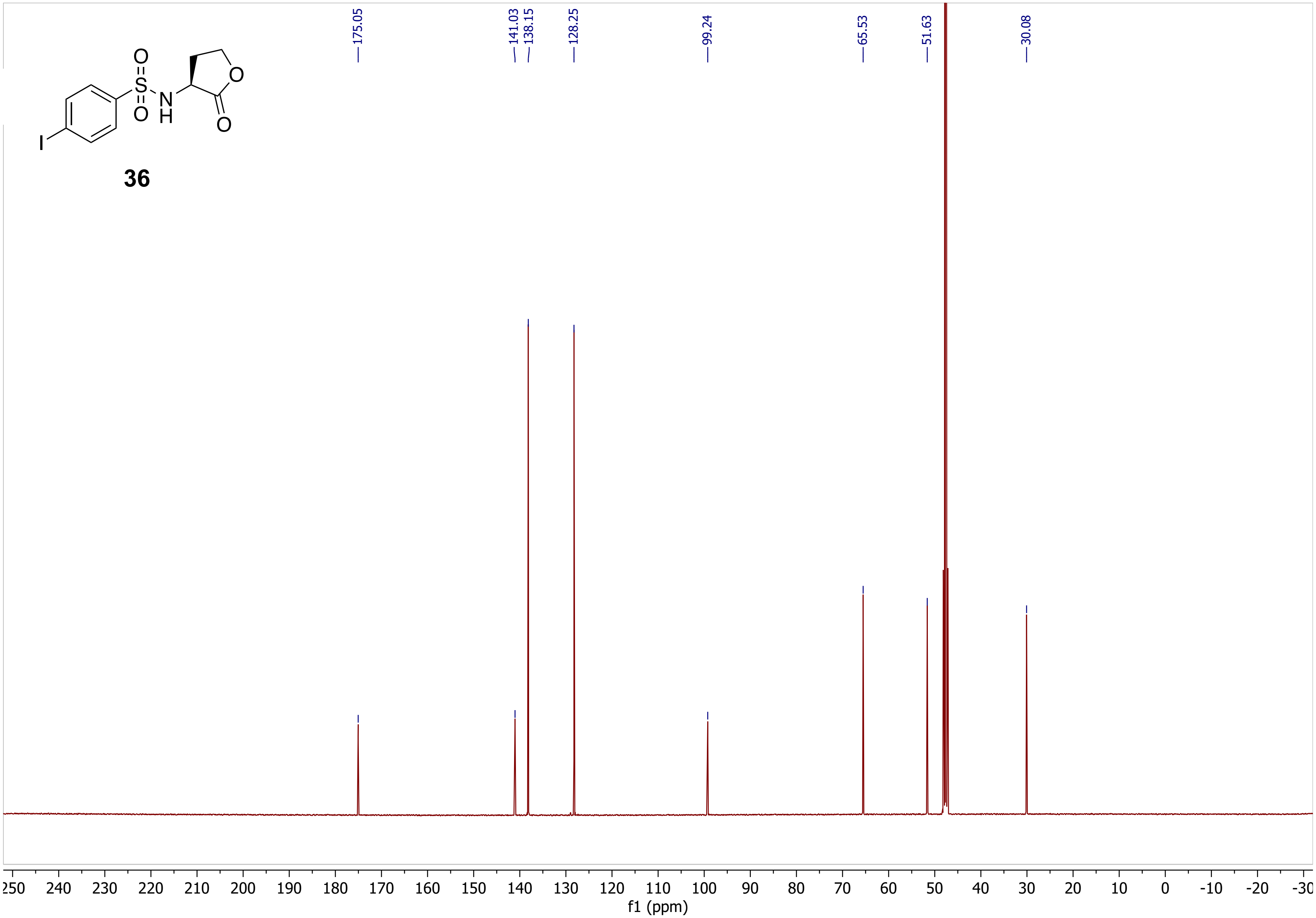
175.05
141.03
138.15
128.25
99.24
65.53
51.63
30.08
250 240 230 220 210 200 190 180 170 160 150 140 130 120 110 100 90 80 70 60 50 40 30 20 10 0 -10 -20 -30
f1 (ppm)

36

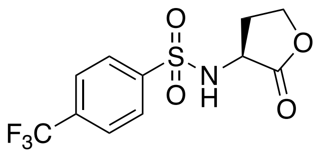

37

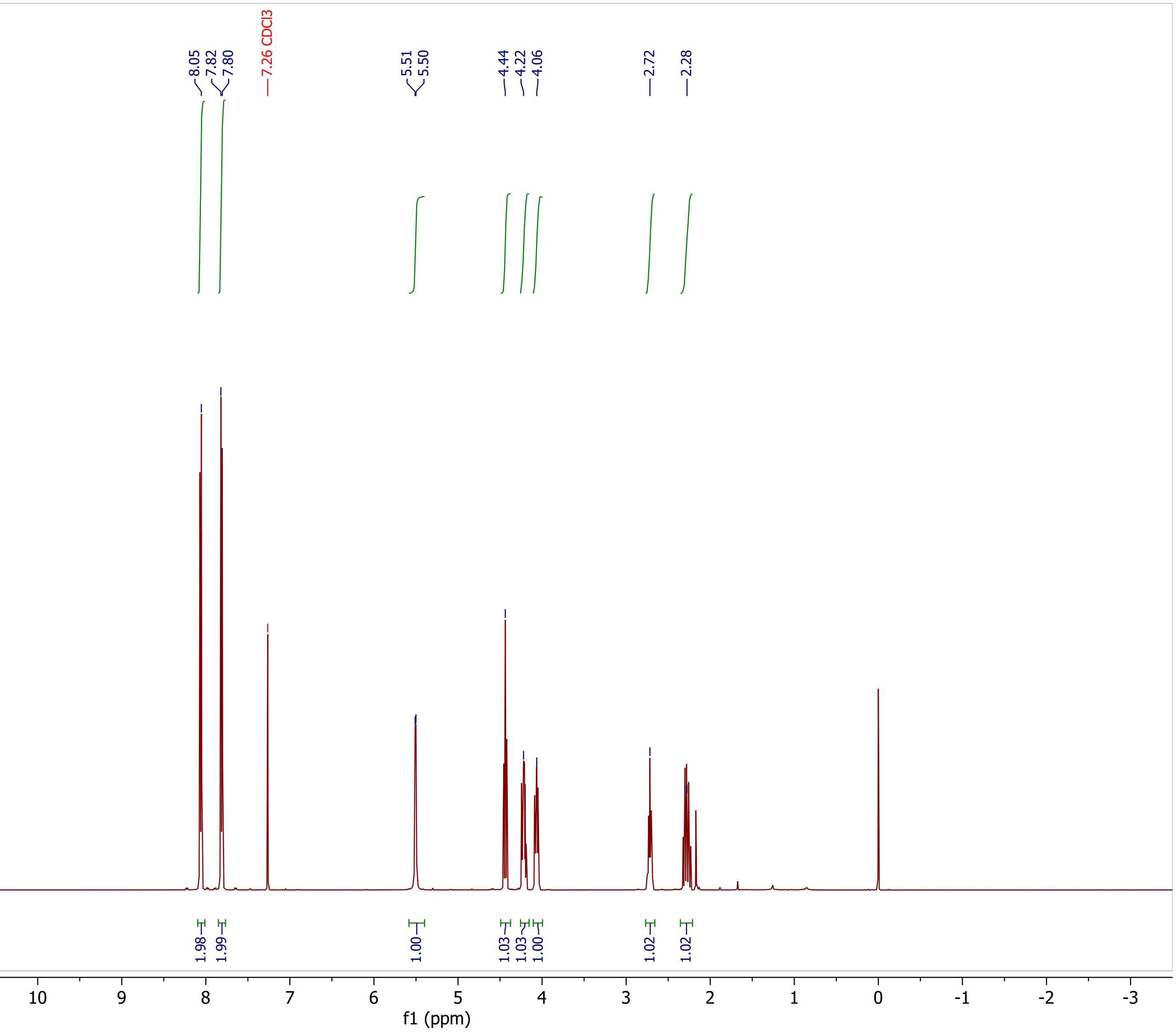

8.05
7.82
7.80
7.26 CDCl3
5.51
5.50
4.44
4.22
4.06
2.72
2.28
1.98
1.99
1.00
1.03
1.03
1.00
1.02
1.02
13
12
11
10
9
8
7
6
5
4
3
2
1
0
-1
-2
-3
f1 (ppm)

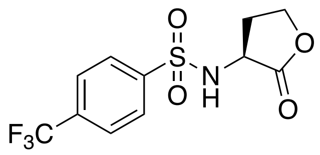

37

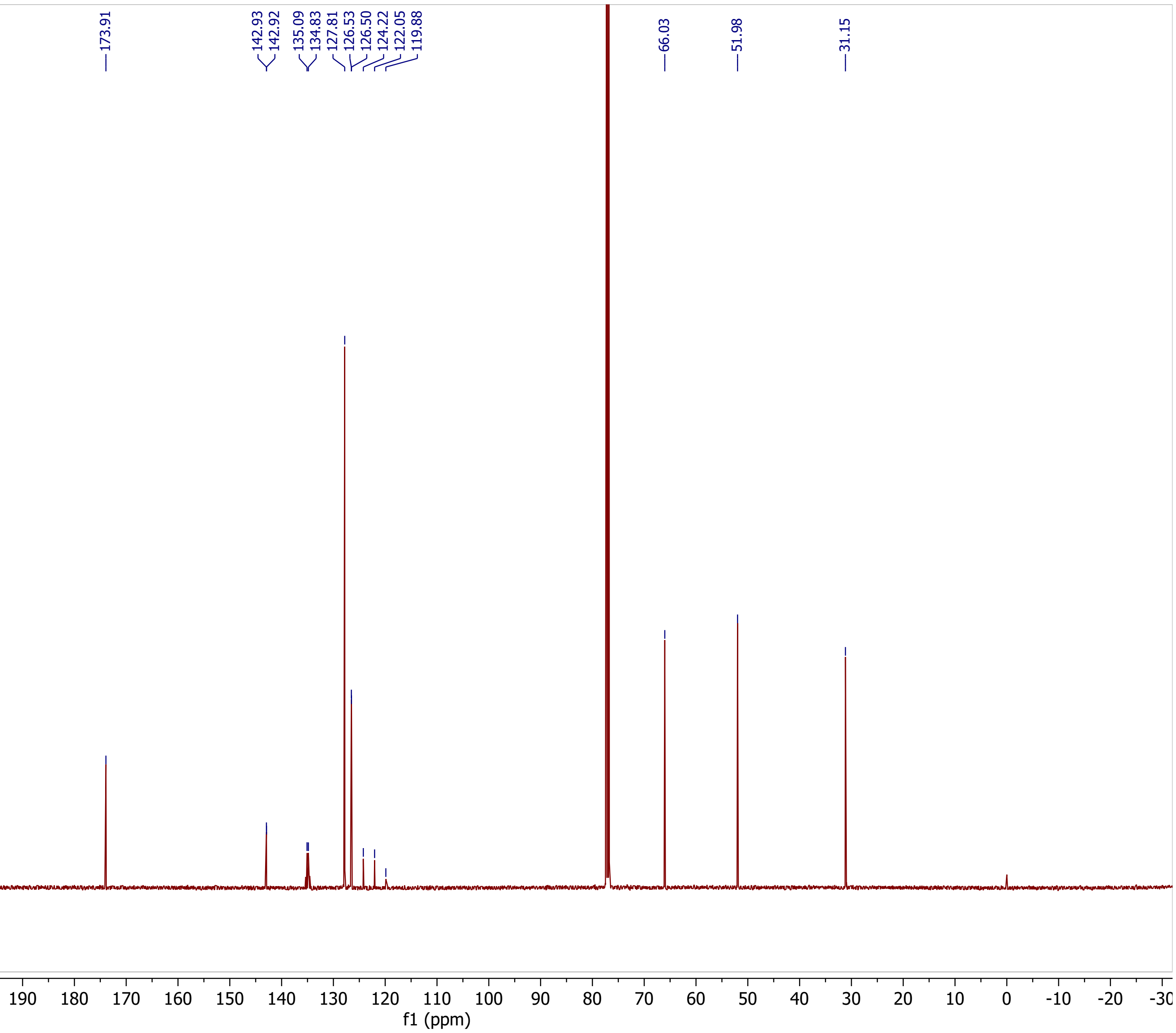
173.91
142.93
142.92
135.09
134.83
127.81
126.53
126.50
124.22
122.05
119.88
66.03
51.98
31.15
f1 (ppm)

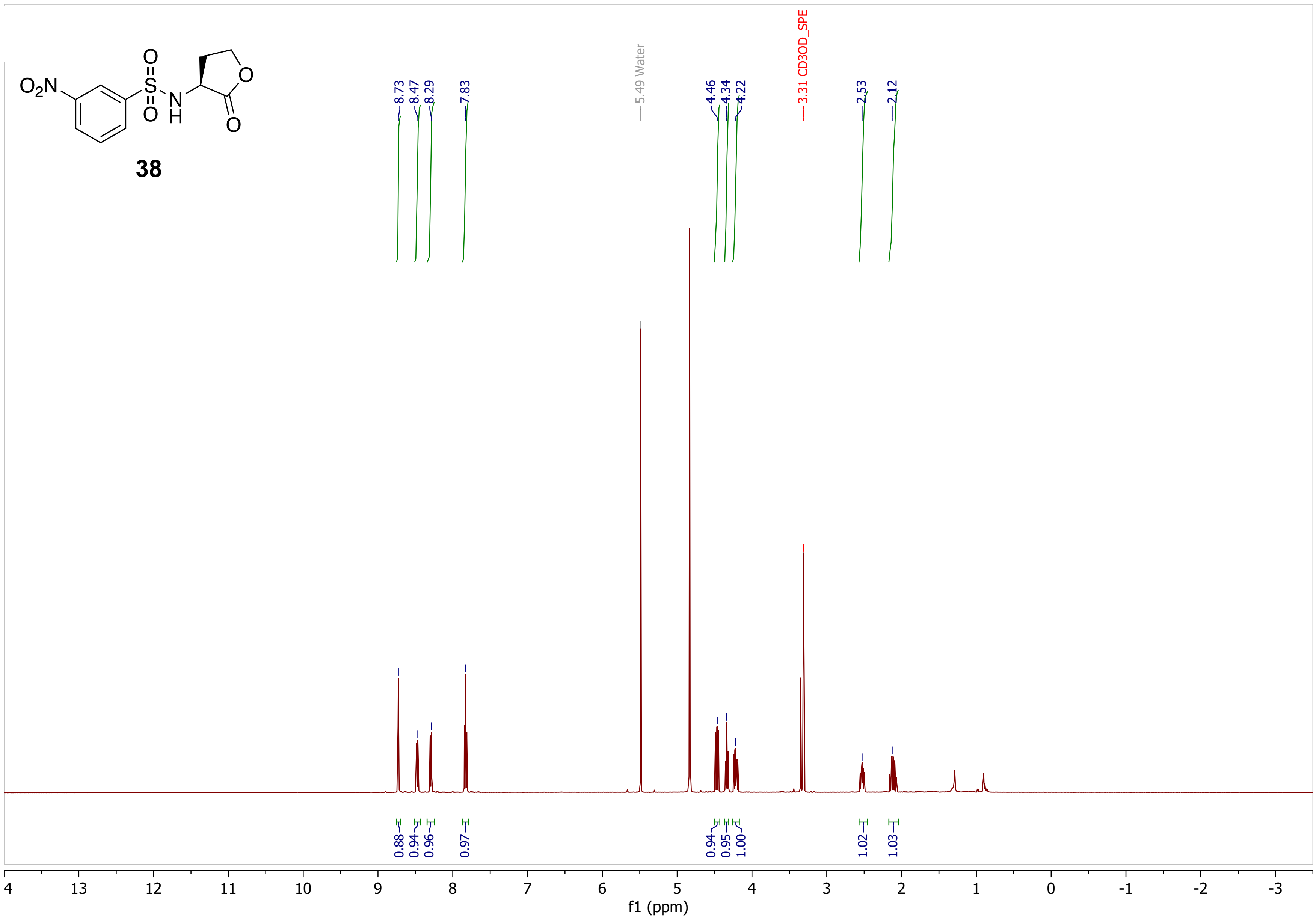

O2N
O
S
O
N
H
O
O
38
8.73
8.47
8.29
7.83
5.49 Water
4.46
4.34
4.22
3.31 CD3OD_SPE
2.53
2.12
0.88
0.94
0.96
0.97
0.94
0.95
1.00
1.02
1.03
f1 (ppm)

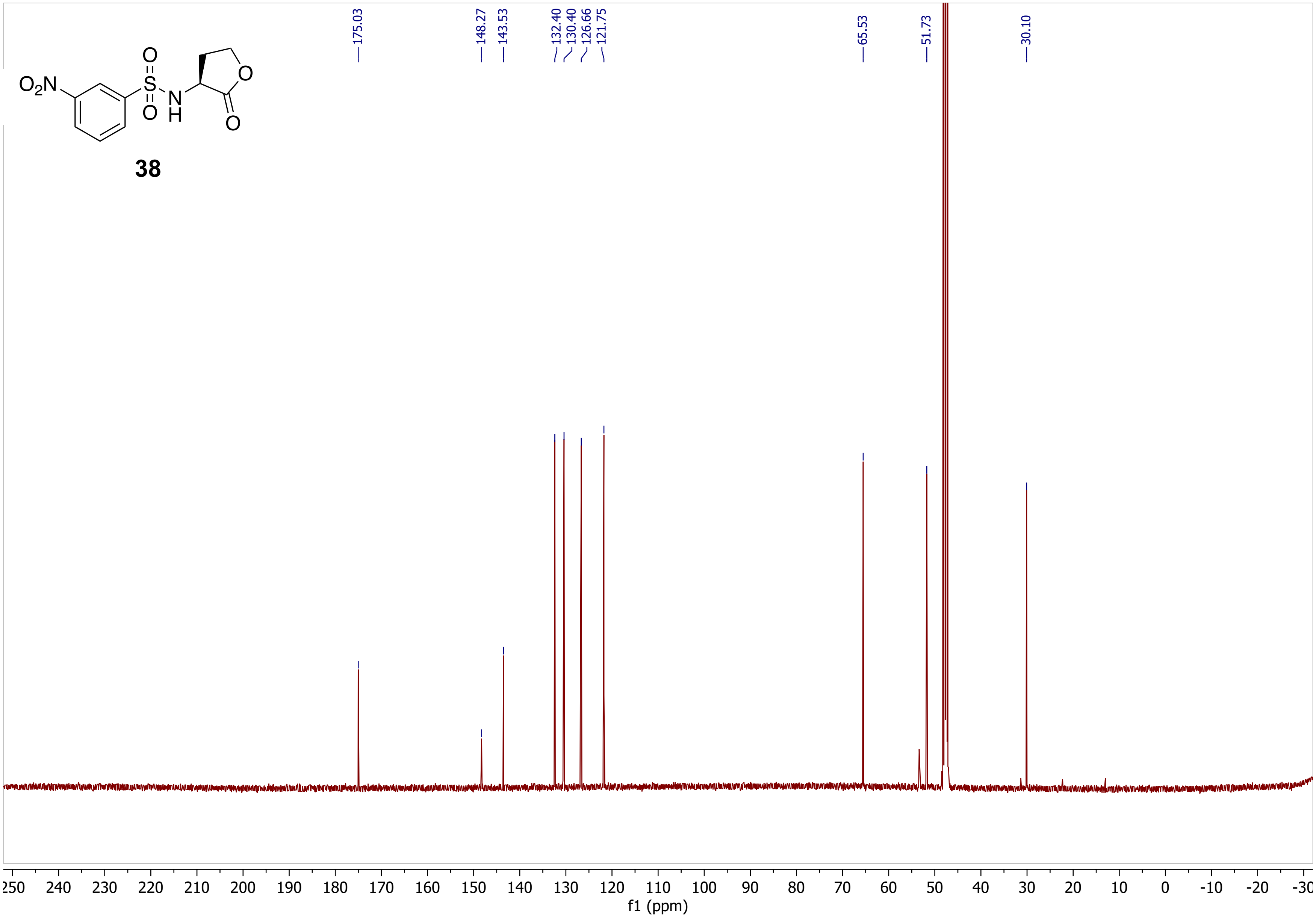

38

175.03
148.27
143.53
132.40
130.40
126.66
121.75
65.53
51.73
30.10
250 240 230 220 210 200 190 180 170 160 150 140 130 120 110 100 90 80 70 60 50 40 30 20 10 0 -10 -20 -30
f1 (ppm)

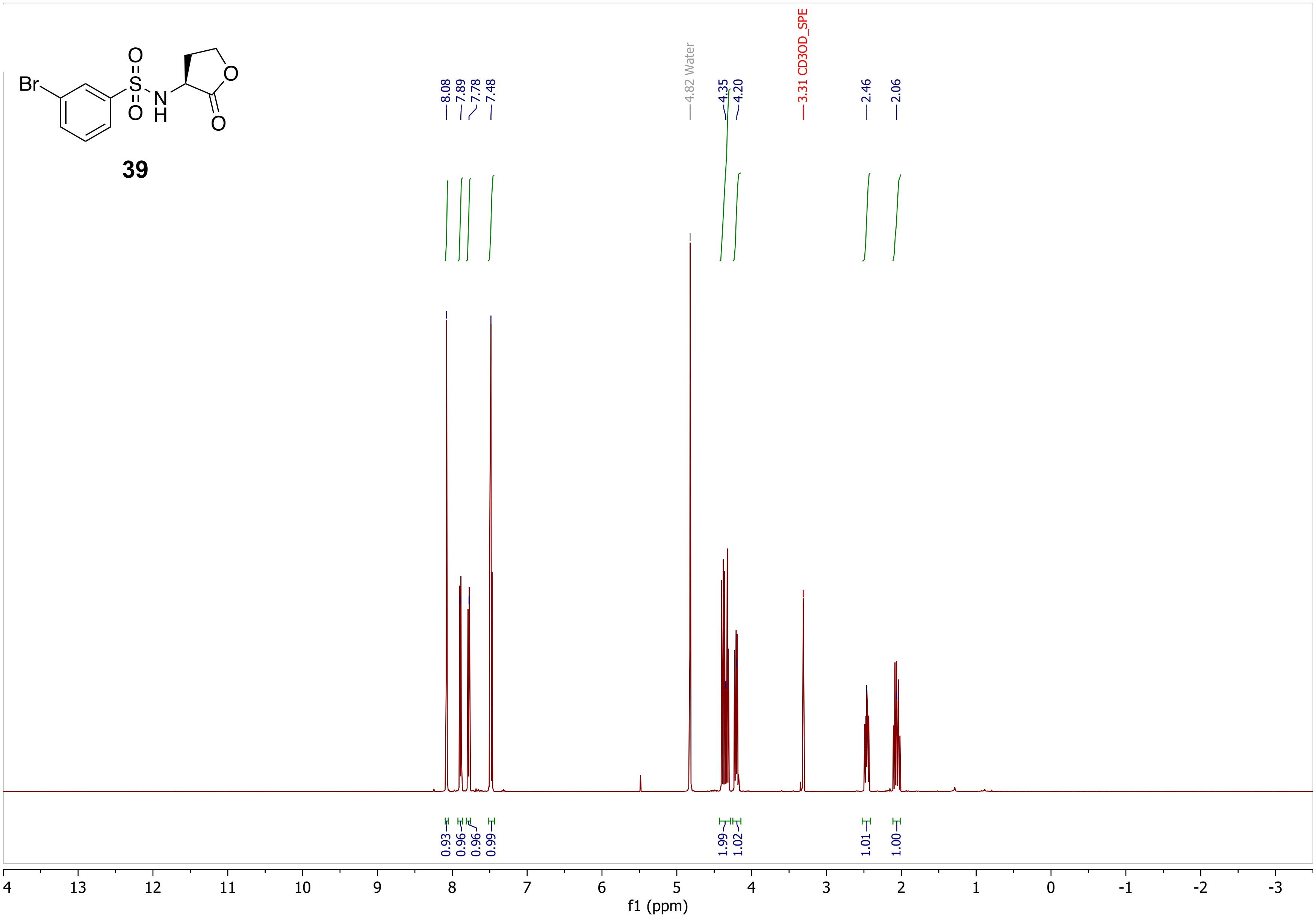

39
8.08
7.89
7.78
7.48
4.82 Water
4.35
4.20
3.31 CD3OD_SPE
2.46
2.06
0.93
0.96
0.96
0.99
1.99
1.02
1.01
1.00
14
13
12
11
10
9
8
7
6
5
4
3
2
1
0
-1
-2
-3
f1 (ppm)

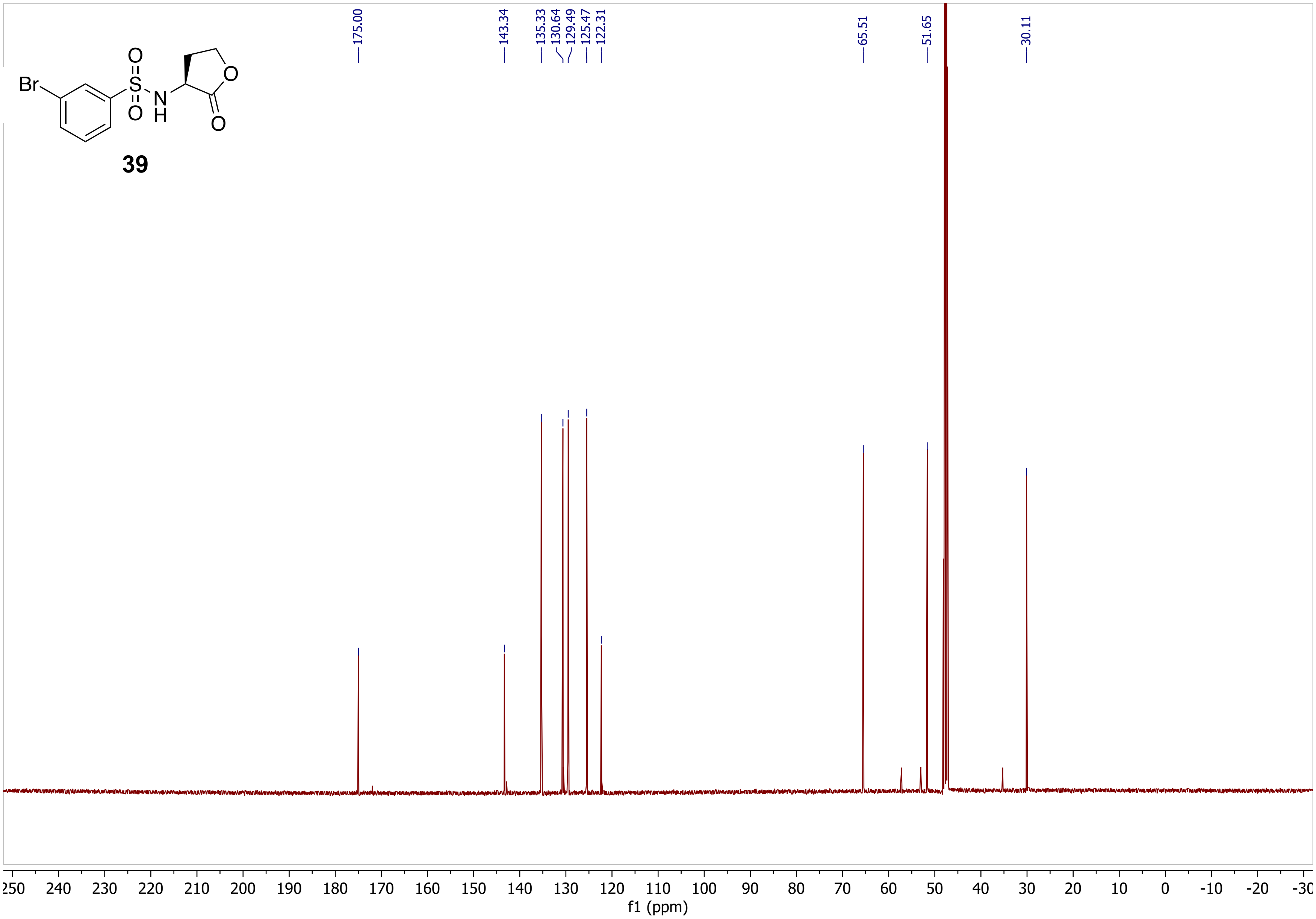
Br
O
S
O
N
H
O
O
175.00
143.34
135.33
130.64
129.49
125.47
122.31
65.51
51.65
30.11
250 240 230 220 210 200 190 180 170 160 150 140 130 120 110 100 90 80 70 60 50 40 30 20 10 0 -10 -20 -30
f1 (ppm)

39

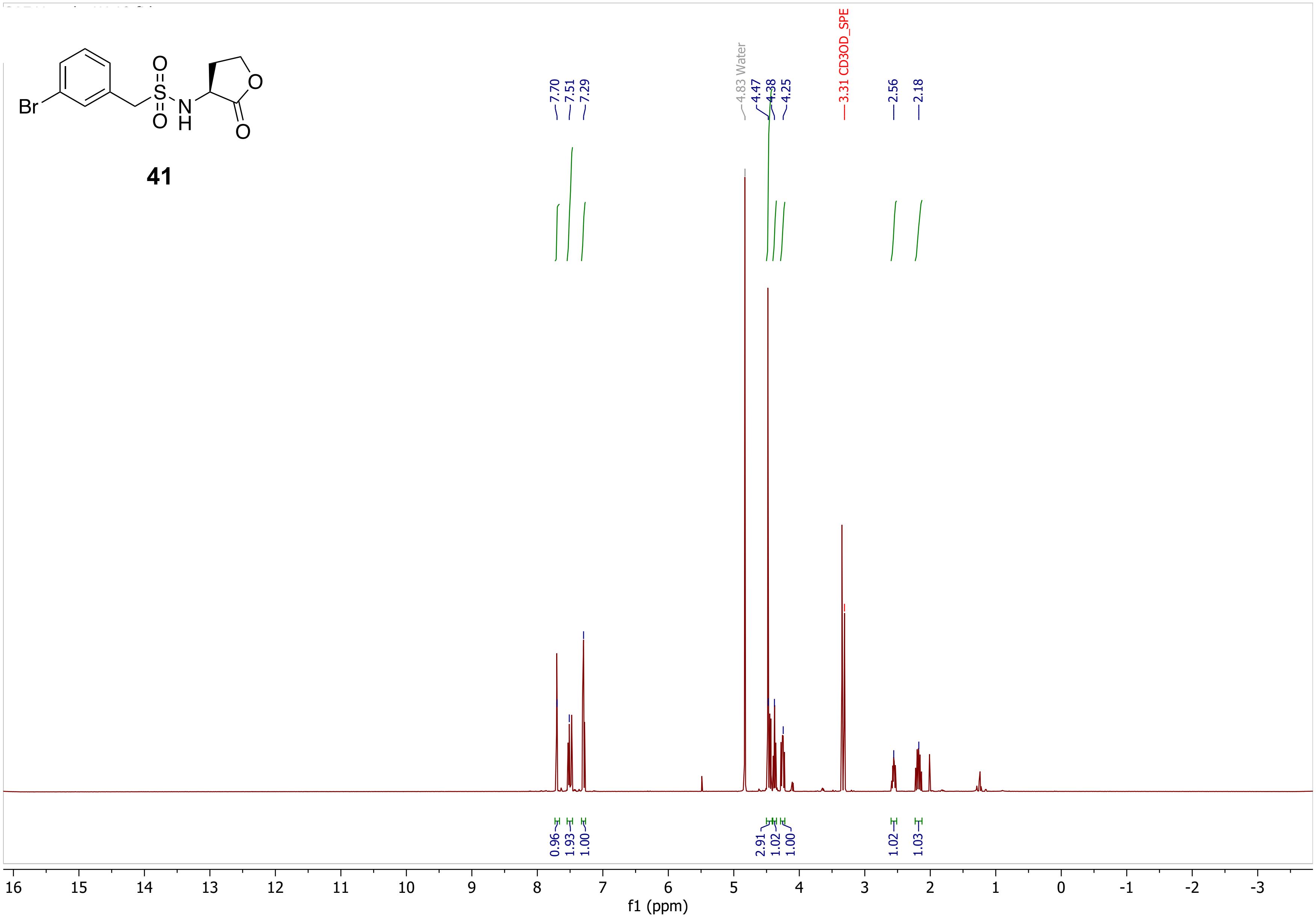

Br
O
S
O
N
H
O
O
41
7.70
7.51
7.29
4.83 Water
4.47
4.38
4.25
3.31 CD3OD_SPE
2.56
2.18
0.96
1.93
1.00
2.91
1.02
1.00
1.02
1.03
16
15
14
13
12
11
10
9
8
7
6
5
4
3
2
1
0
-1
-2
-3
f1 (ppm)

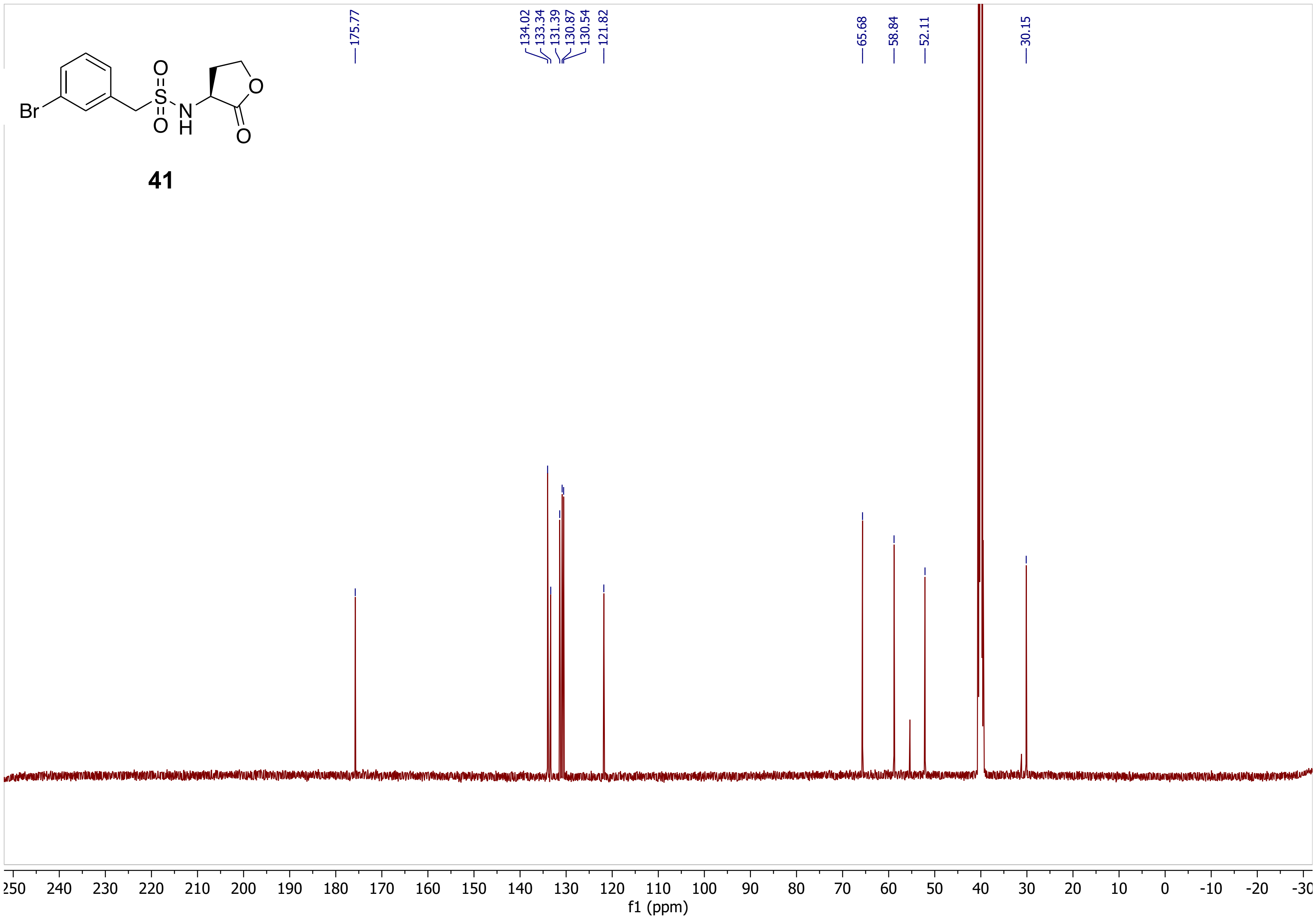
Br
O
S
O
N
H
O
O
41
175.77
134.02
133.34
131.39
130.87
130.54
121.82
65.68
58.84
52.11
30.15
250 240 230 220 210 200 190 180 170 160 150 140 130 120 110 100 90 80 70 60 50 40 30 20 10 0 -10 -20 -30
f1 (ppm)

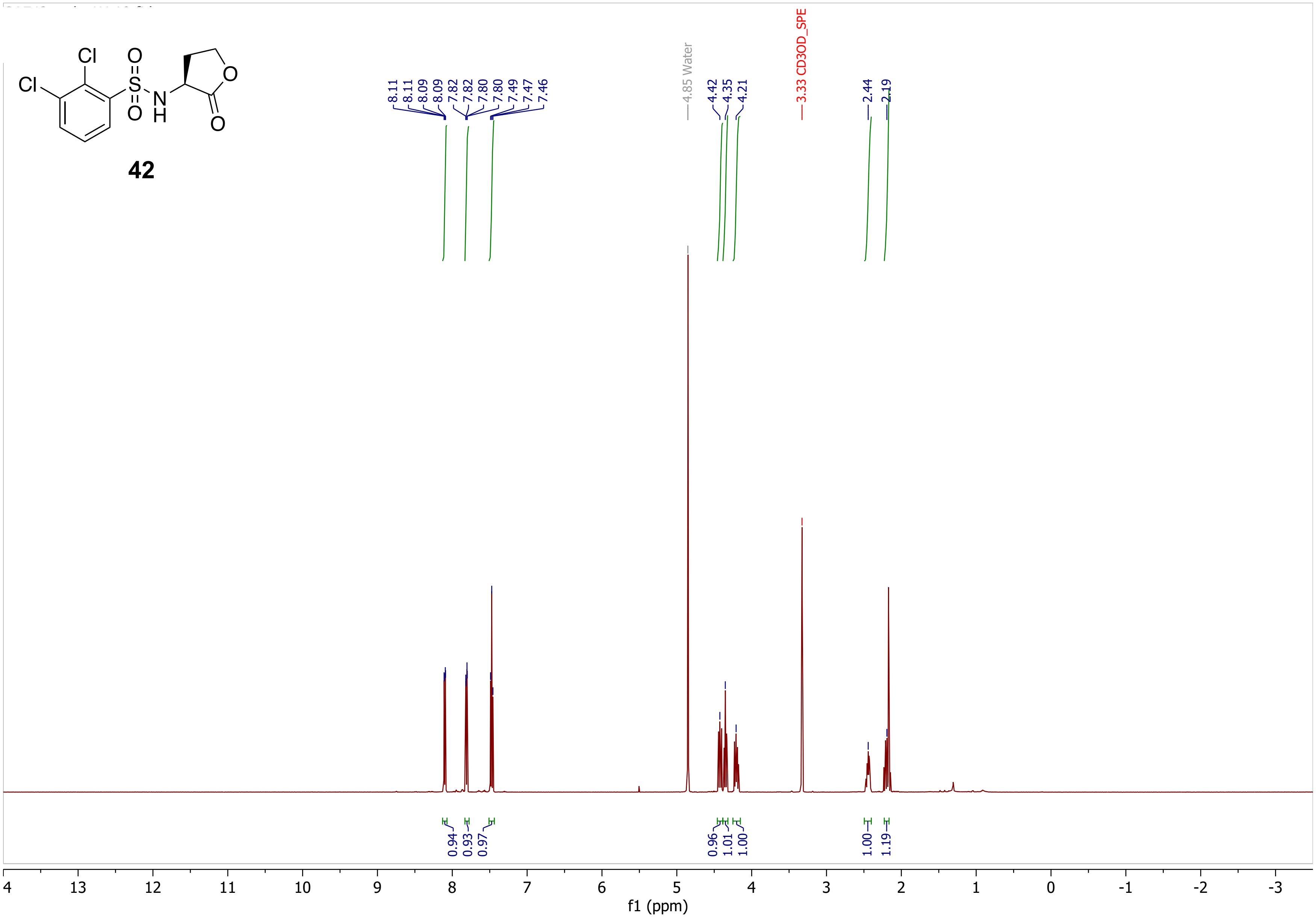
42
8.11
8.11
8.09
8.09
7.82
7.82
7.80
7.80
7.49
7.47
7.46
4.85 Water
4.42
4.35
4.21
3.33 CD3OD_SPE
2.44
2.19
0.94
0.93
0.97
0.96
1.01
1.00
1.00
1.19
f1 (ppm)

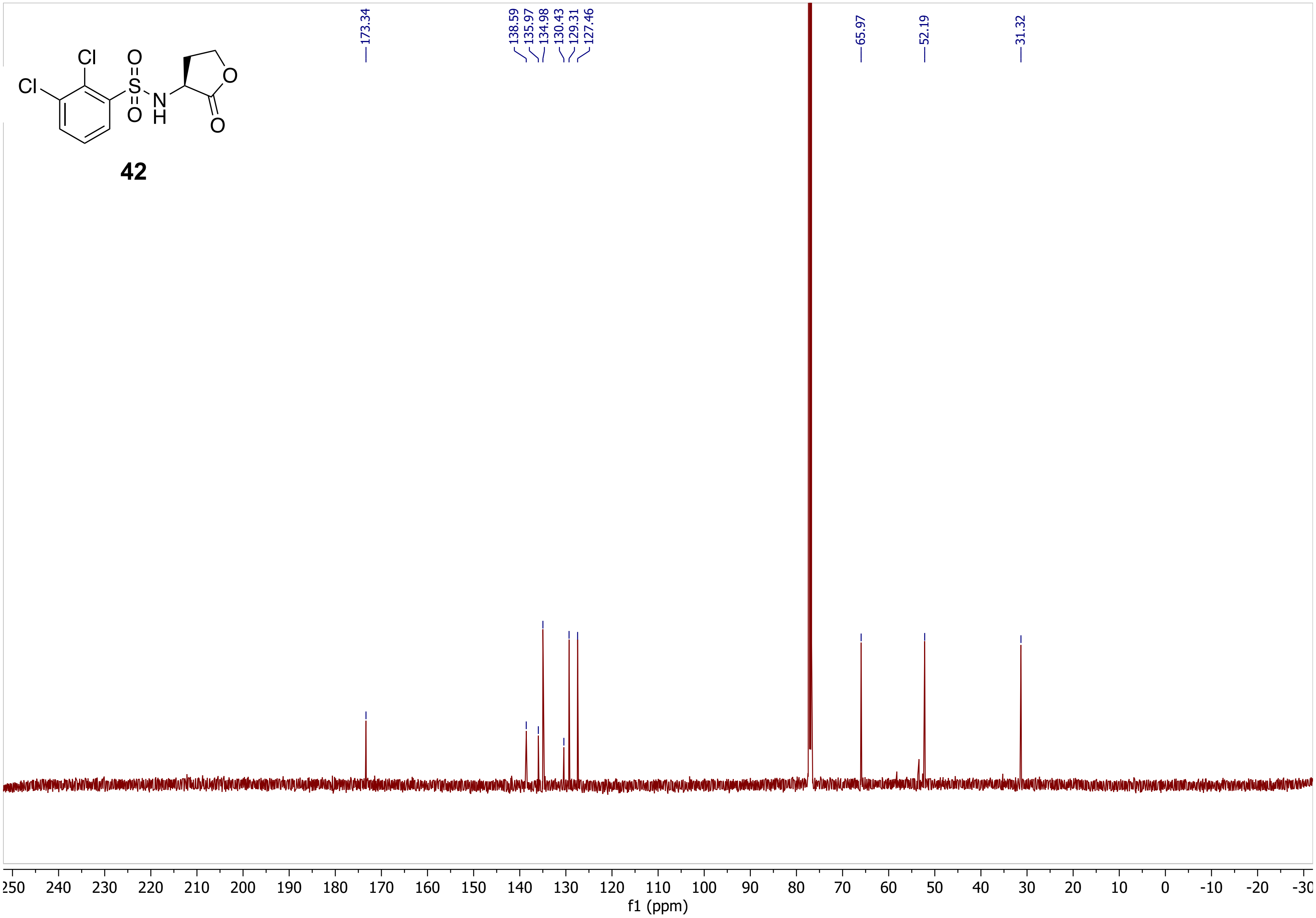
173.34
138.59
135.97
134.98
130.43
129.31
127.46
65.97
52.19
31.32
Cl
Cl
O
O
S
N
H
O
O
42
250 240 230 220 210 200 190 180 170 160 150 140 130 120 110 100 90 80 70 60 50 40 30 20 10 0 -10 -20 -30
f1 (ppm)

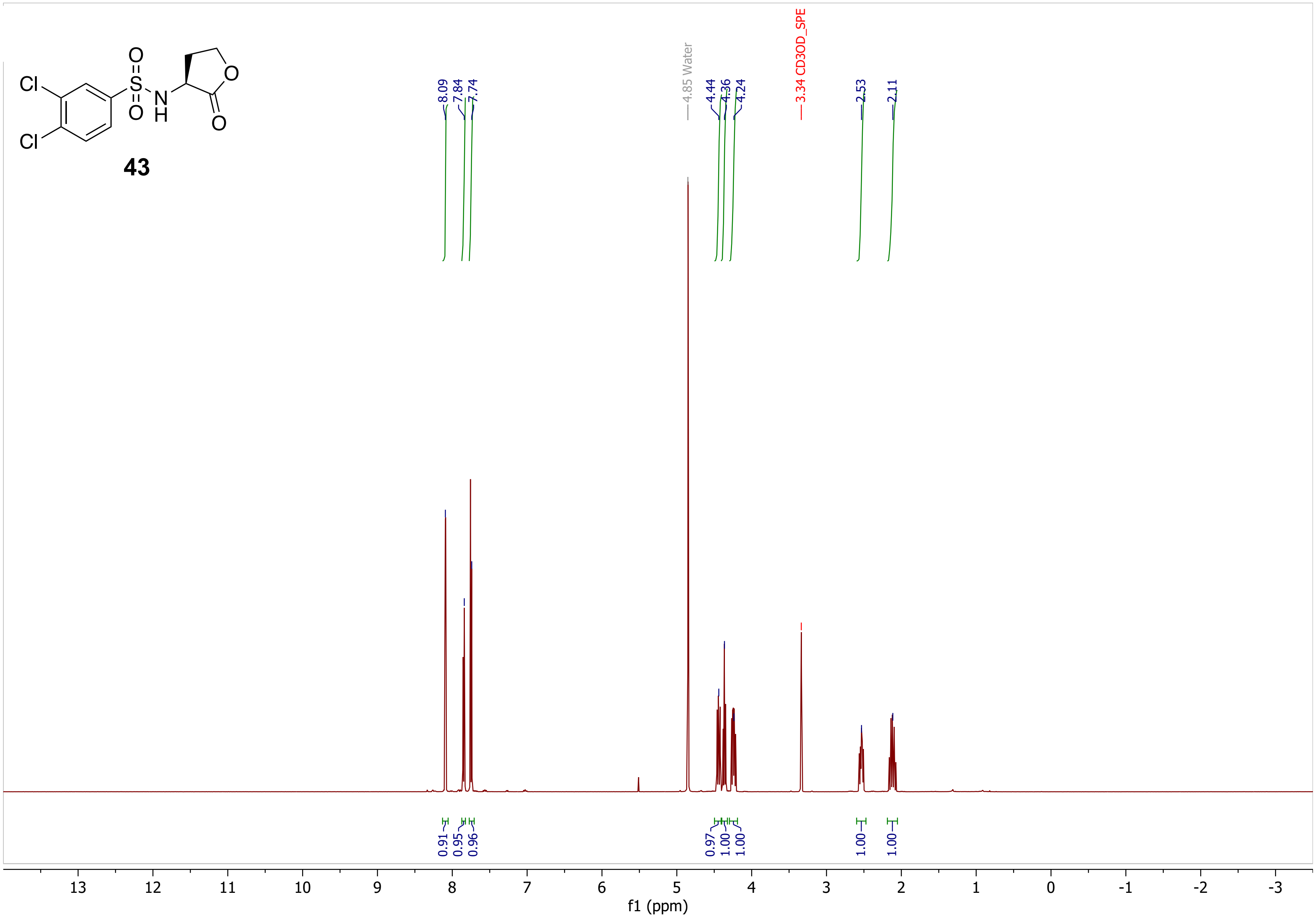
Cl
Cl
O
S
O
N
H
O
O
43
8.09
7.84
7.74
4.85 Water
4.44
4.36
4.24
3.34 CD3OD_SPE
2.53
2.11
0.91
0.95
0.96
0.97
1.00
1.00
1.00
1.00
13
12
11
10
9
8
7
6
5
4
3
2
1
0
-1
-2
-3
f1 (ppm)

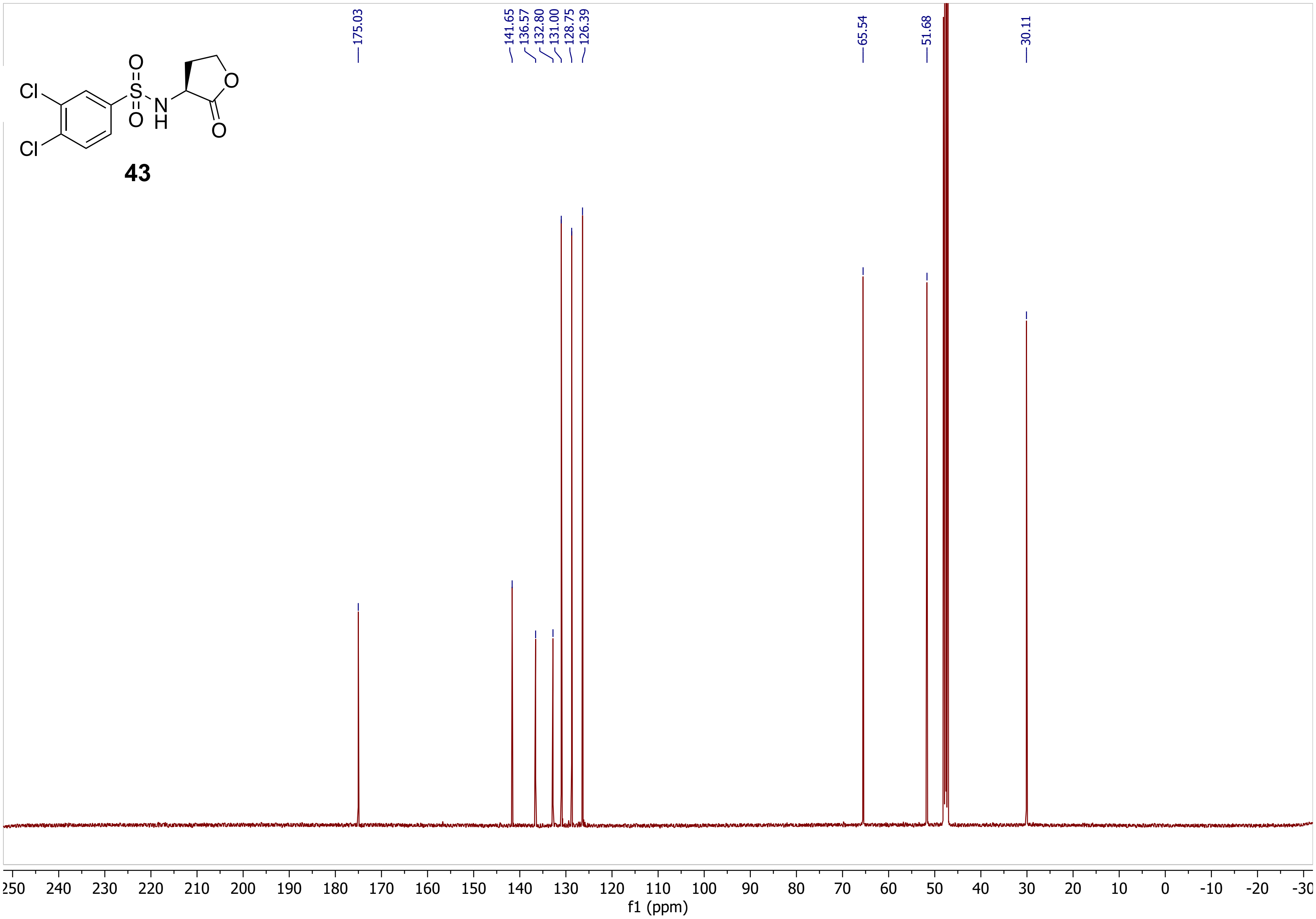
175.03
141.65
136.57
132.80
131.00
128.75
126.39
65.54
51.68
30.11
43
250 240 230 220 210 200 190 180 170 160 150 140 130 120 110 100 90 80 70 60 50 40 30 20 10 0 -10 -20 -30
f1 (ppm)

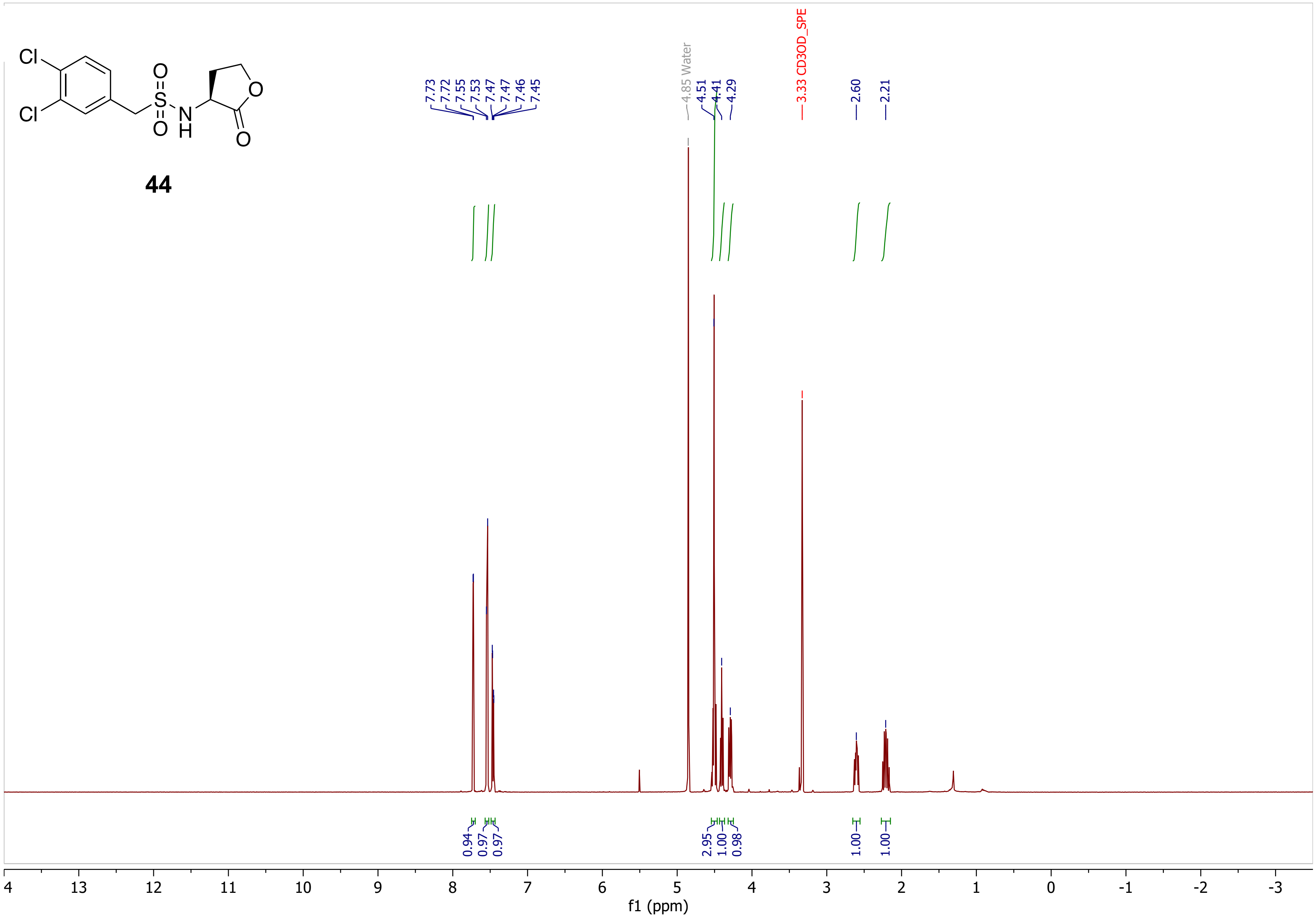
44
7.73
7.72
7.55
7.53
7.47
7.47
7.46
7.45
4.85 Water
4.51
4.41
4.29
3.33 CD3OD_SPE
2.60
2.21
0.94
0.97
0.97
2.95
1.00
0.98
1.00
1.00
f1 (ppm)

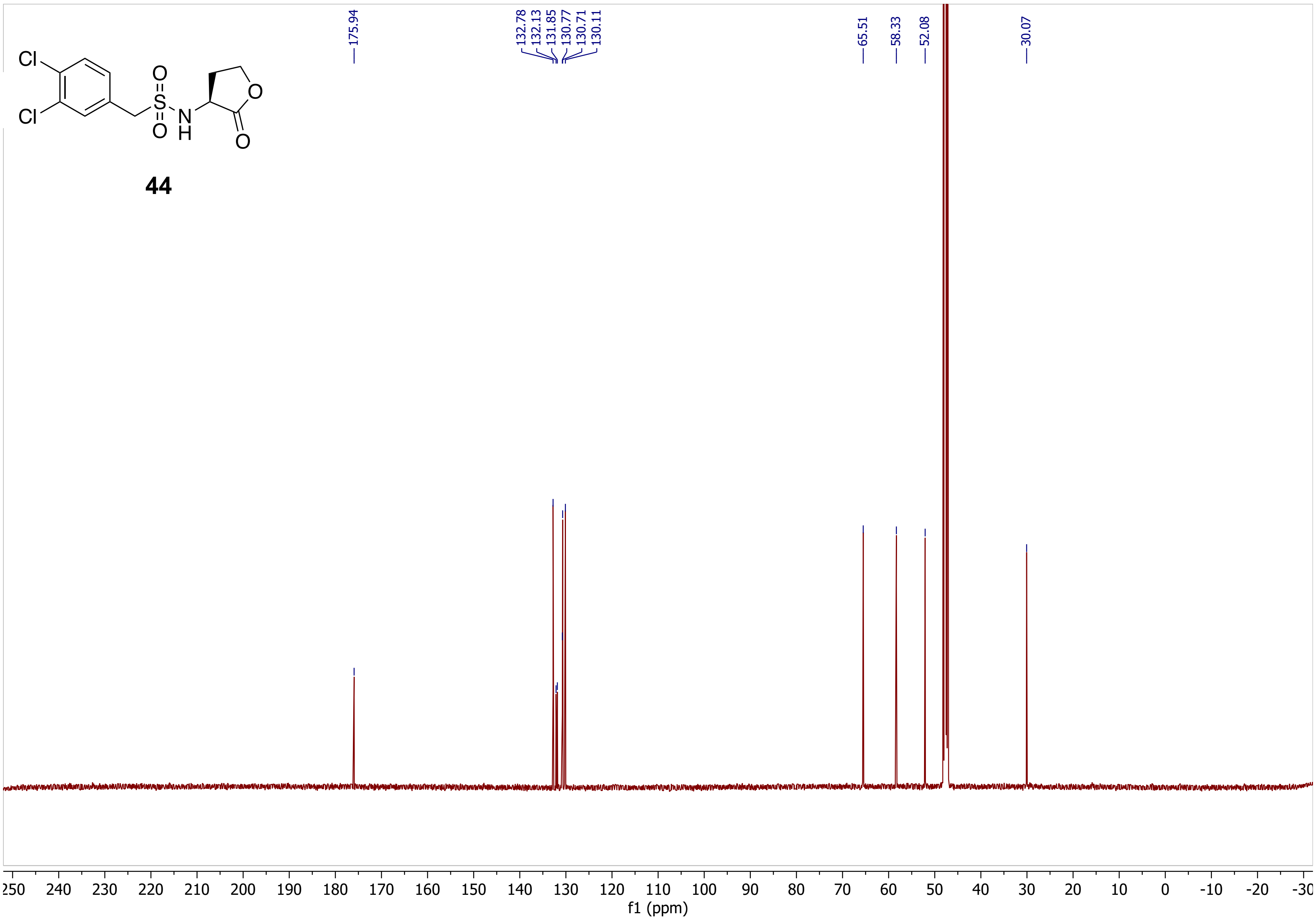
Cl
Cl
O
S
O
N
H
O
O
44
175.94
132.78
132.13
131.85
130.77
130.71
130.11
65.51
58.33
52.08
30.07
250
240
230
220
210
200
190
180
170
160
150
140
130
120
110
100
90
80
70
60
50
40
30
20
10
0
-10
-20
-30
f1 (ppm)

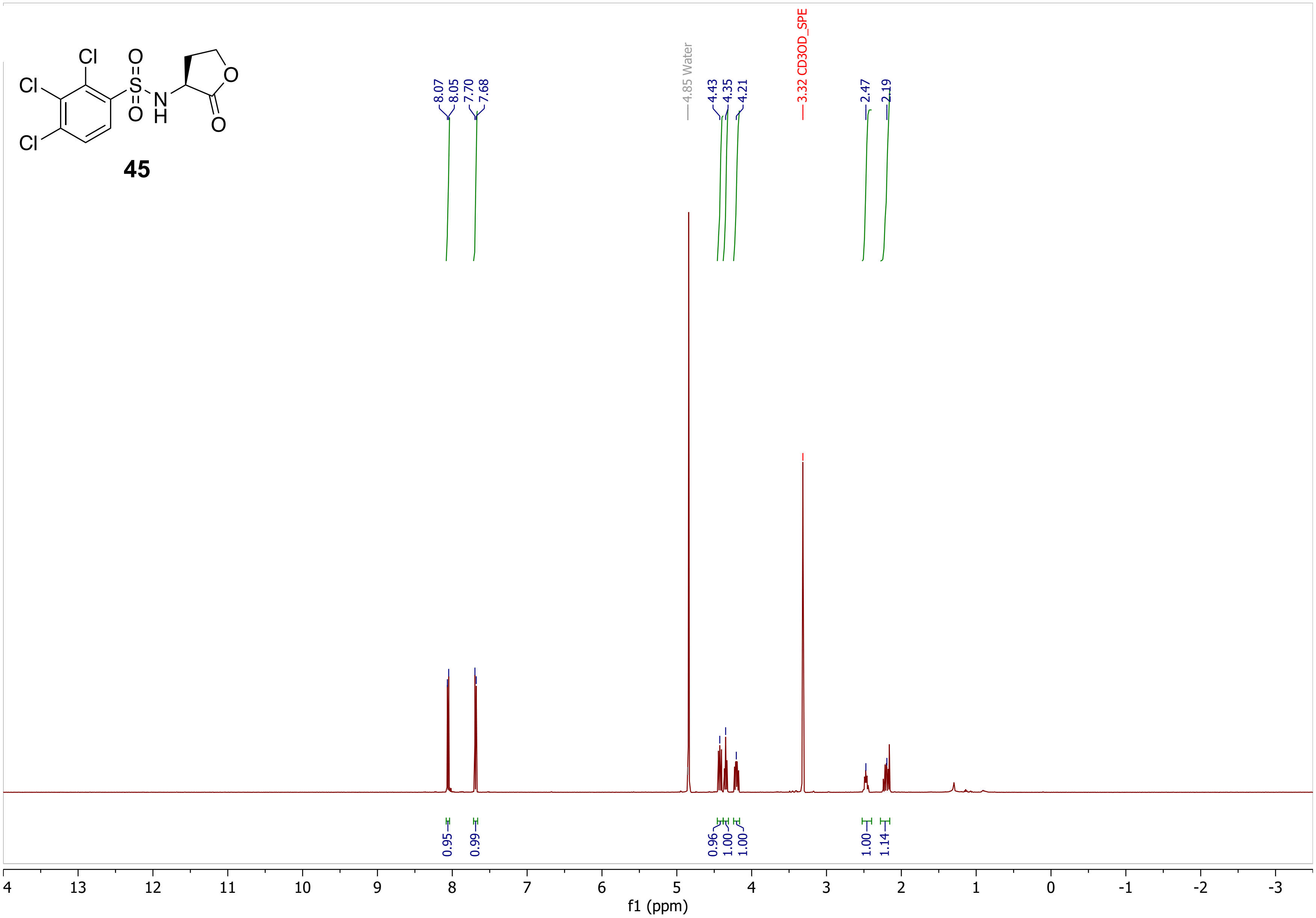
Cl
Cl
Cl
O
S
O
N
H
O
O
45
8.07
8.05
7.70
7.68
4.85 Water
4.43
4.35
4.21
3.32 CD3OD_SPE
2.47
2.19
0.95
0.99
0.96
1.00
1.00
1.00
1.14
4
13
12
11
10
9
8
7
6
5
4
3
2
1
0
-1
-2
-3
f1 (ppm)

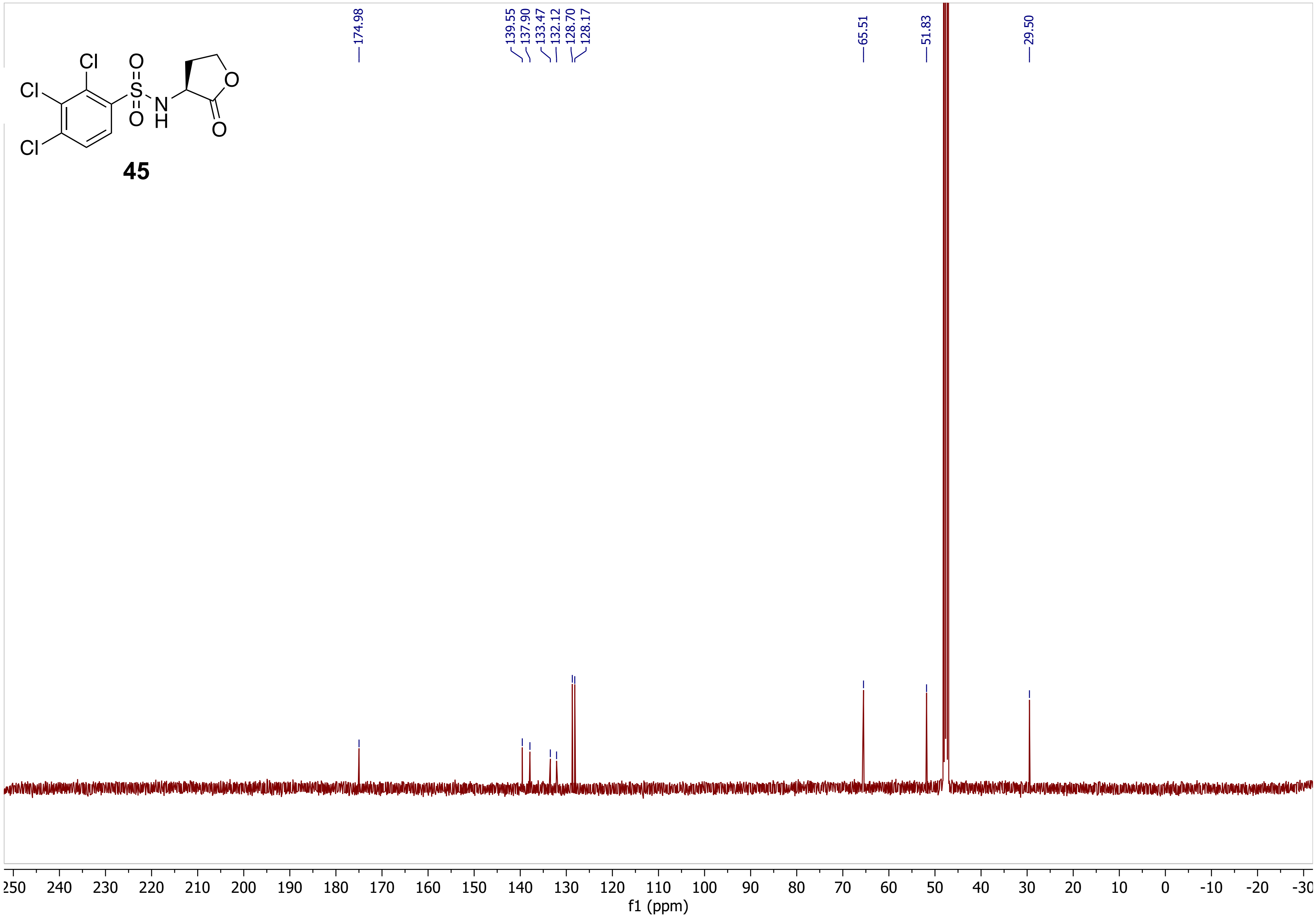
Cl
Cl
Cl
O
S
O
N
H
O
O
45
174.98
139.55
137.90
133.47
132.12
128.70
128.17
65.51
51.83
29.50
250
240
230
220
210
200
190
180
170
160
150
140
130
120
110
100
90
80
70
60
50
40
30
20
10
0
-10
-20
-30
f1 (ppm)

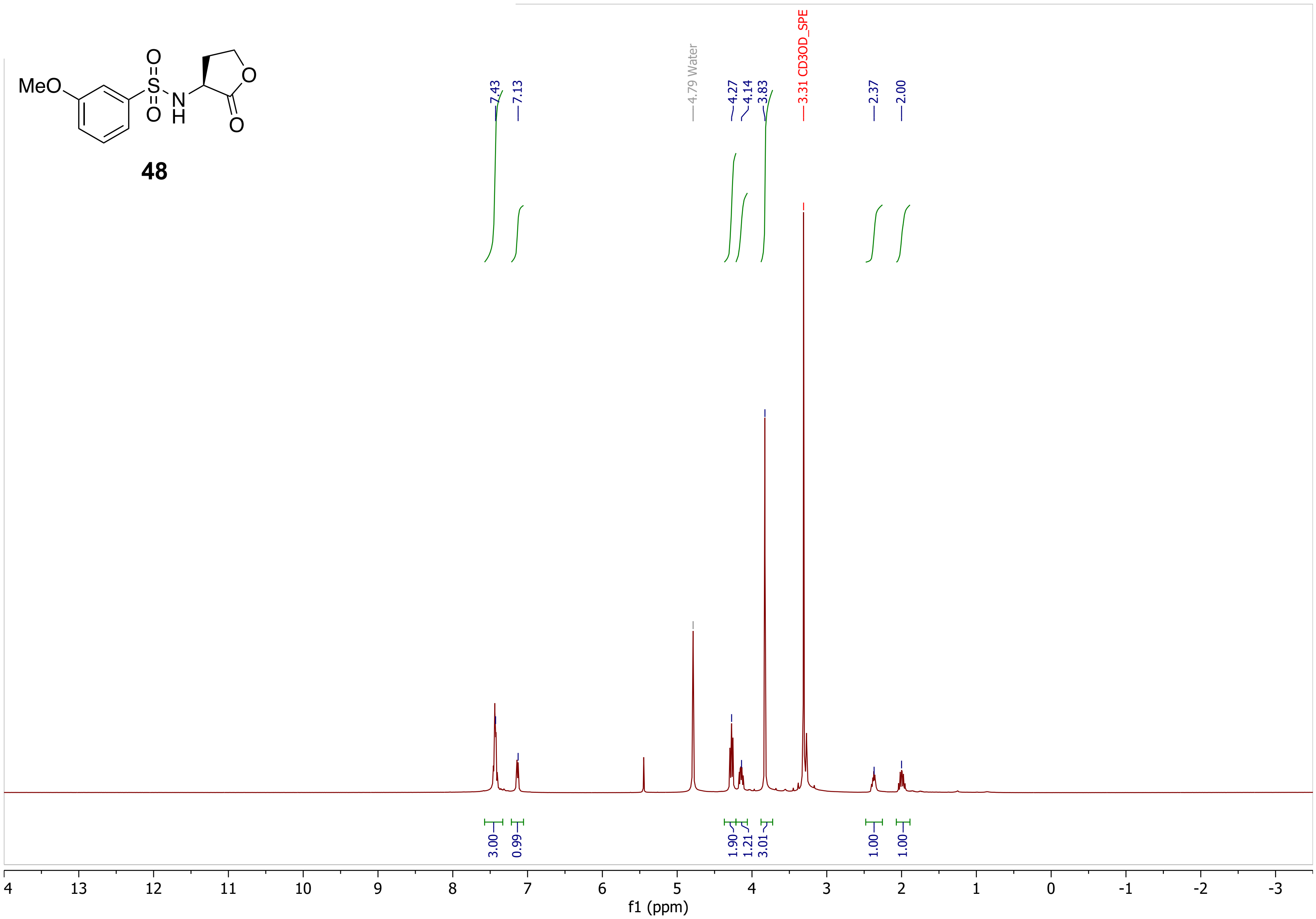
MeO
48
7.43
7.13
4.79 Water
4.27
4.14
3.83
3.31 CD3OD_SPE
2.37
2.00
3.00
0.99
1.90
1.21
3.01
1.00
1.00
f1 (ppm)

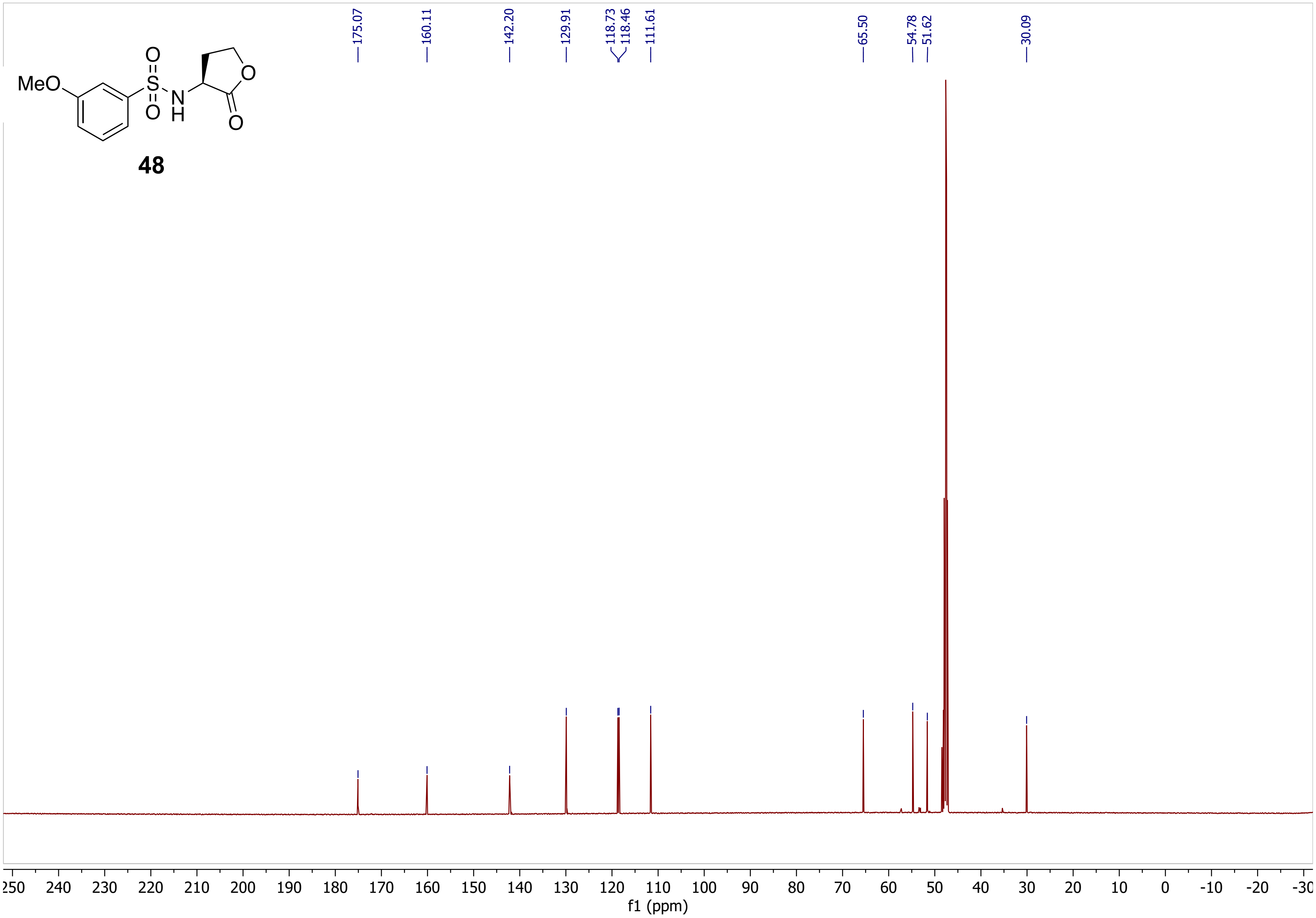
48
175.07
160.11
142.20
129.91
118.73
118.46
111.61
65.50
54.78
51.62
30.09
250 240 230 220 210 200 190 180 170 160 150 140 130 120 110 100 90 80 70 60 50 40 30 20 10 0 -10 -20 -30
f1 (ppm)

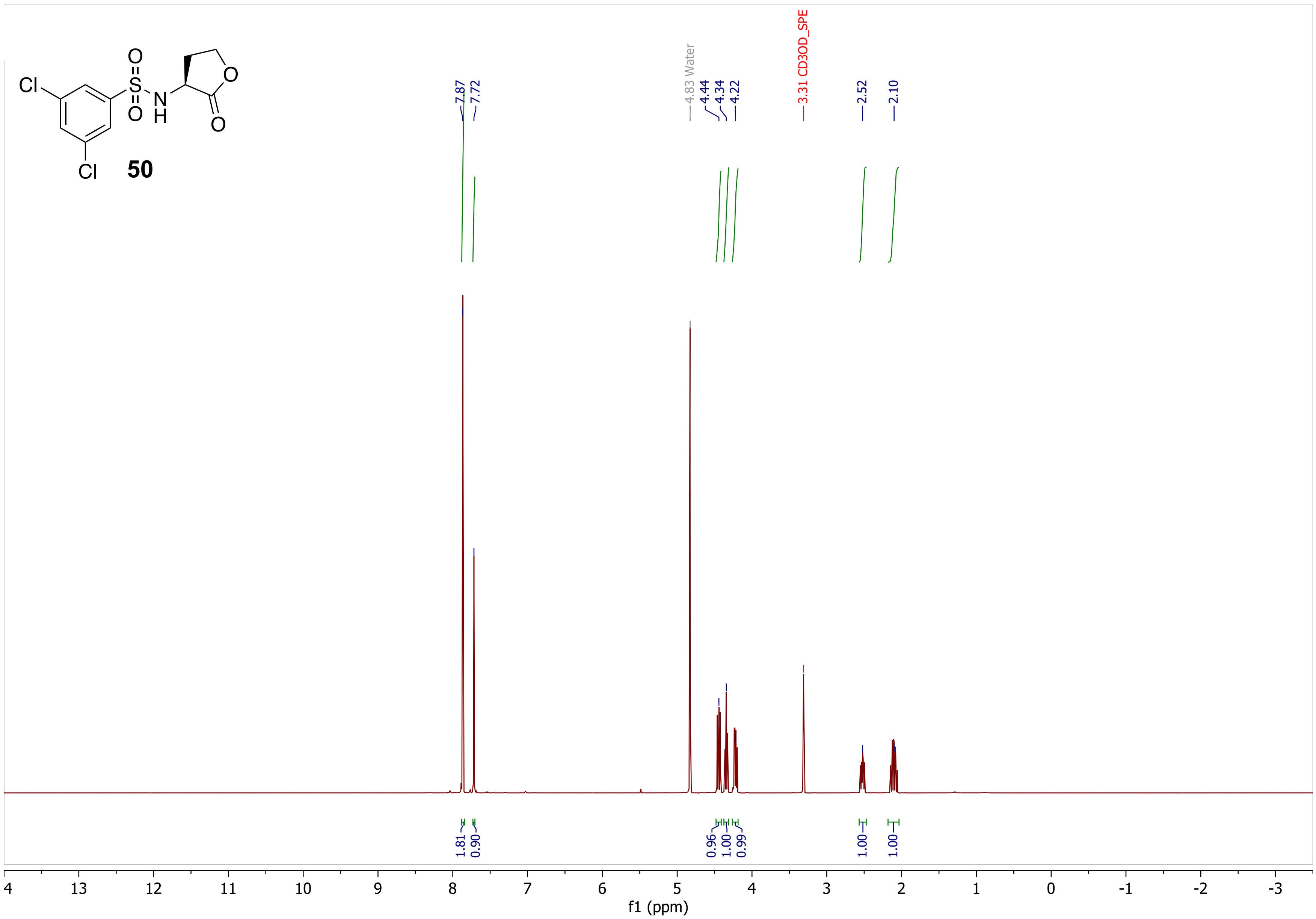
50
7.87
7.72
4.83 Water
4.44
4.34
4.22
3.31 CD3OD_SPE
2.52
2.10
1.81
0.90
0.96
1.00
0.99
1.00
1.00
f1 (ppm)

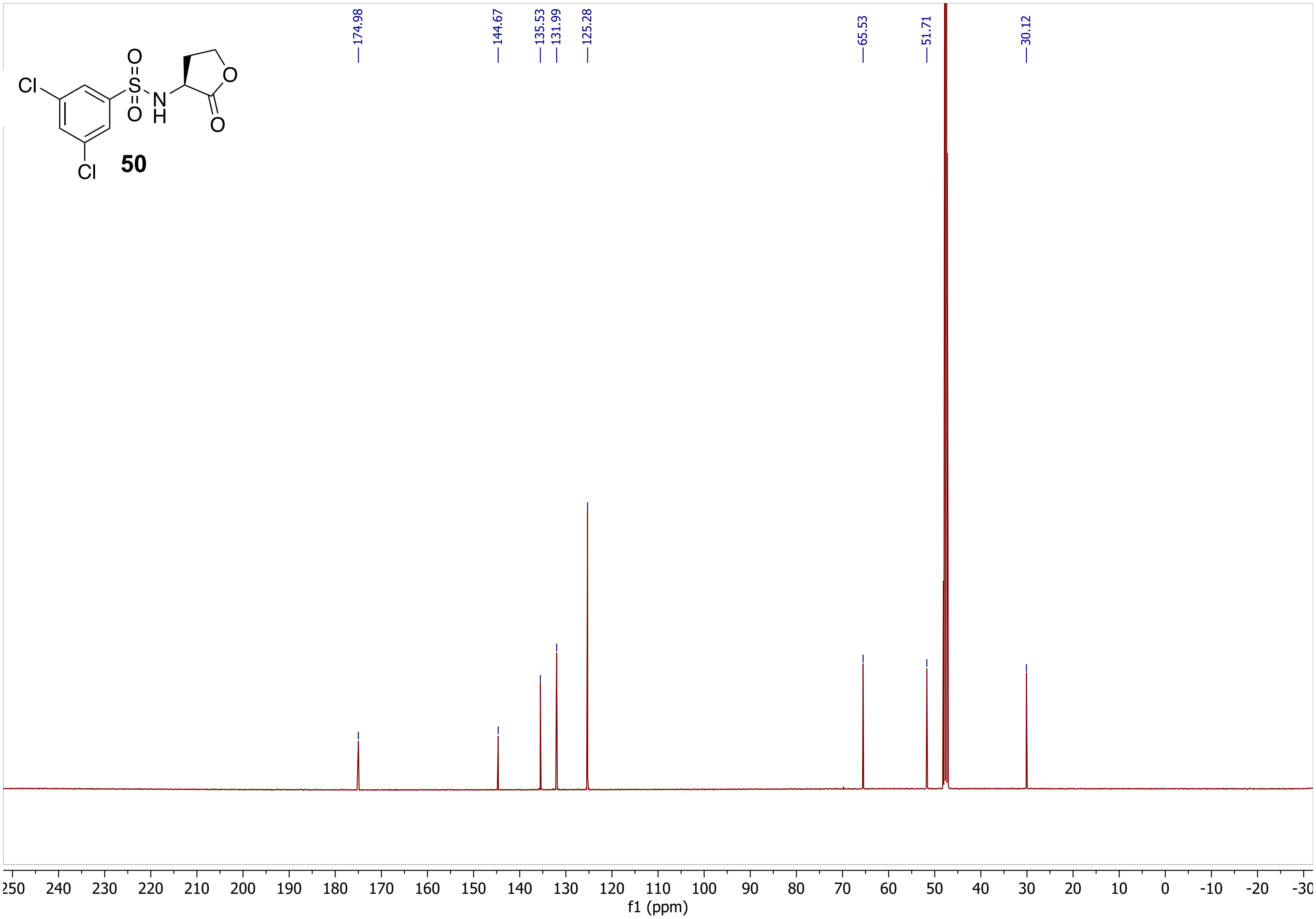
174.98
144.67
135.53
131.99
125.28
65.53
51.71
30.12
50
250 240 230 220 210 200 190 180 170 160 150 140 130 120 110 100 90 80 70 60 50 40 30 20 10 0 -10 -20 -30
f1 (ppm)

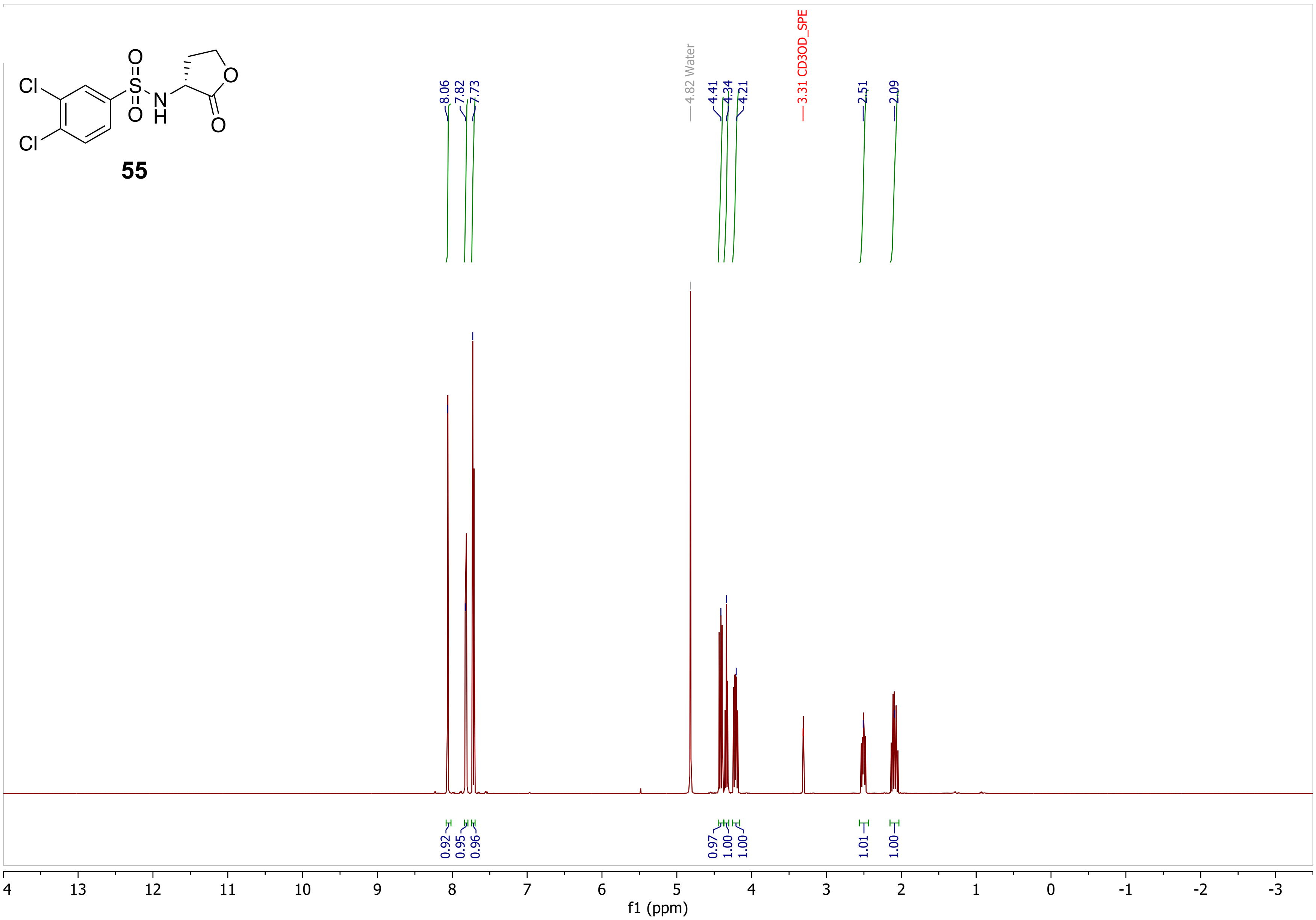
55
8.06
7.82
7.73
4.82 Water
4.41
4.34
4.21
3.31 CD3OD_SPE
2.51
2.09
0.92
0.95
0.96
0.97
1.00
1.00
1.01
1.00
f1 (ppm)

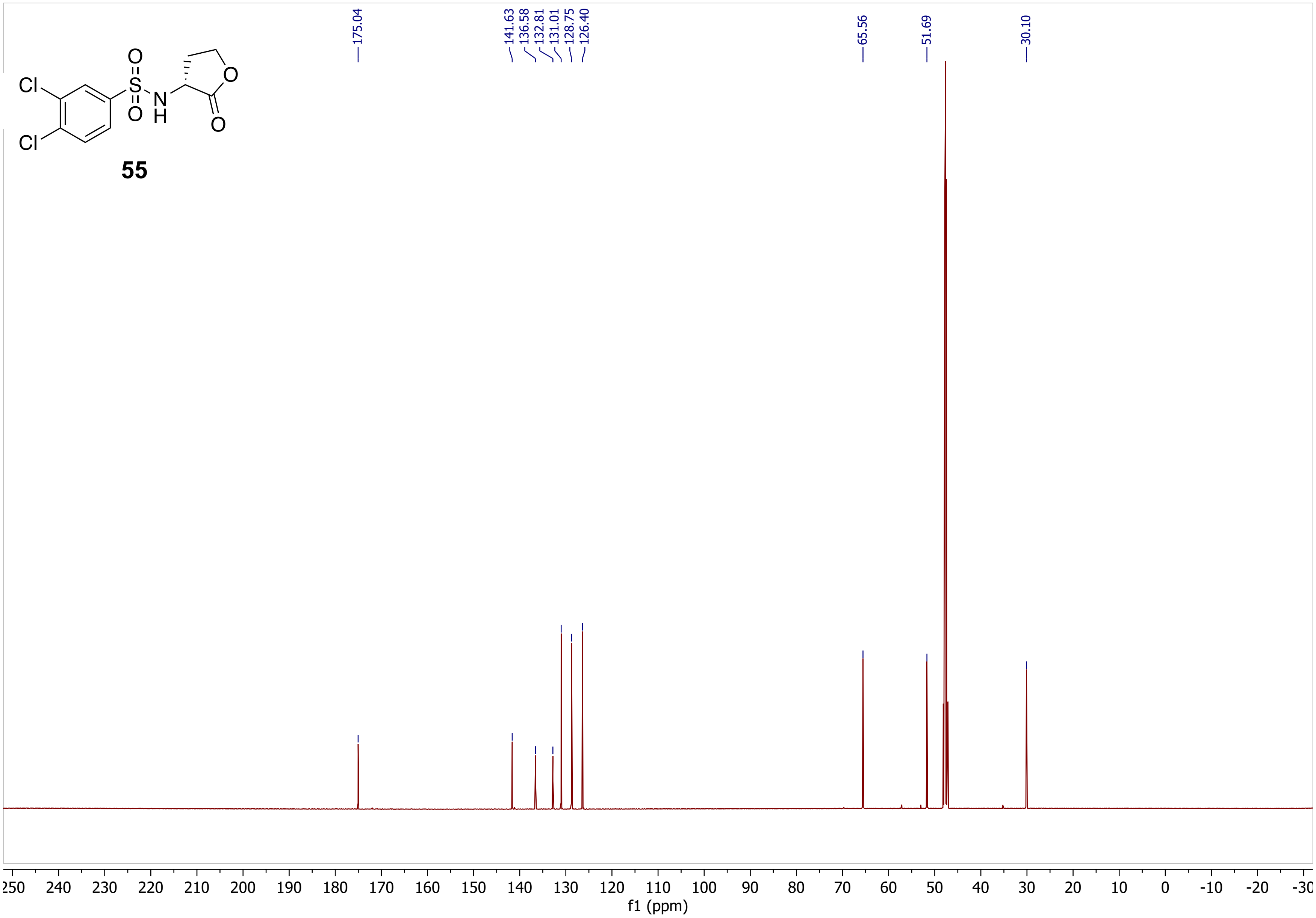

175.04
141.63
136.58
132.81
131.01
128.75
126.40
65.56
51.69
30.10
Cl
Cl
O
S
O
N
H
O
O
55
250 240 230 220 210 200 190 180 170 160 150 140 130 120 110 100 90 80 70 60 50 40 30 20 10 0 -10 -20 -30
f1 (ppm)

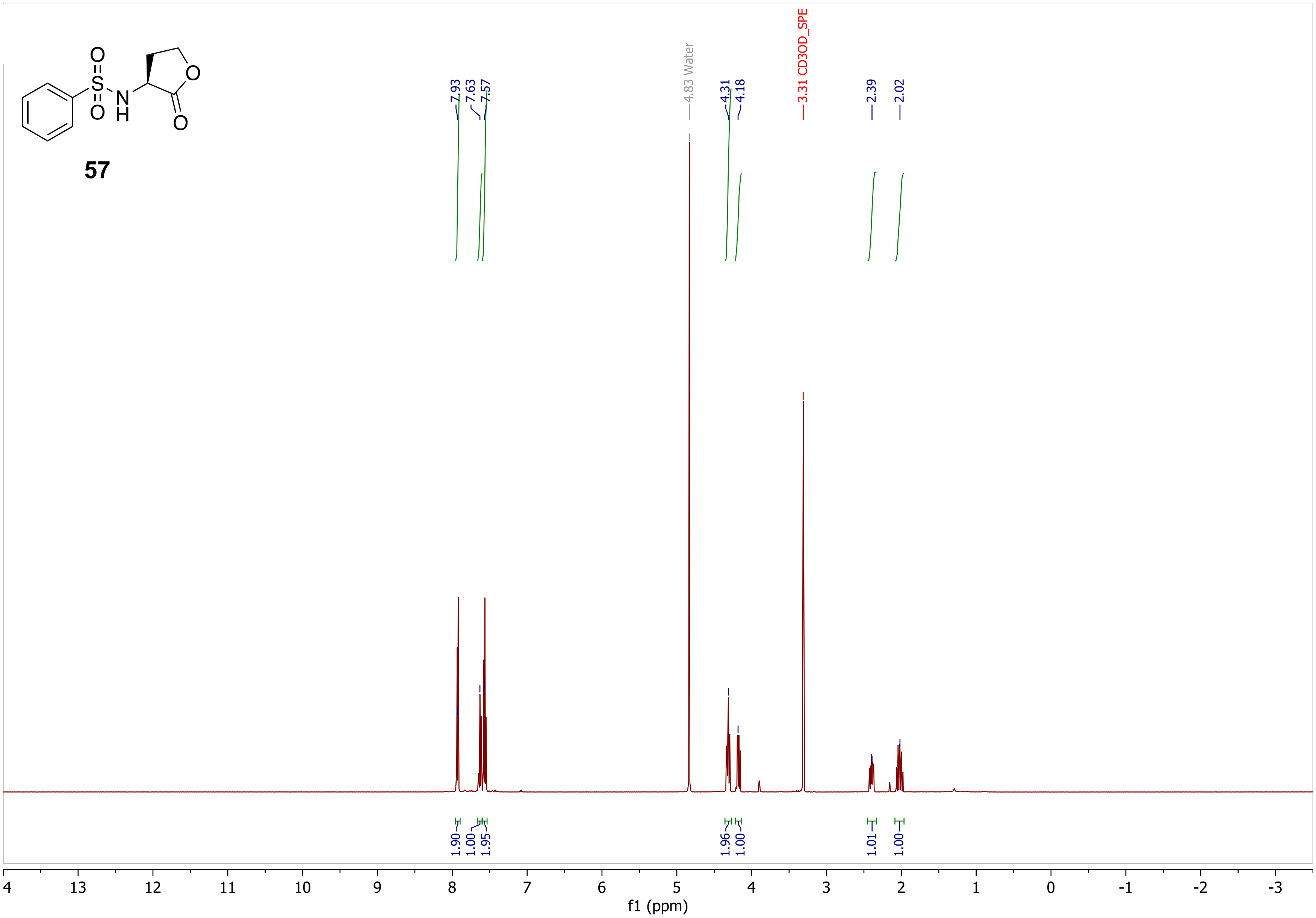
57
7.93
7.63
7.57
4.83 Water
4.31
4.18
3.31 CD3OD_SPE
2.39
2.02
1.90
1.00
1.95
1.96
1.00
1.01
1.00
4
13
12
11
10
9
8
7
6
5
4
3
2
1
0
-1
-2
-3
f1 (ppm)

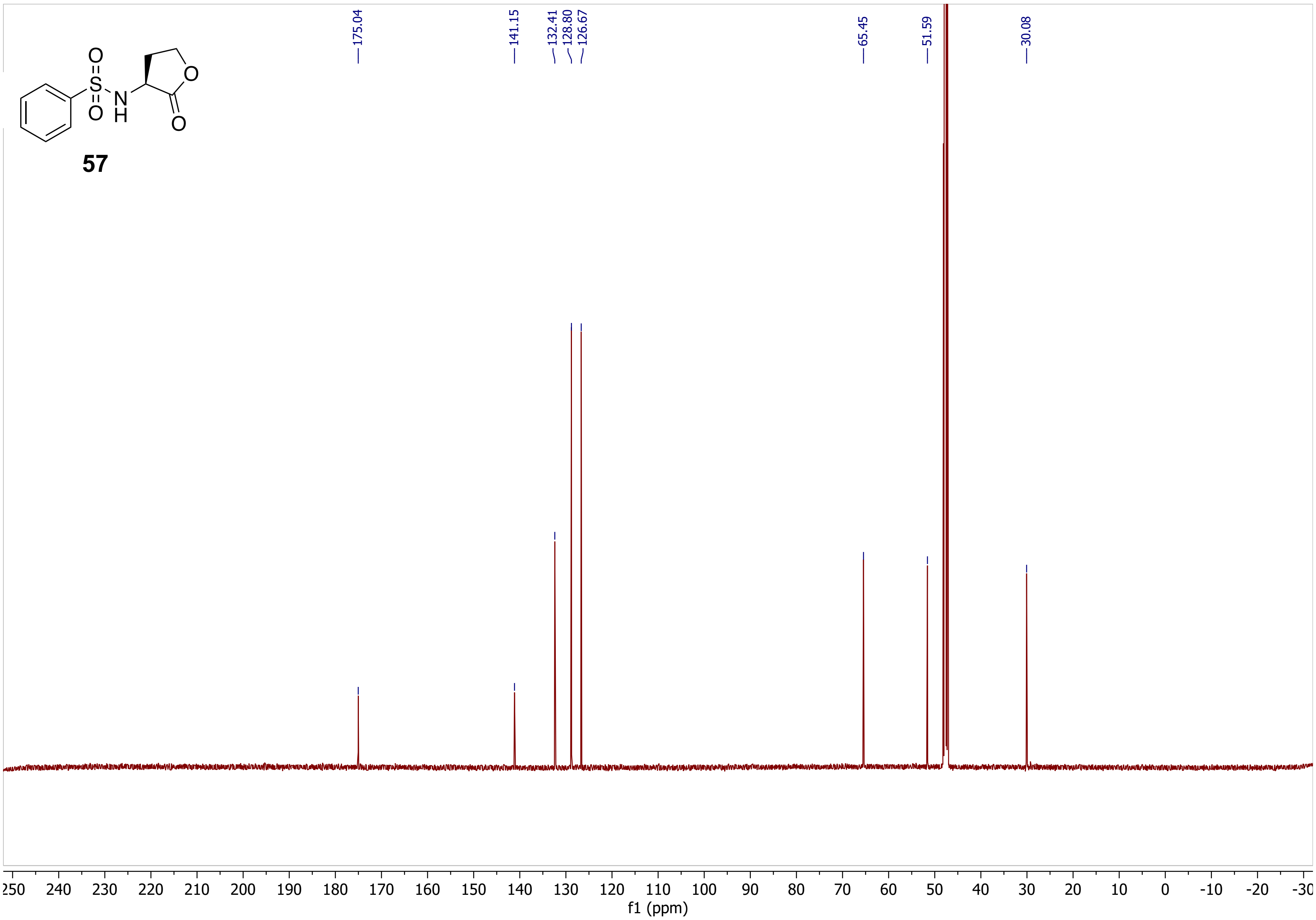
175.04
141.15
132.41
128.80
126.67
65.45
51.59
30.08
57
250 240 230 220 210 200 190 180 170 160 150 140 130 120 110 100 90 80 70 60 50 40 30 20 10 0 -10 -20 -30
f1 (ppm)

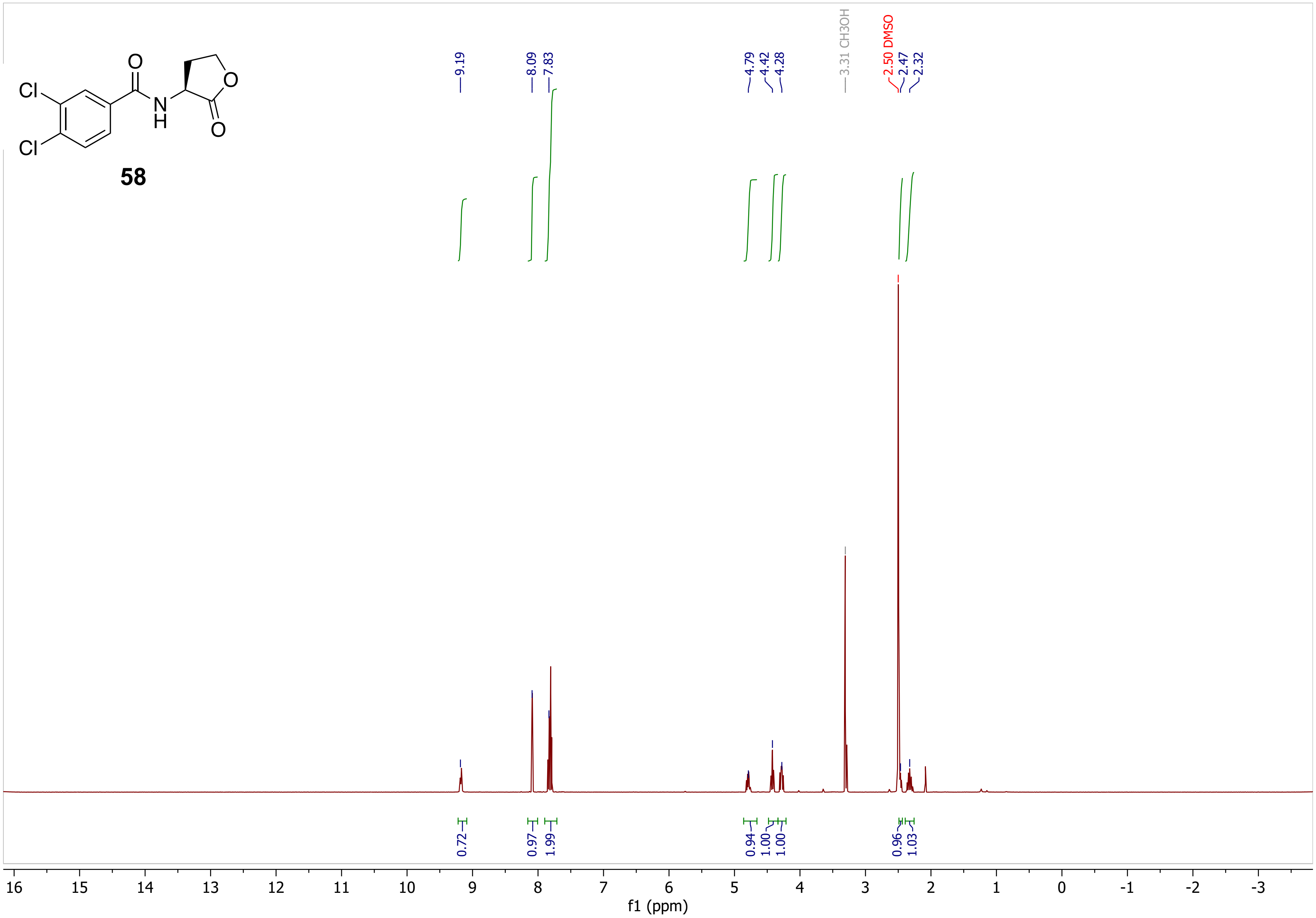
58
9.19
8.09
7.83
4.79
4.42
4.28
3.31 CH3OH
2.50 DMSO
2.47
2.32
0.72
0.97
1.99
0.94
1.00
1.00
0.96
1.03
16 15 14 13 12 11 10 9 8 7 6 5 4 3 2 1 0 -1 -2 -3
f1 (ppm)

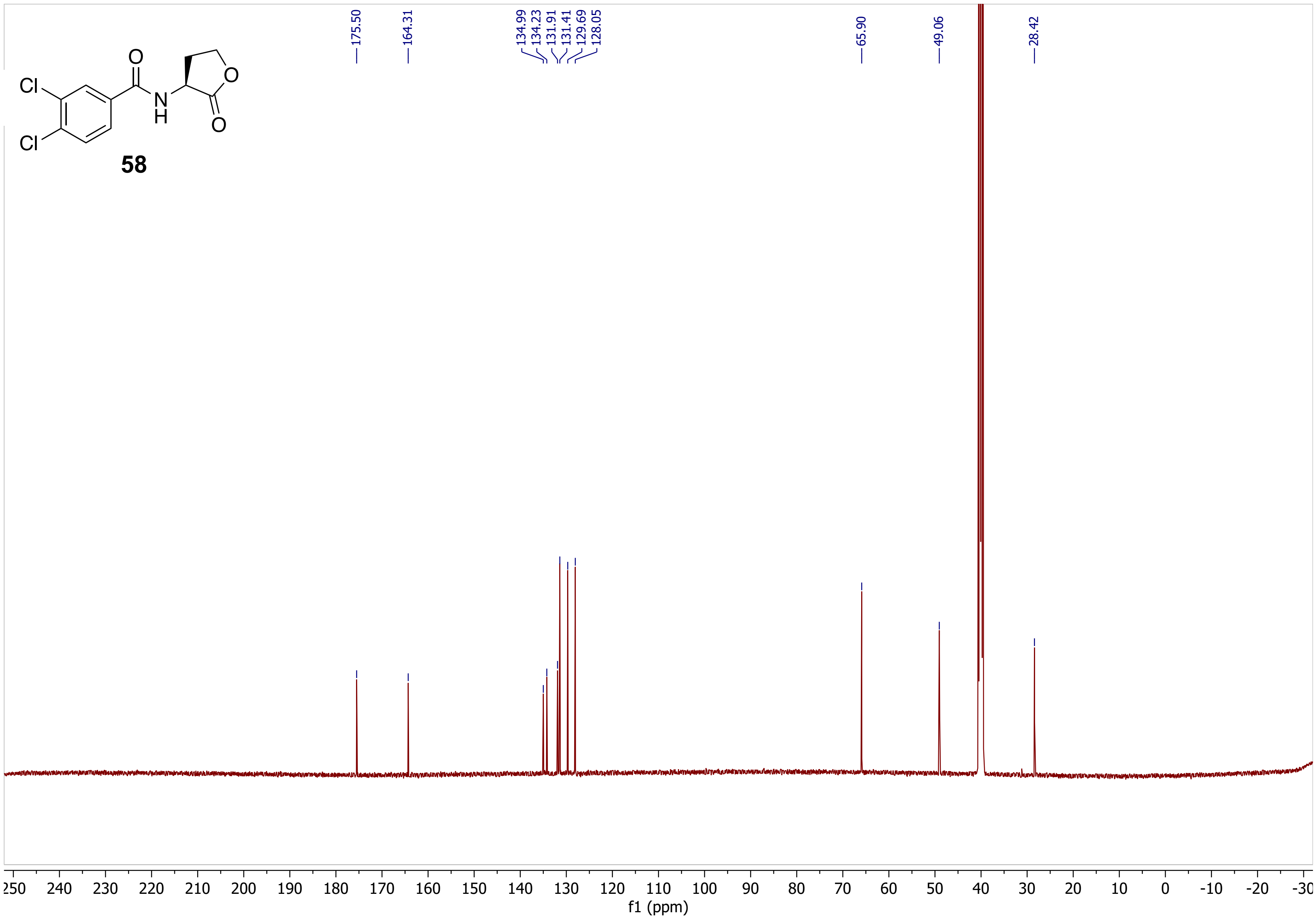
Cl
Cl
O
N
H
O
O
58
175.50
164.31
134.99
134.23
131.91
131.41
129.69
128.05
65.90
49.06
28.42
250 240 230 220 210 200 190 180 170 160 150 140 130 120 110 100 90 80 70 60 50 40 30 20 10 0 -10 -20 -30
f1 (ppm)

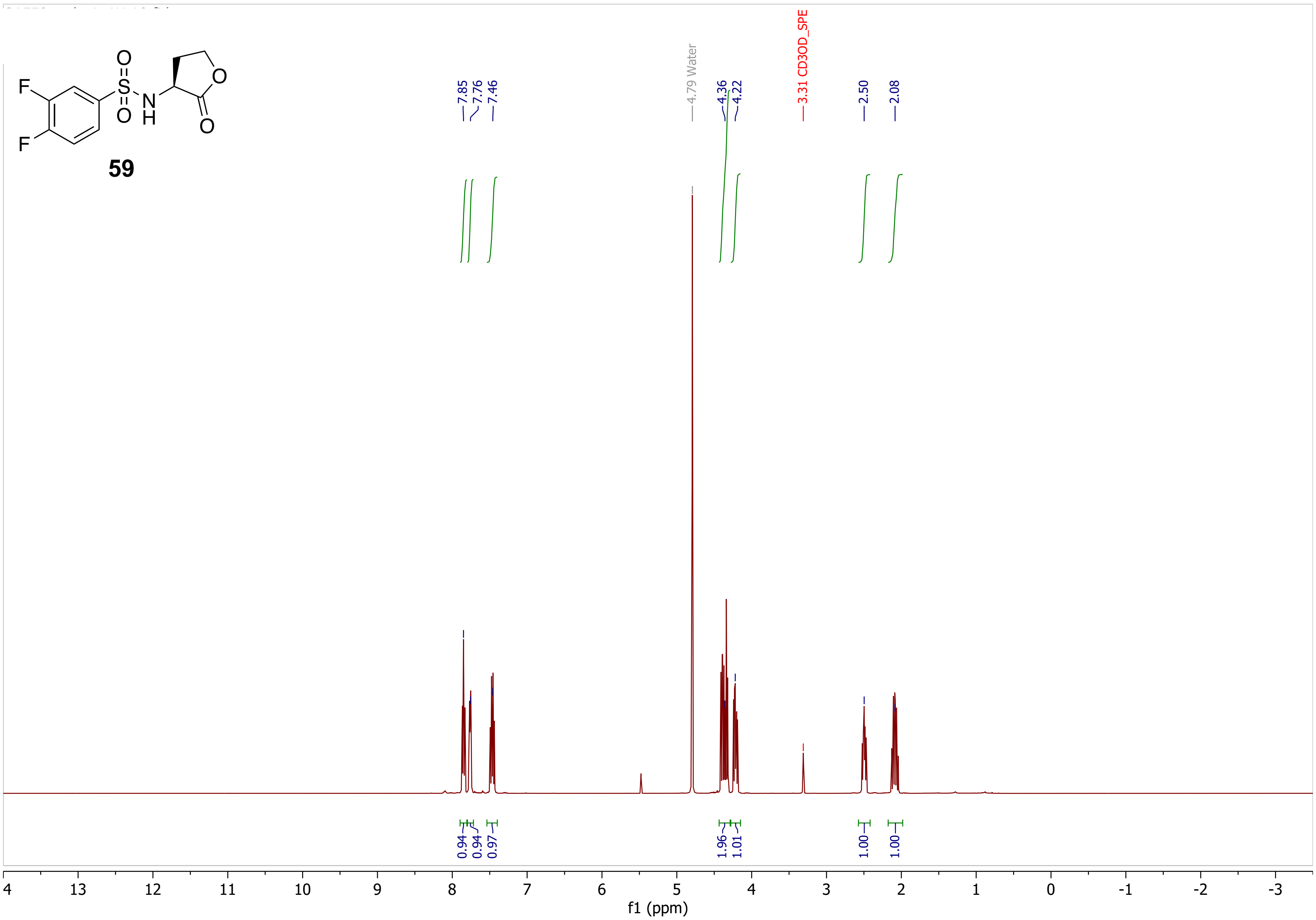
59
7.85
7.76
7.46
4.79 Water
4.36
4.22
3.31 CD3OD_SPE
2.50
2.08
0.94
0.94
0.97
1.96
1.01
1.00
1.00
f1 (ppm)

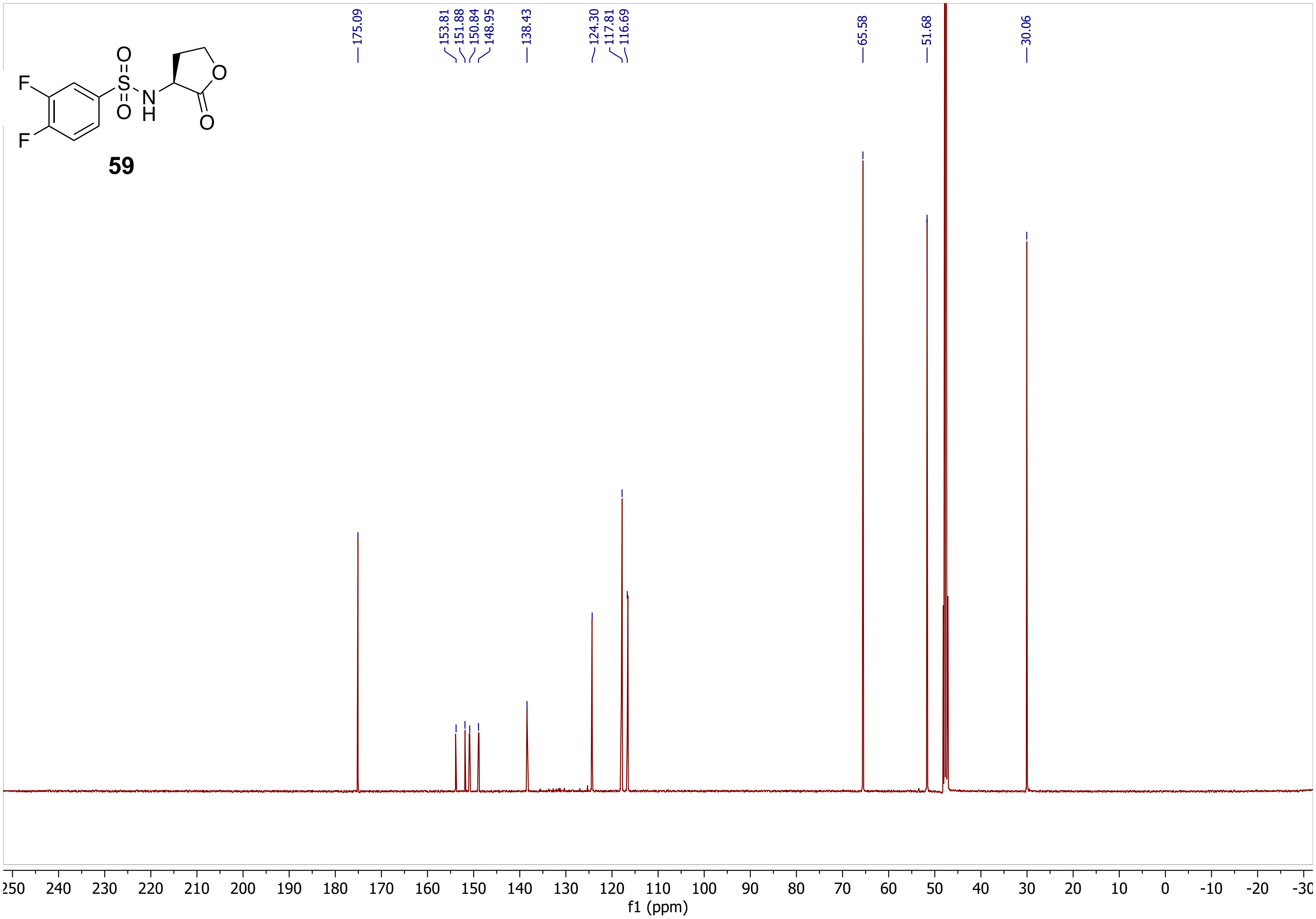
59
175.09
153.81
151.88
150.84
148.95
138.43
124.30
117.81
116.69
65.58
51.68
30.06
250 240 230 220 210 200 190 180 170 160 150 140 130 120 110 100 90 80 70 60 50 40 30 20 10 0 -10 -20 -30
f1 (ppm)

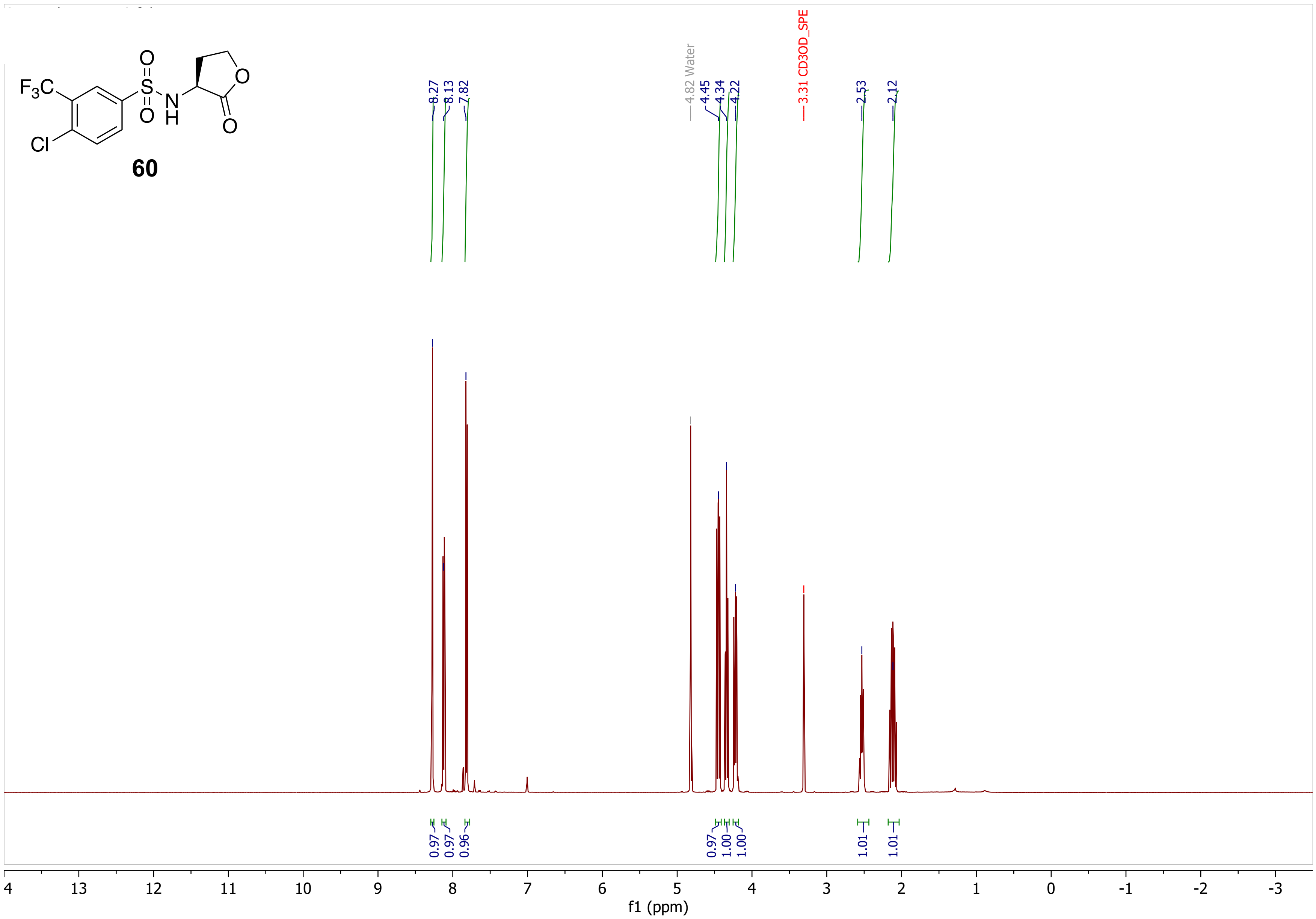
60
8.27
8.13
7.82
4.82 Water
4.45
4.34
4.22
3.31 CD3OD_SPE
2.53
2.12
0.97
0.97
0.96
0.97
1.00
1.00
1.01
1.01
f1 (ppm)

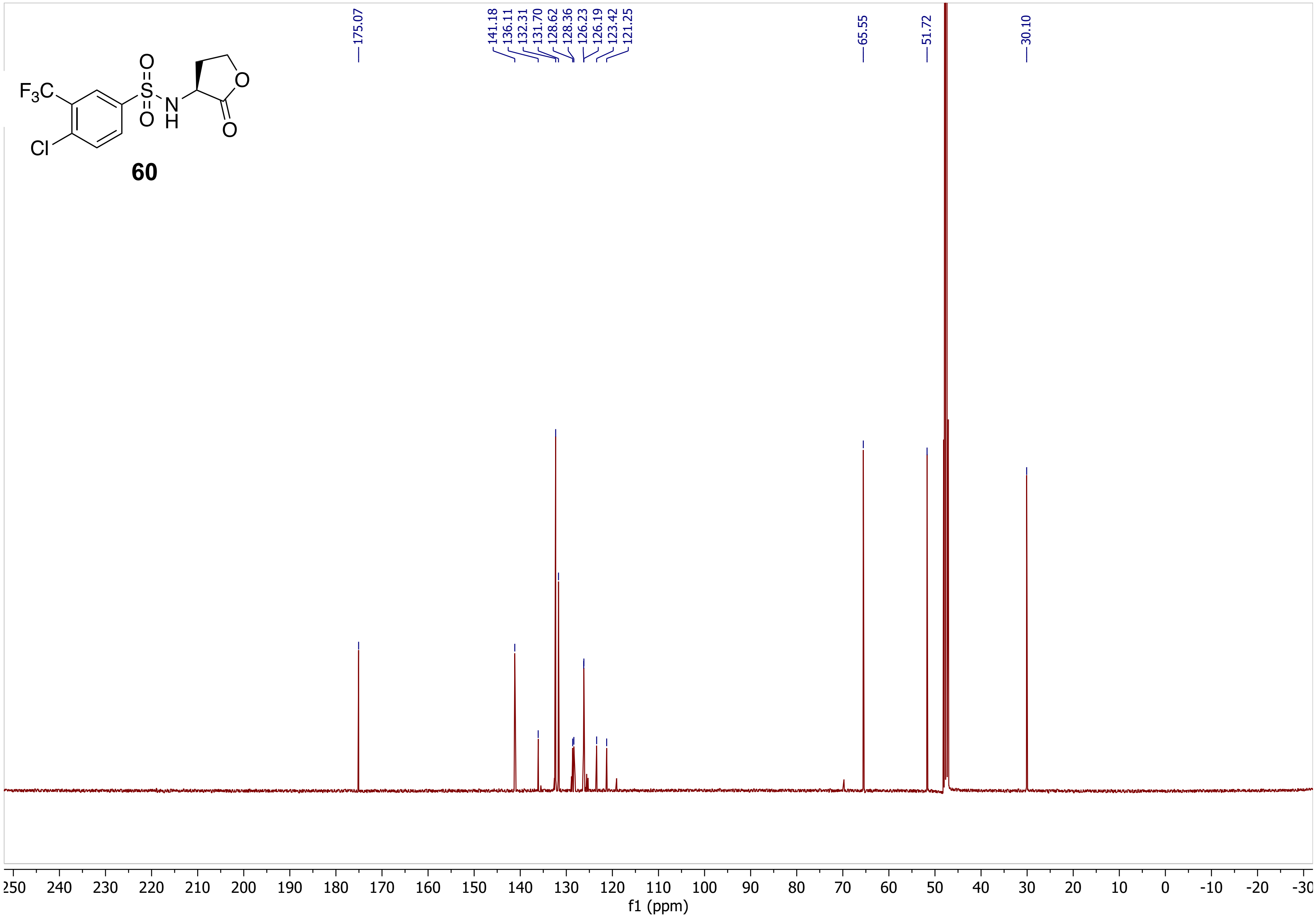
F3C
Cl
O
S
O
N
H
O
O
60
175.07
141.18
136.11
132.31
131.70
128.62
128.36
126.23
126.19
123.42
121.25
65.55
51.72
30.10
250 240 230 220 210 200 190 180 170 160 150 140 130 120 110 100 90 80 70 60 50 40 30 20 10 0 -10 -20 -30
f1 (ppm)

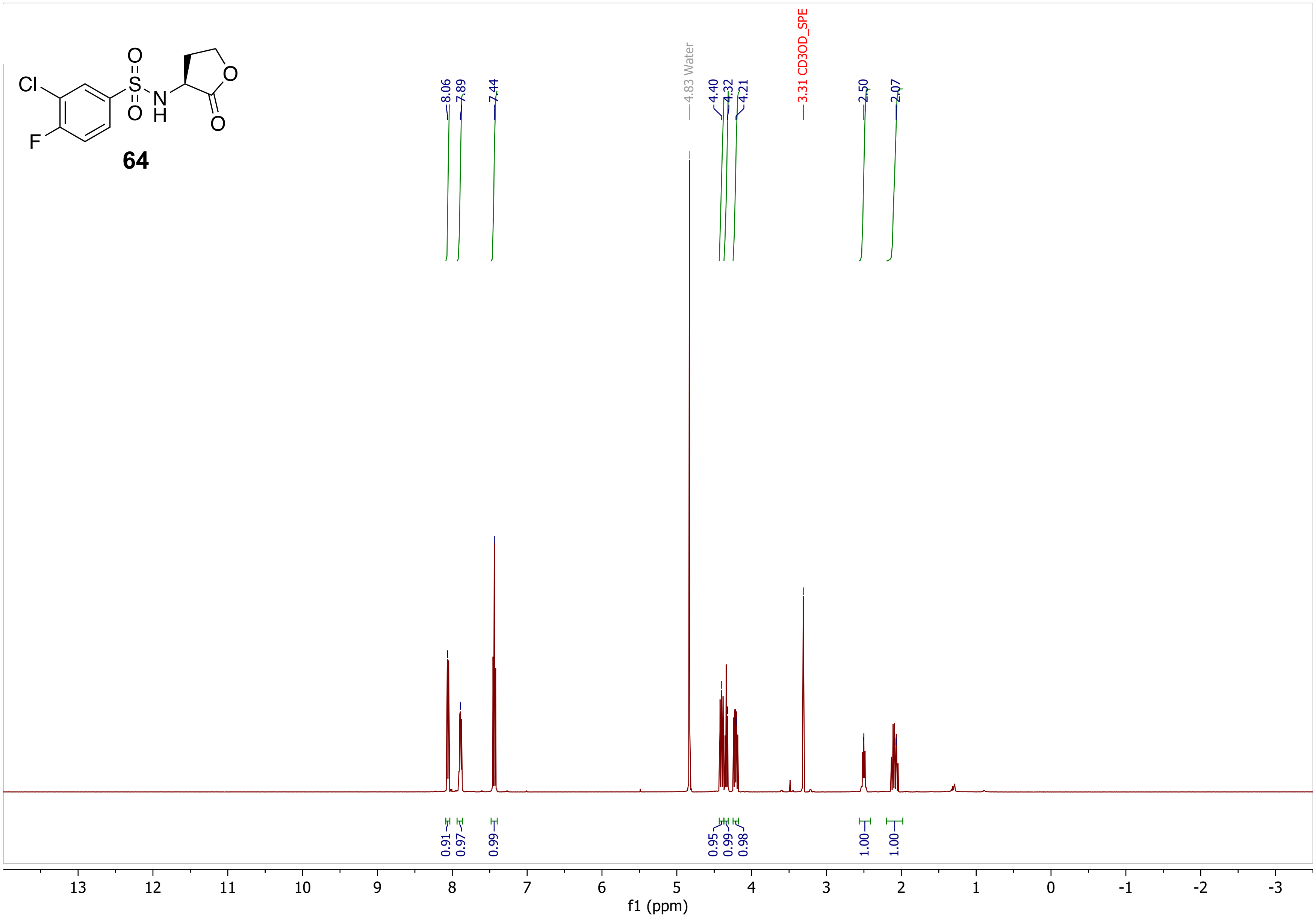
64
8.06
7.89
7.44
4.83 Water
4.40
4.32
4.21
3.31 CD3OD_SPE
2.50
2.07
0.91
0.97
0.99
0.95
0.99
0.98
1.00
1.00
f1 (ppm)

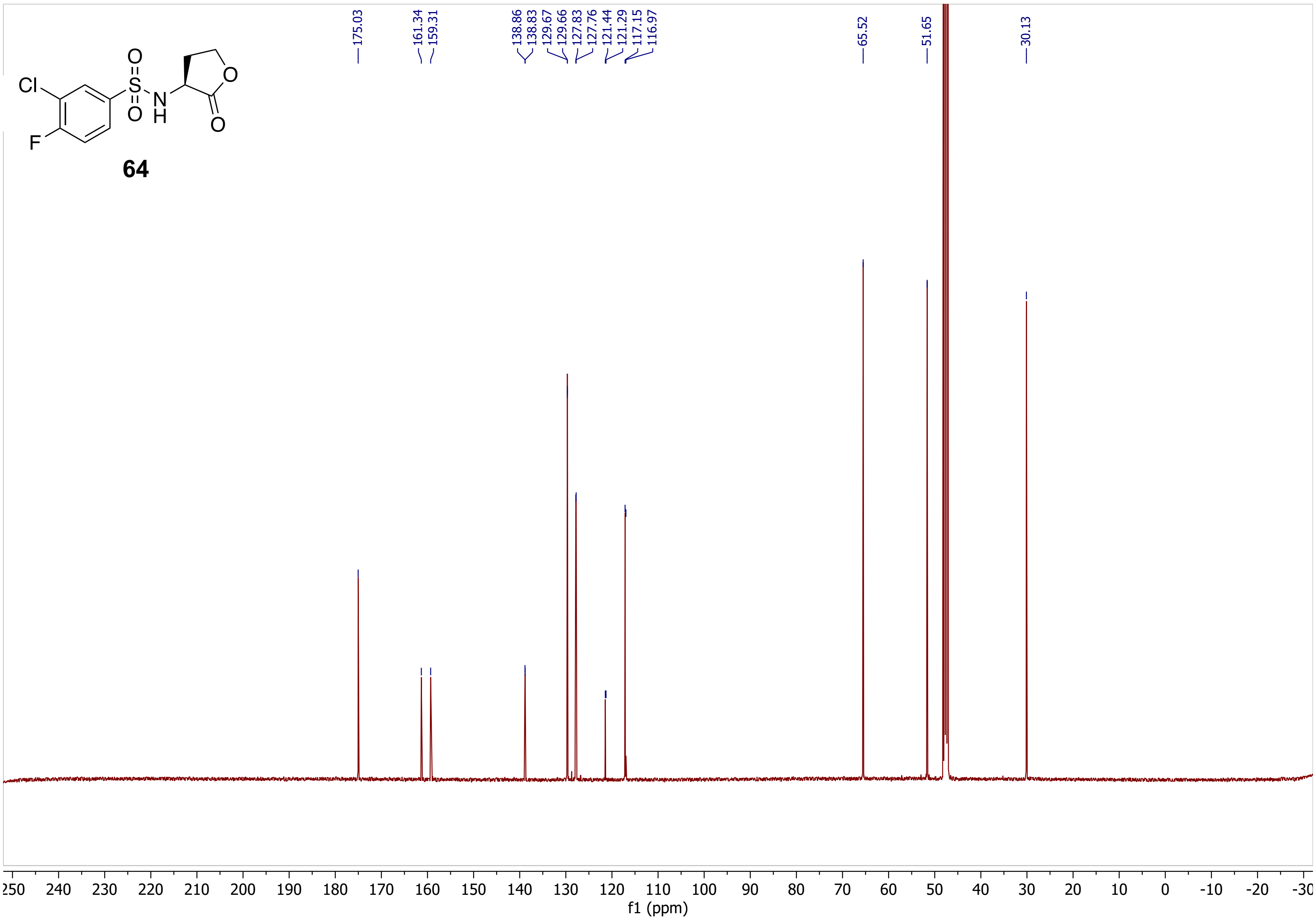

Cl
F
O
S
O
N
H
O
O
64
175.03
161.34
159.31
138.86
138.83
129.67
129.66
127.83
127.76
121.44
121.29
117.15
116.97
65.52
51.65
30.13
250 240 230 220 210 200 190 180 170 160 150 140 130 120 110 100 90 80 70 60 50 40 30 20 10 0 -10 -20 -30
f1 (ppm)

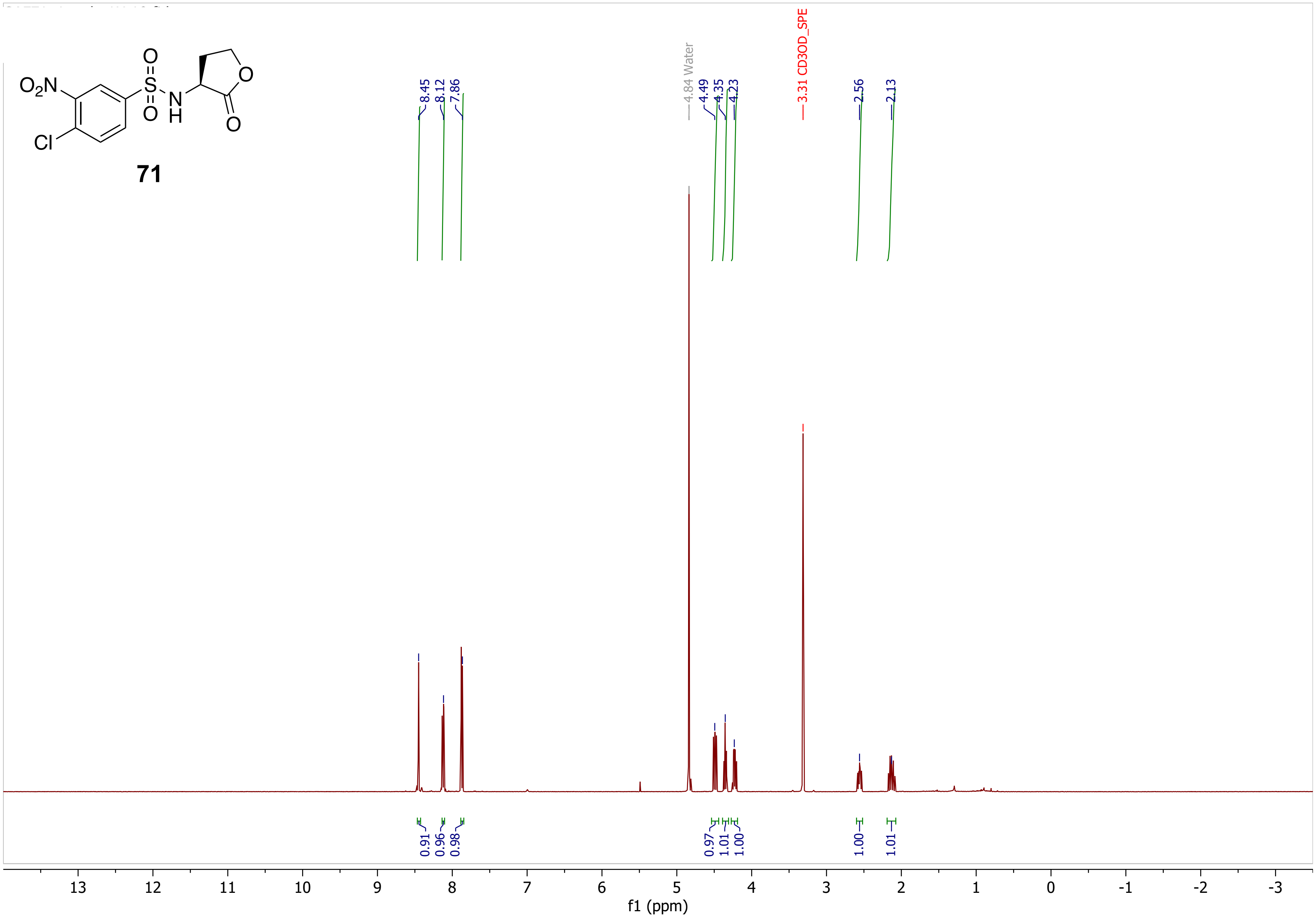
O2N
Cl
O
S
O
N
H
O
O
71
8.45
8.12
7.86
4.84 Water
4.49
4.35
4.23
3.31 CD3OD_SPE
2.56
2.13
0.91
0.96
0.98
0.97
1.01
1.00
1.00
1.01
13
12
11
10
9
8
7
6
5
4
3
2
1
0
-1
-2
-3
f1 (ppm)

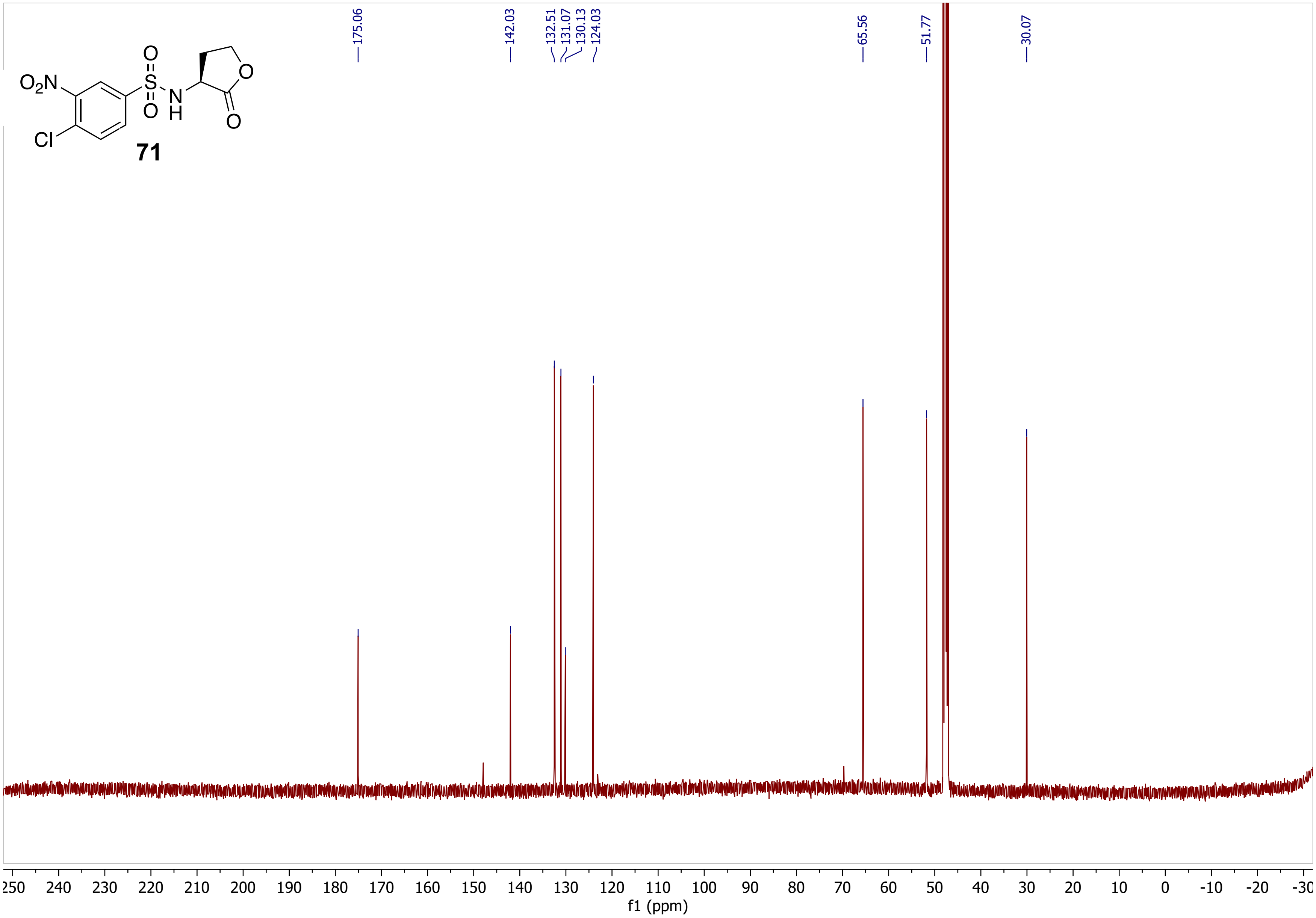
71
175.06
142.03
132.51
131.07
130.13
124.03
65.56
51.77
30.07
250
240
230
220
210
200
190
180
170
160
150
140
130
120
110
100
90
80
70
60
50
40
30
20
10
0
-10
-20
-30
f1 (ppm)

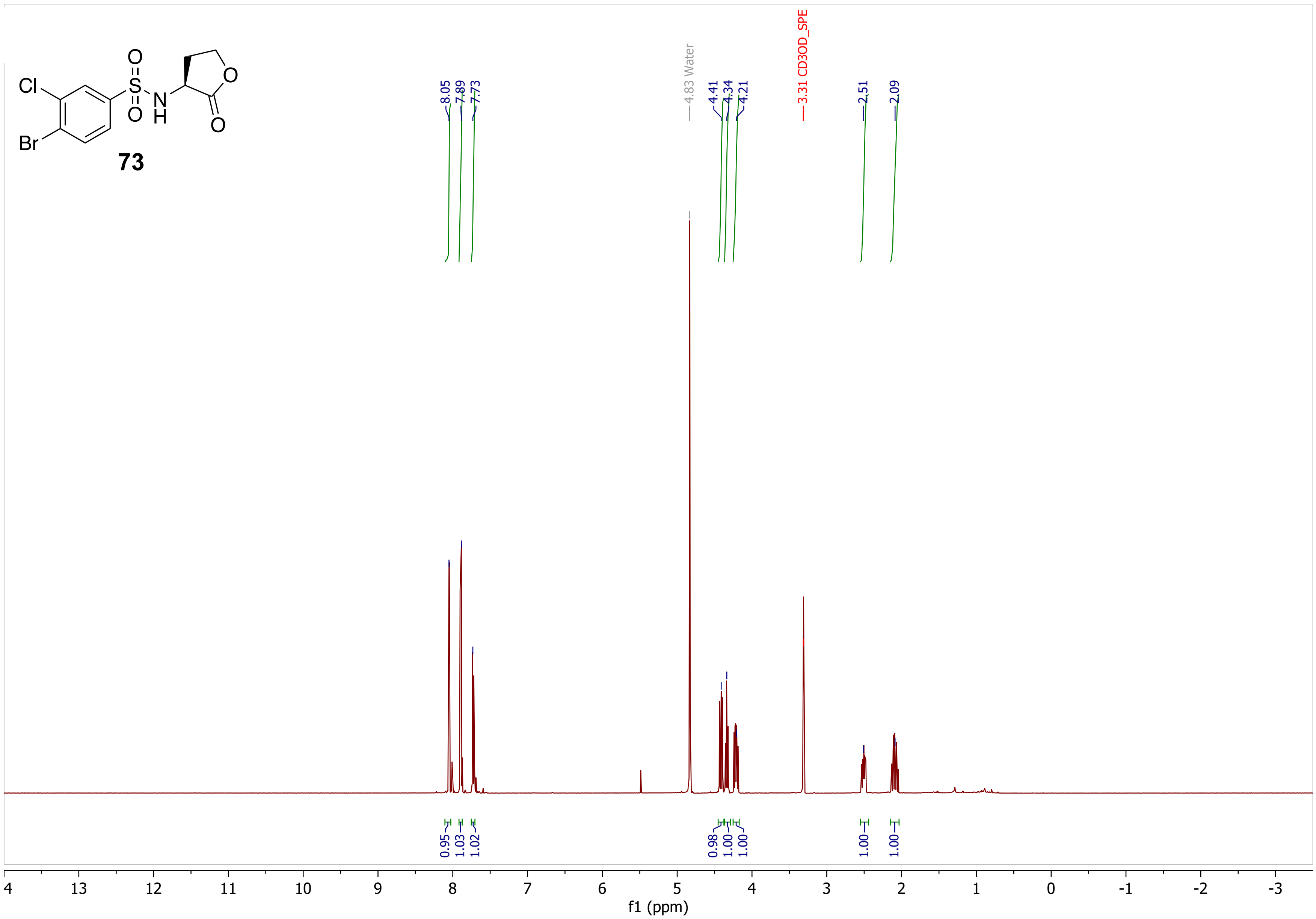
Cl
Br
O
S
O
N
H
O
O
73
8.05
7.89
7.73
4.83 Water
4.41
4.34
4.21
3.31 CD3OD_SPE
2.51
2.09
0.95
1.03
1.02
0.98
1.00
1.00
1.00
1.00
14
13
12
11
10
9
8
7
6
5
4
3
2
1
0
-1
-2
-3
f1 (ppm)

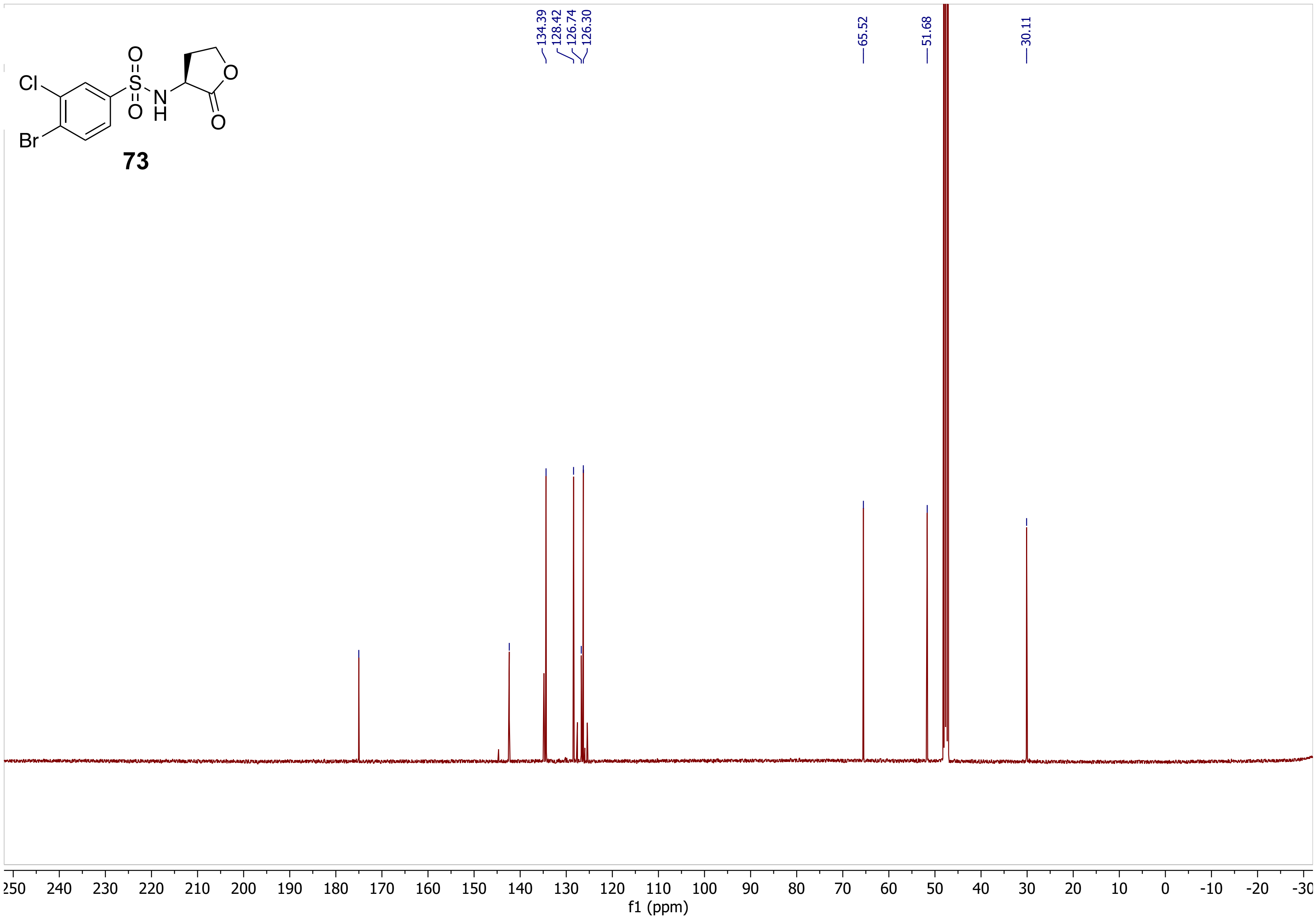
Cl
Br
O
S
O
N
H
O
O
73
134.39
128.42
126.74
126.30
65.52
51.68
30.11
250 240 230 220 210 200 190 180 170 160 150 140 130 120 110 100 90 80 70 60 50 40 30 20 10 0 -10 -20 -30
f1 (ppm)

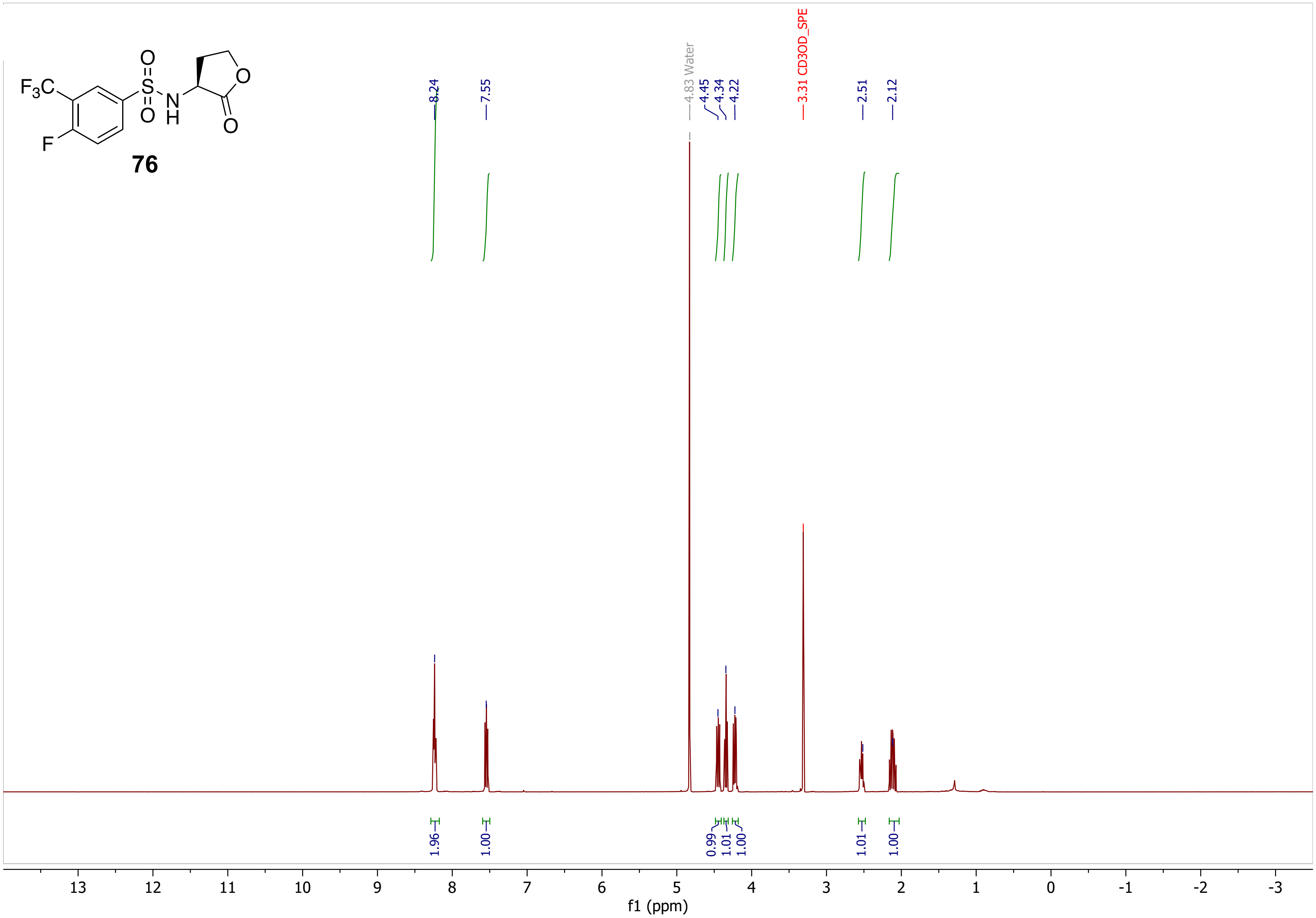
F3C
F
O
S
O
N
H
O
O
76
8.24
7.55
4.83 Water
4.45
4.34
4.22
3.31 CD3OD_SPE
2.51
2.12
1.96
1.00
0.99
1.01
1.00
1.01
1.00
13
12
11
10
9
8
7
6
5
4
3
2
1
0
-1
-2
-3
f1 (ppm)

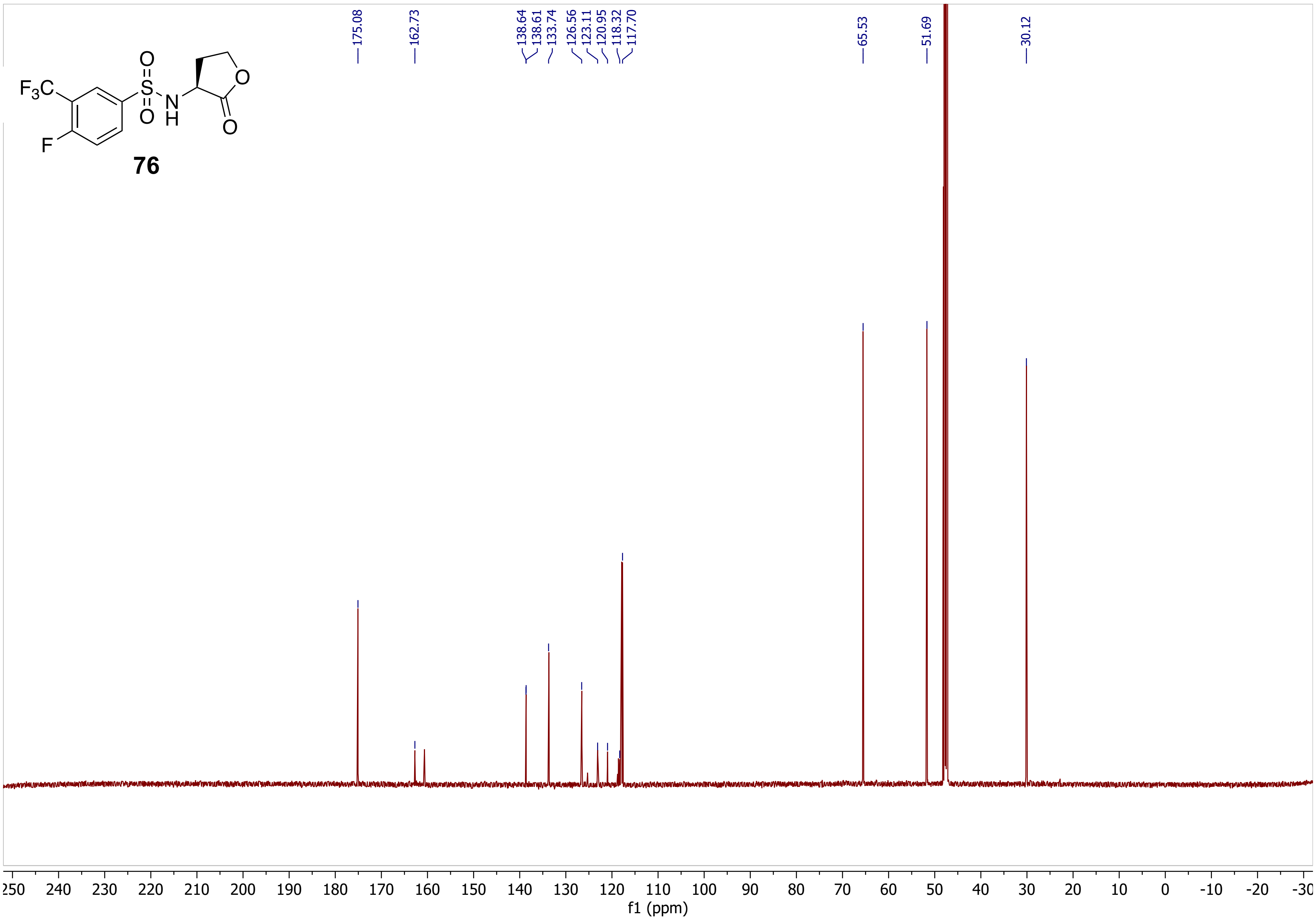
F3C
F
O
S
O
N
H
O
O
76
175.08
162.73
138.64
138.61
133.74
126.56
123.11
120.95
118.32
117.70
65.53
51.69
30.12
250 240 230 220 210 200 190 180 170 160 150 140 130 120 110 100 90 80 70 60 50 40 30 20 10 0 -10 -20 -30
f1 (ppm)

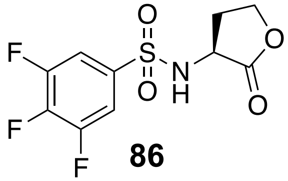

86

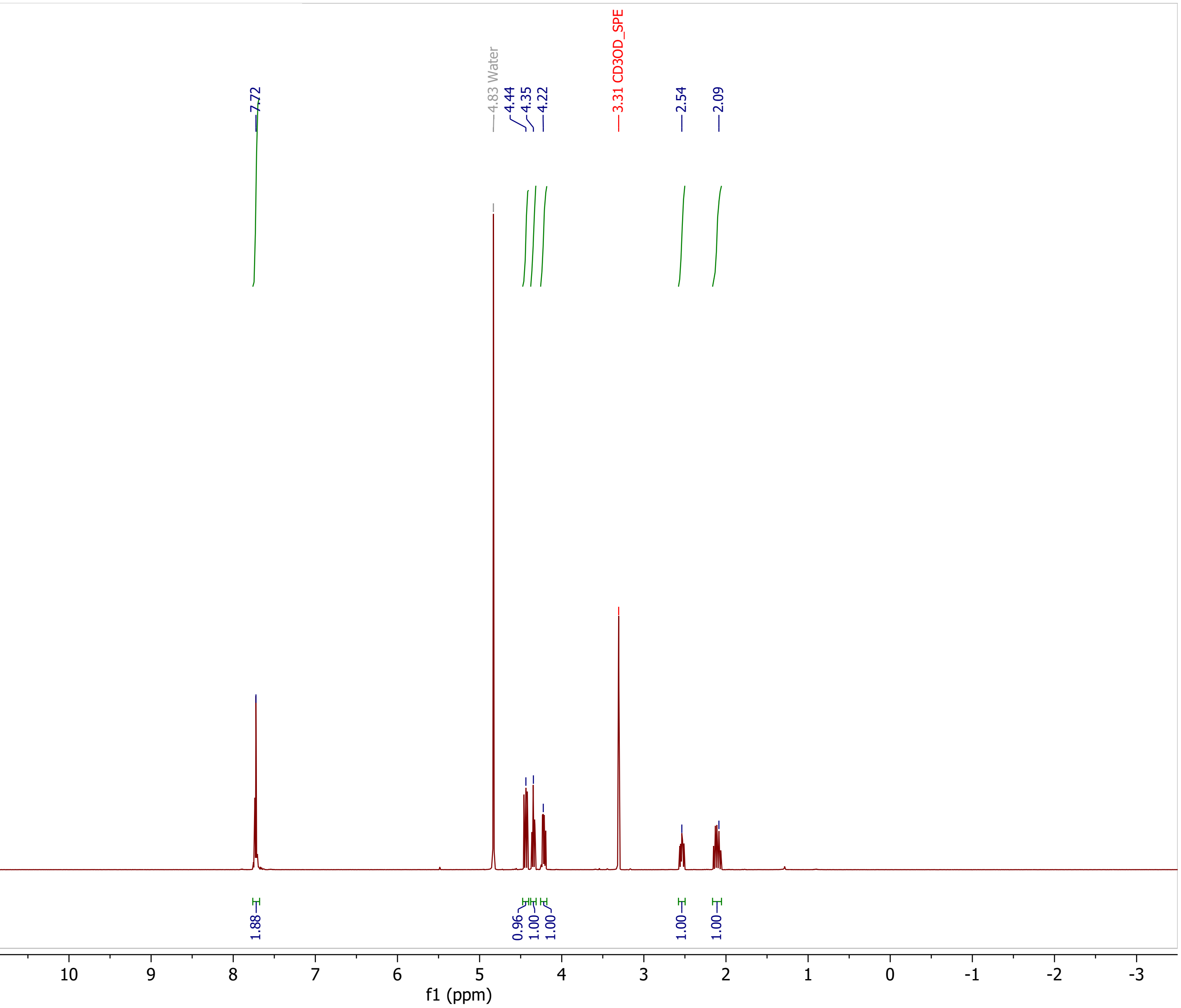
7.72
4.83 Water
4.44
4.35
4.22
3.31 CD3OD_SPE
2.54
2.09
1.88
0.96
1.00
1.00
1.00
1.00
13
12
11
10
9
8
7
6
5
4
3
2
1
0
-1
-2
-3
f1 (ppm)

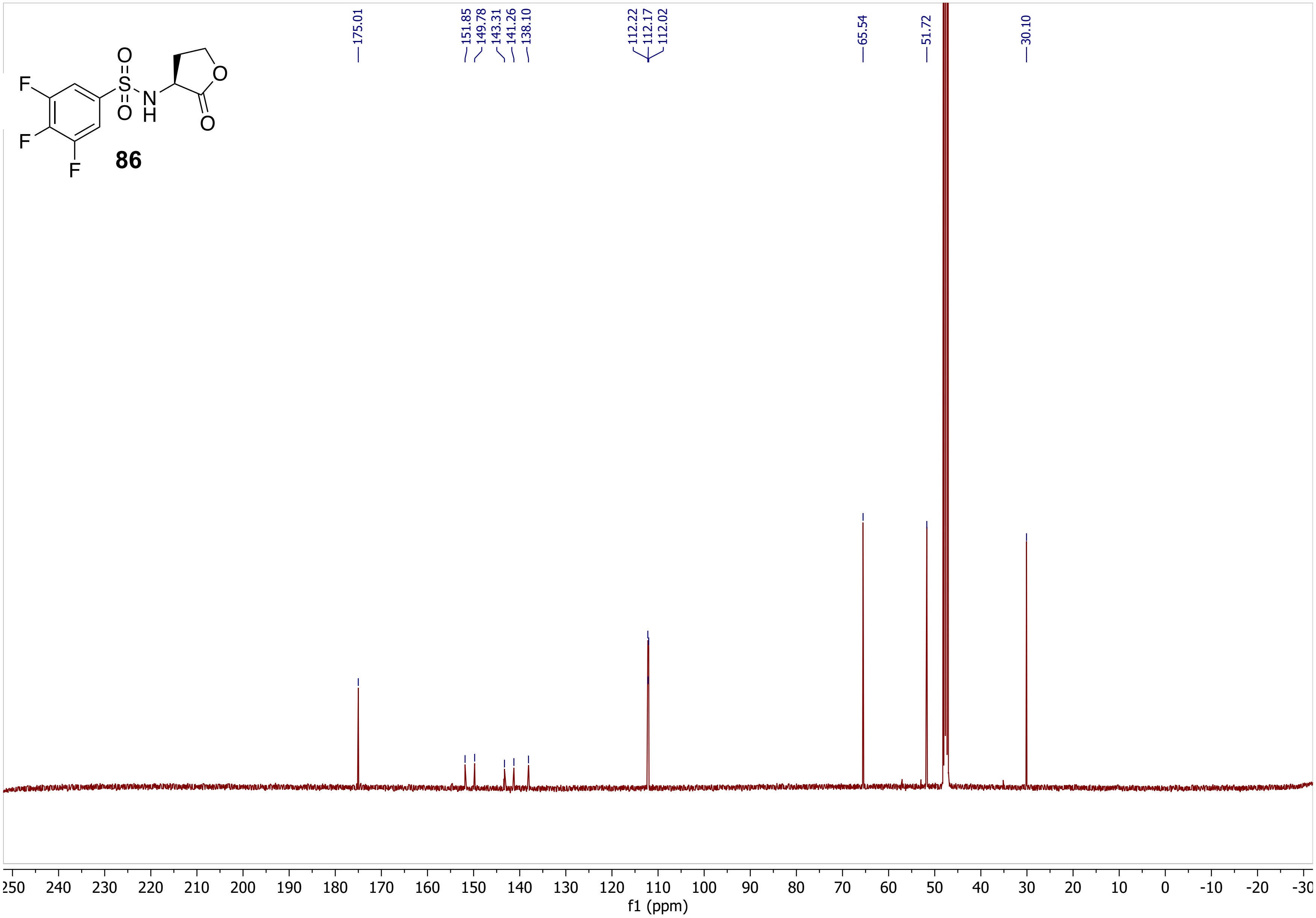
86
175.01
151.85
149.78
143.31
141.26
138.10
112.22
112.17
112.02
65.54
51.72
30.10
250 240 230 220 210 200 190 180 170 160 150 140 130 120 110 100 90 80 70 60 50 40 30 20 10 0 -10 -20 -30
f1 (ppm)

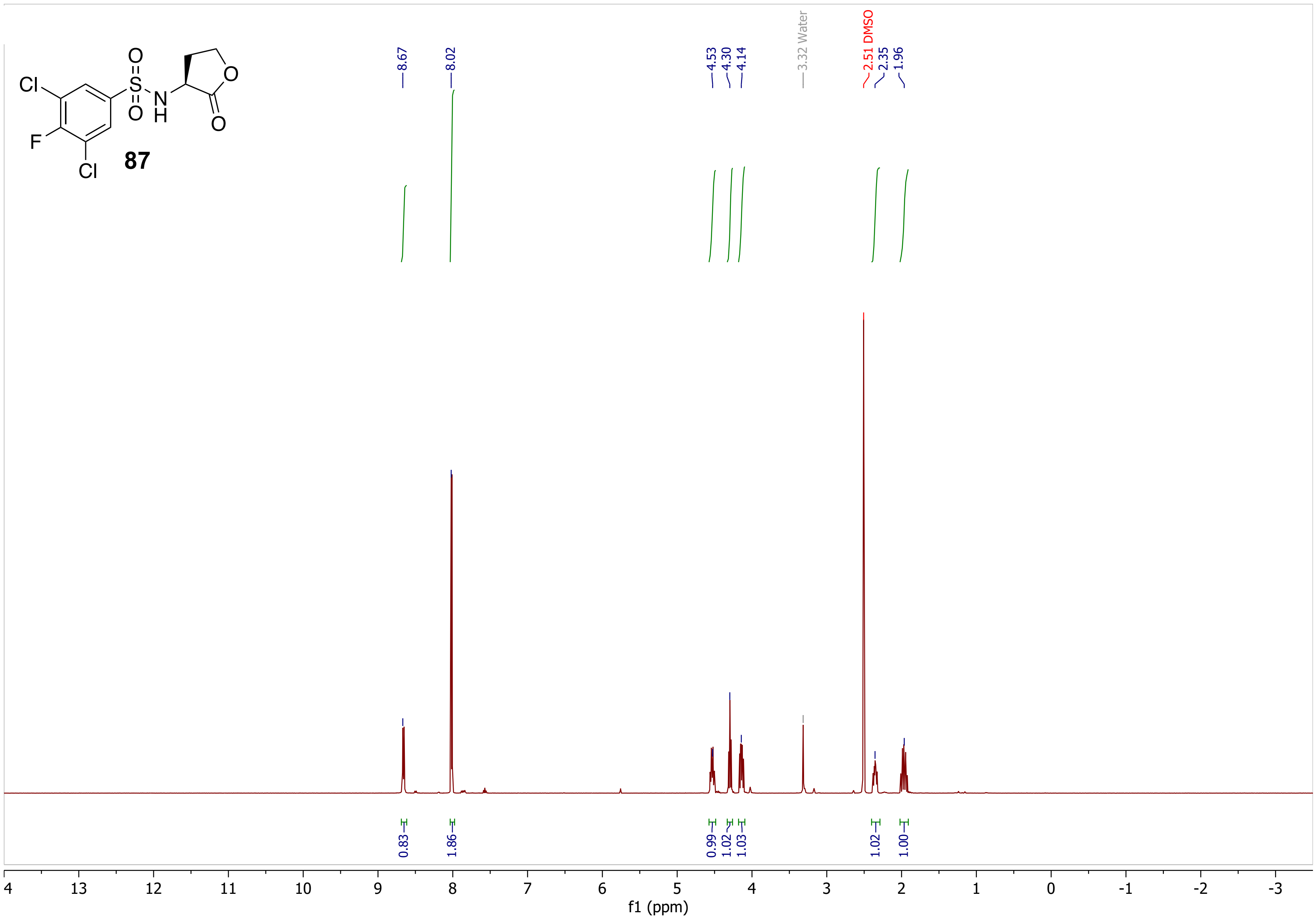
Cl
F
Cl
O
S
O
N
H
O
O
87
8.67
8.02
4.53
4.30
4.14
3.32 Water
2.51 DMSO
2.35
1.96
0.83
1.86
0.99
1.02
1.03
1.02
1.00
14
13
12
11
10
9
8
7
6
5
4
3
2
1
0
-1
-2
-3
f1 (ppm)

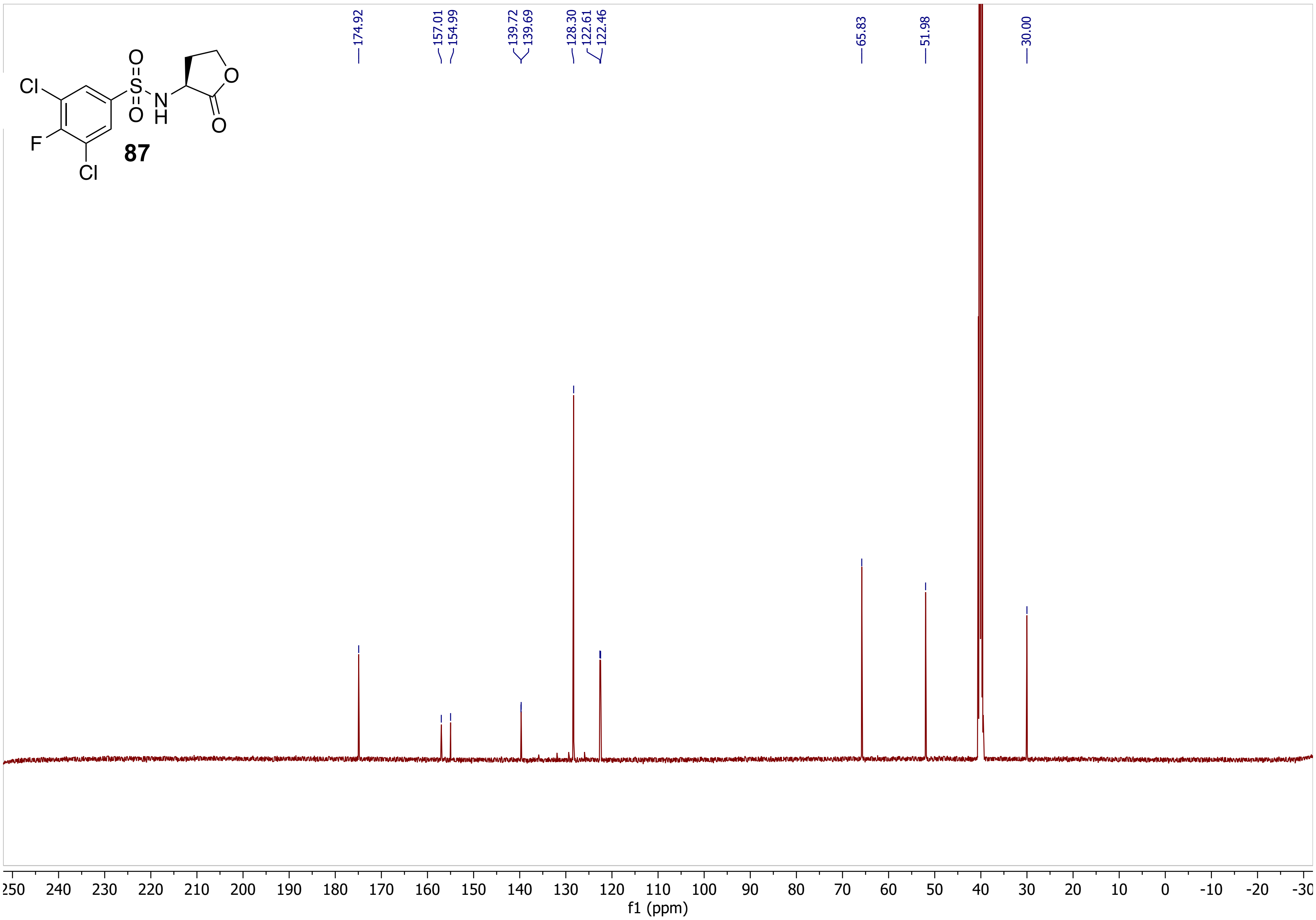
Cl
F
Cl
O
S
O
N
H
O
O
87
174.92
157.01
154.99
139.72
139.69
128.30
122.61
122.46
65.83
51.98
30.00
250
240
230
220
210
200
190
180
170
160
150
140
130
120
110
100
90
80
70
60
50
40
30
20
10
0
-10
-20
-30
f1 (ppm)

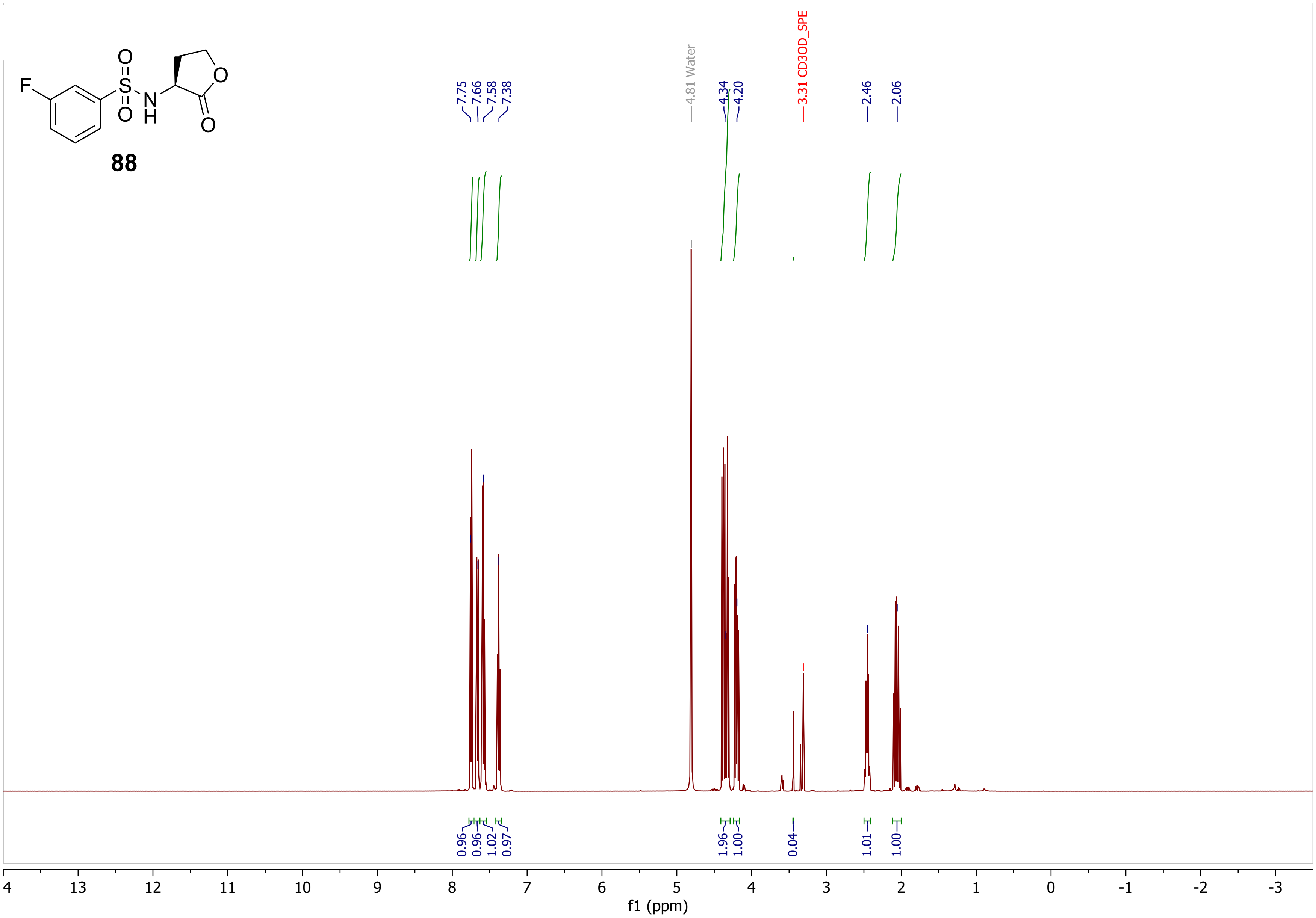

F
O
S
O
N
H
O
O
88
7.75
7.66
7.58
7.38
4.81 Water
4.34
4.20
3.31 CD3OD_SPE
2.46
2.06
0.96
0.96
1.02
0.97
1.96
1.00
0.04
1.01
1.00
4
13
12
11
10
9
8
7
6
5
4
3
2
1
0
-1
-2
-3
f1 (ppm)

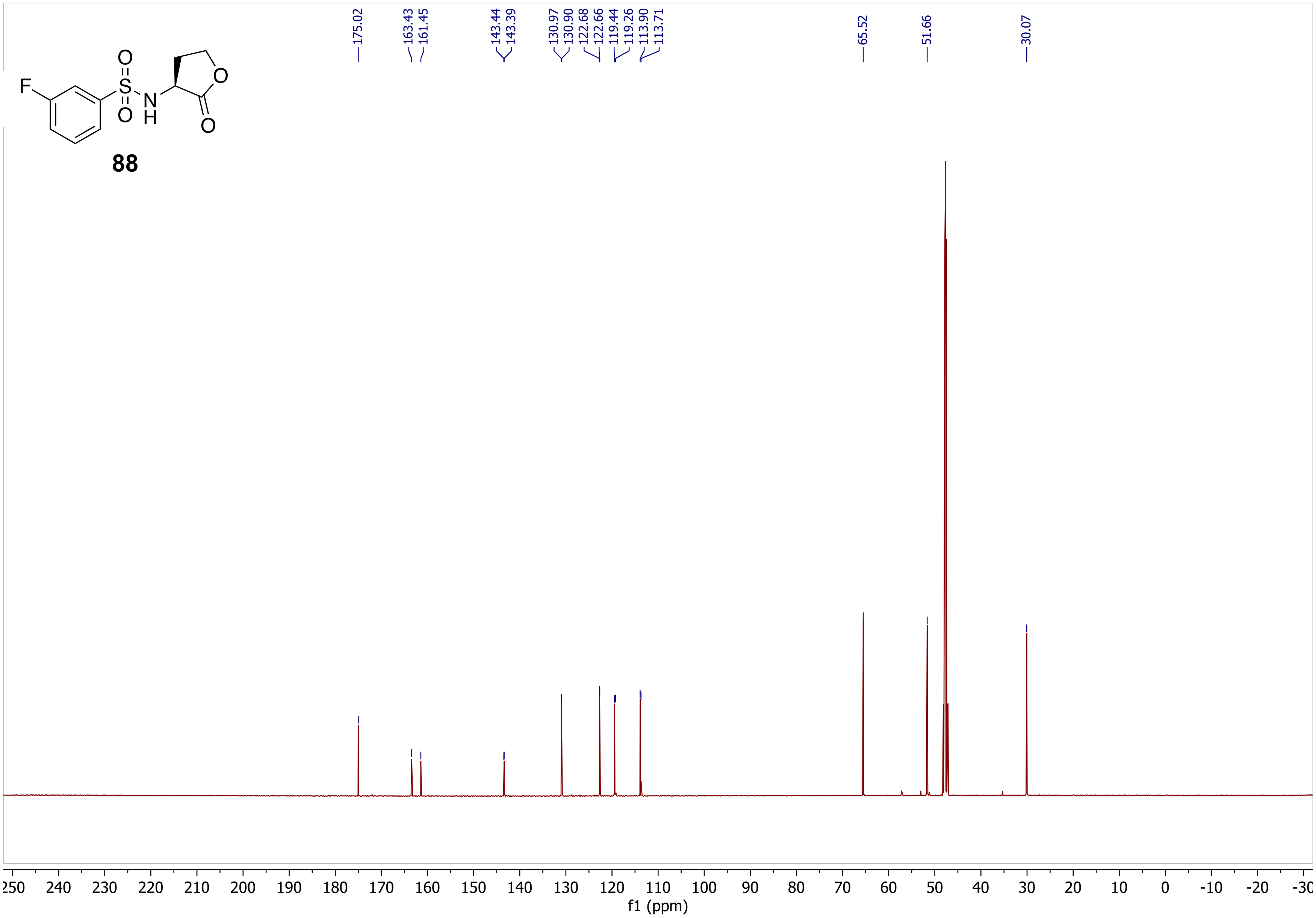
175.02
163.43
161.45
143.44
143.39
130.97
130.90
122.68
122.66
119.44
119.26
113.90
113.71
65.52
51.66
30.07
F
O
S
O
N
H
O
O
88
250 240 230 220 210 200 190 180 170 160 150 140 130 120 110 100 90 80 70 60 50 40 30 20 10 0 -10 -20 -30
f1 (ppm)

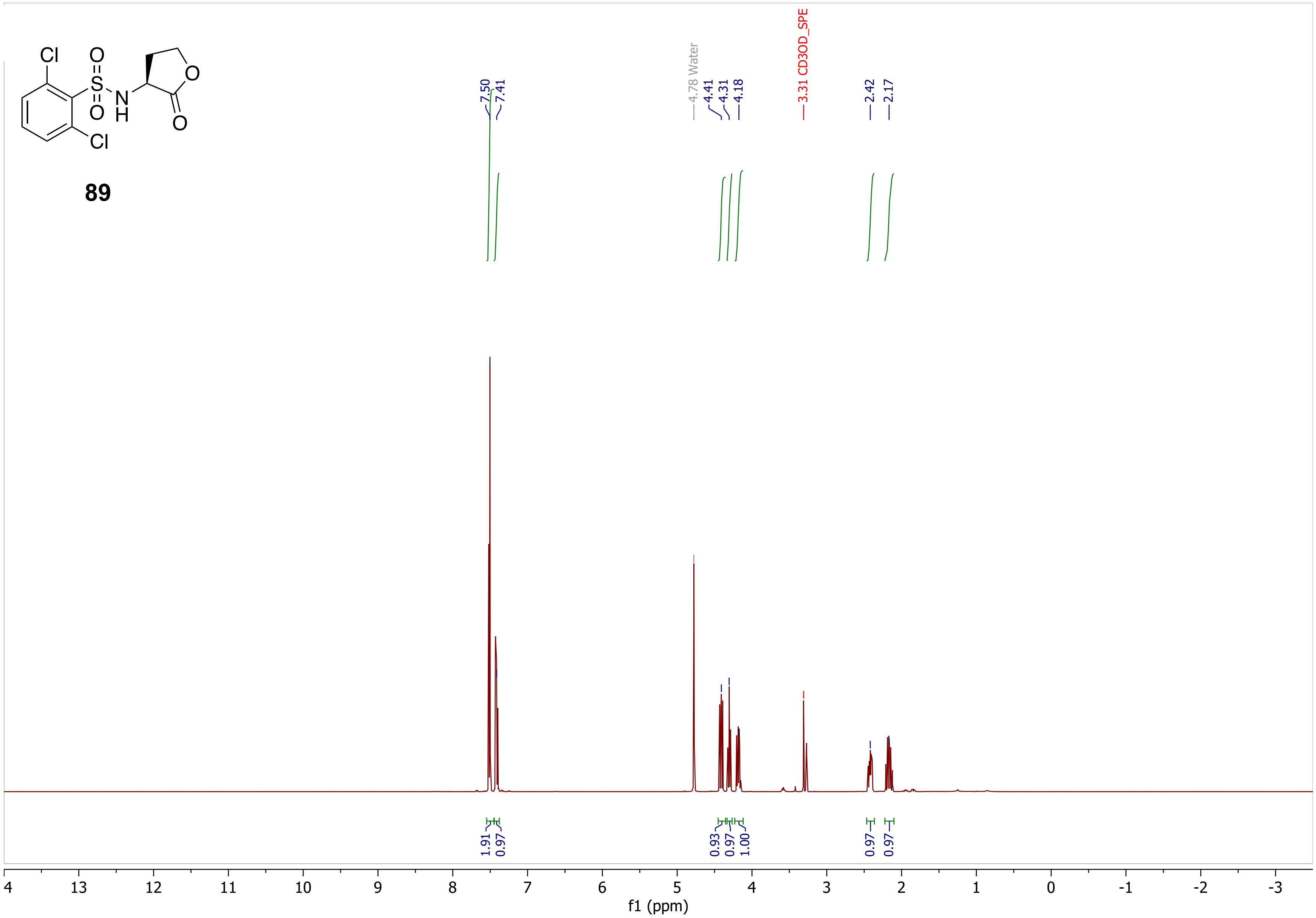
Cl
O
S
N
H
O
O
Cl
O
89
7.50
7.41
4.78 Water
4.41
4.31
4.18
3.31 CD3OD_SPE
2.42
2.17
1.91
0.97
0.93
0.97
1.00
0.97
0.97
4
13
12
11
10
9
8
7
6
5
4
3
2
1
0
-1
-2
-3
f1 (ppm)

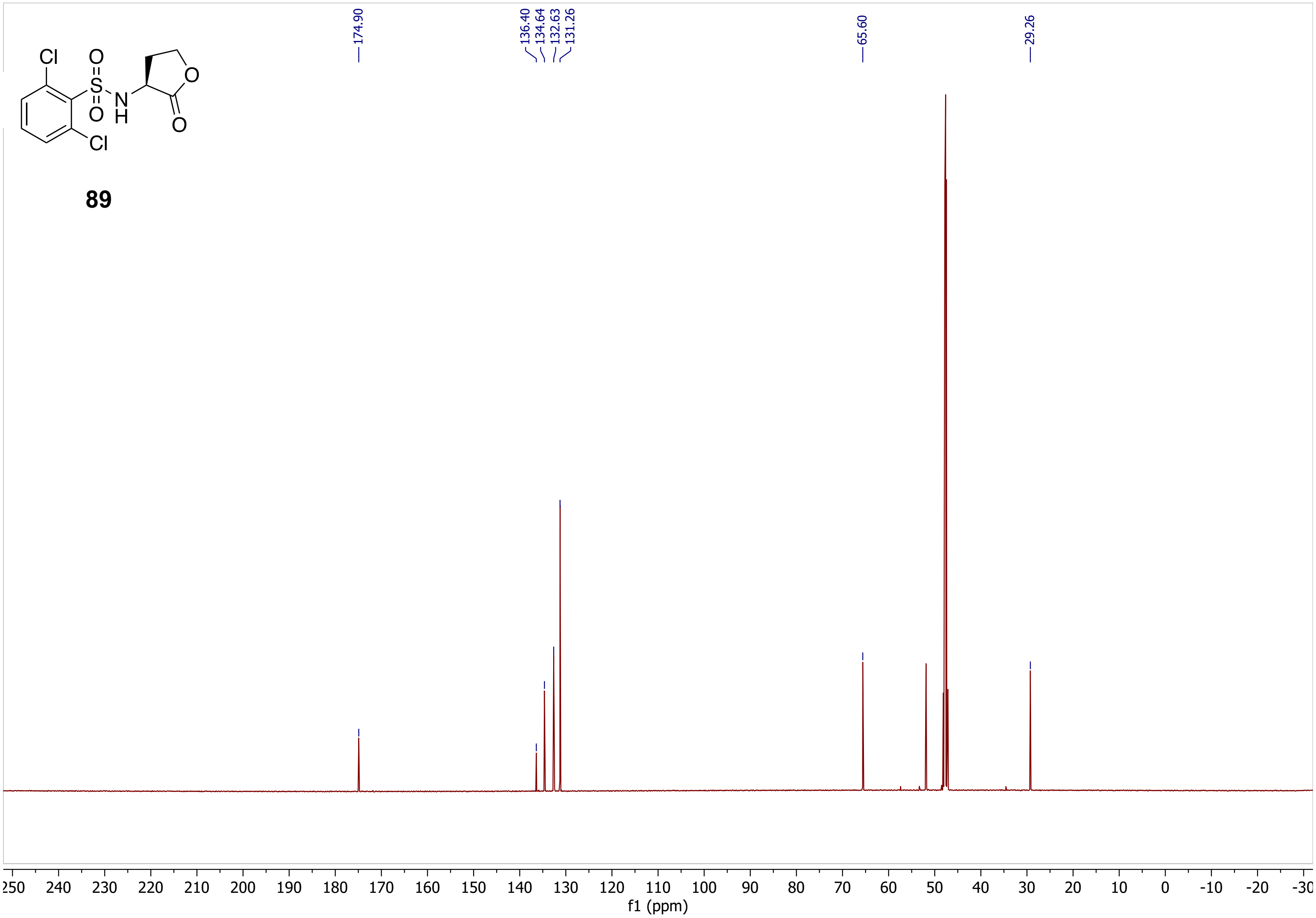
174.90
136.40
134.64
132.63
131.26
65.60
29.26
Cl
O
S
N
H
O
O
O
Cl
89
250 240 230 220 210 200 190 180 170 160 150 140 130 120 110 100 90 80 70 60 50 40 30 20 10 0 -10 -20 -30
f1 (ppm)

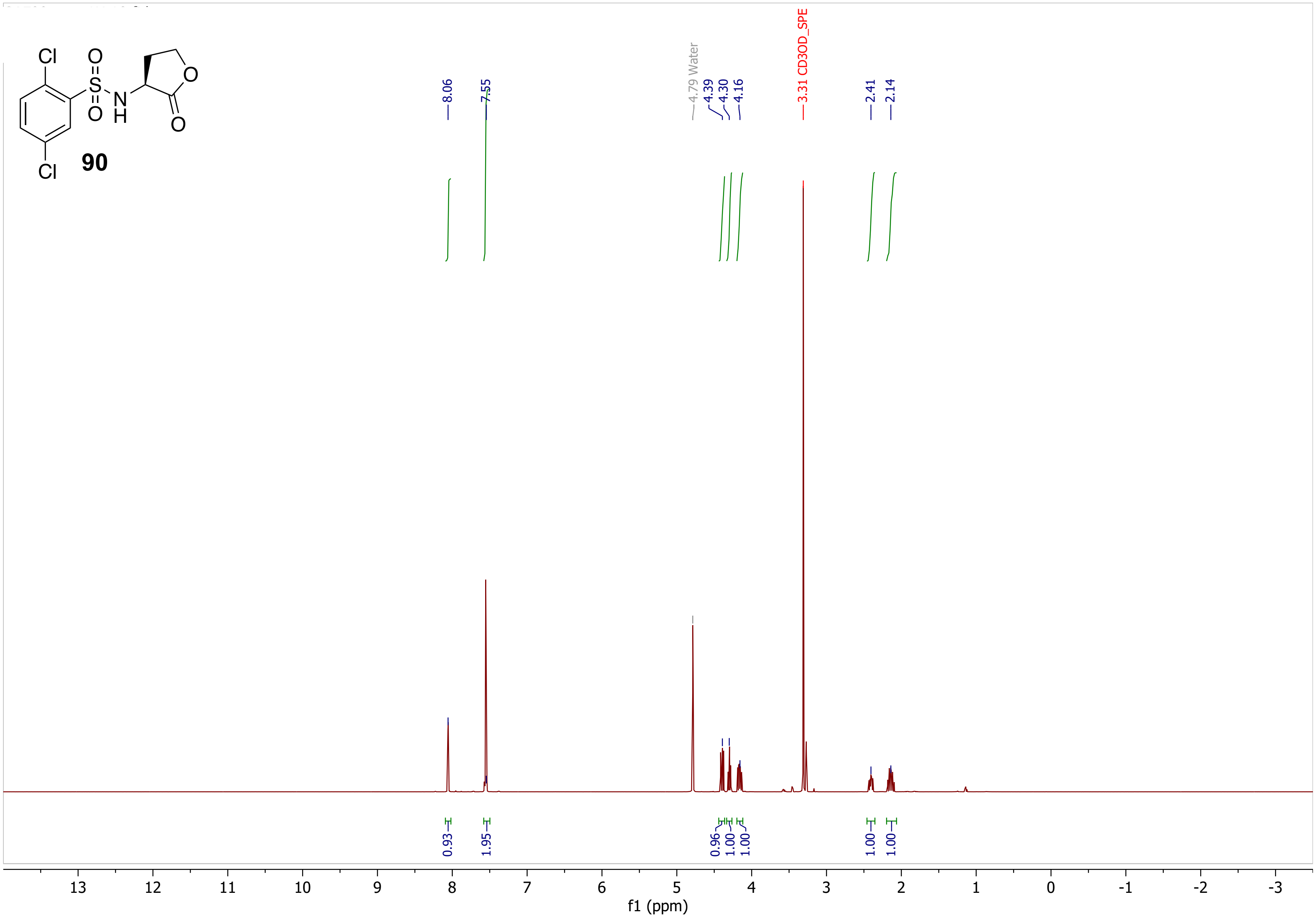

Cl
O
S
O
N
H
O
O
Cl
90
8.06
7.55
4.79 Water
4.39
4.30
4.16
3.31 CD3OD_SPE
2.41
2.14
0.93
1.95
0.96
1.00
1.00
1.00
1.00
13
12
11
10
9
8
7
6
5
4
3
2
1
0
-1
-2
-3
f1 (ppm)

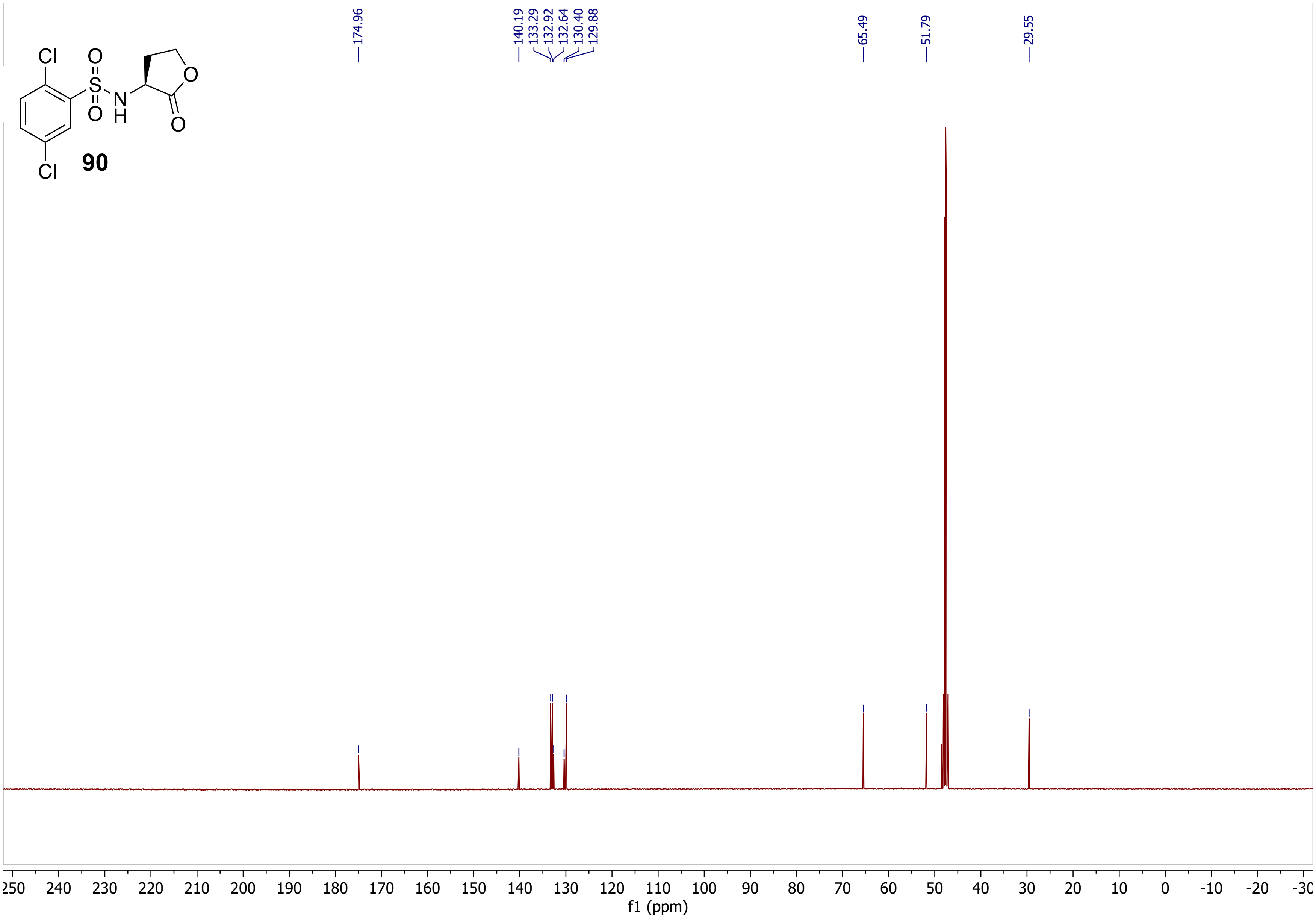
90
174.96
140.19
133.29
132.92
132.64
130.40
129.88
65.49
51.79
29.55
f1 (ppm)

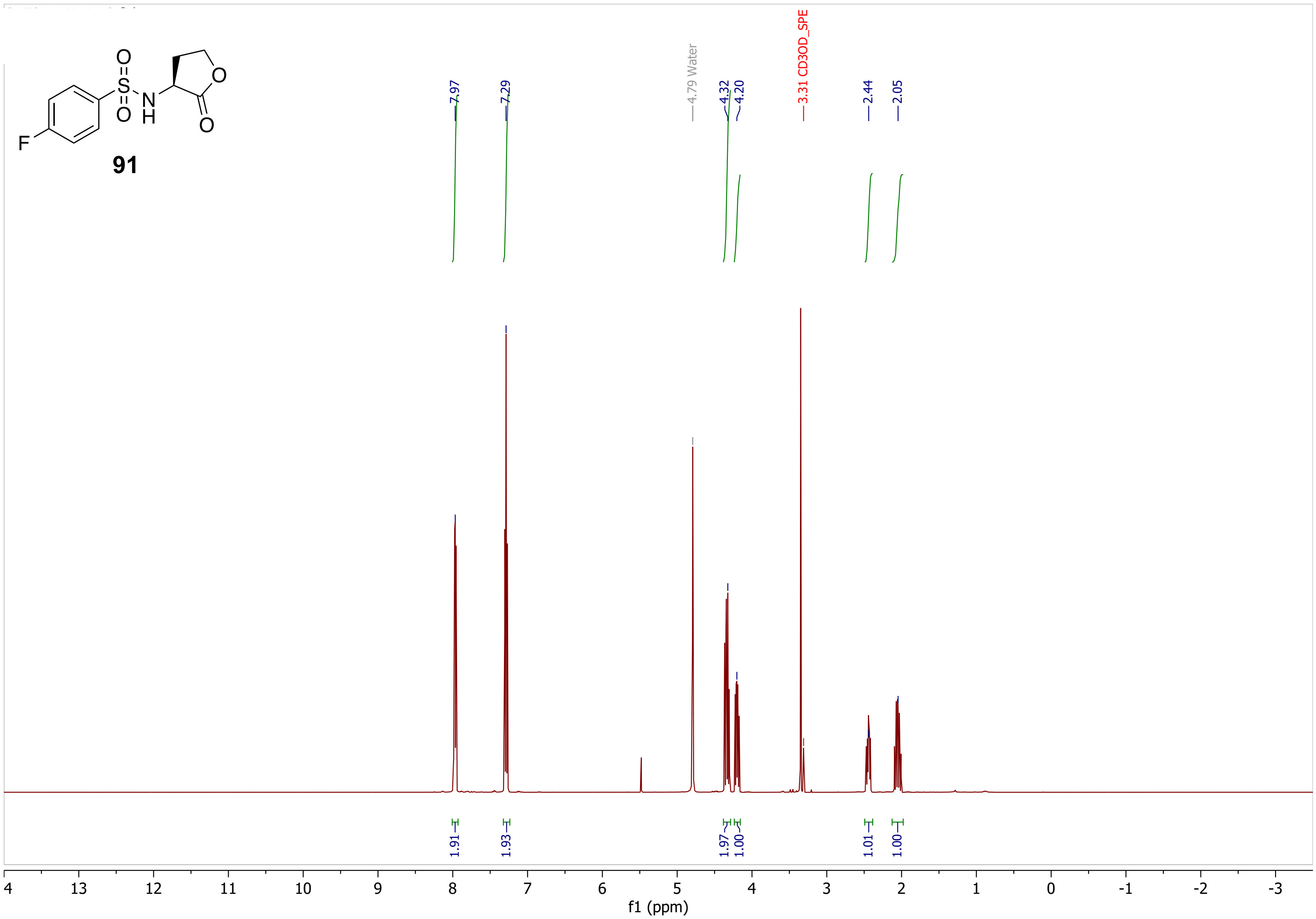
91
7.97
7.29
4.79 Water
4.32
4.20
3.31 CD3OD_SPE
2.44
2.05
1.91
1.93
1.97
1.00
1.01
1.00
f1 (ppm)

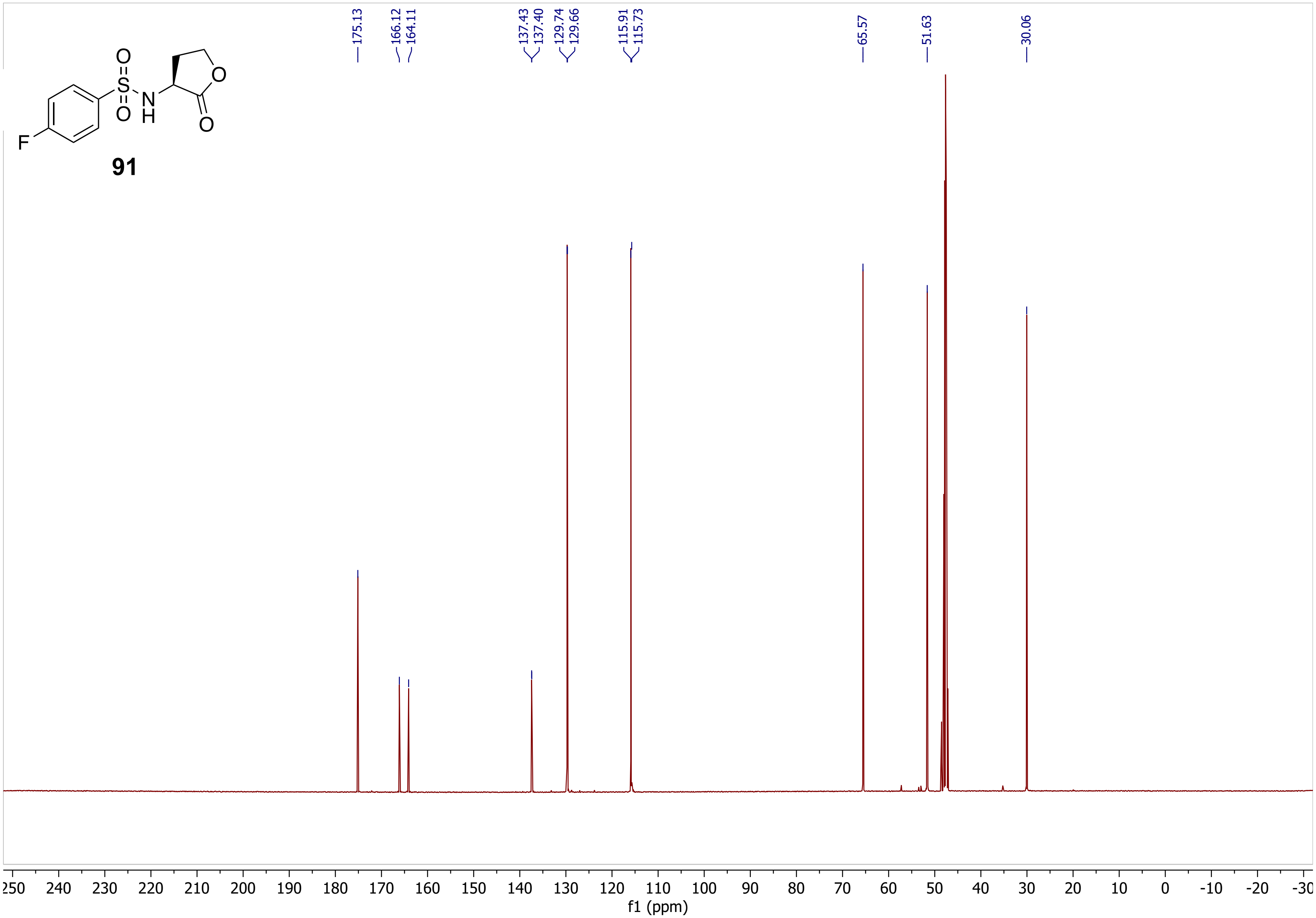

O
S
N
H
O
O
O
F
91
175.13
166.12
164.11
137.43
137.40
129.74
129.66
115.91
115.73
65.57
51.63
30.06
250 240 230 220 210 200 190 180 170 160 150 140 130 120 110 100 90 80 70 60 50 40 30 20 10 0 -10 -20 -30
f1 (ppm)

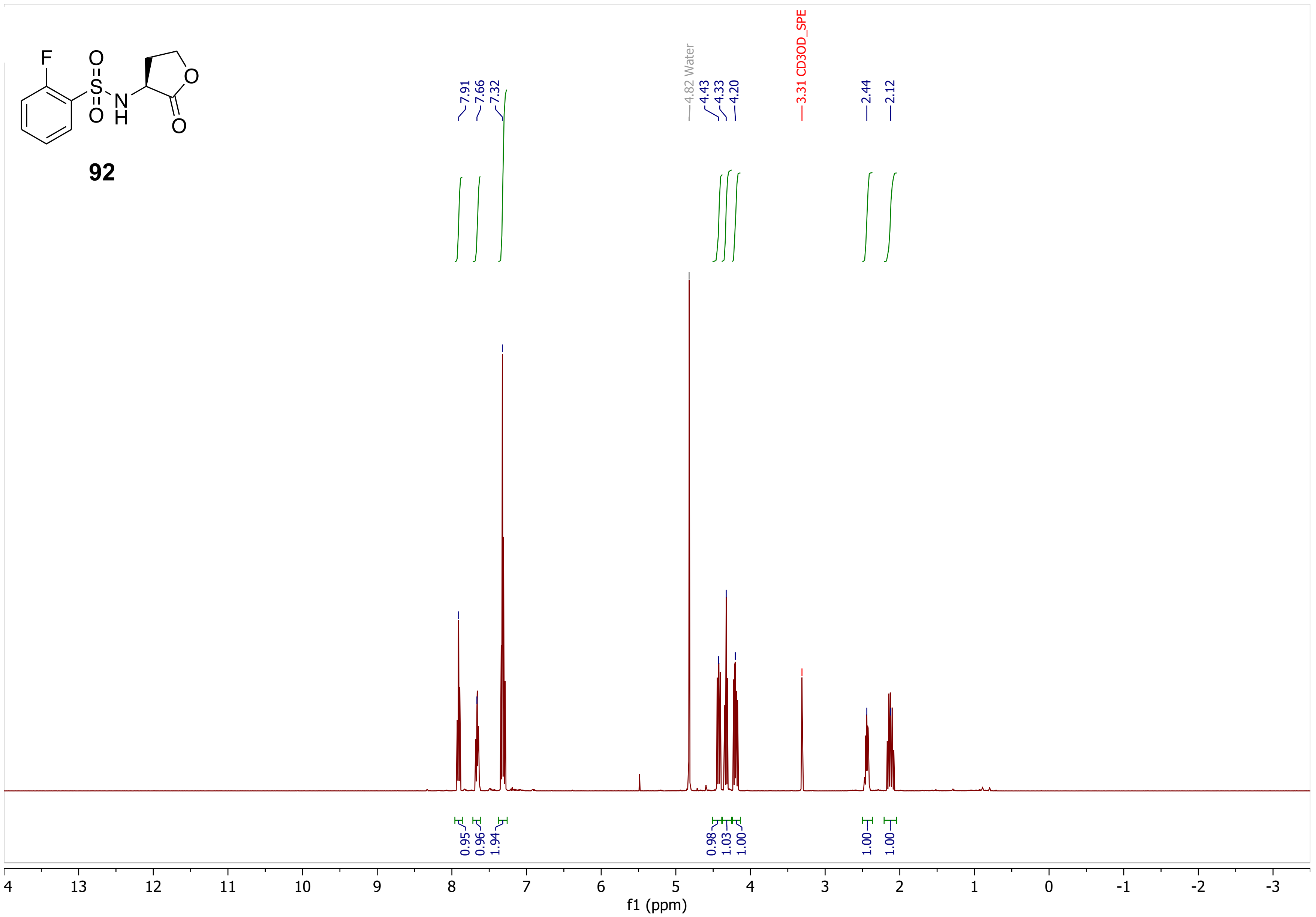
92
4.82 Water
3.31 CD3OD_SPE
7.91
7.66
7.32
4.43
4.33
4.20
2.44
2.12
0.95
0.96
1.94
0.98
1.03
1.00
1.00
1.00
f1 (ppm)

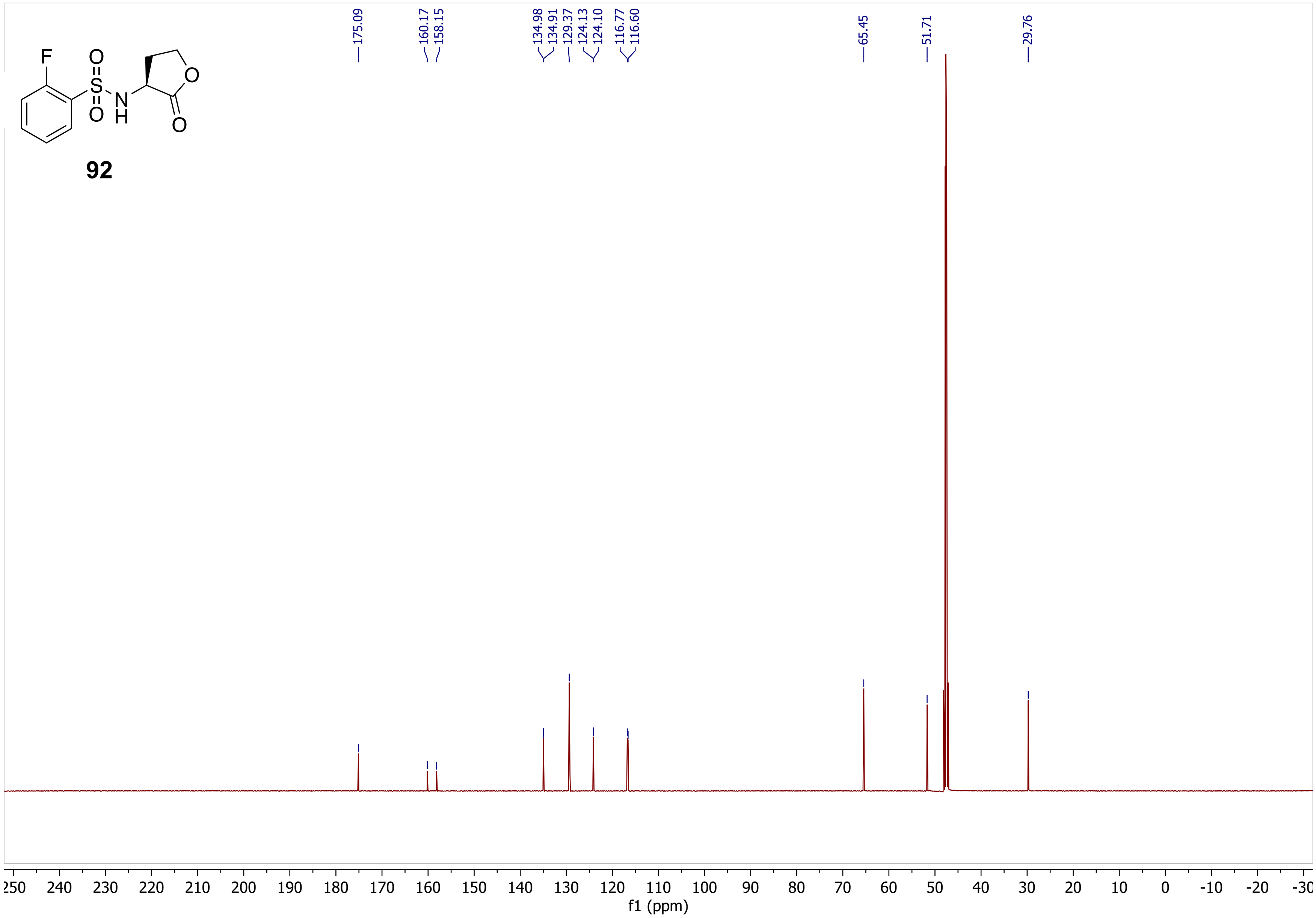
F
O
S
O
N
H
O
O
92
175.09
160.17
158.15
134.98
134.91
129.37
124.13
124.10
116.77
116.60
65.45
51.71
29.76
250 240 230 220 210 200 190 180 170 160 150 140 130 120 110 100 90 80 70 60 50 40 30 20 10 0 -10 -20 -30
f1 (ppm)

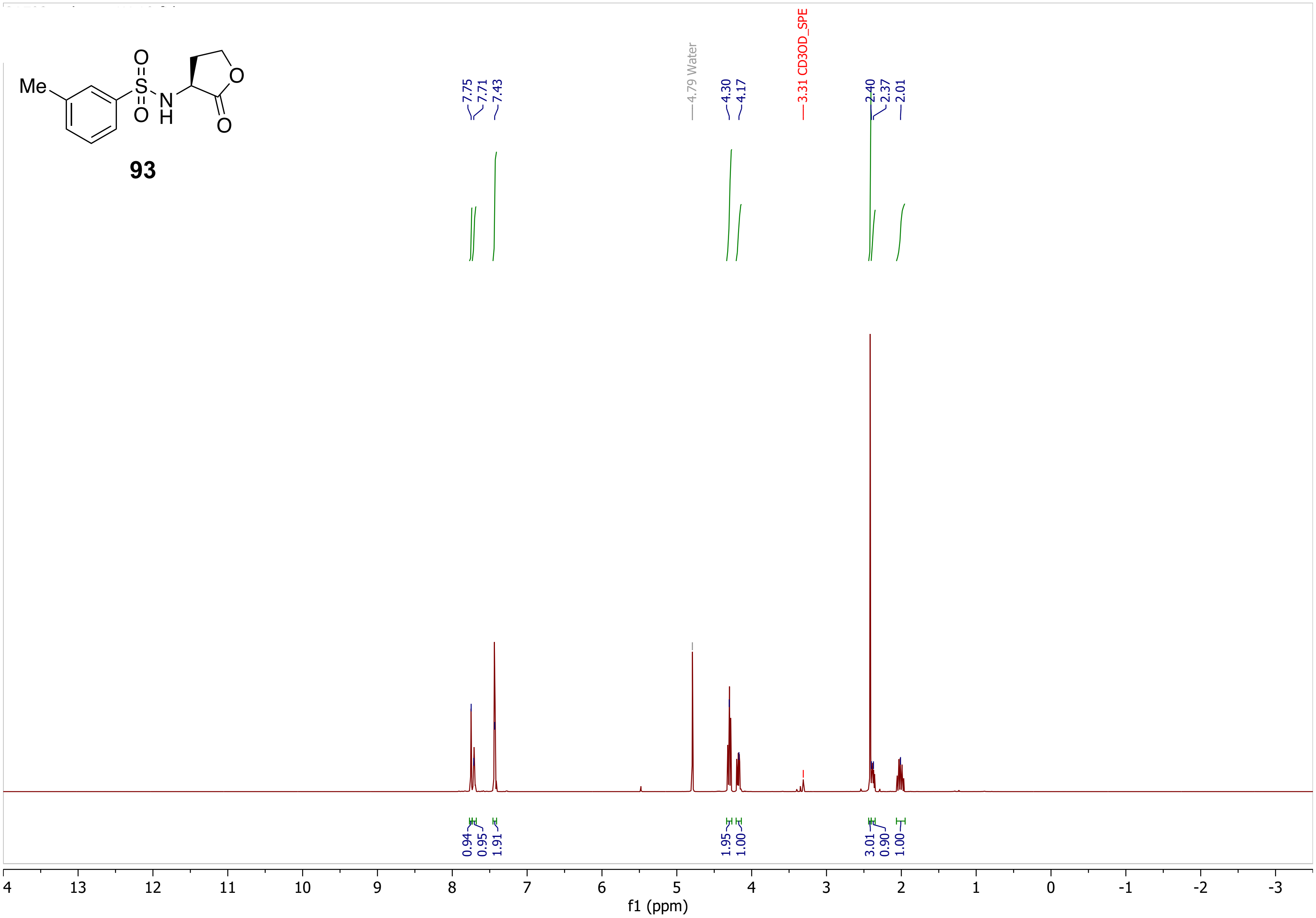
Me
O
S
O
N
H
O
O
93
7.75
7.71
7.43
4.79 Water
4.30
4.17
3.31 CD3OD_SPE
2.40
2.37
2.01
0.94
0.95
1.91
1.95
1.00
3.01
0.90
1.00
f1 (ppm)

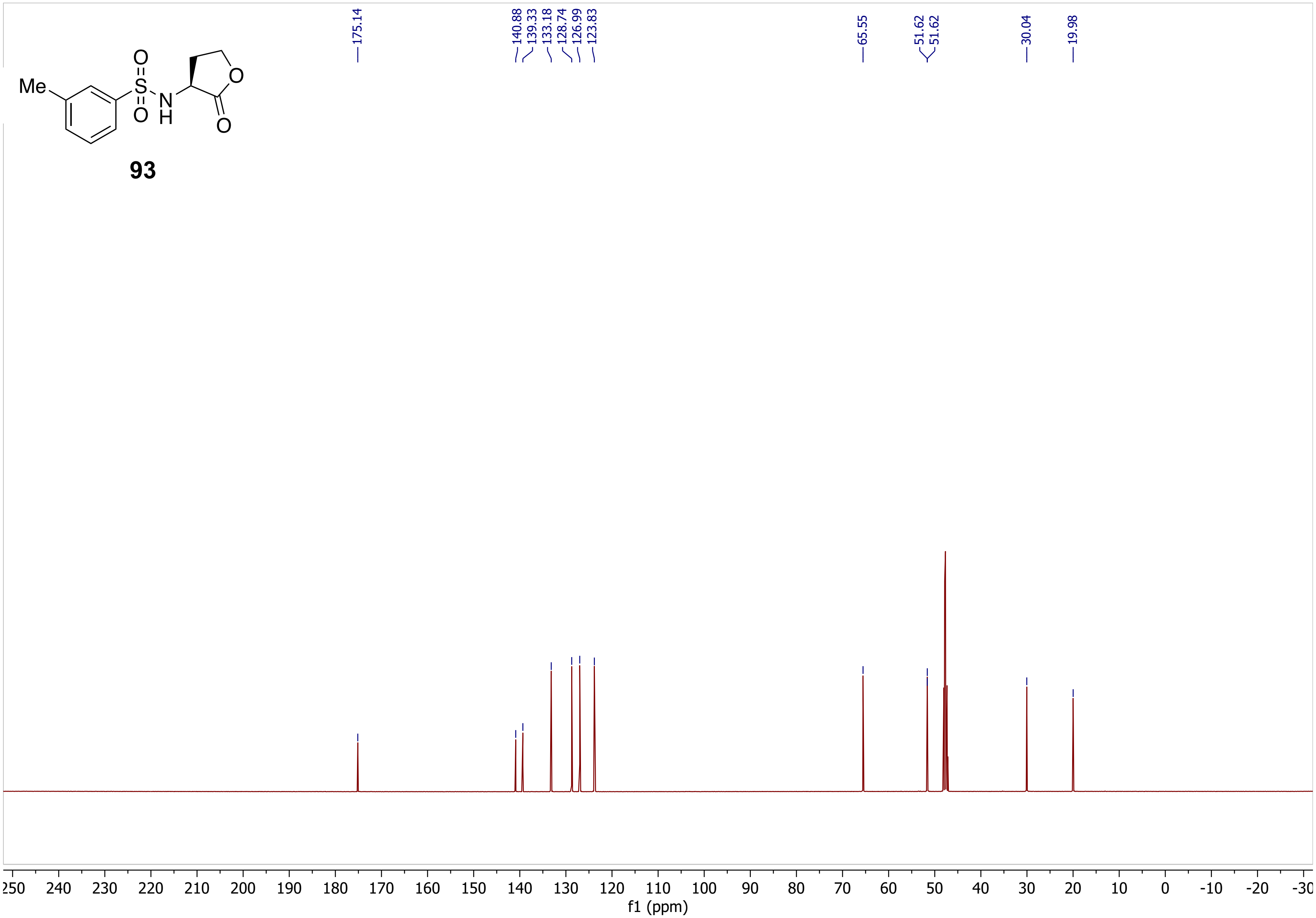
Me
O
S
O
N
H
O
O
93
175.14
140.88
139.33
133.18
128.74
126.99
123.83
65.55
51.62
51.62
30.04
19.98
250 240 230 220 210 200 190 180 170 160 150 140 130 120 110 100 90 80 70 60 50 40 30 20 10 0 -10 -20 -30
f1 (ppm)
