# Supplementary material for: Correction to “Sulfonyl Homoserine Lactones are Tunable Probes to Inhibit the Quorum Sensing Receptor RhlR and Reduce Swarming Motility in Pseudomonas aeruginosa”
Source: ACS Infect Dis. 2025 Nov 5;11(11):3377–8. doi: 10.1021/acsinfecdis.5c00927 (PMC12624830; doi:10.1021/acsinfecdis.5c00927)
Supplement: Supplementary file 1 [file id5c00927_si_001.pdf]

Correction to “Sulfonyl Homoserine Lactones are Tunable Probes to Inhibit the Quorum Sensing Receptor RhlR and Reduce Swarming Motility in *Pseudomonas aeruginosa*”

Guadalupe Aguirre-Figueroa, Diana A. Morales Mijares, Isabel D. Cannell, Irene M. Stoutland, and Helen E. Blackwell*

JOURNAL CITATION

ACS Infect. Dis. **2025**, *11*, 2836−2846.

DOI

10.1021/acsinfecdis.5c00542

CORRECTION TEXT

There are errors in two structures in the Supporting Information. The structures of compounds **38** and **71** are incorrect in Figure S1. The correct structure for **38** lacks the benzyl carbon and is shown in the corrected Figure S1 below. The correct structure of **71** contains a 4-chloro group instead of a 4-fluoro group and is shown in the corrected Figure S1 below. Neither of these errors alter the conclusions of the study.

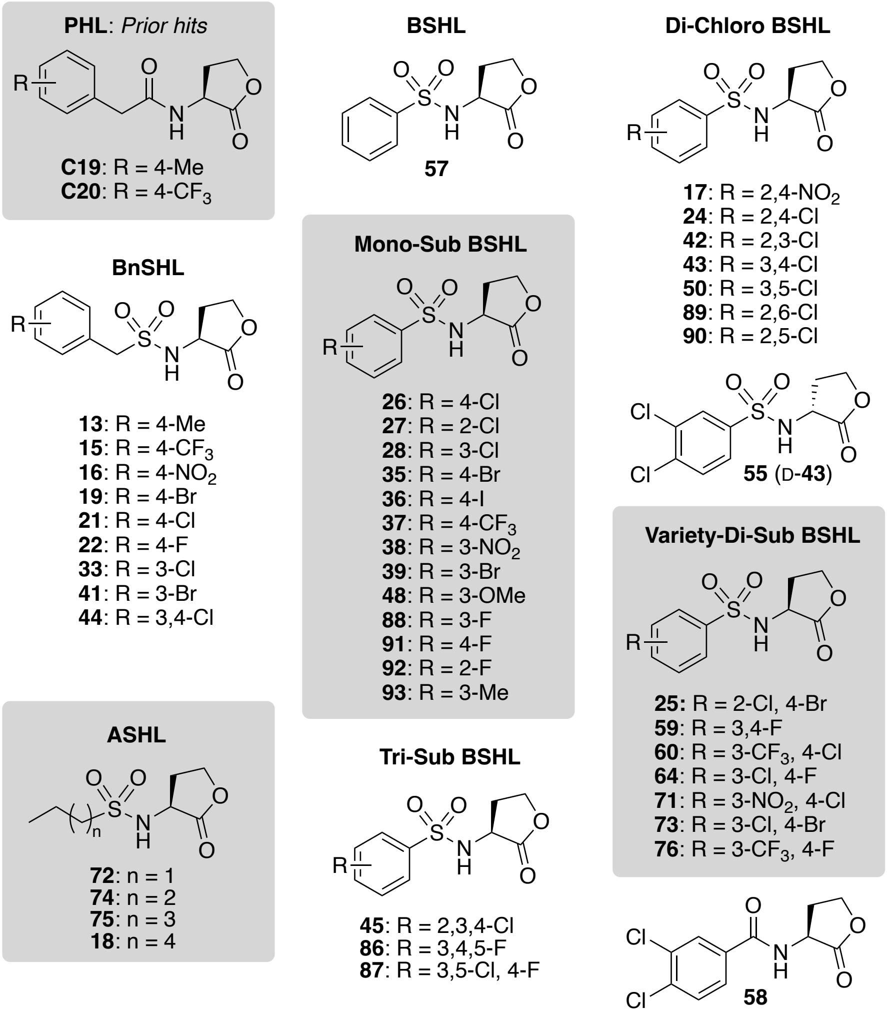

Figure S1. Structures of prior RhlR modulators and all the compounds synthesized in this study. Bioactivity data for compounds **17**, **25**, **38**, **41**, and **58** in RhlR not included in this report.

SUPPORTING INFORMATION CONTENTS

Full details of experimental methods, compound synthesis and characterization, structures of additional compounds, dose-response activity curves, and computational docking analyses.
